# Genomics of Neotropical biodiversity indicators: Two butterfly radiations with rampant chromosomal rearrangements and hybridization

**DOI:** 10.1073/pnas.2410939122

**Published:** 2025-07-28

**Authors:** Eva S. M. van der Heijden, Karin Näsvall, Fernando A. Seixas, Carlos Eduardo Beserra Nobre, Artur Campos D. Maia, Patricio Salazar-Carrión, Jonah M. Walker, Daiane Szczerbowski, Stefan Schulz, Ian A. Warren, Kimberly Gabriela Gavilanes Córdova, María José Sánchez-Carvajal, Franz Chandi, Alex P. Arias-Cruz, Nicol Rueda-M, Camilo Salazar, Kanchon K. Dasmahapatra, Stephen H. Montgomery, Melanie McClure, Dominic E. Absolon, Thomas C. Mathers, Camilla A. Santos, Shane McCarthy, Jonathan M. D. Wood, Gerardo Lamas, Caroline Bacquet, André Victor Lucci Freitas, Keith R. Willmott, Chris D. Jiggins, Marianne Elias, Joana I. Meier

**Affiliations:** ^a^Tree of Life Programme, Wellcome Sanger Institute, Hinxton CB10 1SA, United Kingdom; ^b^Department of Zoology, University of Cambridge, Cambridge CB2 3EJ, United Kingdom; ^c^Department of Organismic and Evolutionary Biology, Harvard University, Cambridge 02138, MA; ^d^Graduate Program in Animal Biology, Federal University of Pernambuco, Recife 50740-570, Brazil; ^e^Graduate Program in Plant Biology, Federal University of Pernambuco, Recife 50670-901, Brazil; ^f^Institute of Organic Chemistry, Technische Universität Braunschweig, Braunschweig 38023, Germany; ^g^Universidad Regional Amazónica Ikiam, Tena 150101, Ecuador; ^h^Biology Program, Faculty of Natural Sciences, Universidad del Rosario, Bogotá 111221, Colombia; ^i^Department of Biology, University of York, York YO10 5DD, United Kingdom; ^j^School of Biological Sciences, University of Bristol, Bristol BS8 1TQ, United Kingdom; ^k^Laboratoire Écologie, Évolution, Interactions des Systèmes Amazoniens, Université de Guyane, CNRS, Institut Français de Recherche pour l’Exploitation de la Mer, Cayenne 97300, France; ^l^Museo de Historia Natural, Universidad Nacional Mayor de San Marcos, Lima Lima11, Peru; ^m^Smithsonian Tropical Research Institute, Gamboa 0843-03092, Panama; ^n^Laboratório de Ecologia e Sistemática de Borboletas, Instituto de Biologia, Universidade Estadual de Campinas, Campinas, CEP 13083-862, Brazil; ^o^McGuire Center for Lepidoptera and Biodiversity, Florida Museum of Natural History, University of Florida, Gainesville, FL 32611-32710; ^p^Instituto Nacional de Biodiversidad, Quito 170506, Ecuador; ^q^Institut de Systématique Évolution Biodiversité, CNRS, Muséum National d’histoire naturelle, École Pratique des Hautes Études, Sorbonne Université, Université des Antilles, Paris 75005, France; ^r^Centre Interdisciplinaire de Recherche en Biologie, Collège de France, CNRS, Institut National de la Santé et de la Recherche Médicale, Paris 75005, France

**Keywords:** speciation, biodiversity, butterflies, Neotropics, genomics

## Abstract

A central question in evolutionary biology is what drives the diversification of lineages. Rapid, recent radiations are ideal systems for this question because they still show key morphological and ecological adaptations associated with speciation. While most research on recent radiations focuses on those occurring in insular environments, less attention has been given to continental radiations with complex species interactions. Here, we study the drivers of continental radiations of *Melinaea* and *Mechanitis* butterflies (Nymphalidae: Ithomiini), which have rapidly radiated in the continental Neotropics. They are classical models for Amazonian biogeography and color pattern mimicry and have been proposed as biodiversity indicators. We generated reference genomes for five species of each genus and whole-genome resequencing data of most species and subspecies covering a wide geographic range to assess phylogeographic relationships, hybridization patterns, and chromosomal rearrangements. Our data help resolve the classification of these taxonomically challenging butterflies and reveal very high diversification rates. We find rampant evidence of historical hybridization and putative hybrid species in both radiations, which may have facilitated their rapid diversification by enriching the genetic diversity. Moreover, we identified dozens of chromosomal fusions and fissions between congeneric species that have likely expedited reproductive isolation. We conclude that interactions between geography, hybridization and chromosomal rearrangements have contributed to these rapid radiations in the highly diverse Neotropical region. We hypothesize that rapid radiations may be spurred if repeated periods of geographic isolation are combined with lineage-specific rapid accumulation of incompatibilities, followed by secondary contact with some gene exchange.

## Significance

Understanding factors contributing to rapid speciation is a key aim of evolutionary biology. Here, we focus on two rapid radiations of Neotropical butterflies. Our genomic data with broad taxonomic and geographic coverage reveal widespread hybridization and rampant chromosomal rearrangements, each likely contributing to the high diversification rates. Our study highlights the use of genomic data to resolve taxonomically challenging species groups and elucidate drivers of diversification in rapid radiations. We show that for biodiversity hotspots with recent radiations, barcoding is insufficient to characterize species richness due to gene flow and recent speciation. The consideration of introgression and karyotype diversity underlying the dynamical species boundaries stimulates process-oriented taxonomic practice, which has important implications for monitoring and managing biodiversity in vulnerable habitats.

Rapid radiations, where a lineage diversifies into many different species over a short time period, are ideal systems for studying how new species evolve (1, 2). They can be driven both by nonadaptive processes, such as the accumulation of differences during periods of allopatry leading to incompatibilities upon secondary contact, and by adaptive processes such as adaptation to different ecological niches or sexual selection for different traits and preferences (3, 4). Sympatric radiations require some degree of niche differentiation among the species for stable coexistence and sufficient reproductive isolation such that incipient lineages do not merge (2).

While most lineages do not readily radiate even in the face of ecological opportunity, some are particularly prone to rapid radiations and do so repeatedly. We are only starting to understand the factors explaining these lineage-specific differences (5). Most knowledge stems from well-studied radiations that evolved in insular environments with little competition with other species and a relatively simple and geographically limited environment (e.g., Darwin’s finches on the Galapagos Islands, cichlid fishes in lakes or Hawaiian silverswords) (2, 6). However, many rapid radiations evolved on the more complex continents (7–9), and much less is known about drivers underlying their diversification. Reduced competition in insular environments allows niche specialization without being immediately outcompeted when a lineage is not yet well-adapted to its environment. However, competition can be strong in large continental areas such as the hyperdiverse Neotropics, potentially limiting (gradual) ecological speciation. The large and complex environments on continents may provide more opportunity for allopatric or parapatric divergence than small islands. The speed of accumulating incompatibilities in geographic separation may thus be particularly important in rapid radiations on continents.

Here, we study the drivers of diversification in two rapid continental radiations of the Neotropical butterfly tribe Ithomiini. Ithomiine butterflies (Nymphalidae: Danainae, ca. 400 species in 42 genera) are found across Central and South America (10, 11). They constitute a substantial part of the butterfly species assemblage and are regarded as good indicators of spatial patterns of biodiversity in the Neotropics, the most biodiverse area in the world (10, 12, 13). Sequestration of pyrrolizidine alkaloids from Asteraceae and Boraginaceae plants renders most Ithomiini unpalatable (14–18), and their color patterns advertise this unpalatability to predators. They form Müllerian mimicry rings, where locally co-occurring species converge in color patterns, thus sharing the cost of predator education (10, 19, 20). We focus on two ithomiine genera, *Melinaea* and *Mechanitis*, which have diversified fast, with most species younger than a million years (11, 21). The study of these radiations has been hampered by taxonomic challenges. *Melinaea* and *Mechanitis* are among the most taxonomically difficult of Ithomiini, as Fox noted (22): “these insects [are] so thoroughly confusing and so thoroughly confused by my predecessors.” The species do not differ in genital or other morphological characteristics and show substantial intraspecific wing pattern variation and mimicry between species. Barcoding does not reliably distinguish species either (23, 24). As prior studies have only used few or no genetic markers or did not have broad geographic coverage, the taxonomy is still partially unresolved, despite many taxonomic revisions (e.g., refs. 22 and 24–28).

While the exact causes of their rapid radiations are unknown, different contributing factors have been proposed. Ecological adaptation may be relevant as species show differences in microhabitats, host plants, mimicry rings, and altitude, but on the other hand, many species share habitats and host plants, and most species occur in the lowlands (25, 29–32). Ithomiine species also differ in male-specific androconial compounds (chemical compounds secreted from specialized wing scales where the fore- and hindwings overlap), which likely act as pheromones (33–35). As coexisting ithomiine species converge in color patterns, assortative mating likely relies strongly on chemical cues.

Allopatric accumulation of differences could also have played a role in the rapid diversification of Ithomiini, as this could have occurred in different rainforest refugia during climatic oscillations e.g., in the Pleistocene (26, 36), but see refs. 37 and 38) or on opposite sides of geographic barriers such as the Andes. Both climatic refugia and the Andes have been proposed as “speciation pumps” in the Neotropics (39), also for Ithomiini (40), where periods of allopatry followed by secondary contact create favorable circumstances for speciation.

Another factor that might contribute to the diversification of ithomiine butterflies is hybridization. Phylogenetic studies using a limited number of markers have revealed mito-nuclear discordances and paraphyletic taxa in Ithomiini (23, 38). This could be due to limited geographic or genetic resolution, incomplete lineage sorting (ILS) in the rapidly speciating lineages, or introgressive hybridization. While gene flow between sister lineages can homogenize gene pools, opposing speciation, recent studies have shown that sometimes introgressive hybridization from more distant relatives can facilitate rapid diversification by enriching the genetic diversity with novel, potentially adaptive variants or contributing to the origin of new hybrid species (41–45). Admixture has been shown to kickstart adaptive radiation (e.g., refs. 46 and 47), facilitate parallel adaptation (e.g., refs. 44 and 48), and novel adaptations (e.g., ref. 49), but the role in ithomiine diversification is hitherto unknown.

Ithomiine butterflies exhibit extensive chromosome number variation (50), which could also contribute to their rapid diversification. Offspring from parents with different karyotypes may suffer reduced fitness due to mismatch in pairing of homologous chromosomes that results in aneuploidy, meiotic failure, or hybrid sterility (51, 52). Furthermore, chromosomal rearrangements might facilitate divergence in the face of gene flow by reducing recombination, promoting the accumulation of incompatibilities (53) or linking together coadapted variants (54, 55). In *Melinaea* and *Mechanitis* butterflies, chromosome counts range from 13 to 30 (50). Chromosomal rearrangements likely contribute to reproductive isolation, as a cross between two closely related *Melinaea* species with different karyotypes resulted in nearly sterile hybrids (56). However, pervasive intraspecific variation in chromosome counts (50, 56) indicates that not all rearrangements reduce fitness and their role in speciation thus remains an open question.

Here, we generate 10 reference genomes and whole-genome resequencing data of almost all species and many subspecies across their geographic range to resolve taxonomic uncertainties and explore whether geography, introgressive hybridization, or chromosomal rearrangements may have played a role in the rapid diversification of *Mechanitis* and *Melinaea* butterflies.

## Results

### Taxonomic Revision.

**Fig. 1.**
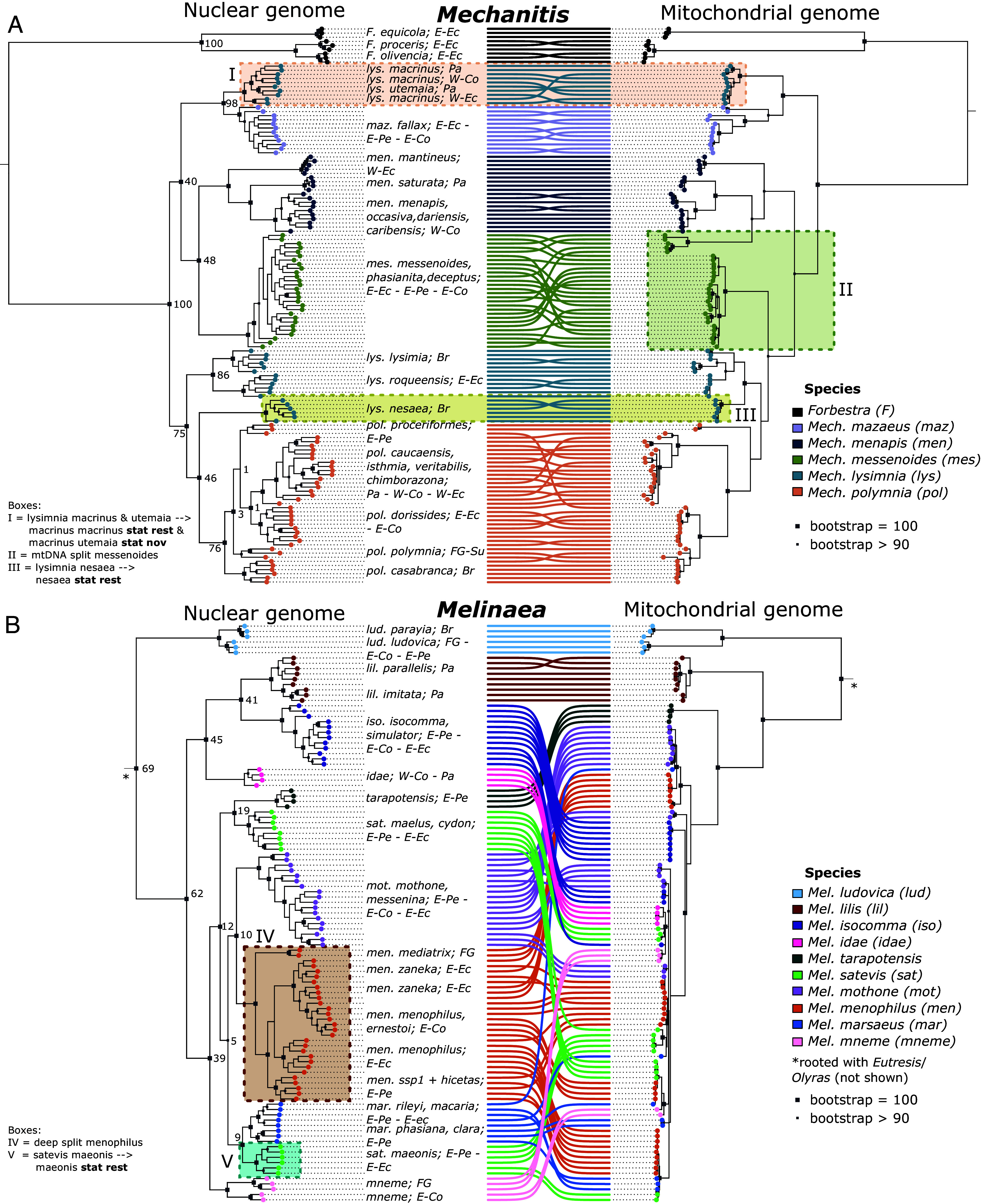
Rampant cytonuclear discordance and a need for taxonomic revision. Cophyloplot showing the nuclear and mitochondrial phylogenies of 135 *Mechanitis* and 109 *Melinaea* individuals. Nuclear phylogeny (*Left*) based on 537,500 sites for *Mechanitis* (*A*) and 784,526 sites for *Melinaea* (*B*) and full mitochondrial genome phylogeny (*Right*). The colored circles and connecting lines indicate the currently classified species (25, 28). Br = Brazil; FG = French Guiana; E-Co = eastern Colombia; W-Co = western Colombia; E-Ec = eastern Ecuador; W-Ec = western Ecuador; E-Pe = eastern Peru; Pa = Panama, Su = Suriname. The colored boxes highlight key findings. The node labels show concordance factors, indicating the percentage of trees produced for windows across the genome that contain that node. Full trees (with root) can be found in *SI Appendix*, Figs. S1–S4.

We adhere to the “genotypic cluster” species concept, which defines a species as “a morphologically or genetically distinguishable group of individuals that has few or no intermediates when in contact with other such clusters” (57). Through whole-genome resequencing of 135 *Mechanitis* and 109 *Melinaea* individuals from across South and Central America, with additional individuals from the outgroup genera *Forbestra*, *Eutresis,* and *Olyras*, we shed light on the phylogenomic relationships and taxonomy (Fig. 1). Our results, laid out in the following sections (and *SI Appendix*, *Text S1*), confirm species vs. subspecies status for most known taxa (in agreement with ref. 28). They support two recent species reclassifications [*Mel. tarapotensis* (56), *Mel. mothone* (58)] and reveal three additional taxa to be elevated to species level [*Mel. maeonis* **stat rest** (Hewitson 1869), *Mec. nesaea* **stat rest** (Hübner 1820), *Mec. macrinus* **stat rest** (Hewitson 1860), Fig. 1 and *SI Appendix*, *Text S1*]. Our data show that the previously uncertain taxon *Mel.* “menophilus/mneme”*mediatrix* (French Guiana) (58) is most closely related to *Mel. menophilus* subspecies, either representing another subspecies of *Mel. menophilus* or its sister species. A revised, annotated taxonomic list for these two genera is given in *SI Appendix*, *Text S2*.

### Mitonuclear Discordance.

In both *Mechanitis* and *Melinaea*, we find rampant mitonuclear discordance, i.e., mismatches between mitochondrial and nuclear phylogenies (IQtree2; maximum likelihood) (Fig. 1). For instance, *Mec. nesaea* is sister to *Mec. polymnia* in the nuclear phylogeny, but to *Mec. lysimnia* in the mitochondrial phylogeny (Fig. 1*A*—box III; *SI Appendix*, Figs. S1 and S2). Such discordance either indicates ILS or admixture (more details below). *Mec. messenoides* harbors two divergent mitochondrial lineages, consistent with previous barcoding results (23, 25) (Fig. 1*A*—box II). One mitochondrial lineage is nested in the mitochondrial clade of *Mec. menapis*, the nuclear sister species of *Mec. messenoides*, whereas the other mitochondrial lineage is sister to the polymnia-lysimnia-nesaea clade. *Mec. messenoides* individuals with different mitochondrial lineages do not form separate clades in the nuclear phylogeny, nor do they differ in collection location or subspecies (*SI Appendix*, Fig. S2 and Table S1).

While the *Melinaea* species are clearly differentiated in the nuclear phylogeny, the mitochondrial phylogeny shows almost no variation among species, extending previous barcoding results (58) (Fig. 1*B* and *SI Appendix*, Figs. S3 and S4). *Mel. ludovica*, *Mel. tarapotensis,* and *Mel. lilis* are the only species that form monophyletic mitochondrial clades and are not part of the shallow clade without species differentiation. Notably, *Mel. ludovica* is the only species that is also an outgroup to the other *Melinaea* species in the nuclear genome, whereas *Mel. lilis* and *Mel. tarapotensis* are sister to *Mel. isocomma* and *Mel. satevis*, respectively.

### Calibrated Phylogenetic Tree.

**Fig. 2.**
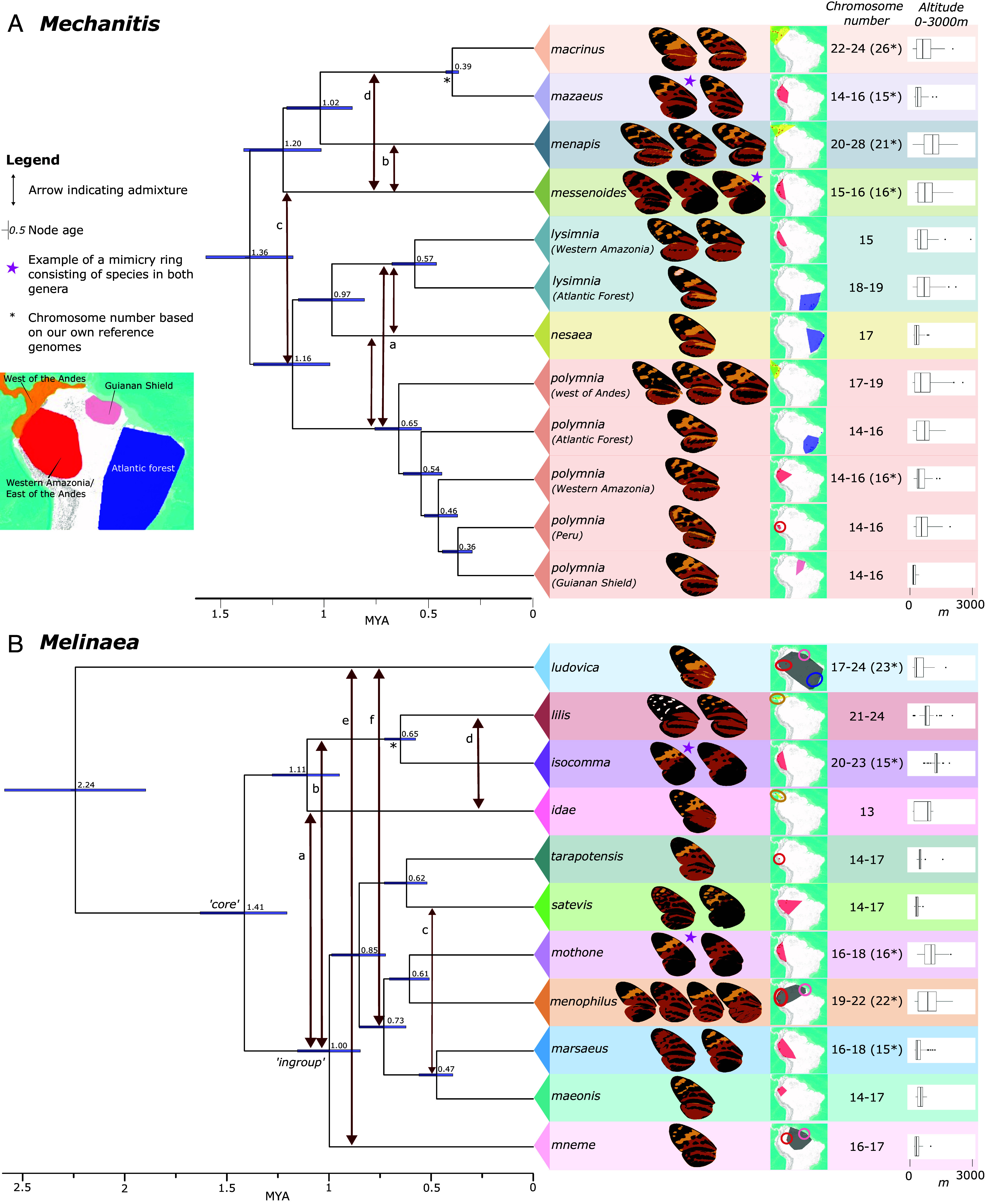
Calibrated phylogeny with evidence of introgression and biogeographic patterns. Time-calibrated BEAST2 phylogenies of *Mechanitis* (*A*) and *Melinaea* (*B*) with the newly proposed species classification and secondary calibrations from ref. 11 (the asterisk indicates which node was used to calibrate). The node labels indicate the age as obtained by the calibration. One individual was included at the species level, or subspecies-level if they were very divergent. Arrows between clades indicate potential hybridization events (based on AIM, Fbranch, and BPP). “Core” in the *Melinaea* phylogeny indicates the core clade of fast diverging *Melinaea*, and “ingroup” is a clade referred to in the text. For each clade, cartoon wings based on representative color patterns are shown, and pink stars exemplify one mimicry ring. The collection location of our individuals is indicated by colored dots and rings on a distribution map based on ref. 10; map from USGS, Esri, TANA, DeLorme, and NPS (*SI Appendix*, Figs. S6 and S7). The region names in the overview map are adapted from ref. 11. Chromosome numbers are based on ref. 50 or our reference genomes (asterisk). The last column contains boxplots of the altitudinal ranges.

We approximated divergence times in the two genera using a Bayesian MCMC method for inferring trees(BEAST2), with one individual representing each lineage to produce a phylogeny calibrated with divergence times from ref. 11 (Fig. 2) (*SI Appendix*, Fig. S5 shows the trees calibrated at a deeper node, which affects divergence time estimates). The seven *Mechanitis* species are estimated to have diversified within the past 1.36 My (Fig. 2*A*), resulting in a speciation rate of 1.431 speciation events per lineage/My (assuming a pure birth model with a constant speciation rate). The 10 species of the core *Melinaea* clade (excluding the most divergent species, *Mel. ludovica*) have diversified in the past 1.41 My (Fig. 2*B*), giving a speciation rate of 1.633. Of the four potential *Melinaea* species missing in our analysis (*Mel. ethra*, *mnasias, mnemopsis,* and *scylax*), two likely form part of the core clade (11, 59), which would increase the speciation rate to 1.672.

However, we note that gene flow may affect our divergence time estimates. Gene flow between sister lineages is expected to shorten their apparent divergence times, and gene flow with nonsister lineages will extend them. Notably, the BEAST2 topologies differ from the IQtree2 topologies at some nodes with low concordance factors in the IQtree2 topologies (Fig. 2 vs. Fig. 1): In the BEAST2 phylogeny *Mec. messenoides* is not sister to *Mec. menapis*, and *Mec. nesaea* is sister to *Mec. lysimnia* instead of *Mec. polymnia*. The relationships among lineages within *Mec. polymnia* also differs between the phylogenies. While ILS could partially explain these discordances, introgression is likely to affect the placement of these taxa (see details below).

### Phylogeographic Patterns.

By combining our sampling locations with those compiled by Doré et al. (10) updated according to our taxonomic revision, we assessed the biogeographic distribution of the species. We identify four main biogeographic regions, with most species restricted to one of them (Fig. 2 and *SI Appendix*, Figs. S6 and S7). Among *Mechanitis* species, some sister species are separated by the Andes: *Mec. messenoides* and *mazaeus* (Western Amazonia) vs. their respective sisters *Mec. menapis* and *macrinus* (West of the Andes). However, the placement of *Mec. messenoides* differs in the BEAST2-topology and is affected by hybridization (see next section). Within *Mec. polymnia*, Ecuadorian and Colombian individuals from opposite sides of the Andes are highly divergent, indicating little gene flow, though our study includes one putative late-generation hybrid (*SI Appendix*, Fig. S8). This pattern of sister species separation by the Andes was not previously as apparent due to species misclassification. Most *Mechanitis* species show overlapping altitudinal ranges, although some show significant differences (Fig. 2 and *SI Appendix*, Fig. S9*A*). For example, *Mec. menapis* occurs at higher elevation compared to all other species, and the Atlantic forest species *Mec. nesaea* occurs at lower elevation than the Atlantic forest subspecies of *Mec. lysimnia* and *Mec. polymnia.*

Most individuals in our *Melinaea* dataset are from Western Amazonia (east of the Andes) and we find that many sister species are sympatric. Only two species in our dataset occur west of the Andes (*Mel. lilis* and *Mel. idae*) and they form a clade with a third species from Western Amazonia (*Mel. isocomma*). Our dataset lacks a potential third species occurring west of the Andes, *Mel. scylax* (10), which may represent a subspecies of *Mel. lilis* (26, 59). Other potential species missing in our dataset are *Mel. mnemopsis* from Western Amazonia, *Mel. ethra* from the Atlantic forest and *Mel. mnasias* from Western Amazonia and the Atlantic Forest (10, 59). There appear to be some altitudinal shifts between clades, with for example *Melinaea isocomma* occurring at higher elevations compared to all other *Melinaea* (Fig. 2 and *SI Appendix*, Fig. S9*B*). Also, six species of the ingroup (excluding *Mel. mneme*) that occur in Western Amazonia vary in altitudinal range.

### Signatures of Rampant Introgression throughout Both Genera.

We find phylogenetic discordance between phylogenies constructed with the nuclear and mitochondrial genomes (Fig. 1), between trees from different genomic windows (concordance factors; Fig. 1), and between the IQtree2 and BEAST2 trees (Fig. 1 vs. Fig. 2). Such discordance is indicative of a history of introgression and/or ILS. We assessed the role of hybridization with windowed species tree inference with BPP (*SI Appendix*, Fig. S10), excess allele sharing between nonsister taxa estimated from Fbranch (*SI Appendix*, Fig. S11) and joint inference of species tree with gene flow using the approximate isolation with migration (AIM) model (*SI Appendix*, Fig. S12). The resulting phylogenies and introgression histories are summarized in Fig. 2, but we stress that this is only one of multiple possible scenarios consistent with our observed patterns of excess allele sharing and gene tree discordance (*SI Appendix*, *Text S3*).

In *Mechanitis*, two lineages show uncertain placements. As mentioned, the placement of *Mec. messenoides* and *Mec. nesaea* varies depending on the methodology (IQtree2 and BEAST2) and genomic regions (Fig. 1*A*—box II and III). The AIM phylogeny allowing for gene flow places *Mec. messenoides* sister to the *polymnia-nesaea-lysimnia* clade rather than with *menapis* and indicates admixture between these lineages (*SI Appendix*, Fig. S12*A*—arrows B-D). An admixed history of *Mec. messenoides* is also supported by Fbranch (*SI Appendix*, Fig. S11*A*—#3) and consistent with its deep mitochondrial divergence, whereby some individuals are mitochondrially sister to the polymnia-nesaea-lysimnia clade, whereas others have a haplotype nested in the mitochondrial clade of *Mec. menapis*. According to the BPP-analysis, *Mec. messenoides* groups with *menapis-mazaeus-macrinus* in ~51% of the genome, and with *polymnia-lysimnia-nesaea* in ~28% of the genome (although in 10% of the trees *messenoides* is sister to *Mec. menapis* and both cluster with *polymnia-lysimnia-nesaea*) (*SI Appendix*, Fig. S10*B*). The branching order within *polymnia-lysimnia-nesaea* is also highly variable across the genome: *Mec. polymnia* and *Mec. nesaea* are sister in 46.3% of the genome, while 31.7% of trees group *polymnia* with *lysimnia*, and 14.7% group *Mec. nesaea* with *Mec. lysimnia* (*SI Appendix*, Fig. S10*C*) (also Fbranch, *SI Appendix*, Fig. S11*A*—#1). In short, these results are consistent with *Mec. messenoides* being admixed and gene flow among *Mec. polymnia, Mec. lysimnia* and *Mec. nesaea*.

In *Melinaea*, IQtree2 and BEAST2 produce the same topology (Fig. 1 vs. Fig. 2), with *Mel. idae* sister to *Mel. lilis* and *Mel. isocomma*. However, AIM groups *Mel. idae* with the ingroup (see label Fig. 2), although showing extensive gene flow from the *lilis*-*isocomma* lineage (Fig. 2*B*—arrow a; *SI Appendix*, Fig. S12*B*—arrow A,D; *SI Appendix*, Fig. S11*B*—#1). To resolve the position of *Mel. idae*, as well as putative introgression between deeper branches of the phylogeny, we ran BPP focusing on the relationships between the *lilis-isocomma-idae* clade and representatives of the ingroup clade (*mneme*, *marsaeus*, *mothone*) (*SI Appendix*, Fig. S10 *E* and *F*). In the majority of the genome (43.4%), the species *lilis, idae,* and *isocomma* form a clade (like IQTree2/BEAST2), while in 34.5% of the genome *Mel. idae* groups with the *mothone-marsaeus-mneme* lineage (like AIM). Notably, the Z chromosome supports almost exclusively the latter relationship. In 4.3% of the genome, *Mel. isocomma* clusters with *mothone-marsaeus-mneme*. *Mel. lilis* is almost as often sister to *Mel. idae* (30.3%) as to *Mel. isocomma* (38.3%), and less commonly an outgroup to both (4.1%), indicating more recent shared ancestry between *Mel. lilis* and *Mel. idae*, which is confirmed by Fbranch excess allele sharing (*SI Appendix*, Figs. S10*F* and S11*B*—#2). *Mel. mothone, Mel. marsaeus,* and *Mel. mneme* also vary in their respective relationships (*SI Appendix*, Fig. S10*E*).

**Fig. 3.**
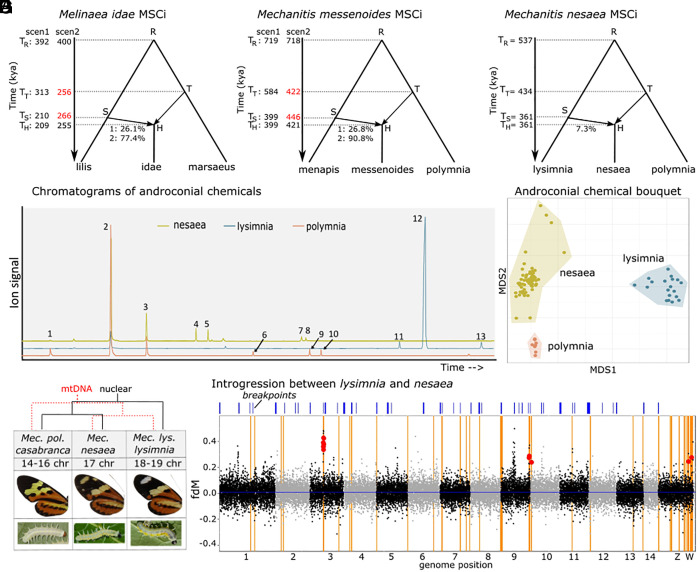
Three ancestrally admixed species, with a focus on *Mechanitis nesaea*. A multispecies coalescent-with-introgression model explored the relation and timing of introgression relative to the divergence times, in (*A*) *Mel. lilis*, *Mel. idae*, and *Mel. marsaeus*; (*B*) *Mec. menapis*, *Mec. messenoides*, and *Mec. polymnia*; and (*C*) the Brazilian *Mec. lysimnia lysimnia*, *Mec. nesaea*, and *Mec. polymnia casabranca*. In (*A* and *B*), several replicates produced different outcomes, indicated with “scen1” and “scen2.” Note that estimated divergence time T_T_ in both second scenarios is more recent than T_S_, contrasting with scenario 1 (indicated in red). (*D*) Overlaid chromatograms of androconial extracts of representative individuals of *Mec. nesaea* (yellow line), *Mec. l. lysimnia* (blue), and *Mec. polymnia casabranca* (orange). Peaks: (1) 4-Hydroxy-3,5,5-trimethylcyclohex-2-enone, (2) Hydroxydanaidal, (3) Methyl hydroxydanaidoate, (4) Methyl farnesoate isomer, (5) Methyl (E,E)-farnesoate, (6) m/z 57, 43, 55, 56, 85, (7) Octadecatrienoic acid (cf.), (8) Octadecanoic acid, (9) Ethyl linolenate, (10) (E)-Phytyl acetate (11) Hexacosene, (12) Heptacosene, (13) Nonacosene (not all compounds of *SI Appendix*, Table S3 are found in these three individuals). (*E*) NMDS shows that the androconial chemical bouquet of *Mec. nesaea* is clearly distinct from sympatric lineages, most similar to *Mec. polymnia*. (*F*) A closer look into the restored species *Mec. nesaea*: phylogenetic relationships, chromosome numbers (50), representative adult and early fifth instar larva (60, 61). The photo of *Mec. lysimnia lysimnia* is courtesy of Augusto Rosa. (*G*) f_dM_ across the genome (20 kb windows) reveals that regions with strong signatures of introgression (f_dM_) between *Mec. nesaea* and *Mec. lysimnia* (P1 = allopatric *polymnia*, P2 = *nesaea*, P3 = *lysimnia*, P4 = *Forbestra*) overlap with regions of high differentiation (F_ST_) between *Mec. nesaea* and its sister species *Mec. polymnia* (orange vertical lines—high F_ST_; red dots—high f_dM_ and high F_ST_). Chromosomal breakpoints between *Mec. polymnia* and the four other reference genomes are shown with blue bars on top.

We further investigated the timing of divergence and introgression for the three species showing the strongest signals of introgression (*Mec. messenoides*, *Mec. nesaea,* and *Mel. idae*), using a multispecies coalescent-with-introgression (MSCi) approach. In all three cases, introgression is estimated to be old (200 to 425 kya) (Fig. 3 *A* and *B* scenario 1; Fig. 3*C*). For *Mec. messenoides* and *Mel. idae,* replicate runs produce different outcomes. Notably, some replicates indicate that the origin of the admixed lineage closely coincides with the split time from both parents, in line with hybrid speciation (Fig. 3 *A* and *B* scenario 2; *SI Appendix*, Fig. S13 and Table S2).

### A Focus on *Mec. nesaea*.

As we propose to re-elevate *Mec. nesaea* to species level from its previous classification as a subspecies of *Mec. lysimnia*, we investigated the relationships and reproductive isolation between *Mec. nesaea*, *Mec. lysimnia,* and *Mec. polymnia* in more detail. We resequenced 15 *Mec. nesaea*, 19 *Mec. l. lysimnia* and nine *Mec. p. casabranca*, of which 24 were sampled from sympatry. Furthermore, we assessed the androconial compounds that might act as pheromones, potentially contributing to assortative mating. Even though *Mec. lysimnia* and *Mec. polymnia* have been observed to interbreed in nature (62) and putative hybrids have been found (*SI Appendix*, Fig. S14), we find relatively high F_ST_ throughout the genome between all three species and no evidence of ongoing gene flow based on ADMIXTURE analyses (*SI Appendix*, Figs. S8 and S15). Furthermore, they differ in the number of chromosomes (Fig. 3*F*) (50), and we find that their androconial bouquets are distinct (Fig. 3 *D* and *E* and *SI Appendix*, Tables S3 and S4 and *Text S4*). All these lines of evidence suggest strong reproductive isolation between *Mec. nesaea* and both *Mec. polymnia* and *Mec. lysimnia.* Genome scans for introgression (f_dM_) between *Mec. lysimnia* and *Mec. nesaea* compared to allopatric *Mec. polymnia* show that this introgression is restricted to few genomic regions and consistent across individuals, indicating ancestral introgression (Fig. 3*G* and *SI Appendix*, Figs. S16 and S17). The MSCi model for *Mec. nesaea* suggests that *Mec. nesaea* had already diverged from *Mec. polymnia* prior to *Mec. lysimnia* introgression. However, *lysimnia*-introgression could have contributed key genetic variation to *Mec. nesaea* and strengthened reproductive isolation to *Mec. polymnia*. Consistent with this hypothesis, we find that *lysimnia*-introgression peaks coincide with peaks of elevated D_xy_ and F_ST_ between *Mec. nesaea* and *Mec. polymnia* (Fig. 3*G*, *SI Appendix*, Figs. S15–S17). However, these regions also show evidence of excess allele sharing between *Mec. nesaea* and sympatric *Mec. polymnia* (*SI Appendix*, Fig. S16), and the low levels of ongoing gene flow make these measures poor predictors of reproductive isolation barriers. More work is thus needed to infer the impact of *Mec. lysimnia* introgression on the speciation of *Mec. nesaea*.

### Chromosomal Rearrangements.

**Fig. 4.**
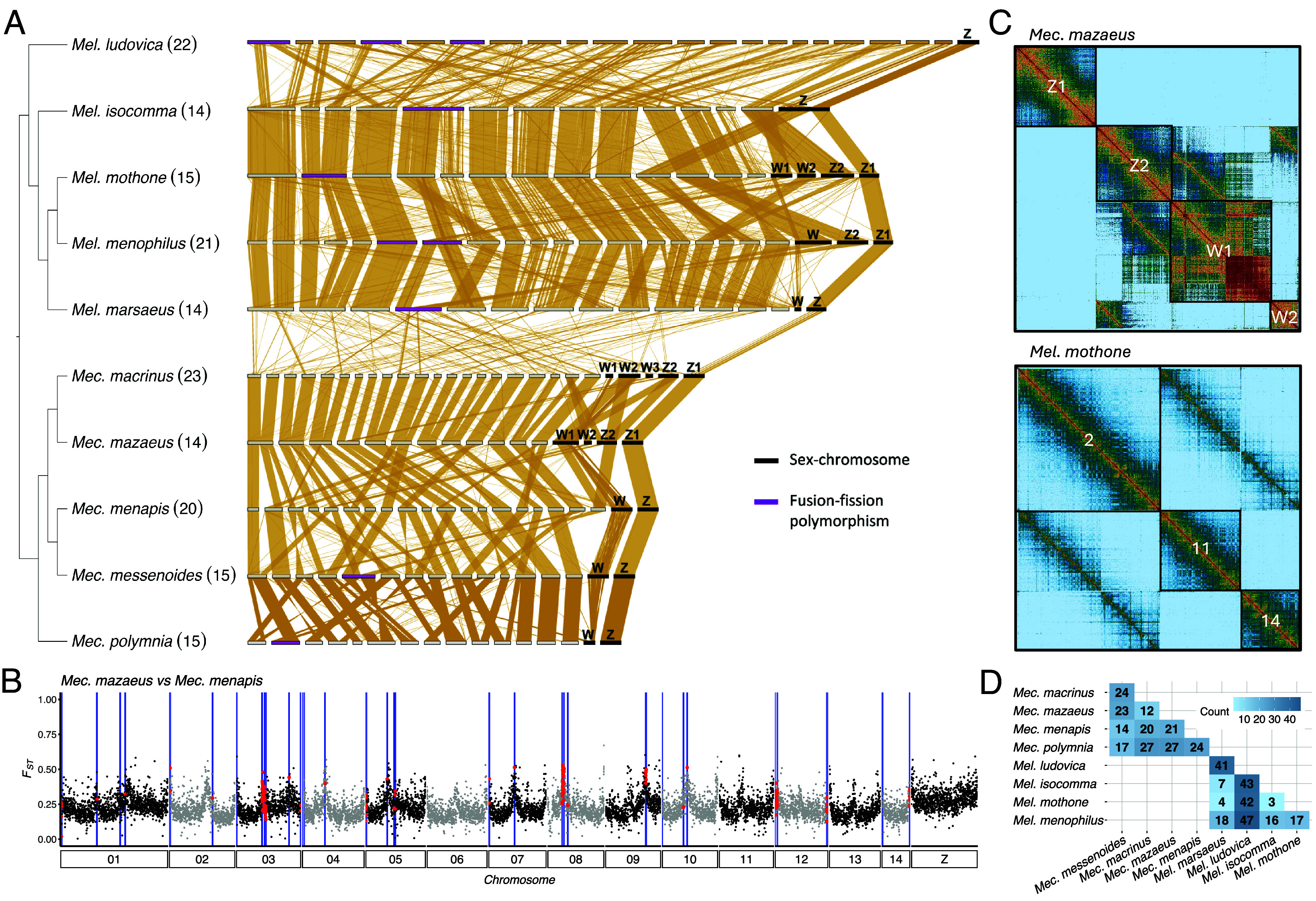
Chromosomal rearrangements. (*A*) Synteny between *Melinaea* and *Mechanitis* chromosomes based on whole genome alignments. Horizontal bars represent individual chromosomes, with sex chromosomes (black bar) and chromosomes involved in within-species fusion–fission polymorphisms (purple bar) highlighted. The cladogram is based on Fig. 2 and shows haploid chromosome numbers in parentheses. (*B*) Example of differentiation (F_ST_) and breakpoints (blue vertical lines) between *Mec. mazaeus* and *Mec. menapis* along the genome. The red dots indicate windows coinciding with breakpoints. (*C*) Examples of HiC-contact maps. *Top* panel: sex chromosomes in *Mec. mazaeus*. *Lower* panel: autosomal fission-fusion heterozygote in *Mel. mothone*. (*D*) Matrix displaying the number of fusion–fission rearrangements between species in each genus.

To study chromosomal rearrangements, we generated haplotype-resolved genomes of five *Melinaea* and five *Mechanitis* species using PacBio and Hi-C data (*SI Appendix*, Fig. S18), resulting in high-quality genomes matching the chromosome counts from karyotyping (50) (*SI Appendix*, Tables S5 and S6). The *Mechanitis* genomes are substantially shorter (291 to 320 Mb) than the *Melinaea* genomes (496 to 661 Mb, *SI Appendix*, Table S5). Whereas most Lepidoptera have conserved karyotypes with 31 chromosomes (63), our synteny analysis shows that *Mechanitis* and *Melinaea* genomes are highly rearranged compared to the ancestral karyotype (*SI Appendix*, Figs. S19 and S20), corroborating earlier findings from two *Melinaea* genomes (64). The median length of conserved syntenic blocks is 32 to 36 genes, compared to 168 genes in *Danaus plexippus*, representing the sister lineage of Ithomiini (*SI Appendix*, Fig. S21 and Table S7). The canonical Z has not undergone any fissions and has retained the longest conserved syntenic blocks (225 to 227 genes; Fig. 4*A* and *SI Appendix*, Fig. S21 and Table S7). However, both genera share a fusion of Z with parts of the ancestral autosome 10 and *Mechanitis* has a further fusion with parts of the ancestral autosome 6 (*SI Appendix*, Fig. S20 and Table S8). In addition, one of the Z chromosomes of the male *Mel. isocomma* is fused with an autosome (*SI Appendix*, Fig. S18 and Table S8). A W chromosome was identified in all females as a segment of varying size depleted of BUSCO genes with moderate sequence similarity within genera, but none between (Fig. 4*A*). We found W-autosome fusions in four species (*SI Appendix*, Table S8). *Mec. macrinus* and *Mec. mazaeus* show complex fusions between the W and multiple autosomes that are partially shared. Four species have two Z chromosomes, as by definition, the homologue of the chromosome fused to the W becomes a Z chromosome. Similarly, three species have multiple W chromosomes. Surprisingly, seven individuals are heterozygous for one or more simple autosomal rearrangements and *Mel. ludovica* even shows a complex chain rearrangement involving four autosomes (*SI Appendix*, Fig. S18 and Table S8). None of these autosomal polymorphisms are shared among species.

While chromosome spreads had previously revealed species differences in chromosome counts (50), we here show that these differences are due to complex rearrangements. Congeneric species show 3 to 47 chromosomal rearrangements (Fig. 4*D*). To assess whether these confer reproductive isolation barriers, we mapped the location of the breakpoints between all species pairs to test for an association between breakpoints and reduced gene flow. We found increased *F*_ST_ in all *Mechanitis* comparisons, and reduced diversity (π) in breakpoint regions in seven of the 10 comparisons (Fig. 4*B* and *SI Appendix*, Fig. S22 and Table S9). In principle, elevated *F*_ST_ could be caused by increased background selection as recombination tends to be reduced at chromosome ends and levels of ongoing gene flow are low (65, 66). However, we also find elevated absolute divergence (D_XY_) in windows coinciding with breakpoints in four of the 10 comparisons, which indicates that background selection alone cannot explain the pattern (67). In *Melinaea*, we did not observe significantly elevated F_ST_ in the breakpoint regions, but we detected an increase in D_XY_ especially in the comparisons involving the more distantly related *Mel. ludovica* (*SI Appendix*, Fig. S23 and Table S9).

## Discussion

Our results confirm that the two ithomiine genera *Melinaea* and *Mechanitis* represent fast and recent radiations. They diverged in the past 1 to 2 My and speciated much faster than the well-studied Neotropical butterfly radiation of *Heliconius* [respectively 1.633 and 1.431 vs. 0.324 speciation events per lineage/My (46 species in 11.8 My)] (68). Kawahara et al. (21) previously found a significant rate shift toward high diversification rates in ithomiine butterflies (clade L; 0.23 speciation events per lineage/My) and we show that among Ithomiini, *Mechanitis,* and *Melinaea* have an even higher speciation rate, corroborating previous results (11). Our results shed light on potential drivers of their rapid diversification.

Given the high number of ancient introgression events across both genera, hybridization may have sped up speciation by boosting genetic variation as seen in other systems (e.g., ref. 44). We identified three species showing ancestral introgression that might have a hybrid origin. Further research on which genomic regions are more or less likely to introgress could inform us as to what extent introgression contributed to reproductive isolation between those taxa and their parental lineages. For instance, similar to *Heliconius* butterflies (69, 70), introgression might have contributed to mimicry ring switches and habitat adaptation.

Despite ancient hybridization, we find little evidence for ongoing gene flow between the species, suggesting strong reproductive isolation. Some of this reproductive isolation is likely attributed to the exceptionally high rates of chromosomal rearrangements across both genera, as complex chromosomal rearrangements are expected to constitute barriers to gene flow (71, 72). While Lepidoptera chromosomes are holocentric and may be able to tolerate simple fusions and fissions during meiosis, most Lepidoptera have retained the same highly conserved karyotypes of 31 chromosomes (63, 73). The massive chromosomal rearrangements we found in Ithomiini are thus unusual, but there are other lineages with high rates of fissions and fusions [e.g., *Leptidea* (74) or *Erebia* (75)]. Variation in chromosome number among species is slightly associated with increased speciation rates across Lepidoptera (76). Also in Ithomiini, the chromosomal rearrangements likely contribute to reproductive isolation, as crosses between two *Melinaea* sister species with different chromosome counts led to nearly sterile hybrids (56).

Nevertheless, many of our genomes showed heterozygosity for chromosomal rearrangements, suggesting that there is no strong selection against them. In principle, genome assembly errors could lead to false inferences of fissions or fusions. However, in this case, the heterozygote fission-fusion polymorphisms are unambiguous in the Hi-C data (*SI Appendix*, Fig. S18), and match the intraspecific variation in chromosome counts documented previously (50) and cytogenetical evidence for pervasive polymorphism in *Mel. satevis cydon* (56). This may be akin to *Leptidea sinapis*, where, despite multiple fusion-fission polymorphisms segregating within populations (74, 77), crosses between chromosomal extremes show low survival of F2 hybrids (78). That complex chromosomal rearrangements involving multiple chromosomes likely constitute stronger barriers to gene flow compared to simple fusions and fissions has also been found in other taxa such as shrews (79) and rock wallabies (80). It is thus possible that simple fusions and fissions are not strongly selected against, but if too many sequential fusions accumulate in isolated populations, they cause reproductive isolation upon secondary contact.

Furthermore, we find many fusions between sex chromosomes and autosomes. Given the commonly disproportionate role of sex chromosomes in speciation (e.g., Haldane’s rule and/or large-Z effect) (81–83), they may play a strong role in reproductive isolation of ithomiine butterflies. We found two Z-autosome fusions shared among all congenerics, but no fission in the canonical Z, consistent with conserved Z chromosomes in other Lepidoptera lineages (73). The heterozygote Z-autosome fusion in *Mel. isocomma* likely represents a recent Z-autosome fusion not (yet) fixed in this species, and thus provides a unique opportunity to study the drivers underlying sex-autosome fusions (84). Simple W-autosome fusions as observed in *Mel. menophilus*, and complex sex-autosome fusions leading to multiple Ws and Zs as in *Mec. macrinus* and *Mec. mazaeus* have also been described in some *Heliconius* (85) and *Leptidea* butterflies (86). They may constitute particularly strong barriers to gene flow (87) and provide opportunities for follow-up studies to identify the causes and dynamics of these neo sex chromosomes.

Many of the evaluated sister species are separated by the Andes or restricted to different geographic regions, which indicates that allopatry may have played a role in speciation in *Melinaea* and *Mechanitis*. In multispecies clades in the same geographic area, we find variation in altitude, indicating a potential role of parapatry. Periods of geographic isolation, which allow the build-up of reproductive isolation between lineages due to drift and local adaptation, followed by secondary sympatry, where reproductive isolation may be consolidated by reinforcement, can facilitate diversification, as proposed in e.g., birds (39), fishes (47), and Andean plants (88). Moreover, the divergence times of *Mechanitis* and *Melinaea* match Pleistocene climatic fluctuations (2.58 Ma to 11.7 ka). The Neotropics is a heterogeneous area with many environmental gradients that limit both historical and contemporary gene flow, which may have been accentuated during climatic fluctuations. The high rates of chromosomal rearrangements in *Mechanitis* and *Melinaea* might accelerate the accumulation of reproductive isolation barriers. On coming together in secondary sympatry, these rearrangements likely cause low hybrid fitness, which might facilitate adaptation to different niches due to low levels of gene flow. Selection against interbreeding might also lead to reinforcement of reproductive isolation via assortative mating, e.g., based on pheromones (18, 89).

Our data suggest that hybridization-derived genetic variation and high rates of chromosomal rearrangements may both have played key roles in the fast diversification of *Melinaea* and *Mechanitis* and potentially contributed to adaptation and assortative mating, facilitating species coexistence. Notably, while an important role of hybridization in diversification has been found in both insular environments [e.g., silverswords (46), cichlids (47)] and continental radiations [e.g., this study, *Heliconius* (90), *Rhagoletis* flies (91)], rapid radiations with high rates of chromosomal rearrangements seem to be restricted to continents [e.g., this study, shrews (79), *Erebia* butterflies (80)]. Due to more opportunities for geographical isolation on large landmasses than in insular environments, factors such as high rates of chromosomal rearrangements expediting the allopatric accumulation of incompatibilities may thus be particularly important in continental radiations.

Our study not only sheds light on drivers of continental radiations but also largely resolves the taxonomy of important biodiversity indicators. Hitherto, the study of these radiations has been hampered by taxonomic challenges, whereas our combination of whole-genome resequencing with vast taxonomic and geographic coverage, genome assemblies, and androconial chemical analysis allowed us to resolve taxonomic issues. Our study confirms that DNA barcoding can be misleading, massively underestimating species richness, and should only be used with care to assess biodiversity. The taxonomic implications of both introgression and karyotype diversity for species delimitation and designation of conservation units are important to consider during monitoring and management of biodiversity in these vulnerable habitats.

## Materials and Methods

### Collecting Butterfly Specimens.

157 specimens of *Mechanitis*, 9 *Forbestra*, 109 *Melinaea,* 1 *Eutresis,* and 1 *Olyras* were collected over the years 2000-2023 across Central and South America (*SI Appendix*, Table S1). Adult butterflies were caught with a net, and their bodies were subsequently preserved in ethanol, DMSO, or flash-frozen and stored at −70 °C. Moreover, legs from dried museum specimens were included (Florida Natural History Museum; Natural History Museum London). Wings were photographed and stored in envelopes. The resulting dataset covers almost all species of *Melinaea* and *Mechanitis* from a wide geographical range. In addition, 65 individuals of *Mec. nesaea*, 19 *Mec. lysimnia lysimnia* and 8 *Mec. polymnia casabranca* were collected for the androconial chemical analysis (*SI Appendix*, Table S3).

### DNA Extractions and Whole Genome Resequencing.

DNA extractions were done with either the Qiagen MagAttract High Molecular Weight kit (Qiagen ID 67563), or the Qiagen QiaAmp DNA mini kit (51304), or a PureLink digestion and lysis step followed by a magnetic bead DNA extraction (92). The dried museum specimens were extracted using a Lysis-C buffer and a MinElute DNA extraction kit (protocol adapted from ref. 93; Qiagen ID 28006). Library preparations were performed using homemade TN5-transposase-mediated tagmentation (protocol adapted from ref. 94), or following the manufacturer’s guidelines with the Illumina DNA PCR-free library prep kit and sequenced (150 bp paired-end) on Illumina NovaSeq 6000 or NovaSeq X machines at Novogene or the Wellcome Sanger Institute.

### Reference Genomes.

Haplotype-level chromosomally resolved reference genomes were assembled for five species each of *Mechanitis* and *Melinaea* (*SI Appendix*, Table S5). Earlier versions of two *Melinaea* genomes were published previously (64). In short, we combined 12-57x PacBio HiFi sequencing and 33-197x Illumina sequencing of Hi-C libraries (haploid coverages, *SI Appendix*, Table S5) and assembled the genomes according to the Tree of Life pipelines (https://github.com/sanger-tol/genomeassembly) (*SI Appendix*, *Text S5*).

### Whole Genome Mapping.

To prepare the whole genome data for analysis, read quality was checked with FastQC (v0.11.9) (95). Sequences below 50 bp were discarded and adapters and PolyG-tails were trimmed with FastP (v0.23.2) (96), before they were aligned to *Melinaea marsaeus* (64) or *Mechanitis messenoides* using BWA-mem (v.0.7.17) (97). Picard removed PCR duplicates (v3.0.0) (98). Samtools (v1.17) (99) and GATK3 HaplotypeCaller (v3.8.1.0) (100, 101) were used for variant calling, with a minimum base quality score of 20.

VCFtools (v0.1.16) (102) was used for filtering. Based on the distribution of sequencing depth (mean *Melinaea*: 7; *Mechanitis*: 15), all sites with a mean depth below 3 (*Melinaea*) or 5 (*Mechanitis*), and above 15 (*Melinaea*) or 30 (*Mechanitis*) were removed. Insertions, deletions, sites with >50% missing data, as well as genotypes with a depth below 2 (*Melinaea*) or 3 (*Mechanitis*) were removed. The mitochondrial DNA was filtered separately, with a maximum depth of 1,700 (*Melinaea*) or 1,200 (*Mechanitis*).

### Phylogenetic Analyses.

For each genus, we inferred a phylogenetic tree based on a filtered subset of the whole genome sequence data (including monomorphic sites, thinned to 1 in 500 sites, with a minimum genotype quality of 10). Our filtered VCF-files were converted to phylip with a custom script which represents heterozygous genotypes with “ambiguity codes” (equal likelihood for both alleles) to generate one sequence per individual (vcf2phylip.py, http://www.github.com/joanam/scripts), and subsequently, IQtree2 (v2.1.2) (103) produced phylogenetic trees with ultrafast bootstrap approximation (-B 1000; UFBoost) (104) and the GTR-model.

We inferred separate phylogenies for mitochondrial and nuclear DNA. The nuclear trees are based on 537,500 sites for *Mechanitis* and 784,526 sites for *Melinaea*. The mitochondrial phylogenies are based on the full mitochondrial genome (not thinned), including 11,818 bp for *Mechanitis* and 11,815 bp for *Melinaea.* For *Mechanitis*, we included a maximum of six individuals of the same subspecies and country in the phylogenetic analyses, thus excluding several Brazilian *Mec. polymnia*, *Mec. nesaea* and *Mec. lysimnia*. These individuals were included in hybridization analyses. For *Melinaea*, all individuals were used.

In addition, a phylogenetic tree calibrated with divergence times from ref. 11 was produced using BEAST2 (105) (https://beast2-dev.github.io/beast-docs/beast2/DivergenceDating/DivergenceDatingTutorial.html) with the nuclear phylogenetic dataset thinned to 1 in 5,000 sites and with each lineage represented by the individual with highest coverage. Divergence times between *Mec. mazaeus* and *Mec. macrinus* (0.39 MYA; Fig. 2) as well as between *Mechanitis* and *Forbestra* (5.22 MYA; *SI Appendix*, Fig. S5*A*) were used for calibration, with the prior picked from a log normal distribution with a SD of 0.04 (mazaeus-macrinus) and 0.1 (Forbestra). For *Melinaea*, divergence between *Mel. isocomma* and *Mel. lilis* (0.65 MYA; Fig. 2; SD 0.06) as well as *Mel. ludovica* and the core clade (4.66 MYA; *SI Appendix*, Fig. S5*B*; SD 0.1) were used. We used the HKY gamma-4 site model with a strict molecular clock and the Calibrated Yule model. The “clockRate” and “birthRateY” were set to “gamma,” with an alpha of 0.001 and a beta of 1,000. The model was run for 15,000,000 chains, stored every 5,000 trees. For *Melinaea*, the calibration with the deep node (*SI Appendix*, Fig. S5*B*) did not converge, also not using an “optimized relaxed molecular clock.”

Phylogenies were visualized using the packages “ape” (v5.7-1) and “phytools” (v2.1-1) in R (106, 107) and FigTree (v1.1.4) (http://tree.bio.ed.ac.uk/software/figtree/). To calculate a minimum constant speciation rate (λ) we assumed a fully symmetric tree and a pure birth model, with n as the number of species and T the time from root to tip: $n=e^{\lambda *T}$ which gives λ=ln(n)/T.

### Distribution Maps.

The coordinates of our individuals and the individuals from ref. 10 were plotted using the libraries “sf,” “tmap,” “tmaptools” and “mapview” in R (108–110). We classified the subspecies of ref. 10 into species following our taxonomic revision. Our individuals were plotted as dots on top of the overall distribution. The basemap “Esri.WorldTerrain” was used (ESRI, ARCGIS; https://github.com/leaflet-extras/leaflet-providers); *SI Appendix*, Figs. S6 and S7 has maps including the attribution.

Elevations were extracted from the coordinates using e.g., “curl https://api.opentopodata.org/v1/srtm30m?locations=-7.405277778,-75.8197222” and altitudinal ranges (no outliers; IQR ± 1.5*IQR) were calculated for each clade. For each genus an ANOVA followed by a Tukey HSD test was performed to explore the statistical significance of altitudinal distribution (*SI Appendix*, Fig. S9).

### Window Trees.

IQtree2 was used to produce phylogenetic trees in windows across the genome (20 kb windows 200 kb apart) and to calculate gene concordance factors between these window-trees and the whole-genome phylogenies (111).

### DSuite.

We explored excess allele sharing between species or divergent subspecies using DSuite (112). We pruned our variable sites to 1 in 100 sites. Dtrios calculated D and f4-ratio statistics for all trios and Fbranch then summarized them as f-branch statistics using a species tree based on the nuclear phylogeny of Fig. 2 (*SI Appendix*, Table S1). The results were visualized with a python script provided by DSuite (112).

### AIM with StarBEAST2.

We followed the Approximate Isolation with Migration (AIM) in StarBEAST2 tutorial to obtain a phylogenetic tree with admixture arrows (105, 113). For each genus, we picked the highest-coverage individual per species, and randomly extracted 150 (*Mechanitis*) or 200 (*Melinaea*) 800 to 1,000 bp windows across the genome. We used the HKY gamma-4 site model with a strict molecular clock and the Yule model. The migration rate was set to 25 with an initial value of 0.1, and we ran the model for 100,000,000 chains, storing every 5,000 trees. We updated the parameters according to the suggestions in the output of the first run and reran the model to improve convergence.

### Species-Tree Inference.

Variation in phylogenies across the genome was inferred using the multispecies coalescent (MSC) approach implemented in BPP v.4.6.2 (114), while accounting for ILS. For each species, the highest-coverage individual was used. To minimize the effect of linked selection, loci were required to be at least 2 kb from annotated exons. Because the analysis assumes no intralocus recombination and independence between loci, we selected loci of 300 bp at least 2 kb apart. Sequence alignments were produced for all loci, masking repetitive elements annotated in the reference genome using RepeatMasker v4.1.5 (http://repeatmasker.org/). Loci where at least one individual showed more than 80% missing data were excluded. Furthermore, sites with missing genotype calls were removed and loci with less than 30 bp passing filters were excluded. Heterozygous sites were assigned IUPAC codes. Loci were grouped into blocks of 100 loci. Species-tree estimation was then performed in BPP v.4.6.2 using the A01 analysis (species-tree inference assuming no gene flow). Inverse gamma priors (invGs) were applied both to the root age (τ0) and to effective population sizes (θ)—invG(3, 0.06) and invG(3, 0.04), respectively. Parameters were scaled assuming a mutation rate of 2.9 × 10^−9^ substitutions per site per generation and four generations per year. The MCMC was run for 1,000,000 iterations after 50,000 iterations of burn-in, sampling every 10 iterations.

### Demographic Modeling.

For the three admixed species *Mel. idae, Mec. messenoides,* and *Mec. nesaea*, we ran a multispecies-coalescent-with-introgression (MSCi) model implemented in BPP v.4.6.2 (114) to better estimate their position in the phylogeny and divergence timing while accounting for admixture. For *Mel. idae,* we considered *Mel. lilis* and *Mel. marsaeus* as sister/donor species, while for *Mec. messenoides*, *Mec. menapis,* and *Mec. polymnia* (West) were chosen. For *Mec. nesaea*, we used one individual of each of the Brazilian populations (*Mec. nesaea, Mec. lysimnia lysimnia*, *Mec. polymnia casabranca*). 2 kb loci were selected from autosomes, and required to be at least 2 kb apart from annotated exons and 10 kb from the nearest locus. For each locus, individuals with more than 50% missing data and sites containing missing genotypes or overlapping annotated repetitive elements were removed. Only loci at least 800 bp after filters and without missing individuals were considered. Heterozygous sites were assigned IUPAC codes. Demographic parameters were estimated using a fixed species tree with introgression events (*SI Appendix*, Fig. S13). An inverse gamma prior (invG) was applied for all population size parameters θ (α = 3; β = 0.04) and root age parameter τ (α = 3; β = 0.06). A beta prior was applied to the introgression probability (ϕ) (α = 1; β = 1). We performed six replicate MCMC runs of 1,000,000 iterations, after a burn-in period of 50,000 iterations, sampling every 50 iterations. Divergence time was calculated based on 4 generations per year, and a mutation rate of 2.90 × 10^−9^ as in *Heliconius* (45) (T = τ/mutation rate/generations/million years).

### Genome Scans, ADMIXTURE, TriangulaR.

F_ST_ (relative divergence), D_XY_ (absolute divergence), and π (diversity) were calculated in all species pairs in 20 kb windows across the genome (including monomorphic sites, with a minimum of 10,000 sites per window) using popgenWindows.py (https://github.com/simonhmartin/genomics_general). To examine gene flow between *Mec. polymnia, Mec. nesaea* and *Mec. lysimnia*, f_dM_ was calculated using ABBABABAwindows.py (https://github.com/simonhmartin/genomics_general) (115). We tried both *Forbestra* and *Mec. messenoides* as an outgroup and five single *Mec. nesaea* individuals, to test whether the signal was consistent in different individuals (*SI Appendix*, Fig. S16). We also ran ADMIXTURE in subsets of *Mechanitis* and *Melinaea* to see clustering between individuals with k ranging from 2 to 9 (116). Moreover, to investigate one putative hybrid individual, we produced a triangle plot with TriangulaR (117) with *Mec. polymnia* from east and west of the Andes.

### Androconial Chemistry.

We obtained samples of the androconial secretions from 92 males (*SI Appendix*, *Text S5*). To exclude nonandroconial compounds from our analyses, for each population the same extraction procedure was adopted with wings of conspecific females and two nonandroconial wing areas of males. Eight solvent negative controls were also taken for each sampling event. The peak areas of each chromatogram were integrated with MSD ChemStation E.02.01.1177 (Agilent Technologies, USA) to obtain the total ion current signals. A series of linear alkanes (C7–C40) was used to determine the linear retention indices (RI) of each compound (118). Compounds were identified by comparing their mass spectra and retention indices with those of reference samples available from personal and commercial mass spectral libraries [FFNSC 2, MassFinder 4, NIST17, MACE v.5.1 (119), and Wiley Registry™ 9th ed.]. The peaks exclusive to the androconial samples were used to determine the relative percentages of each compound per sample. Degradation peaks from dihydropyrrolizines were excluded from the generated ion chromatograms and statistical analysis (34). To avoid nonevident contaminants, we only considered compounds present in more than three individuals for any given taxon. The data were plotted in R (120) using the vegan package (https://doi.org/10.32614/CRAN.package.vegan) for nonmetric multidimensional scaling with “monoMDS,” specifying a global model, square root transformation, and Wisconsin double standardization (autotransform=TRUE).

### Chromosome Rearrangements.

Synteny analysis was performed using orthologous genes identified with BUSCO version 5.7.1 with the lineage database lepidoptera_odb10 and otherwise default options (121), including two outgroup genomes, *Melitaea cinxia* (122) and *D. plexippus* (123). A custom script was used to exclude Ws and retain only complete genes located in chromosome-sized scaffolds. Single copy genes were included with the exception of some genes on the neo-Z2 classified as duplicated, since the sex-linking of Z2 was so recent that most genes had high similarity to the genes on the corresponding W’s. We determined syntenic blocks, excluding single gene translocations, by comparing the position of the BUSCO-genes in each genome against the Merian elements (represented by *M. cinxia*), visualized with the R-package gggenomes (124). Minimum number of fusions was determined by the number of different Merian elements located in every query chromosome and fissions were determined as the number of query chromosomes containing parts of each Merian element for each species. The breakpoints between all species within *Mechanitis* and *Melinaea* were determined by the same principle as above using an all against all approach. Synteny analysis of the sex chromosomes and between haplotypes were performed with whole genome alignment using minimap2/2.27 with default settings and -x asm10 (1% sequence divergence) (125) and visualized after removing short alignments (<100 kb for multispecies alignment, <500 kb for haplotype alignment) using the R-package Farre-lab/syntenyPlotteR (126).

To investigate the association between chromosomal rearrangements and species divergence, we mapped the location of the breakpoints between each comparison to the reference genomes *Mec. messenoides* and *Mel. marsaeus*. Divergence and diversity was estimated in 20 kb windows along the genome (detailed above). To assess the difference from random expectation of regions of the same size and number as the breakpoint regions across the genome, we used permTest with the options “randomizeRegions” and “meanInRegions” for 50,000 permutations, as implemented in the R-package regioneR (127) in R v4.4.0 (120). Multiple correction was performed with Bonferroni adjustment (α/n), where α = 0.05 and n = 10 for the comparisons within each genus, resulting in *P*-values below 0.005 to be considered significantly different.

## Supplementary Material

Appendix 01 (PDF)

## Data Availability

All raw sequence data and genomes have been deposited in the European Nucleotide Archive (ENA) under projects PRJEB87706 (128) and PRJEB63068 (129) (accession numbers of Illumina data for all specimens are give in Table S1 and for reference genomes in Table S5). All code and phylogenies are available on GitHub (https://github.com/rapidspeciation/mechanitis_melinaea) (130).
