# Supplementary material for: Genomics of Neotropical biodiversity indicators: Two butterfly radiations with rampant chromosomal rearrangements and hybridization
Source: Proc Natl Acad Sci U S A. 2025 Jul 28;122(31):e2410939122. doi: 10.1073/pnas.2410939122 (PMC12337270; doi:10.1073/pnas.2410939122)
Supplement: Supplementary file 1 — Appendix 01 (PDF) [file pnas.2410939122.sapp.pdf]

PNAS

Supporting Information for
Genomics of Neotropical biodiversity indicators: two butterfly radiations with rampant chromosomal rearrangements and hybridisation

Eva SM van der Heijden[1,2], Karin Näsvall[1], Fernando A. Seixas[1,3], Carlos Eduardo Beserra Nobre[4], Artur Campos D Maia[4,5], Patricio Salazar-Carrión[1,2], Jonah M Walker[1,2], Daiane Szczerbowski[6], Stefan Schulz[6], Ian A Warren[2], Kimberly Gabriela Gavilanes Córdova[7], María José Sánchez-Carvajal[7], Franz Chandi[7], Alex P Arias-Cruz[7], Nicol Rueda-M[1,8], Camilo Salazar[8], Kanchon K Dasmahapatra[9], Stephen H Montgomery[10], Melanie McClure[11], Dominic E Absolon[1], Thomas C Mathers,[1] Camilla A Santos[1], Shane McCarthy[1], Jonathan MD Wood[1], Gerardo Lamas[12], Caroline Bacquet[7,13], André Victor Lucci Freitas[14], Keith R. Willmott[15], Chris D Jiggins[2], Marianne Elias[13,16], Joana I Meier[1,2]

1. Tree of Life Programme, Wellcome Sanger Institute, UK
2. Department of Zoology, University of Cambridge, UK
3. Department of Organismic and Evolutionary Biology, Harvard University, Cambridge, MA, USA
4. Graduate Program in Animal Biology, Federal University of Pernambuco, Brazil
5. Graduate Program in Plant Biology, Federal University of Pernambuco, Brazil
6. Institute of Organic Chemistry, Technische Universität Braunschweig, Germany
7. Universidad Regional Amazónica Ikiam, Ecuador
8. Biology Program, Faculty of Natural Sciences, Universidad del Rosario, Bogotá, Colombia
9. Department of Biology, University of York, UK
10. School of Biological Sciences, University of Bristol, UK
11. Laboratoire Écologie, Évolution, Interactions des Systèmes Amazoniens (LEEISA), Université de Guyane, CNRS, IFREMER, Cayenne, France
12. Museo de Historia Natural, Universidad Nacional Mayor de San Marcos, Lima, Peru
13. Smithsonian Tropical Research Institute, Gamboa, Panama
14. Laboratório de Ecologia e Sistemática de Borboletas, Instituto de Biologia, Universidade Estadual de Campinas, Brazil
15. McGuire Center for Lepidoptera and Biodiversity, Florida Museum of Natural History, University of Florida, Gainesville, FL, USA
16. Institut de Systématique Évolution Biodiversité (ISYEB), CNRS, MNHN, EPHE, Sorbonne Université, Université des Antilles, Paris, France

Corresponding author: Joana Isabel Meier
joana.meier@sanger.ac.uk

This PDF file includes:

Supporting Text S1 to S5
Supplementary Figures S1 to S23
Supplementary Tables S1 to S9
Supplementary Information References

Text S1. Taxonomic revision

For decades, taxonomists have acknowledged that species in *Melinaea* and *Mechanitis* are notoriously difficult to classify (1–3). For instance, (3) recognized only five species of *Mechanitis*, whereas (4) split them into 16 species in Colombia alone. In order to study diversification and speciation, we need to know how populations relate to each other, and what the species and subspecies are.

In *Mechanitis*, our data suggests two clades should be reclassified. The nuclear genome phylogeny of *Mechanitis* shows that the subspecies *Mec. lysimnia macrinus* (Panama and western Colombia) and *Mec. lysimnia utemaia* (Panama) form a clade that is phylogenetically distinct from the rest of *Mec. lysimnia* (Fig. 1A - box I; Fig. S1). We recognize them as a distinct species named *Mec. macrinus* **status restored** (Hewitson 1860). Furthermore, the Brazilian subspecies *Mec. lysimnia nesaea* does not cluster with the remaining South American *Mec. lysimnia* in the nuclear phylogeny (Fig. 1A - box III; Fig. S1), but is sister to *Mec. polymnia*. *Mec. lysimnia nesaea* is sympatric with *Mec. polymnia casabranca* and we therefore treat it as a distinct species, *Mechanitis nesaea* **status restored** (Hübner, 1820).

Two species show deeply divergent lineages that could potentially merit species classification, but in the absence of sequenced specimens from zones of sympatry, and given their monophyly, we currently refrain from a reclassification. There is deep divergence between Brazilian *Mec. lysimnia lysimnia* and the east Ecuadorian *Mec. lysimnia roqueensis* (Fig. 1; Fig. S1) and between the Panamanian *Mec. menapis saturata*, *Mec. menapis mantineus* from Ecuador, and a third clade with several west Colombian subspecies (*Mec. menapis occasiva* and *Mec. menapis menapis*). The overall placement of *Mechanitis menapis* in our phylogenetic tree does not match the placement of *Mechanitis menapis* in (5), where *Mech. menapis* is the most basal group. In our phylogenetic tree, it is the sister-species of *Mech. messenoides*.

Within *Mechanitis polymnia*, most individuals cluster by region (Fig. 1 and 2). There are three mitochondrial clades, which don't match the nuclear tree completely. The three *Mechanitis messenoides* subspecies (Peruvian *phasianita*, Colombian and Ecuadorian *messenoides* and *deceptus*) form a mixed monophyletic clade together; they do not cluster by country or colour pattern. This is also seen for *Mechanitis mazaeus.*

In *Melinaea*, one clade has to be reclassified. The *Mel. satevis* subspecies are not monophyletic (Fig. 1B - box V; Fig. S3). *Mel. satevis maeonis* clusters with *Mel. marsaeus*, and the other *Mel. satevis* subspecies (*Mel. s. maelus*, *Mel. s. cydon, Mel. s. lamasi*) cluster with the former *satevis* subspecies that was recently reclassified as its own species, *Mel. tarapotensis* (6). We propose to restore *Mel. satevis maeonis* as a distinct species *Melinaea maeonis* **status restored** (Hewitson 1869).

Mel. menophilus forms four clades (Fig. 1 - box IV; Fig. S3), the first consisting of the French Guianan *Mel. men. mediatrix,* the second consisting of the Colombian *Mel. men. ernestoi/menophilus* and Ecuadorian *Mel. men. zaneka*, a third with the Ecuadorian *Mel. men. menophilus* and another containing the Peruvian *Mel. men. 'ssp1' and Mel. men. hicetas.* However, since these subspecies do not occur sympatrically, we cannot assess the levels of reproductive isolation, and we retain them all as subspecies.

Text S2. Annotated synonymic list of *Mechanitis* and *Melinaea*

Genus ***Mechanitis*** Fabricius

Mechanitis Fabricius, 1807: 284. Type-species, *Papilio polymnia* Linnaeus (Scudder, 1875: 212 (7)).

Nerëis [sic] Hübner, [1806]a: [1]. Type-species, *Papilio polymnia* Linnaeus (mon.). Invalid (ICZN, Opinion 97).

Nerëis [sic] Hübner, [1806]b: pls. [1-2], [5-8], [12], [15]. Type-species, *Papilio polymnia* Linnaeus (Scudder, 1875: 227 (7)). Junior homonym of *Nereis* Linnaeus, 1758.

Hymenitis [Illiger], 1807: 1180. Type-species, *Papilio polymnia* Linnaeus (Hemming, 1934: 28 (8)). Suppressed (ICZN, Opinion 985).

Epimetes Billberg, 1820: 77. Type-species, *Papilio polymnia* Linnaeus (Hemming, 1933: 199 (9)). Junior objective synonym.

lysimnia elisa (Guérin), [1844]: 472 (*Heliconia élisa* [sic]). TL: Bolivia, [Santa Cruz, Santa Cruz de la Sierra]. D: Bolivia, NW Argentina, S Brazil. Lectotype ♂, MNHN (Fox, 1967: 123 (2)). **reinst. stat.** [raised to species by Le Crom & Winhard (2024: 28) (4) on the basis of "sus *habitus* diferentes", i.e. their different wing patterns, the molecular data confirm that SE Brazil and west Amazonian populations form a clade that we believe is best treated as a species]

elisa connectens Talbot, 1928: 412, pl. 14, fig. 8. TL: Brazil, Mato Grosso, Rio Serragem, 1500'. Holotype ♂, NHMUK.

lysimnia lysimnia (Fabricius), 1793: 161 (*Papilio*). TL: Unknown. D: SE Brazil, Paraguay, Uruguay, NE Argentina. Syntypes (♀), CCWM? Fig.- Jones, 2: pl. 8 (see Vane-Wright, 2021 (10)).

lysimene (Godart), 1819: 218 (*Heliconia*). *Nomen novum*.

aurea (Moreira), 1881: 2, pl. 1, figs. A-E (*Heliconia*). TL: "Brazil". Lectotype ♀, MNRJ (lost) (Lamas, MS).

lysimnia ab. *albescens* Haensch, 1905: 148. TL: Brazil, [Minas Gerais, Fazenda] Monte Cristo. “Lectotype” ♂, MfN (Lamas, 1994: 273 (11)). Unavailable name.

castalia (Larrañaga), 1923: 417 (*Papilio*). TL: "Uruguay". Syntypes, lost. Junior primary homonym of *Papilio castalia* Fabricius, 1793.

lysimnia menecles Hewitson, 1860: [30], pl. [15], fig. 13 (*Mechanitis m.*). TL: "Amazon". D: E Colombia, E Ecuador, E Perú, N Bolivia, W Brazil. Syntypes (♀), NHMUK. **reinst. stat.** [treated as subspecies of *M. elisa* by Le Crom & Winhard (2024) (4)]

elisa acreana d'Almeida, 1950: 394, figs. 2-3. TL: Brazil, Acre, Xapuri. Holotype ♂, DZUP.

elisa roqueensis Bryk, 1953: 30. TL: Perú, [San Martín], Roque]. Holotype ♂, RMS. **n. syn.** [treated as a valid subspecies in Lamas (2004) (12)]

lysimnia ocona H. Druce, 1876: 208 (*Mechanitis o.*). TL: Perú, [Cuzco], Valle del Río Santa Ana, Huiro. D: E Perú. Holotype ♂, NHMUK. **reinst. stat.** [treated as subspecies of *M. elisa* by Le Crom & Winhard (2024) (4)]

vilcanota Röber, 1904: 105. TL: Perú, Cuzco. Lectotype ♀, SMTD (Lamas, 1992: 176 (13)).

lysimnia tapajona Freitas & Mota, *in* Mota, Rosa, Vasconcellos, Willmott & Freitas, 2022: 48, figs. 1-3. TL: Brazil, Pará, Novo Progresso, Alvorada da Amazônia, Côrrego rio Quico/Arco-íris, -7.281835°, -55.311290°. D: Brazil. Holotype ♂, ZUEC. [relationships with respect to *M. nesaea* **rev. stat.** and *M. l. lysimnia* need clarifying]

macrinus bipuncta Forbes, 1948: 19, fig. 7 (*Mechanitis mazaeus b.*). TL: Venezuela, [Bolívar], upper Caroní River, Surukum Basin. D: S Venezuela, Guyana, N Brazil. Holotype ♀, CUIC. **rev. stat.** [treated as a subspecies of *M. lysimnia* in Lamas (2004) (12)]

macrinus labotas Distant, 1876: xii (*Mechanitis l.*). TL: "Costa Rica". D: Pacific Costa Rica, Pacific Panamá. Syntypes (♀), NHMUK. **rev. stat.** [treated as a subspecies of *M. lysimnia* in Lamas (2004) (12)]

macrinus limnaea Forbes, 1930: 317 (*Mechanitis l.*). TL: French Guiana, [Guyane], St. Laurent. D: Guyana, Suriname, French Guiana, N Brazil. Holotype ♂, CUIC. **rev. stat.** [treated as a subspecies of *M. lysimnia* in Lamas (2004) (12)]

mantineus var. Forbes, 1924: 146, 156.

mantineus f. *forbesi* Bryk, 1937: 491, 641. TL: French Guiana, [Guyane, St. Jean]. Holotype ♀, USNM.

macrinus macrinus Hewitson, 1860: [29], pl. [15], fig. 11. TL: "New Granada". D: E Panamá, Colombia, W Ecuador, NW Perú. Syntypes (♂), NHMUK. [treated as a species by Le Crom & Winhard (2024) (12)]

numerianus C. Felder & R. Felder, 1865: 368, pl. 45, fig. 9. TL: Colombia, "Bogotá" (error). Syntypes (♀), NHMUK.

macrinus blissi Fox, 1942: 26, figs. 7-8. TL: Panamá, [Canal Zone], Fort Kobbe. Holotype ♂, RPM. [*m. macrinus* x *m. utemaia*].

macrinus pacificus Le Crom & Winhard, 2024: 5, 29, pl. 29. TL: [Colombia], Antioquia, Turbo, Río León, Urabá. 50m. Holotype ♂, IAVH. **n. syn.** [described by Le Crom & Winhard (2024) (4) based on supposedly reduced yellow DFW postdiscal markings in comparison with typical *M. macrinus macrinus*, but the ST of *macrinus* (Warren et al., 2024) (14) shows even more reduced markings than the HT of *pacificus*]

aurantiacus Winhard & Le Crom, *in* Le Crom & Winhard, 2024: 5, 29, pl. 30. TL: [Colombia], Valle [del Cauca], El Salto, 9km W Cisneros, 300m. Holotype ♂, ZSM. **n. syn.** [described by Le Crom & Winhard (2024) (4) as a species in comparison with *M. macrinus macrinus* based on complete orange DHW submarginal band and black at the base of DFW cell Cu2-Cu1; the extent of DHW orange is variable throughout the range of *M. m. macrinus*, and a black spot at the base of DFW cell Cu2-Cu1 occurs occasionally in specimens in Panama and western Ecuador; in the absence of other morphological or genetic evidence, we consider the type specimen to represent a form of *M. m. macrinus*]

aurantiacus magdalenae Le Crom & Winhard, 2024: 5, 30, pl. 30. TL: [Colombia], Huila, Garzón, Vereda El Espinal, Reserva Privada Taky-Huaylla, 1000m. Holotype ♂, IAVH. **n. syn.** [described by Le Crom & Winhard (2024) (4) as a subspecies in comparison with '*M. aurantiacus*', the wing pattern is typical of *M. macrinus macrinus* specimens with more extensive DHW orange]

macrinus solaria Forbes, 1948: 15, fig. 5 (*Mechanitis polymnia s*.). TL: Venezuela, Sucre, El Chorro. D: NE Venezuela, Trinidad. Holotype ♂, CUIC. **rev. stat.** [treated as a subspecies of *M. lysimnia* in Lamas (2004) (12)]

macrinus utemaia Reakirt, 1866: 241 (*Mechanitis u.*). TL: "Honduras". D: S México to W Panamá, N Colombia, N Venezuela. Holotype ♀, FMNH. **rev. stat.** [treated as a species distinct from *M. lysimnia* by Le Crom & Winhard (2024) (4) on the basis of the "*habitus* diferentes" (different appearance), the molecular data in the present study suggest this taxon is best treated as a subspecies of *M. macrinus*]

doryssus f. *utemaia* ab. *extrema* Hoffmann, 1940: 636, fig. 1. TL: México, Chiapas, Mapastepec. “Holotype” ♂, AMNH. Unavailable name.

utemaia guajira Le Crom & Winhard, 2024: 6, 33, pl. 34. TL: [Colombia], Guajira, Río Palomino, 50m. Holotype ♂, ZSM. **n. syn.** [described by Le Crom & Winhard (2024) (4) based on supposedly more reddish DFW yellow markings and broader DFW yellowish subapical marking, in comparison with *M. m. utemaia*; these supposed characters lie within the typical range of variation of *M. m. utemaia*]

mazaeus beebei Forbes, 1948: 18, fig. 6. TL: Venezuela, Monagas, Caripito. D: E Venezuela, Guyana. Holotype ♂, CUIC.

mazaeus lanei Fox, 1967: 83, figs. 63, 69B, 71, 80A-B (*Mechanitis l*.). TL: Brazil, Pará, Igarapé Açú. D: Brazil (E Pará). Holotype ♂, NHMUK.

mazaeus mazaeus Hewitson, 1860: [28], pl. [14], fig. 8. TL: "Amazon". D: E Colombia, E Ecuador, E Perú, W Brazil. Lectotype ♀, NHMUK (Lamas, 1994: 284 (11)).

fallax Butler, 1873: 154. TL: Colombia, "Bogotá" (error). Lectotype ♀, NHMUK (Lamas, 1999: 133 (15)). **rev. stat.** [treated as a species by Le Crom & Winhard (2024) (4) on basis of its sympatry with *M. mazaeus*, but without discussion of diagnostic characters, the LT of *fallax* (see Warren et al. 2024 (14)) is a common phenotype found throughout the western Amazon]

polymnia var. *plagifera* Staudinger, 1885: 62. TL: Perú, [Loreto], Yurimaguas. Lectotype ♂, MfN (Lamas, MS).

mazaeus ab. *lucifera* Haensch, 1905: 146, pl. 4, fig. 1. TL: Perú, [Loreto], Yurimaguas. “Lectotype” ♀, MfN (Lamas, 1994: 284 (11)). Unavailable name.

mazaeus ab. *phasianita* Haensch, 1905: 146. TL: [Perú, Loreto], Yurimaguas. “Lectotype” ♂, MfN (Lamas, 1994: 290 (11)). Unavailable name.

mazaeus ab. *nigroapicalis* Haensch, 1905: 146. TL: Brazil, [Amazonas], São Paulo de Olivença. “Lectotype” ♂, MfN (Lamas, 1994: 287 (11)). Unavailable name.

visenda elevata Riley, 1919: 182. TL: Brazil, [Amazonas], Rio Purús, Aliança, Canutama; Tefé; Fonteboa. Syntypes (♂♀), NHMUK. **rev. stat.** [treated as a subspecies of '*M. fallax*' by Le Crom & Winhard (2024) (4), the ST of *elevata* (see Warren et al. 2024 (14)) is a common phenotype found throughout the western Amazon]

lorigae Fernández, v.1928: 209, fig. 6. TL: Perú, [Loreto], Iquitos. Lectotype ♀. MNCN (Lamas, 1999: 133, fig. 6 (15)).

egaensis septentrionalis Apolinar, xi.1928: 180. TL: Colombia, Boyacá, Garagoa, Valle de Tensa. Holotype, lost (Lamas, 1979: 67 (16)).

mazaeus williamsi Fox, 1941: 6, pl. 2, fig. 16. TL: Perú, [San Martín], Achinamiza. Holotype ♂, RPM.

fallax pothetoides d'Almeida, 1951: 13, pl. 5, fig. 2. TL: Brazil, [Rondônia], Rio Verde. Holotype ♀, MNRJ (lost). **n. syn.** [Lamas (2004) (12) treated *pothetoides* as a valid subspecies, but the wing pattern of the HT occurs commonly throughout the western Amazon, so we here treat it as a synonym of *M. mazaeus mazaeus*]

foxi d'Almeida, 1951: 14, pl. 1, figs. 2-3. TL: Brazil, Acre, alto Rio Juruá. Holotype ♂, MNRJ (lost).

messenoides dissimilis Le Crom & Winhard, 2024: 6, 35, pl. 38. TL: [Colombia], Vaupés, Taraira, Estación Caparú. Holotype ♂, IAVH. **n. syn.** [the protruding edge of the DFW yellow postdiscal band in cell M2-M1, relatively broad forewings, and white VHW marginal spots, suggest that this is a form of *M. mazaeus mazaeus* (see Hill et al. 2012 (17))]

deceptus dicolor Le Crom & Winhard, 2024: 6, 36, pl. 39. TL: [Colombia], Guaviare, San José del Guaviare. Holotype ♂, IAVH. **n. syn.** [the protruding edge of the DFW orange postdiscal band in cell M2-M1, relatively broad forewings, and white VHW marginal spots, suggest that this is a form of *M. mazaeus mazaeus* (see Hill et al. 2012 (17))]

mazaeus metaensis Le Crom & Winhard, 2024: 6, 36, pl. 39. TL: [Colombia, Meta], Monterredondo, 1600m [error ?]. Holotype ♂, ICNB. n**. syn.** [described by Le Crom & Winhard (2024) from two specimens, based on supposedly less extensive black markings on the DFW in comparison with typical *M. m. mazaeus*; these differences are well within the range of wing pattern variation observed in *M. mazaeus* (see Hill et al. 2012 (17))]

mazaeus neukircheni Neild, 2008: 109, 234, figs. (*Mechanitis lysimnia n.*) TL: Venezuela, Táchira, Parque Nacional El Tamá, Río Frío, 600m. D: NE Colombia, SW Venezuela. Holotype ♂, MIZA. **rev. stat.** [Neild (2008) (18) described this taxon as a subspecies of *M. lysimnia*, due to its similarity to *M. macrinus utemaia* (then a subspecies of *M. lysimnia*), but discussed the possibility that it might represent *M. mazaeus*, whereas Le Crom & Winhard (2024) (4) treated it as subspecies of '*M. dorissides*' (=*M. polymnia*); our molecular data support Neild's (2008) (18) supposition of a close relationship between *M. macrinus utemaia* and *M. mazaeus mazaeus*, and we here treat *neukircheni* as a subspecies of *M. mazaeus* because of its geographic proximity to *M. mazaeus mazaeus*]

mazaeus pannifera Butler, 1877: 150 (*Mechanitis p.*) TL: Brazil, [Amazonas], Ega (= Tefé); [Pará], Óbidos. D: ?E Colombia, SE Venezuela, Guyana, Suriname, French Guiana, N Brazil. Syntypes (♂), NHMUK. **reinst. stat.** [treated as a species by Le Crom & Winhard (2024) (4) on basis of its yellow rather than orange DFW subapical band, a highly variable character (in color, size, presence or absence) as supported by lack of correlation of DNA sequences with these forms; *pannifera* represents a relatively stable phenotype in the northern Amazon and Guianas, albeit one that also occurs infrequently in the western Amazon, and we retain it as a distinct subspecies for the present]

mazaeus visenda Butler, 1877: 150 (*Mechanitis v.*). TL: Brazil, [Pará, Rio] Tapajós, [Trovador]. D: Brazil (Pará). Syntypes (♂), NHMUK.

menapis caribensis Fox, 1967: 114, figs. 69H, 104, 106. TL: Venezuela, Distrito Federal, Puerto La Cruz. D: N Colombia, N Venezuela, ?Curaçao, Trinidad. Holotype ♂, CMNH.

menapis dariensis Brown, 1977: 187, fig. 80. TL: Panamá, Darién, near El Real, base of Cerro Pirre, near sea level. D: E Panamá, NW Colombia. Holotype ♂, AMNH.

macrinus extremus Le Crom, *in* Le Crom & Winhard, 2024: 5, 29, pl. 30. TL: [Colombia], Antioquia, San Jerónimo, 700m. Holotype ♂, IAVH. **n. syn.**

doryssus subobscurus Winhard & Le Crom, *in* Le Crom & Winhard, 2024: 5, 30, pl. 31. TL: [Colombia, Risaralda], 22km W Pueblo Rico, 700m. Holotype ♂, ZSM. **n. syn.**

menapis doryssus Bates, 1864: 33 (*Mechanitis d.*). TL: Guatemala, [Alta Verapaz, San Gerónimo]. D: S México to W Panamá. Lectotype ♂, NHMUK (Lamas, 1988: 49, fig. 1 (19)).

doryssus f. *saturata* ab. *escalantei* Hoffmann, 1940: 636, fig. 2. TL: México, Guerrero, Acahuizotla. "Holotype" ♀, AMNH. Unavailable name.

saturata Godman, *in* Godman & Salvin, 1901: 642 (*Mechanitis s.*). TL: Costa Rica, [Cartago], Caché. Lectotype ♀, NHMUK (Lamas, 1988: 49, fig. 2 (19)). **n. syn.** [Lamas (1988) (19) clarified the taxonomic identities of *doryssus* and *saturata*, and treated them as valid subspecies of *M. menapis*, a classification followed by Lamas (2004) (12). Earlier authors, e.g. Fox (1967) (2), regarded all individuals from Mexico to Panamá as representing the same taxon, although treating *doryssus* as a subspecies of *M. polymnia*; we see insufficient geographic variation to retain the name]

menapis mantineus Hewitson, 1869: 12 (*Mechanitis mantineus*). TL: Ecuador, [Cañar, Río] Angas. D: SW Colombia, W Ecuador, ?NW Perú. Syntypes (♂), NHMUK. **reinst. stat.** [treated as species by Le Crom & Winhard (2024) (4) based on its distinctive wing pattern, our molecular data show that this taxon groups with other *M. menapis* taxa, albeit with some distance between the clades, and we tentatively treat it as a subspecies, for the present]

menapis menapis Hewitson, [1856]: [17], pl. [9], fig. 1. TL: "New Granada". D: Colombia. Syntypes (♀), NHMUK.

franis Reakirt, 1868: 90. TL: Colombia, [Cundinamarca], "Insagasugá" (= Fusagasugá). Syntypes (♀), FMNH. **reinst. syn.** [treated as species by Le Crom & Winhard (2024) (4) based on a narrower black DHW postdiscal band and orange DFW tornal marking, both of these phenotypes occur close together in the same clade in our data (vouchers Sal_5683 and Sal_4723, for example), with variation in both characters among other specimens; we therefore restore the name as a synonym of *M. menapis menapis*]

menapis var. *peruana* Hopffer, 1879: 419. TL: ["Bolivia"] (error). Lectotype ♂, MfN (Lamas, MS).

menapis anchicayaensis Le Crom & Winhard, 2024: 5, 31, pl. 33. TL: [Colombia, Valle del Cauca], Anchicayá, 400-500m. Holotype ♂, MPUJ. **n. syn.** [the holotype is within the range of typical variation of *M. menapis menapis*, and we conclude that all four type specimens, bearing the same label data in the collection of the Universidad Javeriana in Bogotá, Colombia (Le Crom & Winhard, 2024), are mislabeled]

menapis nariniensis Le Crom & Winhard, 2024: 5, 30, pl. 31. (*M. doryssus nariniensis*). TL: [Colombia], Nariño, Ricaurte, La Planada, 1600m. D: SW Colombia, ?NW Ecuador. Holotype ♂, IAVH. **rev. stat.** [described as a subspecies of '*M. doryssus*' [*M. menapis doryssus*] by Le Crom & Winhard (2024) (4), this phenotype resembles specimens known from northwestern Ecuador which DNA barcodes indicate to belong to *M. menapis*, and we thus place it as a subspecies of that species]

menapis occasiva Fox, 1967: 117, figs. 104, 108. TL: Colombia, Cauca, Munchique, [2000m]. D: W Colombia (Cauca). Holotype ♂, AMNH. **reinst. stat.** [treated as a subspecies of *Mechanitis franis* by Le Crom & Winhard (2024) (4), see discussion under *franis*]

aurantiacus tenebrosus Winhard, *in* Le Crom & Winhard, 2024: 5, 29, pl. 30. TL: [Colombia], Valle [del Cauca], Quebrada El Pital, 10km N La Paila, 800m. Holotype ♂, ZSM. **n. syn.** [described from a single specimen from Valle del Cauca (Le Crom & Winhard, 2024) (4), the status of this name is speculative; the reduced yellow DFW postdiscal band is consistent with *M. macrinus macrinus* (although some specimens of that taxon do show a broken band, albeit not as reduced as in the HT of *tenebrosus*), while the reduced DHW orange band is consistent with *M. menapis occasiva*. Without DNA sequence data the issue is unlikely to be resolved, but given the large wing pattern variation in all *Mechanitis* species from western Colombia, and the fact that this subspecies would otherwise be based on a single specimen, we believe there is insufficient evidence to regard it as valid, and we treat it for the present as a synonym of *M. menapis occasiva*]

franis salazari Le Crom, *in* Le Crom & Winhard, 2024: 5, 31, pl. 32. TL: [Colombia], Caldas, Anserma, 1000m. Holotype ♂, IAVH. **n. syn.** [described by Le Crom in Le Crom & Winhard (2024) (4) based on the thin yellow line joining the postdiscal and discal markings on the DFW, and more extensive black markings, in comparison with *M. m. occasiva*; both are variable characters within series of specimens from the same locality, e.g. Valle del Cauca, Cali, Cañas Gordas (specimens in MGCL), and we therefore treat this name as a synonym of *M. m. occasiva*]

franis morales Winhard, *in* Le Crom & Winhard, 2024: 5, 31, pl. 32. TL: [Colombia], Cauca, Vereda Los Cafés, cerca de Morales, 1550m. Holotype ♂, ZSM. **n. syn.** [described by Winhard in Le Crom & Winhard (2024) (4) based on the narrower black bar at the end of the FW discal cell and supposedly broader orange DHW postdiscal band; both are variable characters within series of specimens from the same locality, e.g. Valle del Cauca, Cali, Cañas Gordas (specimens in MGCL), and we therefore treat this name as a synonym of *M. m. occasiva*]

menapis flavus Winhard & Le Crom, *in* Le Crom & Winhard, 2024: 5, 32, pl. 33. TL: [Colombia], Valle [del Cauca], Calcedonia, 1150m. Holotype ♂, IAVH. **n. syn.** [described by Le Crom & Winhard (2024) (4) based on the more extensive yellow markings in the middle of the DFW, which is variation seen within series of specimens from the same locality, e.g. Valle del Cauca, Cali, Cañas Gordas (specimens in MGCL), and we therefore treat this name as a synonym of *M. m. occasiva*]

menapis submonte Le Crom & Winhard, 2024: 5, 32, pl. 33. TL: [Colombia], Meta, Sierra La Macarena, Río Guayabero. Holotype ♂, ICNB. **n. syn.** [the HT is indistinguishable from specimens of *M. menapis* from the Cauca valley (Valle del Cauca), while the figured female (Le Crom & Winhard, 2024, plate 33 (4)), is typical of specimens from the Magdalena valley (*M. menapis menapis*). The putative type locality (Meta, Sierra La Macarena, Río Guayabero) is in the Amazon basin, but it, and all of the type specimens with dates, were collected prior to 1960, and the absence of modern records from the Amazon (including in online resources such as iNaturalist), and the fact that there are no clear characters separating this putative taxon from others, suggests the possibility of mislabeling]

messenoides C. Felder & R. Felder, 1865: 356. TL: "New Granada". D: E Colombia, E Ecuador, E Perú, Bolivia. Holotype ♀, NHMUK. *Nomen novum* for *Mechanitis menophilus* Hewitson, [1856] (in part).

menophilus Hewitson, [1856]: [17] (in part), pl. [9], fig. 2 (*nec* 3). Misidentification.

deceptus Butler, 1873: 154. TL: Ecuador, [Azuay], Cuenca. Syntypes (♂), NHMUK. **n. syn.** [treated as a species by Le Crom & Winhard (2024) (4) due to the orange rather than yellow distal half of the FW median band, Hill et al. (2012) (17) showed that these phenotypes are not distinguishable genetically; although an entirely orange form is somewhat more common in Ecuador than it is in Colombia, both forms are frequent throughout the distribution of this species, so we treat *deceptus* as a synonym of *messenoides*]

mazaeus simplex Bryk, 1953: 29. TL: Perú, [San Martín], Roque. Holotype ♀, RMS.

mazaeus holmgreni Bryk, 1953: 29. TL: Perú, [Puno], Chaquimayo. Holotype ♂, RMS. **n. syn.** [the HT of *ballucatus* has the distal half of the FW median band tinged yellow, but as discussed under *deceptus*, despite likely geographic variation in the frequency of yellow vs. orange forms, we do not think that they are sufficiently geographically stable to merit recognition as distinct subspecies, so we treat *holmgreni* as a synonym of *messenoides*]

messenoides ballucatus Fox, 1967: 110, figs. 98, 102. TL: Bolivia, [Santa Cruz], Río Yapacani. Holotype ♂, CMNH.

messenoides calidus Le Crom & Winhard, 2024: 6, 35, pl. 38. TL: [Colombia], Guaviare, San José del Guaviare. Holotype ♂, IAVH. **n. syn.** [described by Le Crom & Winhard (2024) (4) based on the presence of an orange discal band and apex on the DHW, which is an infrequent variation seen within populations of typical *M. messenoides* (e.g., Hill et al. 2012, Fig. 1B (17))]

pannifera australis Le Crom, *in* Le Crom & Winhard, 2024: 6, 36, pl. 40. TL: [Colombia], Putumayo, Puerto Leguízamo, 400m. Holotype ♀, IAVH. **n. syn.** [the HT female has the FW band without a strong distal projection in cell M2-M1, and no white VHW marginal spots, suggesting it is a form of *M. messenoides* (e.g., Hill et al. 2012, Fig. 1B (17))]

nesaea Hübner, [1820]: pl. [2], figs. 1-4. TL: Unknown. D: NE Brazil. Syntypes (♂♀), lost? **rev. stat.** [as discussed in the text, our molecular data support recognition of this taxon as a distinct species]

lysimnia ab. *sulphurescens* Haensch, 1905: 148. TL: Brazil, Bahia. "Lectotype" ♀, MfN (Lamas, 1994: 293 (11)). Unavailable name.

polymnia angustifascia Talbot, 1928: 411, pl. 14, fig. 7; pl. 16, figs. 4, 7. TL: Brazil, Mato Grosso, Rio Serragem, 1500'. D: SE Perú, Bolivia, S Brazil. Holotype ♂, NHMUK.

travassosi d'Almeida, 1951: 11, pl. 2, figs. 1-2. TL: Brazil, Acre, [alto Rio Juruá]. Holotype ♂, DZUP.

polymnia apicenotata Zikán, 1941: 15 (*Mechanitis p.* r. *a.*). TL: Brazil, [Amazonas], Rio Negro, São Gabriel. D: E Colombia, S Venezuela, NW Brazil. Lectotype ♂, IOC (d'Almeida, 1956: 3, fig. 2 (20)).

polymnia sancti-gabrielis [sic] Bryk, 1953: 29. TL: Brazil, [Amazonas], São Gabriel. Lectotype ♂, RMS (Lamas, MS).

polymnia bolivarensis Fox, 1967: 77, figs. 71, 74 (*Mechanitis isthmia b.*). TL: Venezuela, Bolívar, Río Suapure. D: E Venezuela, Guyana. Holotype ♂, AMNH.

polymnia casabranca Haensch, 1905: 145, fig. 3 (*Mechanitis c.*). TL: Brazil, [Minas Gerais, Fazenda] Monte Cristo. D: E Brazil, NE Argentina. Lectotype ♂, MfN (Lamas, 1994: 278 (11)).

polymnia caucaensis Haensch, 1909: 124 (*Mechanitis p.* f. *caucaënsis* [sic]). TL: Colombia, Valle del Cauca. D: E Panama (?), W Colombia. Lectotype ♂, MfN (Lamas, 1994: 278 (11)). **reinst. stat.** [treated as subspecies of *Mechanitis chimborazona* by Le Crom & Winhard (2024) (4)]

isthmia lapaila Winhard & Le Crom, *in* Le Crom & Winhard, 2024: 6, 34, pl. 37. TL: [Colombia], Valle del Cauca, Quebrada Pital, 10km N La Paila, 800m. Holotype ♂, ZSM. **n. syn.** [described by Le Crom & Winhard (2024) (4) from four specimens from Valle del Cauca, in a transition zone between *M. p. caucaensis* and *M. p. veritabilis* (treated as distinct species by Le Crom & Winhard (2024) (4)), the status of this name is not clear, but we feel there is insufficient evidence to regard it as representing a distinct phenotype inhabiting a clearly defined geographic region]

utemaia luteola Winhard & Le Crom, *in* Le Crom & Winhard, 2024: 6, 32, pl. 34. TL: [Colombia], Valle del Cauca, Quebrada Las Cañas, 10km N La Paila, 900m. Holotype ♂, ZSM. **n. syn.** [described by Le Crom & Winhard (2024) (4) based on yellowish DHW discal band, broader black postdiscal band and margin, and more yellowish markings in cell Cu2-Cu1, in comparison with *M. macrinus utemaia*; the HT is from the same locality as *M. isthmia lapaila*, and it seems most parsimonious to assume they represent the same species; without DNA sequence data and better information about nearby populations and geographic variation, the status of this name will remain unclear]

polymnia chimborazona Bates, 1864: 33, footnote (*Mechanitis c.*). TL: Ecuador, [Chimborazo], western foot of Chimborazo, 2-4000'. D: SW Colombia, W Ecuador, NW Perú. Syntypes (♂♀), NHMUK. **reinst. stat.** [treated as species by Le Crom & Winhard (2024) (4), the molecular data are consistent with this and other Transandean taxa being treated as subspecies of *M. polymnia*]

werneri Hering, *in* Hering & Hopp, 1925: 188. TL: Colombia, [Cauca], Río [San Juan de] Micay, El Correo. Holotype ♀, MfN. **n. syn.** [Le Crom & Winhard (2024) (4) applied the name *werneri* to a population of *M. polymnia* from the western slopes of the northern Cordillera Occidental in Colombia, which differs from *M. p. chimborazona* in having a complete black discal band on the VHW in the female; this may well represent a distinct subspecies, but the HT of *werneri* has a reduced black VHW postdiscal band typical of *M. p. chimborazona*, and we thus synonymize it with that subspecies]

polymnia dorissides Staudinger, [1884]: pl. 28, fig. [8] (*Mechanitis d.*); 1885: 62 (*Mechanitis doryssides* [sic]). TL: Perú, [Loreto], Yurimaguas. D: E Colombia, E Ecuador, NE Perú. Lectotype ♂, MfN (Fox, 1967: 81). **reinst. stat.** [raised to species by Le Crom & Winhard (2024) (4), the molecular data show that this taxon forms a clade with Guianan *M. polymnia* and we thus treat it as a subspecies]

aurantiacus orientalis Le Crom, *in* Le Crom & Winhard, 2024: 5, 30, pl. 31. TL: [Colombia], Caquetá, San Vicente del Caguán, Vereda Las Vegas, Pato Bajo, Finca Napuraña. Holotype ♂, ICNB. **n. syn.** [the DFW of the HT has the yellow postdiscal band broken, reduced orange in cell Cu2-Cu1, and an extensive yellow-orange subapical band, but these are all characters seen within west Amazonian populations of *M. polymnia*, from which the HT otherwise does not differ]

dorissides vaupesensis Le Crom & Winhard, 2024: 6, 33, pl. 35. TL: [Colombia], Vaupés, Río Apaporis, Comunidad de Jirijirimo. Holotype ♀, ICBN. **n. syn.** [described by Le Crom & Winhard (2024) (4) based on a supposedly broader yellow DFW postdiscal band, the HT female is a typical specimen of *M. p. dorissides*, whereas the only other type specimen, the male figured under this name on p. 33 and pl. 35 is *M. mazaeus mazaeus*].

isthmia remolinensis Le Crom & Winhard, 2024: 6, 35, pl. 37. TL: [Colombia], Meta, Remolinos. Holotype ♂, IAVH. **n. syn.** [without explanation, Le Crom & Winhard (2024) (4) described this name as a subspecies of *Mechanitis isthmia*, noting that the yellow DFW postdiscal band was broken in the middle (as in the HT of *aurantiaca*

orientalis Le Crom, from the same region); this is an uncommon variant within typical *M. p. dorissides* that, based on images in iNaturalist, is not stable even within the putative range of *remolinensis*]

isthmia tacana Le Crom & Winhard, 2024: 6, 35, pl. 38. TL: [Colombia], Amazonas, Tacana. Holotype ♂, UCaldas. **n. syn.** [Le Crom & Winhard (2024) (4) described this putative subspecies as differing from *isthmia remolinensis* in having reduced black discal markings on the DFW, a variable character throughout the distribution of *M. p. dorissides*, from which the HT otherwise does not differ]

polymnia egaensis Bates, 1862: 531, pl. 56, fig. 7a (*Mechanitis p*. var. *e*.). TL: Brazil, [Amazonas], Ega (= Tefé). D: W Brazil. Lectotype ♀, NHMUK (Fox, 1967: 106 (2)).

obscura Butler, 1877: 149. TL: Brazil, [Amazonas], Ega (= Tefé); Rio Juruá, Pupunha. Syntypes (♂♀), NHMUK. *Nomen novum* for *Mechanitis egaensis* var. 1 Bates, 1862.

egaensis contracta Riley, 1919: 182. TL: Brazil, [Amazonas], Rio Purús, Aliança, Canutama. Syntypes (♂♀), NHMUK.

egaensis obumbrata d'Almeida, 1951: 10, pl. 1, fig. 1. TL: Brazil, Acre, alto Rio Juruá. Holotype ♀, MNRJ (lost).

polymnia eurydice Haensch, 1905: 147, fig. 4 (*Mechanitis e*.). TL: Perú, [Cuzco], Illapani. D: E Perú. Lectotype ♂, MfN (Lamas, 1994: 280 (11)). **reinst. stat.** [treated as a subspecies of *Mechanitis dorissides* by Le Crom & Winhard (2024) (4)]

lycidice f. *argentea* Prüffer, 1922: 5, pl. 2, fig. 3. TL: Perú, [Ayacucho], Monte Rico. Syntypes (♂), lost.

polymnia isthmia Bates, 1863: 247, pl. 29, fig. 1 (*Mechanitis i*.). TL: Panamá, [Panamá], Lion Hill. D: Honduras, E Nicaragua, Costa Rica, W Panamá. Syntypes (♂♀), NHMUK. **reinst. stat.** [treated as a species by Le Crom & Winhard (2024) (4), the molecular data show that this taxon forms a clade with other *M. polymnia*, including the nominate subspecies, and we believe it is best treated as a subspecies]

isthmica Bates, 1863: pl. 29, fig. 1. Incorrect original spelling.

californica Reakirt, [1866]: 223. TL: "USA, California, Los Angeles" (error). Syntypes (2♂), FMNH.

ovata Distant, 1876: xi. TL: "Costa Rica". Syntypes (♀), NHMUK.

polymnia kayei Fox, 1967: 78, figs. 71, 75 (*Mechanitis isthmia k*.). TL: Trinidad, Mt. Michael. D: Trinidad. Holotype ♂, CMNH.

polymnia lycidice Bates, 1864: 33 (*Mechanitis l*.). TL: Guatemala, [Baja Verapaz], Chuacús. D: S México, Guatemala, Belize, El Salvador, W Honduras, W Nicaragua. Lectotype ♂, NHMUK (Lamas, 1994: 275 (11)).

doryssa Boisduval, 1870: 31. TL: "Honduras"; "México". Syntypes (♂♀), NHMUK. Junior primary homonym of *Mechanitis doryssus* Bates, 1864.

lycidice f. *arcana* Haensch, 1909: 126. TL: Honduras, [Cortés, San Pedro Sula]. Lectotype ♀, MfN (Lamas, 1994: 275 (11)).

polymnia mauensis Forbes, 1948: 15, fig. 4. TL: Brazil, [Pará], Santarém. D: Brazil (Tapajós). Holotype ♂, CUIC.

polymnia polymnia (Linnaeus), 1758: 466 (*Papilio*). TL: "Surinam". D: Guyana, Suriname, French Guiana, N Brazil. Lectotype ♀, UUZM (Honey & Scoble, 2001: 370 (21)).

mopsus (Linnaeus), 1758: 487 (*Papilio*). TL: "Indiis". Syntypes, lost?

plagigera Butler, 1877: 150. TL: Brazil, [Pará], Prainha. Syntypes (♂), NHMUK.

polymnia atrata Brévignon, 2007: 539, pl. 1, figs. 1-4. TL: French Guiana, Saint-Laurent-du-Maroni. Holotype ♀, coll. Brévignon. **n. syn.** [the extent of black on the HW is locally variable in this and many co-mimetic taxa from the Guianan region]

polymnia proceriformis Bryk, 1953: 30 (*Mechanitis doryssus p*.). TL: Perú, [San Martín], Roque. D: E Perú (Huallaga). Holotype ♂, RMS.

polymnia veritabilis Butler, 1873: 155 (*Mechanitis v*.). TL: Colombia, "Bogotá" (error); "Venezuela". D: E Panamá, N & W Colombia, N Venezuela. Syntypes (♂♀), NHMUK. **reinst. stat.** [treated as a subspecies of *Mechanitis isthmia* by Le Crom & Winhard (2024) (4)]

utemaia fluminea Le Crom & Winhard, 2024: 6, 33, pl. 34. TL: [Colombia], Santander, Socorro, Potrero. Holotype ♂, ICNB. **n. syn.** [described by Le Crom & Winhard (2024) (4) in comparison with *Mechanitis 'lysimnia' utemaia*, the HT is a typical specimen of *M. p. veritabilis*]

isthmia occidentalis Winhard & Le Crom, *in* Le Crom & Winhard, 2024: 6, 34, pl. 36. TL: [Colombia], Valle del Cauca, Río Dagua, El Salto, W of Cisneros, 300m. Holotype ♂, ZSM. **n. syn.** [described by Le Crom & Winhard (2024)

(4) from two specimens from Valle del Cauca and Risaralda, they noted the more yellowish discal cell patch in comparison with typical *M. p. veritabilis*; given the few specimens examined and lack of information about neighboring populations, this might represent a distinct subspecies on the western slopes of the Cordillera Occidental, or transitional forms between *M. p. veritabilis* and *M. p. isthmia*; for the moment, we feel there is insufficient evidence to regard this as a distinct phenotype inhabiting a clearly defined geographic region]

Nomina nuda

anah Doubleday, 1847: 130 ("Guyana")
balea Doubleday, 1847: 130 ("Honduras")
baucis Doubleday, [1845]: 148 ("Venezuela")
polymnia var. *cruda* Fassl, 1914: 43
didyma Doubleday, 1847: 130 ("Brazil")
polymnia var. *donna* Fassl, 1914: 43
doryssa Doubleday, [1845]: 55 ("Colombia")
edocla Doubleday, 1847: 130
francesca Doubleday, 1847: 130 ("Brazil")
hispulla Doubleday, 1847: 130 ("Brazil")
lysidice Doubleday, [1845]: 55 ("Colombia")
macrinellus Herrich-Schäffer, 1865: 175
neria Herrich-Schäffer, 1865: 175
rythiria Herrich-Schäffer, 1865: 175
zarina Doubleday, 1847: 130 ("Bolivia")
zillah Doubleday, 1847: 130 ("Honduras")

Genus ***Melinaea*** Hübner

Melinaea Hübner, 1816: 11. Type-species, *Papilio egina* Cramer (Scudder, 1875 (7)).
Melinaea Bates, 1862: 549. Type-species, *Papilio egina* Cramer (Hemming, 1964: 118 (22)). Junior homonym and objective synonym.
Czakia Kremky, 1925: 192. Type-species, *Melinaea mneme* var. *mediatrix* Weymer (orig. des.).

ethra (Godart), 1819: 221 (*Heliconia*). TL: "Brazil". D: E Brazil. Syntypes (♀), MNHN.
phasis Herrich-Schäffer, 1865: 175. *Nomen nudum*.
phasis C. Felder & R. Felder, 1865: 354. TL: Brazil, Bahia. Lectotype ♂, NHMUK (Lamas, MS).

idae idae (C. Felder & R. Felder), 1862: 414 (in part) (*Mechanitis*). TL: Colombia, "Bogotá, 7200'" (error). D: E Panamá, NW Colombia. Lectotype ♂, NHMUK (Lamas, MS).
idae vespertina Fox, 1945: 1, fig. 21. TL: Ecuador, Los Ríos, Playas de Juan Montalvo, 30m. D: SW Colombia, W Ecuador. Holotype ♂, AMNH.
idae (C. Felder & R. Felder), 1862: 414 (in part) (*Mechanitis*). Misidentification.

isocomma isocomma Forbes, 1948: 6, pl. 1, fig. 2. TL: Colombia, [Villavicencio], upper Río Negro, 800m. D: E Colombia. Holotype ♂, CUIC.
isocomma simulator Fox, 1960: 162, pl. 5, fig. 69 (*Melinaea comma s*.). TL: Ecuador, [Pastaza], Puyo, 1000m. D: E Ecuador, E Perú. Holotype ♂, CMNH.

lilis dodona Hopffer, 1874: 344 (*Melinaea d*.). TL: "Bolivia" (error). D: W Colombia. Holotype ♀, MfN.
lilis imitata f. *erica* Bargmann, 1929: 141. TL: Colombia, [Valle del Cauca], Río Dagua. “Syntypes” (♂♀), NHMUK. Unavailable name.

lilis ezra Fox, 1939: 74. TL: Colombia, Magdalena, Minca. D: N Colombia, NW Venezuela. Holotype ♂, CMNH.

lilis flavicans Hoffmann, 1924: 70, fig. 3 (*Melinaea imitata f.*). TL: México, "Tabasco, Villa Hermosa" (error) [= Colima, Colima]. D: W México. Holotype ♀, AMNH.

lilis imitata Bates, 1864: 55 (*Melinaea i.*). TL: Guatemala, [Alta Verapaz, Río Polochic valley]. D: E México to Atlantic Costa Rica. Syntypes (♂), NHMUK.

tachypetis Herrich-Schäffer, 1865: 175. *Nomen nudum*.

tachypetis C. Felder & R. Felder, 1865: 355. TL: "México". Lectotype ♂, NHMW (Lamas, MS).

lilis kayei Brown, 1977: 167, fig. 24 (*Melinaea ethra k.*). TL: Guyana, [Mazaruni-Potaro], Quonga. D: Guyana. Holotype ♂, NHMUK.

lilis lateapicalis A. Hall, 1935: 227. TL: Venezuela, Mérida. D: N Colombia, SW Venezuela. Syntypes (2♂), NHMUK.

lilis lilis (Doubleday), 1847: 130, pl. 17, fig. 4 (*Mechanitis*). TL: "Venezuela". D: N Venezuela. Holotype ♂, NHMUK.

lilis limitata A. Hall, 1935: 227. TL: "Venezuela". D: NE Venezuela. Syntypes (2♂, 1♀), NHMUK. [raised to subspecies by Neild, 2008: 98 (18)]

lilis messatis (Hewitson), [1856]: [18], pl. [9], fig. 4 (*Mechanitis m.*). TL: "New Granada". D: E Panamá, N Colombia. Neotype ♂, NHMUK (Fox, 1965: 78 (23)), **reinst. stat.** [treated as a species by Le Crom & Winhard (2024) (4) on the basis of sympatry with *M. lilis dodona* in west Colombia; genomic data from two similar white (*M. lilis parallelis*) and yellow (*M. lilis imitata*) taxa that are sympatric in Panama grouped specimens by phenotype, although they were not differentiated in the mitochondrial DNA alone. Nevertheless, the split in the genomic tree is very shallow, more study is needed to determine the status of these phenotypes, and for the present we believe they are best treated as representing a single species]

lilis parallelis Butler, 1873: 155 (*Melinaea p.*). TL: Panamá, [Panamá, Lion Hill]. D: SE Costa Rica, Central Panamá. Holotype ♀, NHMUK.

lilis sola Kaye, 1925: 413 (*Melinaea mneme s.*). TL: Trinidad, [St. Patrick, Silverstream River]. D: Trinidad. Syntypes (♀), MGCL.

ludovica ludovica (Cramer), 1780: 17, 250, pl. 297, fig. E (*Papilio*). TL: "Surinam". D: S Venezuela, S Guyana, S Surinam, French Guiana, N Brazil, E Colombia, E Ecuador, NE Perú, E Bolivia. Syntypes (♂), NHMUK?

egina (Cramer), 1777: 144, 148, pl. 191, fig. D (*Papilio*). TL: "Surinam". Syntypes (♂), NHMUK? Junior homonym of *Papilio egina* Cramer, 1775.

ludovica manuelito Tessmann, 1928: 119, pl. 5, fig. 22 (*Melinaea m.*). TL: Perú, [Pasco], Pozuzo valley, 750m; between San Nicolás and Puchalina, Pichis road, ca. 1000m. D: E Perú. Lectotype ♂, MfN (Lamas, MS).

ludovica paraiya Reakirt, 1866: 242 (*Melinaea p.*). TL: Brazil, Rio de Janeiro; [Santa Catarina], Ilha de Santa Catarina. D: E Brazil. Syntypes (♂), FMNH.

maeonis maeonis Hewitson, 1869: 11. TL: Ecuador, [Pastaza], Sarayacu. D: E Colombia, E Ecuador, N Perú. Syntypes (♂), NHMUK, **rev. stat.**

strigilis Weymer, 1891: 280. TL: Unknown. Holotype ♂, MfN.

madeira aequatoriensis d'Almeida, 1951: 23, pl. 5, fig. 1. TL: "Ecuador". Holotype ♂, MNRJ (lost).

maeonis zamora Haensch, 1909: 123 (*Melinaea m.* f. *z.*). TL: "Perú, Ucayali" (error) [= Venezuela, Zamora (= Barinas)]. D: SW Venezuela. Lectotype ♂, MfN (Lamas, 1994: 296 (11)), **rev. stat.**

maeonis borealis Hall, 1935: 226. TL: Venezuela, [Mérida], Mucuchachí. Syntypes, NHMUK (3♂, 1♀).

marsaeus clara Rosenberg & Talbot, 1914: 672 (*Melinaea orestes c.*). TL: Perú, [Puno], Yahuarmayo, 1200'. D: SE Perú, NW Bolivia, SW Brazil. Holotype ♂, NHMUK. Fig.- Jordan (1923: pl. 4, fig. 9 (24)).

clara juruaënsis [sic] d'Almeida, 1943: 165, pl., fig. [1]. TL: Brazil, [Amazonas], Rio Juruá. Holotype ♂, MZUSP. [*m. clara* x *m. pothete*].

marsaeus macaria Godman & Salvin, 1898: 107 (*Melinaea macaria*). TL: Colombia, [Meta], San Martín, Llanos of Río Meta. D: E Colombia, E Ecuador, NE Perú. Syntypes (5♂), NHMUK, **reinst. stat.** [treated as a species by Le Crom & Winhard (2024) (4) on the basis of wing pattern differences in comparison with "*M. marsaeus*"; although there are no molecular data for the nominate subspecies, the genomic data for four other taxa, *macaria*, *rileyi*,

phasiana, and *clara*, support these as being conspecific, despite their wing pattern differences, so for the present we retain the previously existing taxonomy until further evidence suggests it should be modified]

macaria casanare Winhard, 2021: 478, fig. 1. Tl: Colombia, Casanare, Vereda Cunamá, 650m. Holotype ♀, ZSM, **n. syn.** [Winhard (2021) (25) stated that this taxon differed from *M. marsaeus macaria* in having reduced black in the wings, but this character is variable throughout the range of the species, and we regard the two listed type specimens from a single locality as insufficient evidence to conclude that they represent a consistent phenotype within a larger geographic area; the name was not mentioned by Le Crom & Winhard (2024) (4)]

marsaeus marsaeus (Hewitson), 1860: [28], pl. [14], fig. 10 (*Mechanitis*). TL: [Brazil], Amazonas, [Ega (= Tefé)]. D: W Brazil. Lectotype ♂, NHMUK (Lamas, 1994: 286 (11)).

maelus var. (Hewitson), 1860: [27] (in part), pl. [14], fig. 6 (*nec* 9) (*Mechanitis*).

manga Haensch, 1909: 123. TL: [Brazil], Amazonas, [Ega (= Tefé)]. Holotype ♀, NHMUK. *Nomen novum* for *Mechanitis maelus* var. Hewitson, 1860. [*m. marsaeus* x *m. phasiana*].

marsaeus phasiana Butler, 1870: 489 (*Melinaea p.*). TL: "Peruvian Amazons", [Loreto, Nauta]. D: E Perú, W Brazil. Syntypes (♀), NHMUK.

lucifer var. *divisa* Staudinger, 1885: 71. TL: Perú, [Loreto], Yurimaguas. Lectotype ♂, MfN (Lamas, MS). [*m. phasiana* x *m. rileyi*].

acreana d'Almeida, 1951: 22, pl. 4, fig. 2. TL: Brazil, Acre, alto Rio Juruá. Holotype ♂, MNRJ (lost). [*m. phasiana* x *m. rileyi* x *m. marsaeus*]

marsaeus pothete d'Almeida, 1945: 12, pl. 2, figs. 1-3. TL: Brazil, [Rondônia], Rio Verde. D: SW Brazil. Holotype ♂, MNRJ (lost).

marsaeus rileyi Fox, 1942: 3. TL: Perú, [Amazonas], upper Río Marañón, [Pongo de Manseriche]. D: SE Ecuador, SE Colombia, NE Perú, W Brazil. Holotype ♂, AMNH.

macaria rocafuerte Winhard, 2021: 478, figs. 4-5. TL: Ecuador, Orellana, Laguna Jatuncocha, 200m. Holotype ♀, ZSM, **n. syn.** [the female holotype has the FW yellow band tinged orange, like the type of *rileyi*, and given that *rileyi* was not mentioned in the original description, we see no reason to regard it as anything other than a specimen of *M. m. rileyi*]

menophilus cocana Haensch, 1903: 164. TL: Ecuador, [Napo], Coca. D: SE Colombia, E Ecuador, NE Perú. Lectotype ♂, MfN (Lamas, 1994: 278 (11)).

menophilus ernestoi Brown, 1977: 180, figs. 62-63. TL: Colombia, Caquetá, 40km above Florencia, 1000m. D: SE Colombia. Holotype ♂, MNRJ (lost), **reinst. stat.** [treated as a subspecies of *M.* "*zaneca*" [= *zaneka*] by Le Crom & Winhard (2024) (4), see discussion under that taxon]

menophilus hicetas Godman & Salvin, 1879: 150 (*Melinaea h.*). TL: Perú, [Loreto], Iquitos. D: E Colomibia, E Perú. Lectotype ♂, NHMUK (Lamas, 1994: 285 (11)), **reinst. stat.** [Le Crom & Winhard (2024) (4) treated this taxon as a species based on sympatry with *M. menophilus menophilus*, but genomic data of both phenotypes suggest that they are conspecific, and that the yellow FW apical spots that differentiate them represent variation that is partly local, partly geographic]

hicetas ab. *flavosignata* Staudinger, 1885: 71. TL: Perú, [Loreto], Pebas; Yurimaguas. "Lectotype" ♂, MfN (Lamas, MS). Unavailable name.

egesta Godman & Salvin, 1898: 107. TL: "Interior of Colombia"; Perú, [Loreto], Yurimaguas. Syntypes (4♂), NHMUK. [*m. hicetas* x *m*. ssp. n.]

magnifica Haensch, 1905: 145. TL: "Perú". Holotype ♂, MfN.

hicetas godmani Winhard & Le Crom, *in* Le Crom & Winhard, 2024: 5, 23, pl. 22. TL: [Colombia], Casanare, Vereda Cunamá, near Planbrisas, N of Aguazul, 650m. Holotype ♂, ZSM, **n. syn.** [Le Crom & Winhard (2024) (4) stated that the name *egesta* was invalid, representing a hybrid between subspecies, and, (presumably) assuming that the name otherwise would apply to an east Colombian taxon, proposed the name *godmani* based on specimens from Casanare, Cundinamarca and Meta; however, we know of no characters that might consistently differentiate *godmani* from *hicetas*, and none were described by Winhard & Le Crom (2024) (4)]

hicetas staudingeri Winhard & Le Crom, *in* Le Crom & Winhard, 2024: 5, 24, pl. 22. TL: [Colombia], Meta, Humadea, N of San Martín, 500m. Holotype ♂, ZSM, **n. syn.** [the reduced yellow FW apical spots described Le Crom & Winhard (2024) (4) as differentiating this taxon from *M.* "*hicetas godmani*" are variable within populations]

menophilus maenius (Hewitson), 1860: [27], pl. [14], fig. 7 (*Mechanitis maenius*). TL: [Brazil], Amazonas, [Tefé (= Ega)]. D: W Brazil. Syntypes (♂), NHMUK.

menophilus mediatrix Weymer, 1891: 282 (*Melinaea mneme* var. *mediatrix*). TL: French Guiana, Cayenne. D: S Venezuela, Guyana, Surinam, French Guiana, N Brazil. Lectotype ♂, MfN (de Lesse, 1970: 855 (26)).

mediatrix ab. *anina* Haensch, 1909: 123. TL: "Guyana". "Lectotype" ♂, MfN (Lamas, 1994: 275 (11)). Unavailable name.

menophilus menophilus (Hewitson), [1856]: [17] (in part), pl. [9], fig. 3 (*nec* 2) (*Mechanitis*). TL: "New Granada". D: E Colombia. Syntypes (♂), NHMUK.

ishka Butler, 1870: 489. TL: Colombia, "Bogotá" (error). Syntypes (♀), NHMUK.

menophilus membrosa Forbes, 1927: 33. TL: Unknown. Syntypes, CUIC?

menophilus orestes Salvin, 1871: 412 (*Melinaea o.*). TL: Perú, [Pasco], Pozuzo. D: E Perú, Bolivia, W Brazil. Syntypes (♂), NHMUK.

chincha Druce, 1876: 211, pl. 17, fig. 3. TL: Perú, [Pasco], Pozuzo. Syntypes (♂), NHMUK.

menophilus zaneka Butler, 1870: 490 (*Melinaea z.*). TL: Ecuador, [Napo], Archidona. D: E Colombia, E Ecuador, N Perú. Lectotype ♂, NHMUK (Lamas, 1994: 280 (11)), **reinst. stat.** [treated as a species by Le Crom & Winhard (2024) (4), there is some support for this hypothesis from the genomic data, which group this Andean foothill taxon with *M. m. ernestoi*, separate from lowland *M. menophilus* taxa; nevertheless, given the absence of sympatry between these phenotypes in well-sampled areas of possible overlap, we feel that they are better treated as conspecific; Le Crom & Winhard (2024) (4) stated that *M. m. zaneka* and *M. m. menophilus* are sympatric in Colombia, but the elevational ranges they provided (1400-2000m and 200-1300m, respectively) do not support this claim. Furthermore, the type specimens of the names *maculosa* and *discurrens* (see Warren et al. (2024) (14) under *M. m. zaneka*) may represent hybrids between *M. m. zaneka* and *M. m. menophilus*]

dora Strecker, 1876: 120. TL: Ecuador, "Esmeraldas" (error). Holotype ♂, FMNH.

zaneka ab. *maculosa* Haensch, 1903: 163. TL: Ecuador, [Tungurahua], Santa Inés. "Lectotype" ♂, MfN (Lamas, 1994: 285 (11)). Unavailable name.

zaneka ab. *discurrens* Haensch, 1909: 122. TL: Ecuador, [Tungurahua, Santa Inés]. "Lectotype" ♀, MfN (Lamas, 1994: 280 (11)). Unavailable name.

mnasias abitagua Brown, 1977: 164, fig. 11. TL: Ecuador, [Napo], km 43 Puyo-Napo road, 600m. D: E Ecuador. Holotype ♀, MNRJ (lost).

mnasias comma Forbes, 1927: 34, pl. 2, fig. [1]; pl. 3, fig. [13] (*Melinaea c.*). TL: Perú, [Junín], Chanchamayo. D: E Perú. Holotype ♂, CUIC.

mnasias eratosthenes A. Hall, 1935: 226, pl. 6, fig. 5 (*Melinaea e.*). TL: "French Guiana". D: SE Venezuela, French Guiana, N Brazil. Holotype ♂, NHMUK.

mnasias lucifer Bates, 1862: 551 (*Melinaea l.*). TL: Brazil, [Amazonas], São Paulo [de Olivença]. D: NE Perú, W Brazil. Syntypes (♂♀), NHMUK, **reinst. stat.** [treated as a subspecies of *Melinaea* (*marsaeus*) *macaria* by Le Crom & Winhard (2024: 23) (4) on the basis of similarity in wing pattern, the figured putative Colombian specimen of *lucifer* differs from the type of *Melinaea lucifer* (see Warren et al. 2024 (14)) in the shape of the FW postdiscal band and in lacking orange FW marginal spots, and actually represents a specimen of *M. marsaeus rileyi*]

hicetas eryx d'Almeida, 1951: 24, pl. 3, fig. 3. TL: Brazil, Acre, alto Rio Juruá. Holotype ♂, MNRJ (lost). [*m. lucifer* x *m. romualdo*].

mnasias lutzi Fox, 1942: 1, fig. 2 (*Melinaea lucifer l.*). TL: Perú, [Amazonas], upper Río Marañón, [Quebrada Huachinza]. D: N Perú. Holotype ♂, AMNH.

mnasias mnasias (Hewitson), [1856]: [18], pl. [9], fig. 5 (*Mechanitis*). TL: [Brazil, Pará, Pará (= Belém)]. D: Brazil (Pará). Syntypes (♂), NHMUK.

mnasias neblinae Brown, 1977: 164, fig. 12. TL: Brazil, Amazonas, Pico Neblina, 1500m. D: S Venezuela, NW Brazil. Holotype ♂, MIZA.

mnasias romualdo Fox, 1965: 80 (*Melinaea comma r.*). TL: Perú, [Puno], "Carabaya, Río Huacamayo, La Unión, 2000'" [= Yahuarmayo, 1200']. D: SE Perú. Holotype ♂, NHMUK.
mnasias rondonia Brown, 1977: 166, fig. 13. TL: Brazil, Rondônia, near Riozinho, 300m. D: SW Brazil. Holotype ♂, MNRJ (lost).
mnasias tecta Haensch, 1909: 124, pl. 33d, fig. [4] (as *mnasias*) (*Melinaea thera* f. *tecta*). TL: "Guyana". D: Guyana. Holotype ♂, MfN.
mnasias thera C. Felder & R. Felder, 1865: 354 (*Melinaea t.*). TL: Unknown. D: SE Brazil. Holotype ♂, NHMUK.
thera Herrich-Schäffer, 1865: 175. *Nomen nudum*.

mneme mauensis Weymer, 1891: 283 (*Melinaea mneme* var. *mauensis*). TL: Brazil, [Amazonas], Ega (= Tefé). D: S Venezuela, E Colombia, NE Perú, Brazil. Holotype ♂, MfN.
mneme mneme (Linnaeus), 1763: 20 (*Papilio*). TL: "China" (error). D: ?SE Venezuela, Guyana, Surinam, French Guiana, N Brazil. Lectotype ♂, LSL (Kaye, 1925: xxii (27)).

mnemopsis Berg, 1897: 234. TL: "Perú" (error?). D: ?SE Perú, Bolivia. Holotype ♂, MBR.
boliviana Weymer, 1907: 2, pl. 1, fig. 2. TL: "Bolivia". Holotype ♂, MfN.

mothone messenina C. Felder & R. Felder, 1865: 356, pl. 45, fig. 11 (*Melinaea messenina*). TL: Colombia, "Bogotá" (error). D: E Colombia, W Venezuela. Syntypes (♂), NHMUK.
mothone mothone (Hewitson), 1860: [30], pl. [15], fig. 14 (*Mechanitis*). TL: [“Ecuador”]. D: SE Colombia, E Ecuador, NE Perú. Neotype ♂, NHMUK (Fox, 1965: 79 (23)).
cydippe Salvin, 1871: 412. TL: Ecuador, [Morona-Santiago], Gualaquiza; Perú, [Pasco], Pozuzo. Syntypes (♂), NHMUK.

satevis aurantia Forbes, 1942: 27 (*Melinaea mneme a.*). TL: Venezuela, Monagas, Caripito. D: NE Venezuela, Trinidad. Syntypes (7♂), CUIC (3♂), MIZA (1♂), USNM (2♂), CMNH (1♂).
satevis crameri Godman & Salvin, 1898: 107 (*Melinaea c.*). TL: Guyana, [Mazaruni-Potaro, Mt. Roraima]. D: E Venezuela, N Guyana, N Surinam. Syntypes (5♂), NHMUK.
mneme (Cramer), 1777: 142, 149, pl. 190, fig. C (*Papilio*). Misidentification.
crameri ab. *incisa* Kaye, 1925: xxiii. TL: Guyana, [Mazaruni-Potaro], Essequibo River. “Syntypes” (♂), MGCL. Unavailable name.
satevis cydon Godman & Salvin, 1879: 151 (*Melinaea c.*). TL: Brazil, [Amazonas], Tabatinga; Perú, [Loreto], Pebas. D: SE Colombia, E Perú, W Brazil. Syntypes (♂), NHMUK.
satevis flavomacula Weymer, 1894: 322 (*Melinaea pardalis* var. *f.*). TL: [Brazil], "obern Amazonenstrom". D: Brazil. Lectotype ♂, MfN (Lamas, 1994: 285 (11)).
madeira Kaye, 1904: 4. *Nomen nudum*.
pardalis madeira Moulton, 20.i.1909: 597, 604, pl. 32, fig. 2. TL: [Brazil], "Amazons". Holotype ♀, OXF.
madeira Haensch, 26.v.1909: 123, pl. 33b, fig. [3]. TL: [Brazil, Amazonas], Manicoré. Lectotype ♂, MfN (Lamas, 1994: 285 (11)). Junior primary homonym of *Melinaea pardalis madeira* Moulton, 1909.
satevis lamasi Brown, 1977: 171, figs. 31-32 (*Melinaea ethra l.*). TL: Perú, Madre de Dios, Iberia, 200m. D: SE Perú, SW Brazil. Holotype ♂, MUSM.
satevis maelus (Hewitson), 1860: [27] (in part), pl. [14], fig. 9 (*nec* 6) (*Mechanitis m.*). TL: [Brazil], Amazonas, [Ega (= Tefé)]. D: E Colombia, NE Perú, W Brazil. Syntypes (♂♀), NHMUK.
pardalis (C. Felder & R. Felder), iii.1862: 78 (*Mechanitis*). *Nomen nudum*.
pardalis Bates, 13.xi.1862: 552. *Nomen novum*.
hicetas brunnea Riley, 1919: 181. TL: Brazil, [Amazonas], upper Rio Purús, Boca do Acre. Holotype ♀, NHMUK. [*s. maelus* x *s. lamasi*]
hicetas purusana Riley, 1919: 181. TL: Brazil, [Amazonas], Rio Purús, Aliança, Canutama, Sebastopol, Lábrea; [Rondônia], Abunã. Syntypes (♂♀), NHMUK. [*s. maelus* x *s. flavomacula*]

madeira var. *purusana* Aurivillius, 1929: 155. TL: Brazil, [Amazonas], Rio Purús, Hiutanaã. Lectotype ♂, RMS (Lamas, MS). Junior primary homonym of *Melinaea hicetas purusana* Riley, 1919. [*s. maelus* x *s. flavomacula*].

maelus romani Bryk, 1937: 641. *Nomen novum* for *Melinaea madeira* var. *purusana* Aurivillius, 1929, *nec M. hicetas purusana* Riley, 1919.

satevis satevis (Doubleday), 1847: 130, pl. 17, fig. 3 (*Mechanitis*). TL: "Bolivia". D: ?SE Perú, Bolivia. Holotype ♀, NHMUK.

scylax Salvin, 1871: 412. TL: Panamá, Chiriquí, Bugaba, [800-1000']. D: Pacific Costa Rica, Pacific W Panamá, NW Colombia. Lectotype ♂, NHMUK (Lamas, MS).

ribbei Staudinger, v.1875: 97. TL: Panamá, Chiriquí. Lectotype ♂, MfN (Lamas, MS).

ribbei Weymer, vii.1875: 379, pl. 2, fig. 4. TL: Panamá, Chiriquí. Lectotype ♂, MfN (Lamas, MS). Junior primary homonym of *Melinaea ribbei* Staudinger, 1875.

tarapotensis Haensch, 1909: 122 (*Melinaea menophilus* f. *t.*). TL: [Perú, San Martín], Tarapoto. D: NE Perú. Lectotype ♀, MfN (Lamas, 1994: 294 (11)).

Interspecific hybrids

agricola Hall, 1935: 227, pl. 6, fig. 6. TL: Brazil, [Amazonas], Ega (= Tefé). Holotype ♂, NHMUK. [*ludovica ludovica* x *mneme mauensis*]

mayi d'Almeida, 1951: 20, pl. 4, fig. 1. TL: Brazil, Acre, alto Rio Juruá. Holotype ♂, MNRJ (lost). [*marsaeus marsaeus* x *mneme mauensis*].

Nomina nuda

eginella Herrich-Schäffer, 1865: 175.
vilis Herrich-Schäffer, 1865: 175.

Literature cited: (2, 4, 7–27)

Collection codens

AMNH: American Museum of Natural History, New York, NY, USA
CCWM: Chau Chak Wing Museum, University of Sydney, Sydney, Australia.
CMNH: Carnegie Museum of Natural History, Pittsburgh, USA
CUIC: Cornell University, Ithaca, USA
DZUP: Departamento de Zoologia, Universidade Federal do Paraná, Curitiba, Brazil
FMNH: Field Museum of Natural History, Chicago, IL, USA
IAVH: Instituto Alexander von Humboldt, Bogotá, Colombia
ICNB: Instituto de Ciencias Naturales, Universidad Nacional, Museo de Historia Natural, Bogota, Colombia
IOC: Instituto Oswaldo Cruzo, Rio de Janeiro, Brazil
LSL: Linnaean Collection, Linnaean Society of London, London, United Kingdom
MBR: Museo Argentina de Ciencias Naturales "Bernardino Rivadavia", Buenos Aires, Argentina
MfN: Museum für Naturkunde, Leibniz-Institut für Evolutions- und Biodiversitätsforschung an der Humboldt Universität, Berlin, Germany
MGCL: McGuire Center for Lepidoptera and Biodiversity, Florida Museum of Natural History, Gainesville, USA

MIZA: Museo del Instituto de Zoología Agrícola, Universidad Central de Venezuela, Maracay, Venezuela
MNCN: Museo Nacional de Ciencias Naturales, Madrid, Spain
MNHN: Muséum National d'Histoire Naturelle, Paris, France
MNRJ: Museu Nacional Rio de Janeiro, Rio de Janeiro, Brazil
MPUJ: Museo de Historia Natural de la Pontificia Universidad Javeriana, Bogotá, Colombia
MUSM: Museo de Historia Natural, Universidad Nacional Mayor de San Marcos, Lima, Peru
NHMUK: Natural History Museum, London, UK
OXF: Oxford University Museum, Oxford, UK
RMS: Rijksmuseum, Amsterdam, Netherlands
RPM: Reading Public Museum, Philadelphia, USA
SMTD: Staatliches Museum für Tierkunde, Dresden, Germany
UCaldas: Museo de Historia Natural, Universidad de Caldas, Manizales, Colombia
USNM: National Museum of Natural History, Smithsonian Institution, Washington, DC, USA
UUZM: Uppsala University, Uppsala, Sweden
ZSM: Zoologische Staatssammlung München, Munich, Germany
ZUEC: Museu de Zoologia da Universidade Estadual de Campinas 'Adão José Cardoso', Campinas, Brazil

Text S3. Hybridisation analysis

The poorly supported nodes and discordance between phylogenies constructed with different methods (Fig. 1 vs Fig. 2), from nuclear versus mitochondrial genomes (Fig. 1) or different genomic regions (Fig. S10) could be due to either ILS or hybridisation. To test for hybridisation, we assessed excess allele sharing between non-sister taxa with Fbranch (Fig. S11) and windowed phylogenetic tree discordance with BPP (Table S2; Fig. S10; Text S2). Moreover, we ran AIM which uses MCMC models to produce a phylogenetic tree that allows for gene flow (Fig. S12). The combined results of all hybridisation analyses are summarised with arrows in Fig. 2 (Text S2), but we would like to stress that this is only one of the multiple possible scenarios consistent with our observed patterns of excess allele sharing and gene tree discordance. Due to hybridisation, there is likely not 'one true species tree' that reflects the 'real' phylogeny of these genera (28, 29).

Mechanitis

In *Mechanitis*, various analyses show that there has been ancient hybridisation between *Mec. messenoides* and the ancestor of the *polymnia-lysimnia-nesaea*-clade*.* The IQtree2 nuclear phylogeny places *messenoides* as a sister-species of *menapis* (Fig. 1A), but BEAST2 places *messenoides* as the sister-species of *menapis*, *macrinus* and *mazaeus* (Fig. 2A). AIM places *messenoides* closer to the *polymnia-lysimnia-nesaea*-clade (Fig. S12A) with various admixture arrows between the *menapis-macrinus-mazaeus* clade and the *messenoides-polymnia-nesaea-lysimnia* clade (Fig. S12A – arrows B-D). The IQtree2 mitochondrial phylogeny shows that *messenoides* has two mitochondrial haplotypes, one clustering with *menapis* and the other with *polymnia*, *lysimnia* and *nesaea* (Fig. 1A – box II). We performed an Fbranch-analysis (with the BEAST2-tree as the 'species'-tree) to assess allele sharing, and this confirms excess allele sharing between *Mec. messenoides* and *Mec. menapis*, as well as *Mec. messenoides* and the *polymnia*-clade (Fig. S11A - #2-3). *Mec. messenoides* co-occurs with *mazaeus*, *polymnia* and *lysimnia* in Western Amazonia, whereas *Mec. menapis* co-occurs with *macrinus* and *polymnia* West of the Andes.

There also appears to have been admixture within the *polymnia-lysimnia*-nesaea-clade. *Mec. nesaea* co-occurs with *polymnia* and *lysimnia* in the Atlantic Forest of Brazil, and both *polymnia* and *lysimnia* have subspecies across South America. *Mec. nesaea* is placed in a different position in the phylogenetic tree with the nuclear IQtree2 (Fig. 1A) and BEAST2 (Fig. 2A) (as sister to *polymnia* and *lysimnia*, respectively), and also the AIM phylogeny shows a different topology (Fig. S12A) (*polymnia* and *lysimnia* as closest sister-species). The mitochondrial IQtree2 phylogeny places *nesaea* as sister to *lysimnia* (Fig. 1A – box III). The Fbranch analysis confirms excess allele sharing between *Mec. nesaea, Mec. polymnia* and *Mec. lysimnia* (Fig. S11A - #1)*.* There is excess allele sharing between *nesaea* and Brazilian *polymnia*, as well as Brazilian *lysimnia* and Brazilian *polymnia* compared to the rest of *polymnia* (Fig. S11A - #1B, #1C). Interestingly, however, the Ecuadorian *lysimnia* share more alleles with *Mec. nesaea* than Brazilian *lysimnia* (Fig. S11A - #1A). The Ecuadorian *lysimnia* also share more alleles with all of the polymnia-lineages, compared to the Brazilian *lysimnia* (Fig. S11A - #1A).

The BPP-analysis, where we plotted phylogenies in windows across the genome, confirms both the jumping of *messenoides*, and the uncertainty within *polymnia-lysimnia-nesaea*. The three most common trees in the genome vary from each other by the relationship amongst *Mec. lysimnia, Mec. polymnia* and *Mec. nesaea* (Fig. S10; accounting for ~41% of the genome). These three trees place *Mec. messenoides* and *Mec. menapis* as closest relatives, which is the topology we found with the IQtree2 analysis, and what the Fbranch analysis confirms with excess allele sharing between *messenoides* and *menapis* (Fig. S11A - #2). According to the BPP-analysis, *Mec. messenoides* clusters in the same clade as *Mec. menapis, mazaeus* and *macrinus* in ~51% of the genome (Fig. S10B). However, in almost ~20% of the genome, *Mec. messenoides* is placed closer to or within the clade consisting of *Mec. polymnia*, *Mec. lysimnia* and *Mec. nesaea*, and not with *Mec. menapis*. The branching order within the *polymnia-lysimnia-nesaea* clade is highly variable across the genome*, with* 46.3% of the genome grouping *polymnia* with *nesaea*, 31.7% grouping *polymnia* with *lysimnia*, and 14.7% grouping *nesaea* with *lysimnia* (Fig. S10C).

In the Fbranch-analysis, another signal of excess allele sharing is found, namely between the *mazaeus-macrinus*-clade and the *polymnia-lysimnia-nesaea*-clade (Fig. S11A – #4). AIM also indicates some gene flow between these two clades (Fig. S12A – arrow E). *Mec. polymnia* and *nesaea* share more alleles with *Mec. mazaeus* compared to *Mec. macrinus*.

The relationships between the various *polymnia*-lineages shift around in the various analyses. With IQtree2, *polymnia* from west of the Andes is closest to the individuals from Western Amazonia, but with BEAST2 *polymnia-West* is

the earliest diverging lineages in the *polymnia*-clade. Fbranch confirms this admixture (Fig. S11A - #5). We found a single individual in Colombia with shared ancestral contribution of lineages East and West of the Andes (Sal_4944 in Fig. S1; Fig. S8).

Melinaea

In *Melinaea*, we find hybridisation between various species and low concordance factors at all nodes, even at the base of the core clade (Fig. 1B). IQtree2 and BEAST produce the same topology, but AIM places *Mel. idae* with another clade and suggests gene flow between *Mel. idae* and the two different sister clades (Fig. 2B - arrow a; Fig. S12B – arrow A), which is confirmed by the Fbranch results (Fig. S11B - #1). We ran the BPP analysis for *Melinaea* in a subset of the species, as including individuals of all species created so many different trees across the genome that the results were difficult to interpret, likely due to the recent divergence and gene flow. To resolve the position of *Mel. idae*, as well as putative introgression between deeper branches of the phylogeny, we focused on the relationships between the *lilis-isocomma-idae* clade and representatives of the other clade (*mneme*, *marsaeus* and *mothone*; thus excluding *satevis*, *tarapotensis*, *maeonis* and *menophilus*) (Fig. S10E-F). The three most common trees in this analysis switch *Mel. idae* from a sister group to *lilis* (~15%; indicating admixture between *lilis* and *idae*, confirmed by Fbranch excess allele sharing; Fig. S10; Fig. S11B - #4), to *idae* as the sister to *mneme-marsaeus-mothone* (like the AIM-results; ~12%), to *idae* as sister to both *lilis* and *isocomma* (like IQTree2 and BEAST2; ~10%).

In the majority of the genome (43.4%) *lilis, idae* and *isocomma* group together (like IQTree2 and BEAST2), while in 34.5% of the genome *idae* groups with *mothone-marsaeus-mneme* (like AIM). Notably, the Z chromosome supports almost exclusively the latter relationship. In 4.3% of the genome, *isocomma* clusters with *mothone-marsaeus-mneme*. *Lilis* and *isocomma* (38.3%) are almost as often sister-species compared to *lilis* and *idae* (30.3%), both a lot more than *idae* and *isocomma* (4.1%), indicating admixture between *lilis* and *idae*, which is confirmed by Fbranch excess allele sharing (Fig. S10B; Fig. S11B - #2). *Mel. mothone, marsaeus* and *mneme* also vary in their respective relationships (Fig. S10E). *Mel. idae* and *Mel. lilis* are both found west of the Andes, whereas all other clades are found east of the Andes.

Moreover, AIM also places *Mel. satevis* closer to *marsaeus-maeonis* instead of *tarapotensis* (Fig. 2B - arrow c; Fig. S12B; Fig. S11B - #3), and Fbranch also indicates some excess allele sharing between *Mel. marsaeus/maeonis* and *Mel. isocomma* and *Mel. mothone* (Fig. S11B - #2,#7). Both AIM and Fbranch indicate admixture between *Mel. ludovica* and several clades in the 'core' *Melinaea* (Fig. 2B - arrows e-f; Fig. S12B – arrows E/F; Fig. S11B - #5-6). BPP always places *Mel. ludovica* as the outgroup in the phylogeny (Fig. S10). There is a slight excess of allele sharing between the *mothone/menophilus*-clade and the *lilis/isocomma/idae*-clade (Fig. S11B; #10). Overall, these results indicate that there has been rampant admixture between many *Melinaea* lineages, and that especially *Mel. idae* has been strongly introgressed.

Text S4. ***Mechanitis nesaea*** **– an ancestrally introgressed species**

Mechanitis nesaea occurs in the eastern tip of the Brazilian Atlantic Forest. *Mec. lysimnia lysimnia* occurs further south, with a small sympatric zone, and *Mec. polymnia casabranca* largely overlaps with both taxa (Fig. 2) (2, 30). *Mec. nesaea* is intermediate between *Mec. polymnia* and *Mec. lysimnia* in chromosome number (31), *lysimnia*-like in wing colour patterns and caterpillar phenotypes, although the head capsule colour of later caterpillar instars is sometimes more *polymnia*-like (32) (Fig. 3A). We find that in androconial compounds, *Mec. nesaea* is distinct from both putative parental species but closest to *Mec. polymnia* (Fig. 3B, Table S3-4). Some of the differences in androconial compounds might contribute to assortative mating, as has been suggested in other Ithomiini (33–35). Of the 26 androconial compounds we found, three show very high concentrations, two of which are derivatives of PAs: hydroxydanaidal (peak 2) and methyl hydroxydanaidoate (peak 3) (Fig. 3C). The content of these chemicals can vary greatly between individuals, because it depends on the access to PA-containing plants, but we find them to be consistently associated with *Mec. nesaea* and *Mec. p. casabranca* and present only in traces in *Mec. l. lysimnia* (across 92 individuals total from multiple locations, Table S3), consistent with previous findings of *Mec. l. lysimnia* (36). On the other hand, heptacosene (peak 12) was dominant in *Mec. l. lysimnia* while only present as traces in *Mec. nesaea* and absent in *Mec. p. casabranca* samples.

To study genomic signatures of differentiation and introgression and to examine whether introgression of *lysimnia* into *nesaea* contributed to reproductive isolation between *nesaea* and *polymnia*, we resequenced 15 *Mec. nesaea*, 19 *Mec. l. lysimnia* and 9 *Mec. p. casabranca*, of which 24 many were sampled from sympatry. We examined patterns of ADMIXTURE, F_{ST}, D_{XY} and f_{dM}. ADMIXTURE and high F_{ST} shows no evidence of recent gene flow between either *lysimnia* and *nesaea*, or *polymnia* and *nesaea* (Fig. S8; Fig. S15), and introgression plots are consistent between various *nesaea*-individuals (Fig. S16), confirming that *lysimnia*-introgression is likely ancient or ancestral to *Mec. nesaea*. We find high peaks of differentiation that overlap with peaks of introgression between *lysimnia* and *nesaea* and also with chromosomal breakpoints (Fig. 3G; Fig. S17), but as we find overall low recent gene flow between *nesaea* and *polymnia*, it is difficult to interpret our F_{ST} and f_{dM} results. We conclude that more research is necessary, and future studies should test if high-differentiation peaks with signatures of *lysimnia*-introgression harbour reproductive isolation barriers such as genes contributing to chemoreceptor differences.

It is interesting to note that *Mec. lysiminia lysiminia* and *Mec. polymnia casabranca* have been observed to interbreed in nature (37) and putative hybrids have been found, some phenotypically resembling *Mec. nesaea* (Fig. S14). It might be the case that all putative hybrids are infertile F1-individuals, and that recent introgression is unlikely to spread.

Text S5. Methods

Androconial Chemistry

We obtained samples of the androconial secretions from 92 males. To obtain samples of the androconial secretions, both wing androconia of each sampled male were excised and soaked into 1.5 mL bidistilled HPLC grade hexane. The extracts were filtered with silanized glass wool (Sigma-Aldrich, USA) to remove any debris, and then concentrated to approximately 25 µL under a gentle nitrogen flow. The samples were analysed on a gas chromatograph coupled to a mass spectrometer (GC-MS; Agilent 7890A™ gas chromatograph, Agilent 5975C Series MSD™ mass spectrometer) equipped with a non-polar DB-5ms column (Agilent J&W; 30 m × 0.25 mm i.d., 0.25 µm film thickness). A split/splitless inlet was fitted to an Agilent Thermal Separation Probe (TSP). For the analyses, 1 µL aliquots were injected into quartz micro vials which were then inserted in the TSP vial holder with the inlet set to split mode (1:1) and the GC injector temperature set to 250 °C. The GC oven temperature started at 60 °C for 2 min and then increased at a rate of 10 $°C \cdot min^{-1}$ to 280 °C. The final temperature was maintained for 12 min. Helium carrier gas flow was maintained at a constant pressure of 7.3 psi. MS source and quadrupole temperatures were set at 230 °C and 150 °C, respectively. Mass spectra were taken at 70 eV (in EI mode) with a scanning speed of 1.0 $scan \cdot s^{-1}$ from m/z 35–450. To exclude non-androconial compounds from our analyses, the same extraction procedure was adopted to excise wing areas of conspecific females (n=2 / population) and non-androconial wing areas of males (n=2 / population). Solvent negative controls were also taken for each sampling event (n=8).

The peak areas of each chromatogram were integrated with MSD ChemStation E.02.01.1177 (Agilent Technologies, USA) to obtain the total ion current signals. A series of linear alkanes (C7–C40) was used to determine the linear retention indices (RI) of each compound (38). Compounds were identified by comparing their mass spectra and retention indices with those of reference samples available from personal and commercial mass spectral libraries (FFNSC 2, MassFinder 4, NIST17, MACE v.5.1 (39), and Wiley Registry™ 9th ed.). The peaks exclusive to the androconial samples were used to determine the relative percentages of each compound per sample. Dihydropyrrolizines, such as hydroxydanaidal or methyl hydroxydanaidoate, are typically accompanied by smaller peaks formed by degradation during GC/MS analyses and/or storage (36). These degradation peaks were excluded from the generated ion chromatograms and statistical analysis. To avoid non-evident contaminants, we only considered compounds present in more than three individuals for any given taxon. The data were plotted in R (40) using the vegan package (https://doi.org/10.32614/CRAN.package.vegan) for nonmetric multidimensional scaling with 'monoMDS', specifying a global model, square root transformation and Wisconsin double standardisation (autotransform=TRUE).

Reference genomes

High molecular weight DNA was extracted from half a thorax using the Qiagen MagAttract High Molecular Weight kit (Qiagen ID 67563), followed by shearing into an average fragment size between 12–20 kb in a Megaruptor 3 system. The sheared DNA was used for low-input PacBio library preparation according to the manufacturers' instructions (see Tree of Life protocol (41)). Pacific Biosciences HiFi circular consensus libraries were constructed and sequencing was performed on PacBio Revio. Next, we used the head for HiC library prep using Arima v2 kit following the manufacturer's protocol, followed by 150 bp paired-end Illumina sequencing on a Novaseq 6000 performed by Scientific Operations at Wellcome Sanger Institute.

The genomes were assembled with Hifiasm version 0.19.5 (42), duplicates removed with purge_dups version 1.2.5 (43). The draft assembly was scaffolded with HiC-data using YaHS version 1.2a.2 (44), and both haplotypes were assembled separately. Contamination check and correction was performed with the TreeVal pipeline version 1.0.0 (45), thereafter the assemblies were manually curated with Higlass (46) and PretextView (https://github.com/sanger-tol/PretextView). Post-processing and evaluation of the genome assemblies were performed with three pipelines: sanger-tol/readmapping (47), sanger-tol/genomenote (48), and sanger-tol/blobtoolkit (49).

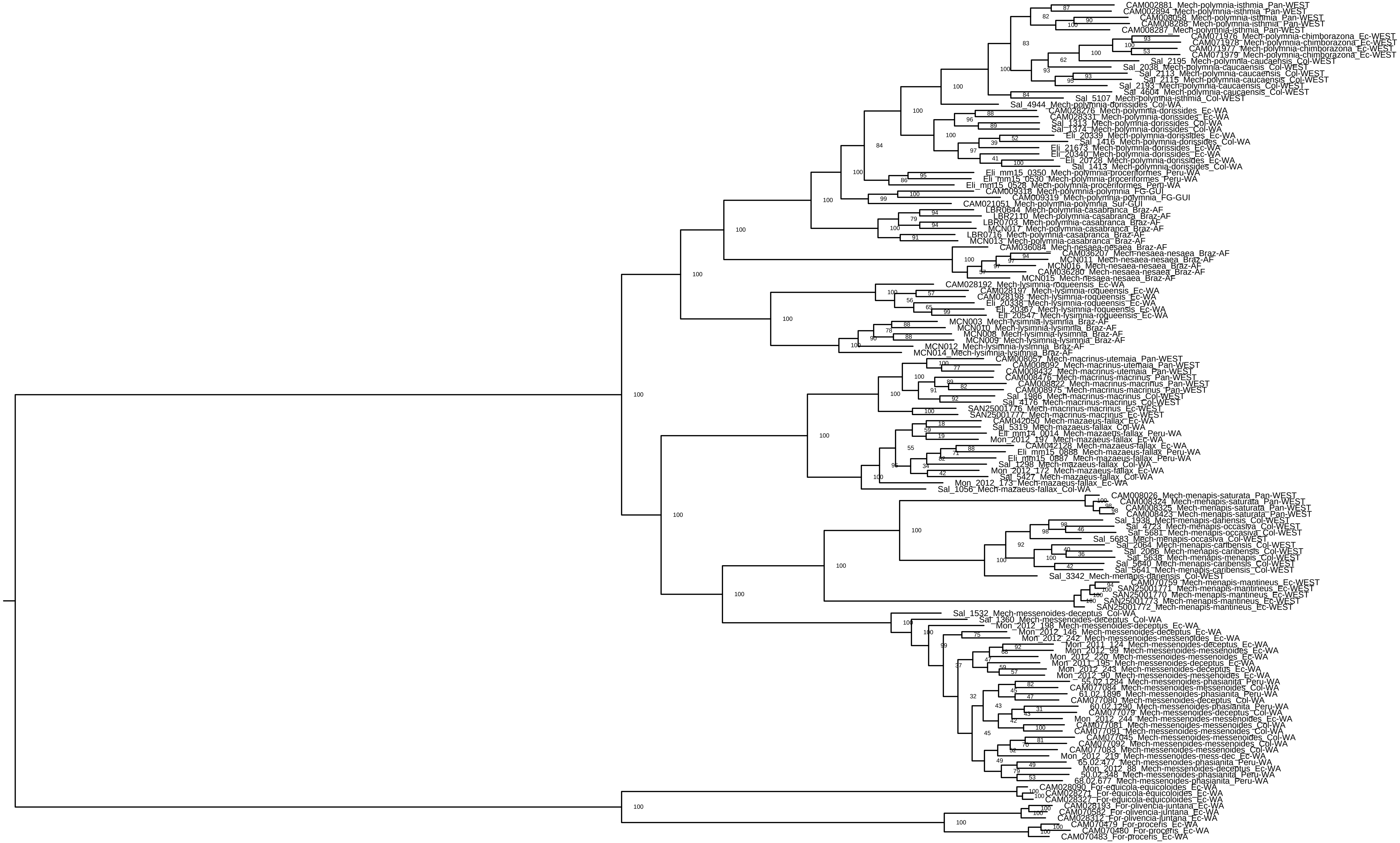

CAM002881_Mech-polymnia-isthmia_Pan-WEST
CAM002894_Mech-polymnia-isthmia_Pan-WEST
CAM008058_Mech-polymnia-isthmia_Pan-WEST
CAM008288_Mech-polymnia-isthmia_Pan-WEST
CAM008287_Mech-polymnia-isthmia_Pan-WEST
CAM071976_Mech-polymnia-chimborazona_Ec-WEST
CAM071978_Mech-polymnia-chimborazona_Ec-WEST
CAM071977_Mech-polymnia-chimborazona_Ec-WEST
CAM071979_Mech-polymnia-chimborazona_Ec-WEST
Sal_2195_Mech-polymnia-caucaensis_Col-WEST
Sal_2038_Mech-polymnia-caucaensis_Col-WEST
Sal_2113_Mech-polymnia-caucaensis_Col-WEST
Sal_2115_Mech-polymnia-caucaensis_Col-WEST
Sal_2193_Mech-polymnia-caucaensis_Col-WEST
Sal_4604_Mech-polymnia-caucaensis_Col-WEST
Sal_5107_Mech-polymnia-isthmia_Col-WEST
Sal_4944_Mech-polymnia-dorissides_Col-WA
CAM028276_Mech-polymnia-dorissides_Ec-WA
CAM028331_Mech-polymnia-dorissides_Ec-WA
Sal_1313_Mech-polymnia-dorissides_Col-WA
Sal_1374_Mech-polymnia-dorissides_Col-WA
Eli_20339_Mech-polymnia-dorissides_Ec-WA
Sal_1416_Mech-polymnia-dorissides_Col-WA
Eli_21673_Mech-polymnia-dorissides_Ec-WA
Eli_20340_Mech-polymnia-dorissides_Ec-WA
Eli_20728_Mech-polymnia-dorissides_Ec-WA
Sal_1413_Mech-polymnia-dorissides_Col-WA
Eli_mm15_0350_Mech-polymnia-proceriformes_Peru-WA
Eli_mm15_0530_Mech-polymnia-proceriformes_Peru-WA
Eli_mm15_0528_Mech-polymnia-proceriformes_Peru-WA
CAM009318_Mech-polymnia-polymnia_FG-GUI
CAM009319_Mech-polymnia-polymnia_FG-GUI
CAM021051_Mech-polymnia-polymnia_Sur-GUI
LBR0644_Mech-polymnia-casabranca_Braz-AF
LBR2110_Mech-polymnia-casabranca_Braz-AF
LBR0703_Mech-polymnia-casabranca_Braz-AF
MCN017_Mech-polymnia-casabranca_Braz-AF
LBR0716_Mech-polymnia-casabranca_Braz-AF
MCN013_Mech-polymnia-casabranca_Braz-AF
CAM036084_Mech-nesaea-nesaea_Braz-AF
CAM036207_Mech-nesaea-nesaea_Braz-AF
MCN011_Mech-nesaea-nesaea_Braz-AF
MCN016_Mech-nesaea-nesaea_Braz-AF
CAM036280_Mech-nesaea-nesaea_Braz-AF
MCN015_Mech-nesaea-nesaea_Braz-AF
CAM028192_Mech-lysimnia-roqueensis_Ec-WA
CAM028197_Mech-lysimnia-roqueensis_Ec-WA
CAM028198_Mech-lysimnia-roqueensis_Ec-WA
Eli_20338_Mech-lysimnia-roqueensis_Ec-WA
Eli_20367_Mech-lysimnia-roqueensis_Ec-WA
Eli_20547_Mech-lysimnia-roqueensis_Ec-WA
MCN003_Mech-lysimnia-lysimnia_Braz-AF
MCN010_Mech-lysimnia-lysimnia_Braz-AF
MCN008_Mech-lysimnia-lysimnia_Braz-AF
MCN009_Mech-lysimnia-lysimnia_Braz-AF
MCN012_Mech-lysimnia-lysimnia_Braz-AF
MCN014_Mech-lysimnia-lysimnia_Braz-AF
CAM008057_Mech-macrinus-utemaia_Pan-WEST
CAM008092_Mech-macrinus-utemaia_Pan-WEST
CAM008432_Mech-macrinus-utemaia_Pan-WEST
CAM008476_Mech-macrinus-macrinus_Pan-WEST
CAM008822_Mech-macrinus-macrinus_Pan-WEST
CAM008975_Mech-macrinus-macrinus_Pan-WEST
Sal_1986_Mech-macrinus-macrinus_Col-WEST
Sal_4176_Mech-macrinus-macrinus_Col-WEST
SAN25001776_Mech-macrinus-macrinus_Ec-WEST
SAN25001777_Mech-macrinus-macrinus_Ec-WEST
CAM042050_Mech-mazaeus-fallax_Ec-WA
Sal_5319_Mech-mazaeus-fallax_Col-WA
Eli_mm14_0014_Mech-mazaeus-fallax_Peru-WA
Mon_2012_197_Mech-mazaeus-fallax_Ec-WA
CAM042128_Mech-mazaeus-fallax_Ec-WA
Eli_mm15_0888_Mech-mazaeus-fallax_Peru-WA
Eli_mm15_0887_Mech-mazaeus-fallax_Peru-WA
Sal_1298_Mech-mazaeus-fallax_Col-WA
Mon_2012_172_Mech-mazaeus-fallax_Ec-WA
Sal_5427_Mech-mazaeus-fallax_Col-WA
Mon_2012_173_Mech-mazaeus-fallax_Ec-WA
Sal_1056_Mech-mazaeus-fallax_Col-WA
CAM008026_Mech-menapis-saturata_Pan-WEST
CAM008324_Mech-menapis-saturata_Pan-WEST
CAM008325_Mech-menapis-saturata_Pan-WEST
CAM008423_Mech-menapis-saturata_Pan-WEST
Sal_1938_Mech-menapis-dariensis_Col-WEST
Sal_4723_Mech-menapis-occasiva_Col-WEST
Sal_5681_Mech-menapis-occasiva_Col-WEST
Sal_5683_Mech-menapis-occasiva_Col-WEST
Sal_2064_Mech-menapis-caribensis_Col-WEST
Sal_2066_Mech-menapis-caribensis_Col-WEST
Sal_5638_Mech-menapis-menapis_Col-WEST
Sal_5640_Mech-menapis-caribensis_Col-WEST
Sal_5641_Mech-menapis-caribensis_Col-WEST
Sal_3342_Mech-menapis-dariensis_Col-WEST
CAM070759_Mech-menapis-mantineus_Ec-WEST
SAN25001771_Mech-menapis-mantineus_Ec-WEST
SAN25001770_Mech-menapis-mantineus_Ec-WEST
SAN25001773_Mech-menapis-mantineus_Ec-WEST
SAN25001772_Mech-menapis-mantineus_Ec-WEST
Sal_1532_Mech-messenoides-deceptus_Col-WA
Sal_1360_Mech-messenoides-deceptus_Col-WA
Mon_2012_198_Mech-messenoides-deceptus_Ec-WA
Mon_2012_146_Mech-messenoides-deceptus_Ec-WA
Mon_2012_242_Mech-messenoides-messenoides_Ec-WA
Mon_2011_124_Mech-messenoides-deceptus_Ec-WA
Mon_2012_99_Mech-messenoides-messenoides_Ec-WA
Mon_2012_220_Mech-messenoides-messenoides_Ec-WA
Mon_2011_195_Mech-messenoides-deceptus_Ec-WA
Mon_2012_243_Mech-messenoides-deceptus_Ec-WA
Mon_2012_90_Mech-messenoides-messenoides_Ec-WA
55.02.1284_Mech-messenoides-phasianita_Peru-WA
CAM077084_Mech-messenoides-messenoides_Col-WA
61.02.1896_Mech-messenoides-phasianita_Peru-WA
CAM077080_Mech-messenoides-deceptus_Col-WA
60.02.1290_Mech-messenoides-phasianita_Peru-WA
CAM077079_Mech-messenoides-deceptus_Col-WA
Mon_2012_244_Mech-messenoides-messenoides_Ec-WA
CAM077081_Mech-messenoides-messenoides_Col-WA
CAM077091_Mech-messenoides-messenoides_Col-WA
CAM077045_Mech-messenoides-messenoides_Col-WA
CAM077092_Mech-messenoides-messenoides_Col-WA
CAM077083_Mech-messenoides-messenoides_Col-WA
Mon_2012_219_Mech-messenoides-mess-dec_Ec-WA
65.02.477_Mech-messenoides-phasianita_Peru-WA
Mon_2012_88_Mech-messenoides-deceptus_Ec-WA
50.02.348_Mech-messenoides-phasianita_Peru-WA
68.02.677_Mech-messenoides-phasianita_Peru-WA
CAM028090_For-equicola-equicoloides_Ec-WA
CAM028271_For-equicola-equicoloides_Ec-WA
CAM028327_For-equicola-equicoloides_Ec-WA
CAM028193_For-olivencia-juntana_Ec-WA
CAM070582_For-olivencia-juntana_Ec-WA
CAM028312_For-olivencia-juntana_Ec-WA
CAM070479_For-proceris_Ec-WA
CAM070480_For-proceris_Ec-WA
CAM070483_For-proceris_Ec-WA

Fig. S1. Nuclear phylogeny for *Mechanitis*

Each individual is labelled with ‘ID_Species_Country-Region’. The countries are: Pan = Panama; Ec = Ecuador; Col = Colombia; Peru = Peru; FG = French Guiana; Braz = Brazil; Sur = Suriname. The regions are: WEST = west of the Andes; WA = Western Amazonia; GUI = Guianan Shield; AF = Atlantic Forest. Numbers at the node indicate bootstrap support.

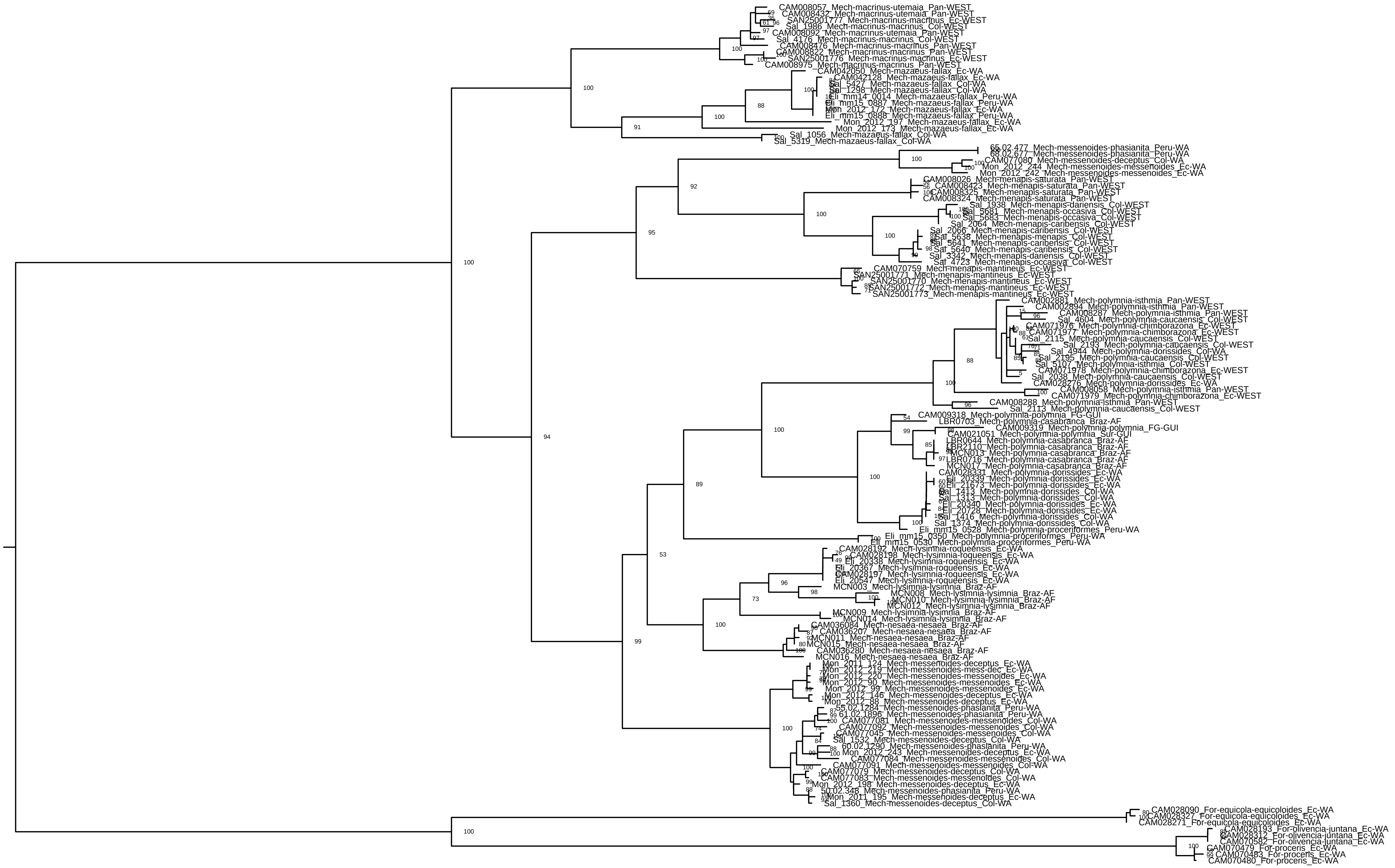
CAM008057_Mech-macrinus-utemaia_Pan-WEST
CAM008432_Mech-macrinus-utemaia_Pan-WEST
SAN25001777_Mech-macrinus-macrinus_Ec-WEST
Sal_1986_Mech-macrinus-macrinus_Col-WEST
CAM008092_Mech-macrinus-utemaia_Pan-WEST
Sal_4176_Mech-macrinus-macrinus_Col-WEST
CAM008476_Mech-macrinus-macrinus_Pan-WEST
CAM008822_Mech-macrinus-macrinus_Pan-WEST
SAN25001776_Mech-macrinus-macrinus_Ec-WEST
CAM008975_Mech-macrinus-macrinus_Pan-WEST
CAM042050_Mech-mazaeus-fallax_Ec-WA
CAM042128_Mech-mazaeus-fallax_Ec-WA
Sal_5427_Mech-mazaeus-fallax_Col-WA
Sal_1298_Mech-mazaeus-fallax_Col-WA
Eli_mm14_0014_Mech-mazaeus-fallax_Peru-WA
Eli_mm15_0887_Mech-mazaeus-fallax_Peru-WA
Mon_2012_172_Mech-mazaeus-fallax_Ec-WA
Eli_mm15_0888_Mech-mazaeus-fallax_Peru-WA
Mon_2012_197_Mech-mazaeus-fallax_Ec-WA
Mon_2012_173_Mech-mazaeus-fallax_Ec-WA
Sal_1056_Mech-mazaeus-fallax_Col-WA
Sal_5319_Mech-mazaeus-fallax_Col-WA
65.02.477_Mech-messenoides-phasianita_Peru-WA
68.02.677_Mech-messenoides-phasianita_Peru-WA
CAM077080_Mech-messenoides-deceptus_Col-WA
Mon_2012_244_Mech-messenoides-messenoides_Ec-WA
Mon_2012_242_Mech-messenoides-messenoides_Ec-WA
CAM008026_Mech-menapis-saturata_Pan-WEST
CAM008423_Mech-menapis-saturata_Pan-WEST
CAM008325_Mech-menapis-saturata_Pan-WEST
CAM008324_Mech-menapis-saturata_Pan-WEST
Sal_1938_Mech-menapis-dariensis_Col-WEST
Sal_5681_Mech-menapis-occasiva_Col-WEST
Sal_5683_Mech-menapis-occasiva_Col-WEST
Sal_2064_Mech-menapis-caribensis_Col-WEST
Sal_2066_Mech-menapis-caribensis_Col-WEST
Sal_5638_Mech-menapis-menapis_Col-WEST
Sal_5641_Mech-menapis-caribensis_Col-WEST
Sal_5640_Mech-menapis-caribensis_Col-WEST
Sal_3342_Mech-menapis-dariensis_Col-WEST
Sal_4723_Mech-menapis-occasiva_Col-WEST
CAM070759_Mech-menapis-mantineus_Ec-WEST
SAN25001771_Mech-menapis-mantineus_Ec-WEST
SAN25001770_Mech-menapis-mantineus_Ec-WEST
SAN25001772_Mech-menapis-mantineus_Ec-WEST
SAN25001773_Mech-menapis-mantineus_Ec-WEST
CAM002881_Mech-polymnia-isthmia_Pan-WEST
CAM002894_Mech-polymnia-isthmia_Pan-WEST
CAM008287_Mech-polymnia-isthmia_Pan-WEST
Sal_4604_Mech-polymnia-caucaensis_Col-WEST
CAM071976_Mech-polymnia-chimborazona_Ec-WEST
CAM071977_Mech-polymnia-chimborazona_Ec-WEST
Sal_2115_Mech-polymnia-caucaensis_Col-WEST
Sal_2193_Mech-polymnia-caucaensis_Col-WEST
Sal_4944_Mech-polymnia-dorissides_Col-WA
Sal_2195_Mech-polymnia-caucaensis_Col-WEST
Sal_5107_Mech-polymnia-isthmia_Col-WEST
CAM071978_Mech-polymnia-chimborazona_Ec-WEST
Sal_2038_Mech-polymnia-caucaensis_Col-WEST
CAM028276_Mech-polymnia-dorissides_Ec-WA
CAM008058_Mech-polymnia-isthmia_Pan-WEST
CAM071979_Mech-polymnia-chimborazona_Ec-WEST
CAM008288_Mech-polymnia-isthmia_Pan-WEST
Sal_2113_Mech-polymnia-caucaensis_Col-WEST
CAM009318_Mech-polymnia-polymnia_FG-GUI
LBR0703_Mech-polymnia-casabranca_Braz-AF
CAM009319_Mech-polymnia-polymnia_FG-GUI
CAM021051_Mech-polymnia-polymnia_Sur-GUI
LBR0644_Mech-polymnia-casabranca_Braz-AF
LBR2110_Mech-polymnia-casabranca_Braz-AF
MCN013_Mech-polymnia-casabranca_Braz-AF
LBR0716_Mech-polymnia-casabranca_Braz-AF
MCN017_Mech-polymnia-casabranca_Braz-AF
CAM028331_Mech-polymnia-dorissides_Ec-WA
Eli_20339_Mech-polymnia-dorissides_Ec-WA
Eli_21673_Mech-polymnia-dorissides_Ec-WA
Sal_1413_Mech-polymnia-dorissides_Col-WA
Sal_1313_Mech-polymnia-dorissides_Col-WA
Eli_20340_Mech-polymnia-dorissides_Ec-WA
Eli_20728_Mech-polymnia-dorissides_Ec-WA
Sal_1416_Mech-polymnia-dorissides_Col-WA
Sal_1374_Mech-polymnia-dorissides_Col-WA
Eli_mm15_0528_Mech-polymnia-proceriformes_Peru-WA
Eli_mm15_0350_Mech-polymnia-proceriformes_Peru-WA
Eli_mm15_0530_Mech-polymnia-proceriformes_Peru-WA
CAM028192_Mech-lysimnia-roqueensis_Ec-WA
CAM028198_Mech-lysimnia-roqueensis_Ec-WA
Eli_20338_Mech-lysimnia-roqueensis_Ec-WA
Eli_20367_Mech-lysimnia-roqueensis_Ec-WA
CAM028197_Mech-lysimnia-roqueensis_Ec-WA
Eli_20547_Mech-lysimnia-roqueensis_Ec-WA
MCN003_Mech-lysimnia-lysimnia_Braz-AF
MCN008_Mech-lysimnia-lysimnia_Braz-AF
MCN010_Mech-lysimnia-lysimnia_Braz-AF
MCN012_Mech-lysimnia-lysimnia_Braz-AF
MCN009_Mech-lysimnia-lysimnia_Braz-AF
MCN014_Mech-lysimnia-lysimnia_Braz-AF
CAM036084_Mech-nesaea-nesaea_Braz-AF
CAM036207_Mech-nesaea-nesaea_Braz-AF
MCN011_Mech-nesaea-nesaea_Braz-AF
MCN015_Mech-nesaea-nesaea_Braz-AF
CAM036280_Mech-nesaea-nesaea_Braz-AF
MCN016_Mech-nesaea-nesaea_Braz-AF
Mon_2011_124_Mech-messenoides-deceptus_Ec-WA
Mon_2012_219_Mech-messenoides-mess-dec_Ec-WA
Mon_2012_220_Mech-messenoides-messenoides_Ec-WA
Mon_2012_90_Mech-messenoides-messenoides_Ec-WA
Mon_2012_99_Mech-messenoides-messenoides_Ec-WA
Mon_2012_146_Mech-messenoides-deceptus_Ec-WA
Mon_2012_88_Mech-messenoides-deceptus_Ec-WA
55.02.1284_Mech-messenoides-phasianita_Peru-WA
61.02.1896_Mech-messenoides-phasianita_Peru-WA
CAM077081_Mech-messenoides-messenoides_Col-WA
CAM077092_Mech-messenoides-messenoides_Col-WA
CAM077045_Mech-messenoides-messenoides_Col-WA
Sal_1532_Mech-messenoides-deceptus_Col-WA
60.02.1290_Mech-messenoides-phasianita_Peru-WA
Mon_2012_243_Mech-messenoides-deceptus_Ec-WA
CAM077084_Mech-messenoides-messenoides_Col-WA
CAM077091_Mech-messenoides-messenoides_Col-WA
CAM077079_Mech-messenoides-deceptus_Col-WA
CAM077083_Mech-messenoides-messenoides_Col-WA
Mon_2012_198_Mech-messenoides-deceptus_Ec-WA
50.02.348_Mech-messenoides-phasianita_Peru-WA
Mon_2011_195_Mech-messenoides-deceptus_Ec-WA
Sal_1360_Mech-messenoides-deceptus_Col-WA
CAM028090_For-equicola-equicoloides_Ec-WA
CAM028327_For-equicola-equicoloides_Ec-WA
CAM028271_For-equicola-equicoloides_Ec-WA
CAM028193_For-olivencia-juntana_Ec-WA
CAM028312_For-olivencia-juntana_Ec-WA
CAM070582_For-olivencia-juntana_Ec-WA
CAM070479_For-proceris_Ec-WA
CAM070483_For-proceris_Ec-WA
CAM070480_For-proceris_Ec-WA

Fig. S2. Mitochondrial phylogeny for *Mechanitis*

Each individual is labelled with 'ID_Species_Country-Region'. The countries are: Pan = Panama; Ec = Ecuador; Col = Colombia; Peru = Peru; FG = French Guiana; Braz = Brazil; Sur = Suriname. The regions are: WEST = west of the Andes; WA = Western Amazonia; GUI = Guianan Shield; AF = Atlantic Forest. Numbers at the node indicate bootstrap support.

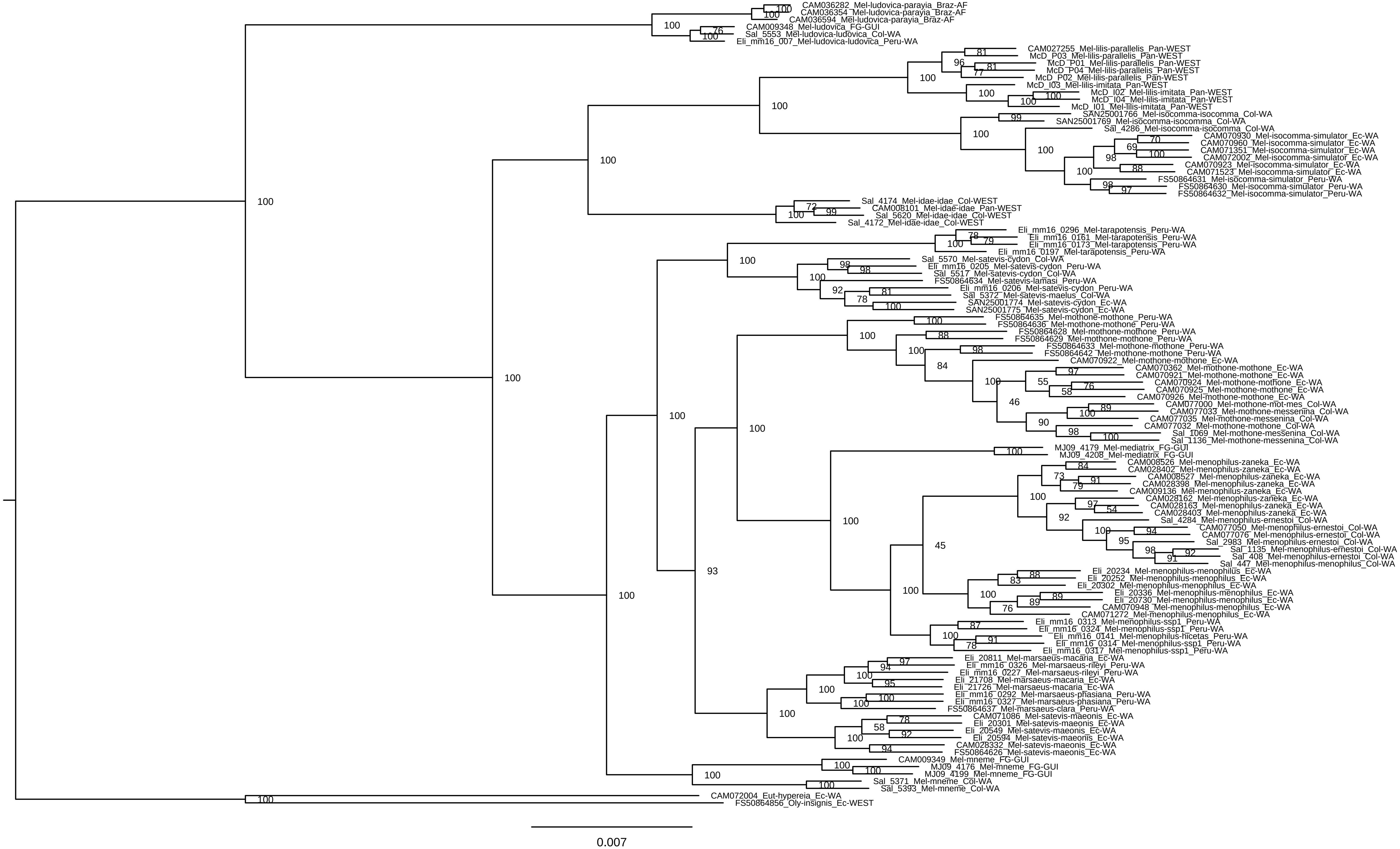
CAM036282_Mel-ludovica-parayia_Braz-AF
CAM036354_Mel-ludovica-parayia_Braz-AF
CAM036594_Mel-ludovica-parayia_Braz-AF
CAM009348_Mel-ludovica_FG-GUI
Sal_5553_Mel-ludovica-ludovica_Col-WA
Eli_mm16_007_Mel-ludovica-ludovica_Peru-WA
CAM027255_Mel-lilis-parallelis_Pan-WEST
McD_P03_Mel-lilis-parallelis_Pan-WEST
McD_P01_Mel-lilis-parallelis_Pan-WEST
McD_P04_Mel-lilis-parallelis_Pan-WEST
McD_P02_Mel-lilis-parallelis_Pan-WEST
McD_I03_Mel-lilis-imitata_Pan-WEST
McD_I02_Mel-lilis-imitata_Pan-WEST
McD_I04_Mel-lilis-imitata_Pan-WEST
McD_I01_Mel-lilis-imitata_Pan-WEST
SAN25001766_Mel-isocomma-isocomma_Col-WA
SAN25001769_Mel-isocomma-isocomma_Col-WA
Sal_4286_Mel-isocomma-isocomma_Col-WA
CAM070930_Mel-isocomma-simulator_Ec-WA
CAM070960_Mel-isocomma-simulator_Ec-WA
CAM071351_Mel-isocomma-simulator_Ec-WA
CAM072002_Mel-isocomma-simulator_Ec-WA
CAM070923_Mel-isocomma-simulator_Ec-WA
CAM071523_Mel-isocomma-simulator_Ec-WA
FS50864631_Mel-isocomma-simulator_Peru-WA
FS50864630_Mel-isocomma-simulator_Peru-WA
FS50864632_Mel-isocomma-simulator_Peru-WA
Sal_4174_Mel-idae-idae_Col-WEST
CAM008101_Mel-idae-idae_Pan-WEST
Sal_5620_Mel-idae-idae_Col-WEST
Sal_4172_Mel-idae-idae_Col-WEST
Eli_mm16_0296_Mel-tarapotensis_Peru-WA
Eli_mm16_0161_Mel-tarapotensis_Peru-WA
Eli_mm16_0173_Mel-tarapotensis_Peru-WA
Eli_mm16_0197_Mel-tarapotensis_Peru-WA
Sal_5570_Mel-satevis-cydon_Col-WA
Eli_mm16_0205_Mel-satevis-cydon_Peru-WA
Sal_5517_Mel-satevis-cydon_Col-WA
FS50864634_Mel-satevis-lamasi_Peru-WA
Eli_mm16_0206_Mel-satevis-cydon_Peru-WA
Sal_5372_Mel-satevis-maelus_Col-WA
SAN25001774_Mel-satevis-cydon_Ec-WA
SAN25001775_Mel-satevis-cydon_Ec-WA
FS50864635_Mel-mothone-mothone_Peru-WA
FS50864636_Mel-mothone-mothone_Peru-WA
FS50864628_Mel-mothone-mothone_Peru-WA
FS50864629_Mel-mothone-mothone_Peru-WA
FS50864633_Mel-mothone-mothone_Peru-WA
FS50864642_Mel-mothone-mothone_Peru-WA
CAM070922_Mel-mothone-mothone_Ec-WA
CAM070362_Mel-mothone-mothone_Ec-WA
CAM070921_Mel-mothone-mothone_Ec-WA
CAM070924_Mel-mothone-mothone_Ec-WA
CAM070925_Mel-mothone-mothone_Ec-WA
CAM070926_Mel-mothone-mothone_Ec-WA
CAM077000_Mel-mothone-mot-mes_Col-WA
CAM077033_Mel-mothone-messenina_Col-WA
CAM077035_Mel-mothone-messenina_Col-WA
CAM077032_Mel-mothone-mothone_Col-WA
Sal_1069_Mel-mothone-messenina_Col-WA
Sal_1136_Mel-mothone-messenina_Col-WA
MJ09_4179_Mel-mediatrix_FG-GUI
MJ09_4208_Mel-mediatrix_FG-GUI
CAM008526_Mel-menophilus-zaneka_Ec-WA
CAM028402_Mel-menophilus-zaneka_Ec-WA
CAM008527_Mel-menophilus-zaneka_Ec-WA
CAM028398_Mel-menophilus-zaneka_Ec-WA
CAM009136_Mel-menophilus-zaneka_Ec-WA
CAM028162_Mel-menophilus-zaneka_Ec-WA
CAM028163_Mel-menophilus-zaneka_Ec-WA
CAM028403_Mel-menophilus-zaneka_Ec-WA
Sal_4284_Mel-menophilus-ernestoi_Col-WA
CAM077050_Mel-menophilus-ernestoi_Col-WA
CAM077076_Mel-menophilus-ernestoi_Col-WA
Sal_2983_Mel-menophilus-ernestoi_Col-WA
Sal_1135_Mel-menophilus-ernestoi_Col-WA
Sal_408_Mel-menophilus-ernestoi_Col-WA
Sal_447_Mel-menophilus-ernestoi_Col-WA
Eli_20234_Mel-menophilus-menophilus_Ec-WA
Eli_20252_Mel-menophilus-menophilus_Ec-WA
Eli_20302_Mel-menophilus-menophilus_Ec-WA
Eli_20336_Mel-menophilus-menophilus_Ec-WA
Eli_20730_Mel-menophilus-menophilus_Ec-WA
CAM070948_Mel-menophilus-menophilus_Ec-WA
CAM071272_Mel-menophilus-menophilus_Ec-WA
Eli_mm16_0313_Mel-menophilus-ssp1_Peru-WA
Eli_mm16_0324_Mel-menophilus-ssp1_Peru-WA
Eli_mm16_0141_Mel-menophilus-hicetas_Peru-WA
Eli_mm16_0314_Mel-menophilus-ssp1_Peru-WA
Eli_mm16_0317_Mel-menophilus-ssp1_Peru-WA
Eli_20811_Mel-marsaeus-macaria_Ec-WA
Eli_mm16_0326_Mel-marsaeus-rileyi_Peru-WA
Eli_mm16_0227_Mel-marsaeus-rileyi_Peru-WA
Eli_21708_Mel-marsaeus-macaria_Ec-WA
Eli_21726_Mel-marsaeus-macaria_Ec-WA
Eli_mm16_0292_Mel-marsaeus-phasiana_Peru-WA
Eli_mm16_0327_Mel-marsaeus-phasiana_Peru-WA
FS50864637_Mel-marsaeus-clara_Peru-WA
CAM071086_Mel-satevis-maeonis_Ec-WA
Eli_20301_Mel-satevis-maeonis_Ec-WA
Eli_20549_Mel-satevis-maeonis_Ec-WA
Eli_20594_Mel-satevis-maeonis_Ec-WA
CAM028332_Mel-satevis-maeonis_Ec-WA
FS50864626_Mel-satevis-maeonis_Ec-WA
CAM009349_Mel-mneme_FG-GUI
MJ09_4176_Mel-mneme_FG-GUI
MJ09_4199_Mel-mneme_FG-GUI
Sal_5371_Mel-mneme_Col-WA
Sal_5393_Mel-mneme_Col-WA
CAM072004_Eut-hypereia_Ec-WA
FS50864856_Oly-insignis_Ec-WEST
0.007

Fig. S3. Nuclear phylogeny for *Melinaea*

Each individual is labelled with 'ID_Species_Country-Region'. The countries are: Pan = Panama; Ec = Ecuador; Col = Colombia; Peru = Peru; FG = French Guiana; Braz = Brazil; Sur = Suriname. The regions are: WEST = west of the Andes; WA = Western Amazonia; GUI = Guianan Shield; AF = Atlantic Forest. Numbers at the node indicate bootstrap support.

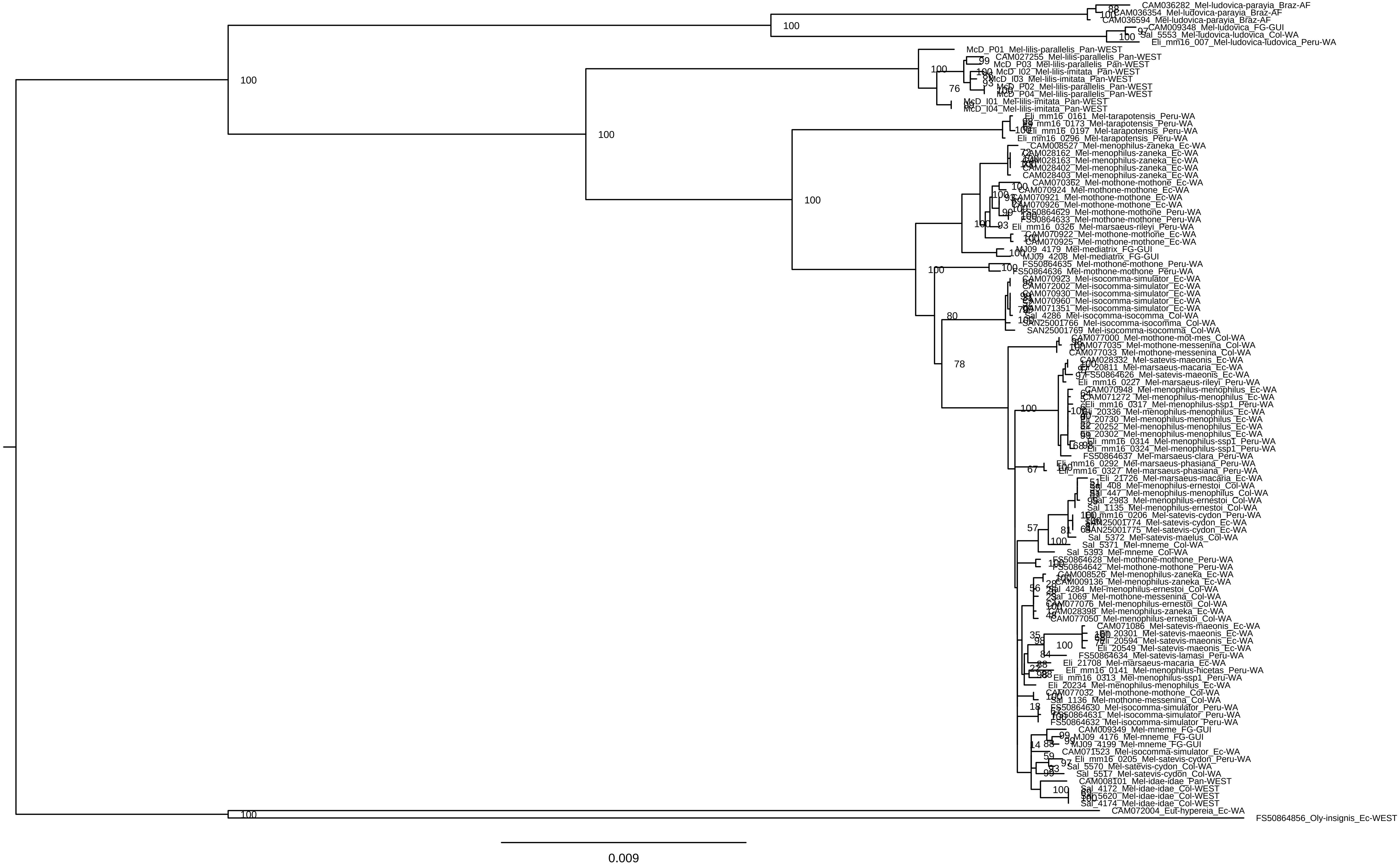
CAM036282_Mel-ludovica-parayia_Braz-AF
CAM036354_Mel-ludovica-parayia_Braz-AF
CAM036594_Mel-ludovica-parayia_Braz-AF
CAM009348_Mel-ludovica_FG-GUI
Sal_5553_Mel-ludovica-ludovica_Col-WA
Eli_mm16_007_Mel-ludovica-ludovica_Peru-WA
McD_P01_Mel-lilis-parallelis_Pan-WEST
CAM027255_Mel-lilis-parallelis_Pan-WEST
McD_P03_Mel-lilis-parallelis_Pan-WEST
McD_I02_Mel-lilis-imitata_Pan-WEST
McD_I03_Mel-lilis-imitata_Pan-WEST
McD_P02_Mel-lilis-parallelis_Pan-WEST
McD_P04_Mel-lilis-parallelis_Pan-WEST
McD_I01_Mel-lilis-imitata_Pan-WEST
McD_I04_Mel-lilis-imitata_Pan-WEST
Eli_mm16_0161_Mel-tarapotensis_Peru-WA
Eli_mm16_0173_Mel-tarapotensis_Peru-WA
Eli_mm16_0197_Mel-tarapotensis_Peru-WA
Eli_mm16_0296_Mel-tarapotensis_Peru-WA
CAM008527_Mel-menophilus-zaneka_Ec-WA
CAM028162_Mel-menophilus-zaneka_Ec-WA
CAM028163_Mel-menophilus-zaneka_Ec-WA
CAM028402_Mel-menophilus-zaneka_Ec-WA
CAM028403_Mel-menophilus-zaneka_Ec-WA
CAM070362_Mel-mothone-mothone_Ec-WA
CAM070924_Mel-mothone-mothone_Ec-WA
CAM070921_Mel-mothone-mothone_Ec-WA
CAM070926_Mel-mothone-mothone_Ec-WA
FS50864629_Mel-mothone-mothone_Peru-WA
FS50864633_Mel-mothone-mothone_Peru-WA
Eli_mm16_0326_Mel-marsaeus-rileyi_Peru-WA
CAM070922_Mel-mothone-mothone_Ec-WA
CAM070925_Mel-mothone-mothone_Ec-WA
MJ09_4179_Mel-mediatrix_FG-GUI
MJ09_4208_Mel-mediatrix_FG-GUI
FS50864635_Mel-mothone-mothone_Peru-WA
FS50864636_Mel-mothone-mothone_Peru-WA
CAM070923_Mel-isocomma-simulator_Ec-WA
CAM072002_Mel-isocomma-simulator_Ec-WA
CAM070930_Mel-isocomma-simulator_Ec-WA
CAM070960_Mel-isocomma-simulator_Ec-WA
CAM071351_Mel-isocomma-simulator_Ec-WA
Sal_4286_Mel-isocomma-isocomma_Col-WA
SAN25001766_Mel-isocomma-isocomma_Col-WA
SAN25001769_Mel-isocomma-isocomma_Col-WA
CAM077000_Mel-mothone-mot-mes_Col-WA
CAM077035_Mel-mothone-messenina_Col-WA
CAM077033_Mel-mothone-messenina_Col-WA
CAM028332_Mel-satevis-maeonis_Ec-WA
Eli_20811_Mel-marsaeus-macaria_Ec-WA
FS50864626_Mel-satevis-maeonis_Peru-WA
Eli_mm16_0227_Mel-marsaeus-rileyi_Peru-WA
CAM070948_Mel-menophilus-menophilus_Ec-WA
CAM071272_Mel-menophilus-menophilus_Ec-WA
Eli_mm16_0317_Mel-menophilus-ssp1_Peru-WA
Eli_20336_Mel-menophilus-menophilus_Ec-WA
Eli_20730_Mel-menophilus-menophilus_Ec-WA
Eli_20252_Mel-menophilus-menophilus_Ec-WA
Eli_20302_Mel-menophilus-menophilus_Ec-WA
Eli_mm16_0314_Mel-menophilus-ssp1_Peru-WA
Eli_mm16_0324_Mel-menophilus-ssp1_Peru-WA
FS50864637_Mel-marsaeus-clara_Peru-WA
Eli_mm16_0292_Mel-marsaeus-phasiana_Peru-WA
Eli_mm16_0327_Mel-marsaeus-phasiana_Peru-WA
Eli_21726_Mel-marsaeus-macaria_Ec-WA
Sal_408_Mel-menophilus-ernestoi_Col-WA
Sal_447_Mel-menophilus-menophilus_Col-WA
Sal_2983_Mel-menophilus-ernestoi_Col-WA
Sal_1135_Mel-menophilus-ernestoi_Col-WA
Eli_mm16_0206_Mel-satevis-cydon_Peru-WA
SAN25001774_Mel-satevis-cydon_Ec-WA
SAN25001775_Mel-satevis-cydon_Ec-WA
Sal_5372_Mel-satevis-maelus_Col-WA
Sal_5371_Mel-mneme_Col-WA
Sal_5393_Mel-mneme_Col-WA
FS50864628_Mel-mothone-mothone_Peru-WA
FS50864642_Mel-mothone-mothone_Peru-WA
CAM008526_Mel-menophilus-zaneka_Ec-WA
CAM009136_Mel-menophilus-zaneka_Ec-WA
Sal_4284_Mel-menophilus-ernestoi_Col-WA
Sal_1069_Mel-mothone-messenina_Col-WA
CAM077076_Mel-menophilus-ernestoi_Col-WA
CAM028398_Mel-menophilus-zaneka_Ec-WA
CAM077050_Mel-menophilus-ernestoi_Col-WA
CAM071086_Mel-satevis-maeonis_Ec-WA
Eli_20301_Mel-satevis-maeonis_Ec-WA
Eli_20594_Mel-satevis-maeonis_Ec-WA
Eli_20549_Mel-satevis-maeonis_Ec-WA
FS50864634_Mel-satevis-lamasi_Peru-WA
Eli_21708_Mel-marsaeus-macaria_Ec-WA
Eli_mm16_0141_Mel-menophilus-hicetas_Peru-WA
Eli_mm16_0313_Mel-menophilus-ssp1_Peru-WA
Eli_20234_Mel-menophilus-menophilus_Ec-WA
CAM077032_Mel-mothone-mothone_Col-WA
Sal_1136_Mel-mothone-messenina_Col-WA
FS50864630_Mel-isocomma-simulator_Peru-WA
FS50864631_Mel-isocomma-simulator_Peru-WA
FS50864632_Mel-isocomma-simulator_Peru-WA
CAM009349_Mel-mneme_FG-GUI
MJ09_4176_Mel-mneme_FG-GUI
MJ09_4199_Mel-mneme_FG-GUI
CAM071523_Mel-isocomma-simulator_Ec-WA
Eli_mm16_0205_Mel-satevis-cydon_Peru-WA
Sal_5570_Mel-satevis-cydon_Col-WA
Sal_5517_Mel-satevis-cydon_Col-WA
CAM008101_Mel-idae-idae_Pan-WEST
Sal_4172_Mel-idae-idae_Col-WEST
Sal_5620_Mel-idae-idae_Col-WEST
Sal_4174_Mel-idae-idae_Col-WEST
CAM072004_Eut-hypereia_Ec-WA
FS50864856_Oly-insignis_Ec-WEST
0.009

Fig. S4. Mitochondrial phylogeny for *Melinaea*

Each individual is labelled with 'ID_Species_Country-Region'. The countries are: Pan = Panama; Ec = Ecuador; Col = Colombia; Peru = Peru; FG = French Guiana; Braz = Brazil; Sur = Suriname. The regions are: WEST = west of the Andes; WA = Western Amazonia; GUI = Guianan Shield; AF = Atlantic Forest. Numbers at the node indicate bootstrap support.

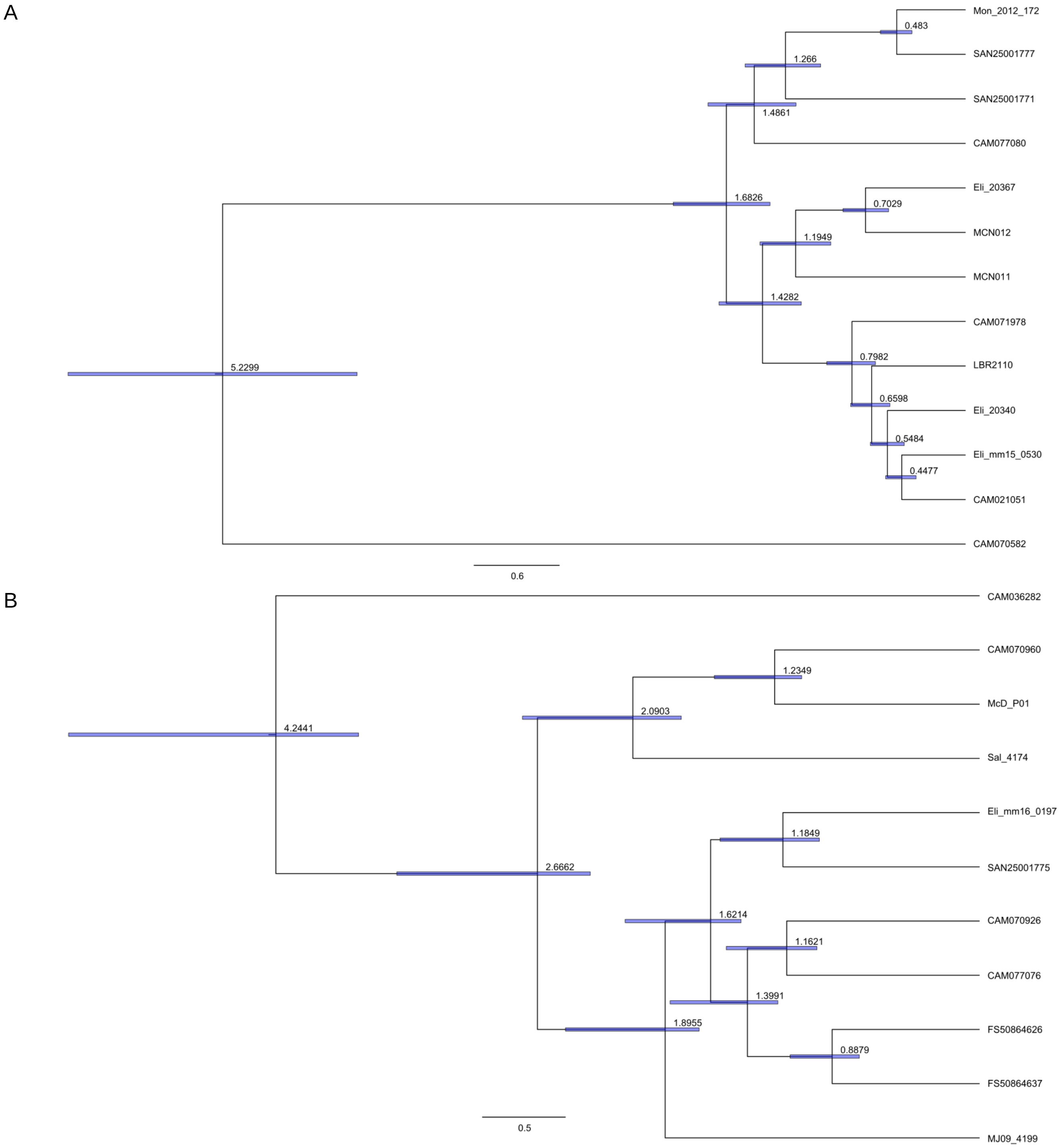

Fig. S5. Calibrated BEAST2 phylogenies

These are the same trees as Figure 2, but calibrated at the divergence time between Mechanitis *and the outgroup* Forbestra (5.22 MYA (5)*) (**A**) and* Mel. ludovica *and the 'core' clade of* Melinaea *(4.66 MYA (5)) (**B**). With these trees, divergence time is longer than with the tree as shown in the main figure: e.g. 1.68 MYA instead of 1.36 MYA for the split between the polymnia-lysimnia-nesaea and menapis-messenoides-macrinus-mazaeus clade (**A**) and 2.67 MYA instead of 1.41 MYA for the split between the lilis-isocomma-idae and the 'ingroup' clade (**B**). The speciation rate for* Mechanitis *would be 1.17 instead of 1.43 as described in the main text, and 0.86 instead of 1.63 for* Melinaea*. The* Melinaea *analysis did not converge properly, and it is thus unclear how trustworthy these results are. The blue bar gives the 95% confidence interval for the branch height. The number at the node gives the estimated divergence time.*

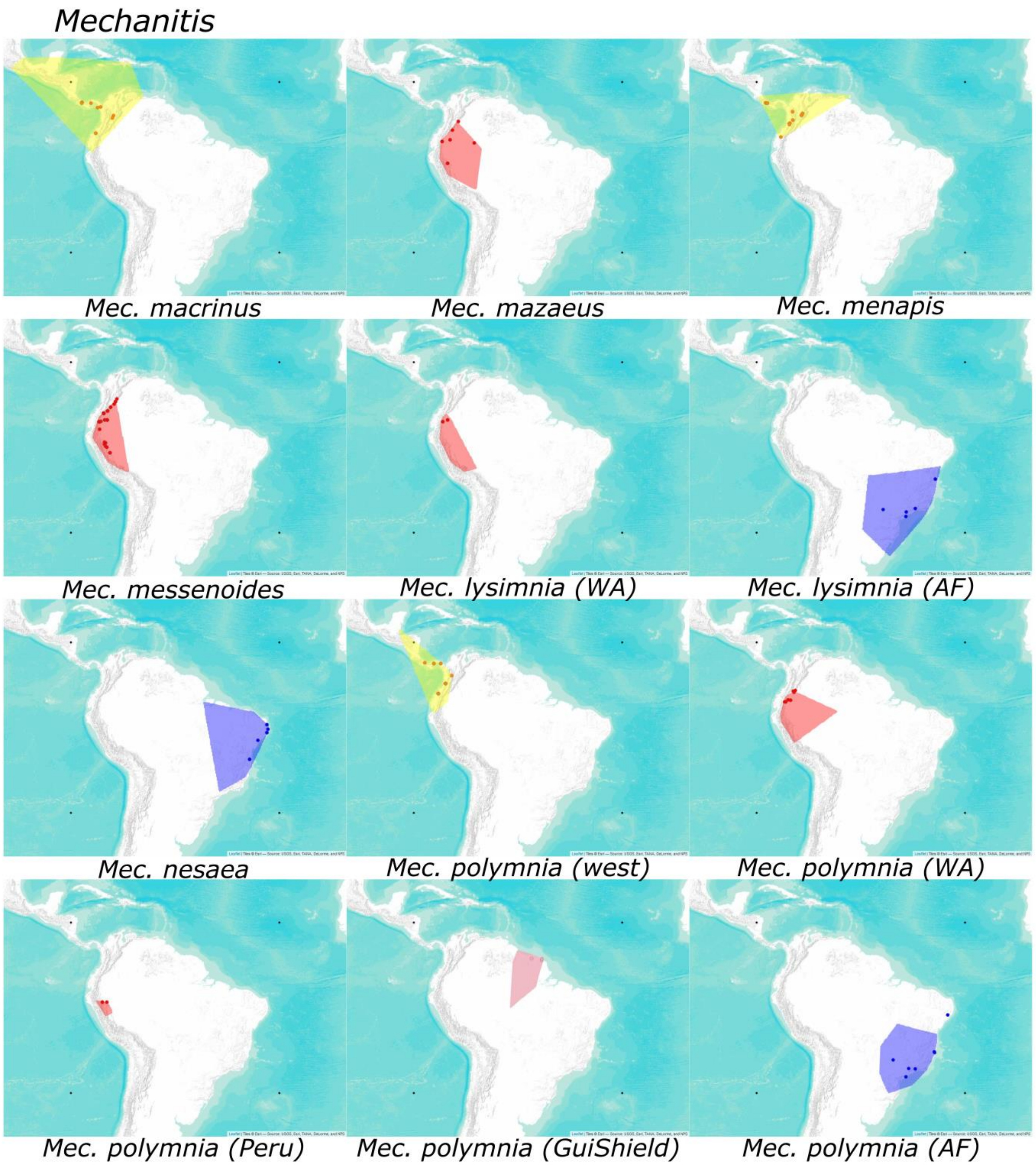

Fig. S6. Distribution maps for *Mechanitis*

Larger sizes of all the distribution maps in main figure 2 for Mechanitis. Concave shapes are based on distribution data from (50)*. Dots are our samples. Attribution for the maps is shown in the corner of each map (downloaded with 'Leaflet', map tiles from USGS, Esri, Tana, DeLorme, and NPS). The four black dots are an aid to crop the maps consistently for the main figure.*

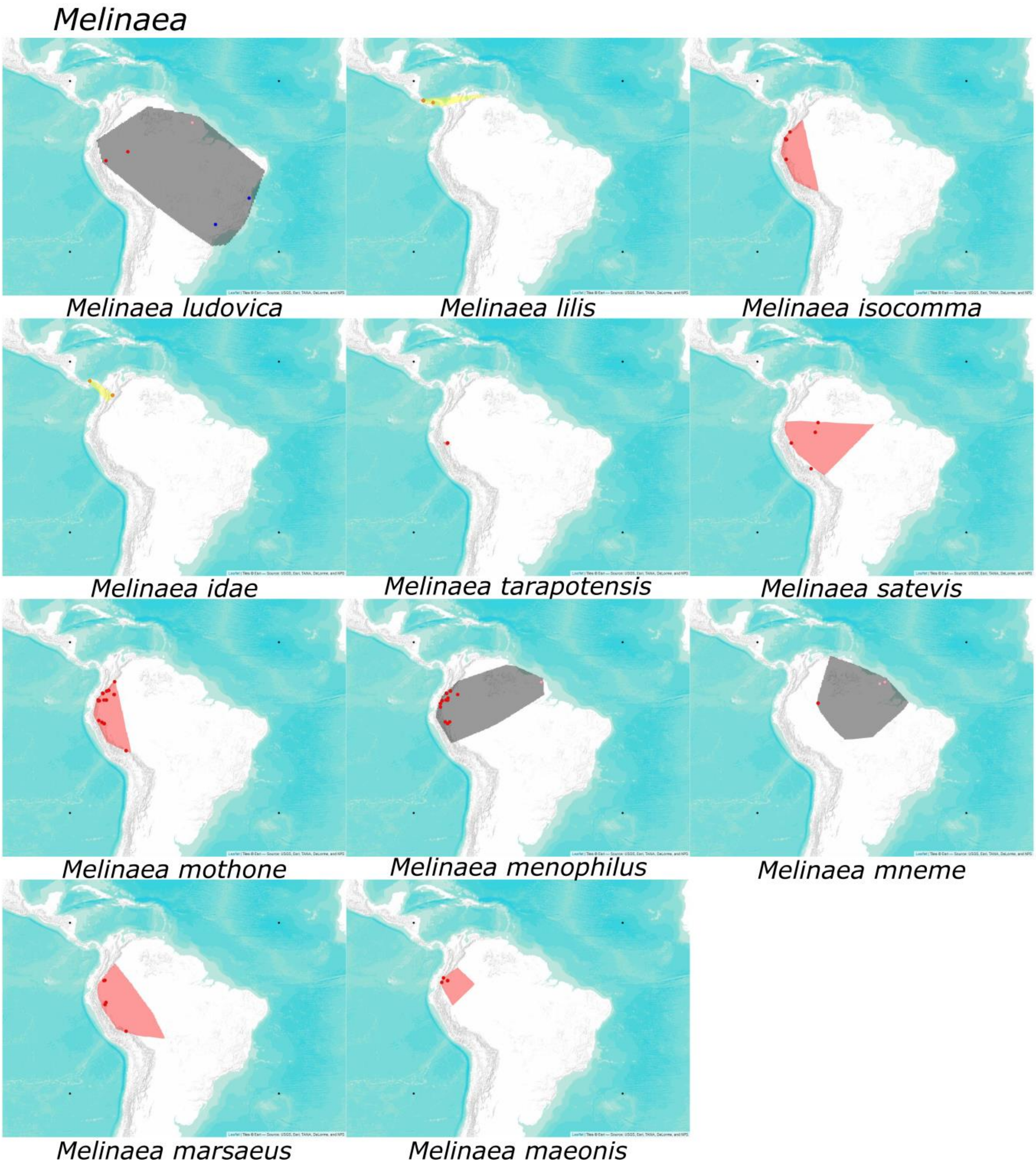

Fig. S7. Distribution maps for *Melinaea*

Larger sizes of all the distribution maps in main figure 2 for Melinaea*. Concave shapes are based on distribution data from* (50)*. Dots are our samples. Attribution for the maps is shown in the corner of each map (downloaded with 'Leaflet', map tiles from USGS, Esri, Tana, DeLorme, and NPS). The four black dots are an aid to crop the maps consistently for the main figure.*

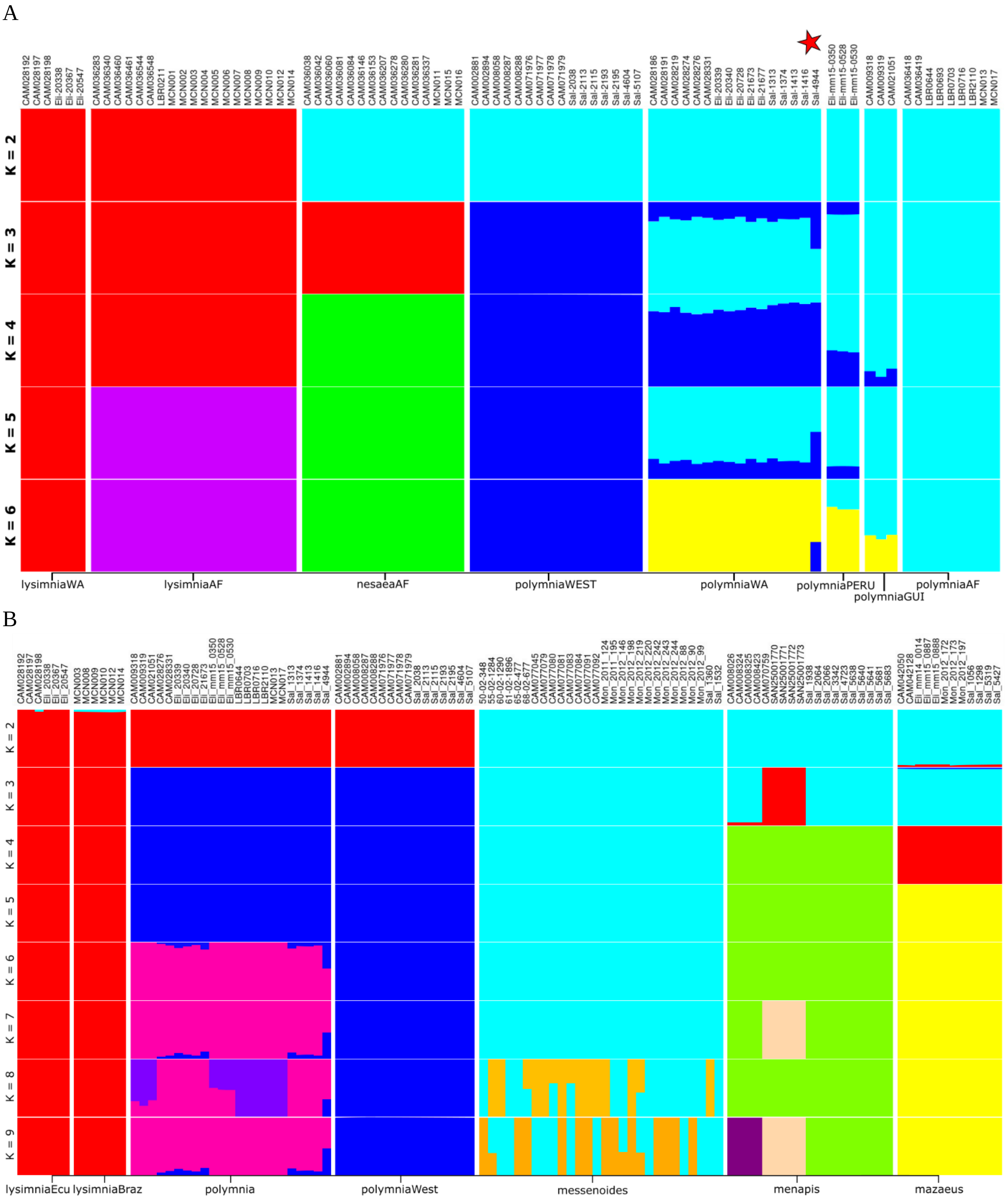
A
K = 2
K = 3
K = 4
K = 5
K = 6
lysimniaWA
lysimniaAF
nesaeaAF
polymniaWEST
polymniaWA
polymniaPERU
polymniaGUI
polymniaAF
B
K = 2
K = 3
K = 4
K = 5
K = 6
K = 7
K = 8
K = 9
lysimniaEcu
lysimniaBraz
polymnia
polymniaWest
messenoides
menapis
mazaeus

C

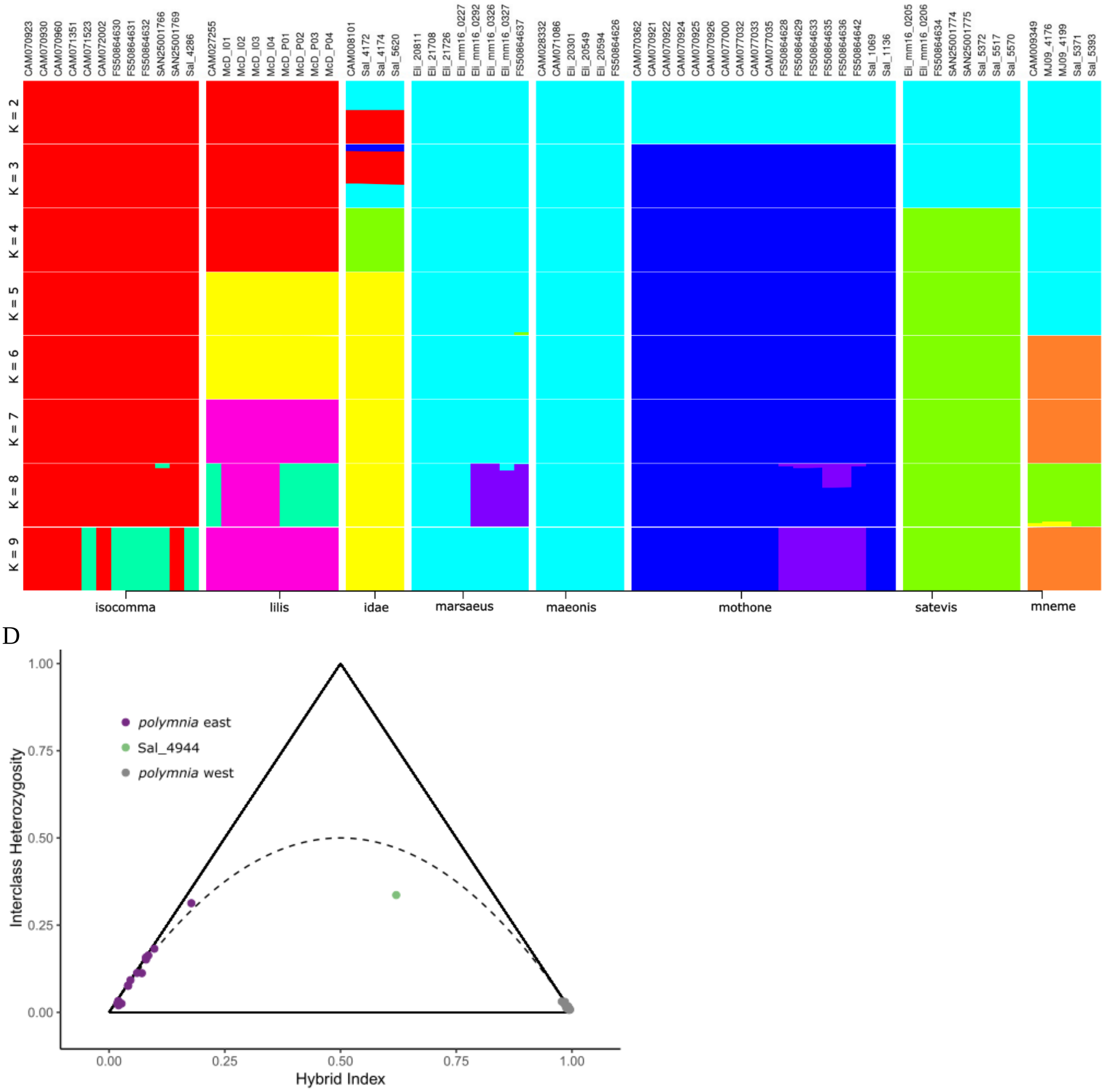

Fig. S8. ADMIXTURE and Triangle plots

A) ADMIXTURE in Mec. polymnia*,* Mec. lysimnia *and* Mec. nesaea*. The red star indicates the Colombian polymnia individual (Sal_4944) caught in Western Amazonia, that appears to have equal ancestry with polymnia Western Amazonia and polymnia West of the Andes. B) ADMIXTURE in* Mec. lysimnia, Mec. polymnia, Mec. messenoides, Mec. menapis, *and* Mec. mazaeus*. C) ADMIXTURE in* Mel. isocomma, Mel. lilis, Mel. idae, Mel. marsaeus, Mel. maeonis, Mel. mothone, Mel. satevis and Mel. mneme. *WA = Western Amazonia; AF = Atlantic Forest; West = west of the Andes; GUI = Guianan Shield; Ecu = Ecuador; Braz = Brazil (same region as Atlantic Forest). D) Triangle plot including all* Mec. polymnia *from Western Amazonia (polymnia east) and from West of the Andes (polymnia west), with the putative hybrid Sal_4944 indicated.*

A. *Mechanitis*

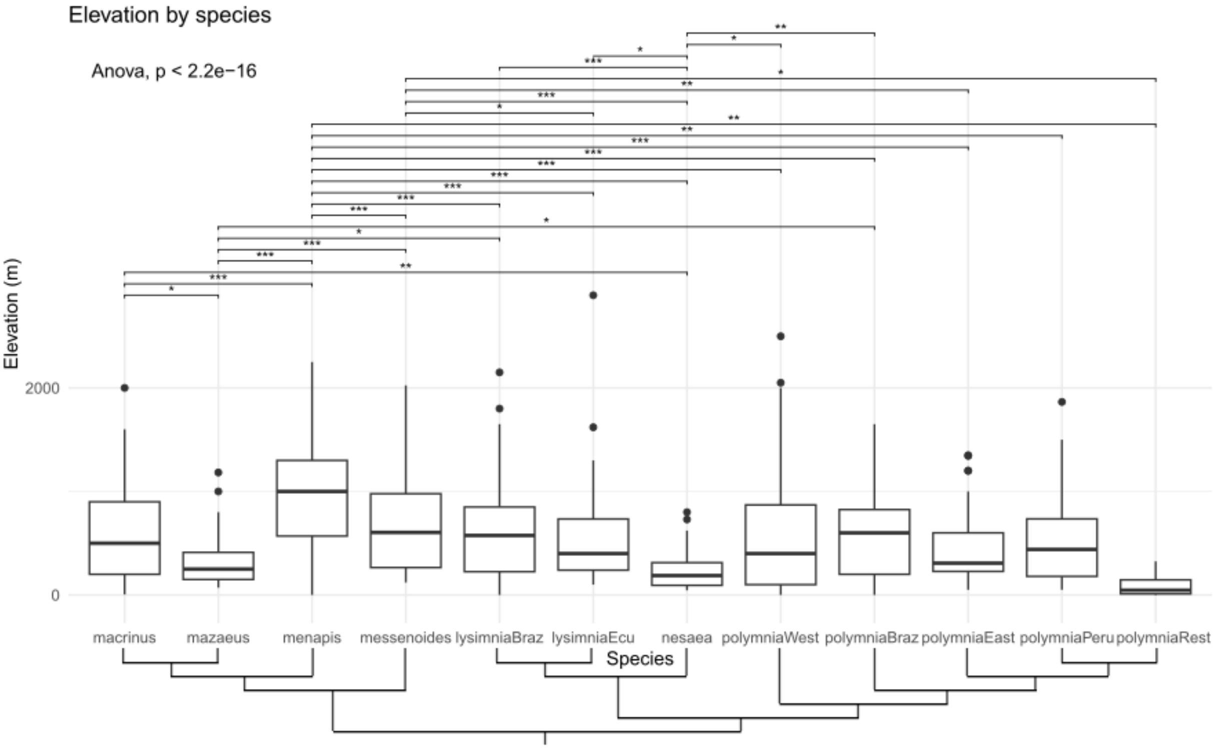

B. *Melinaea*

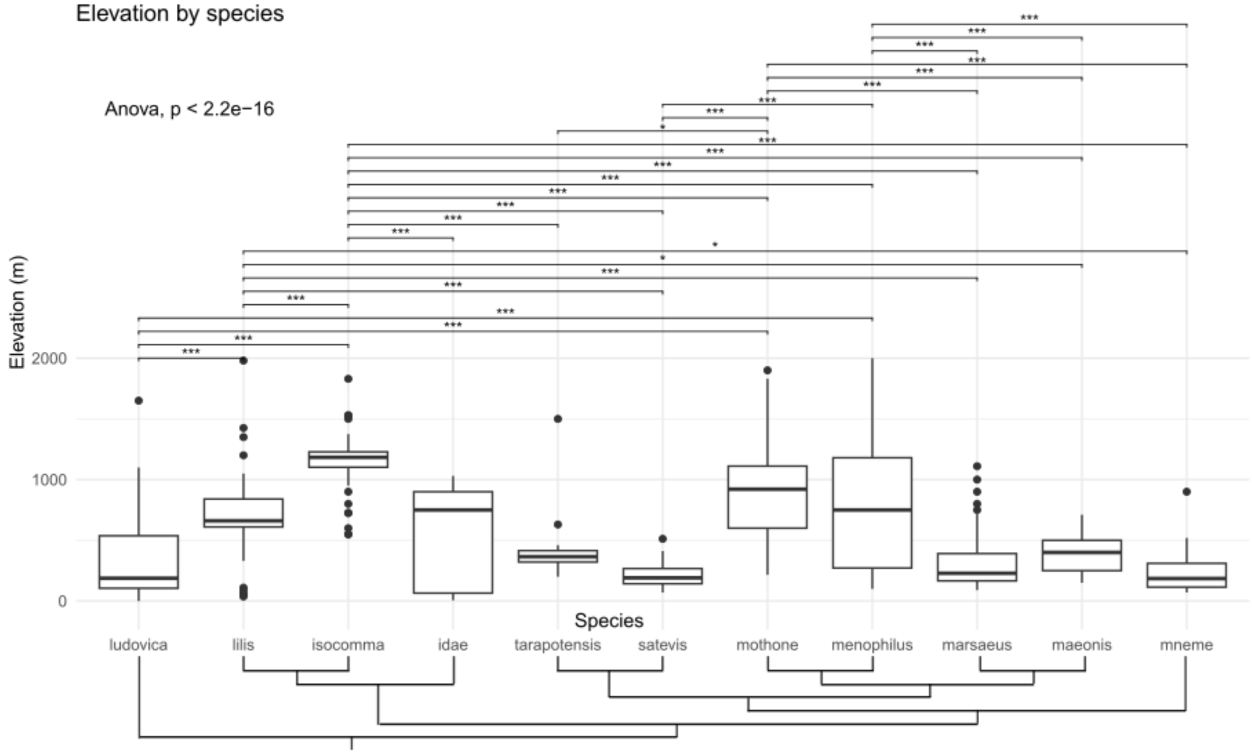

Fig. S9. Boxplots for altitude in *Mechanitis* and *Melinaea*

Boxplots showing the distribution of altitude at which samples were found for Mechanitis *(A) and* Melinaea *(B), combining our individuals and the individuals from* (50)*. The statistical analysis performed is Anova followed by a Tukey HSD test, with *** = p < 0.0001 ; ** = p < 0.001 ; * = p < 0.05. The phylogeny at the bottom shows the same relationships as Figure 2.*

A. *Mechanitis* – all species; each topology coloured differently

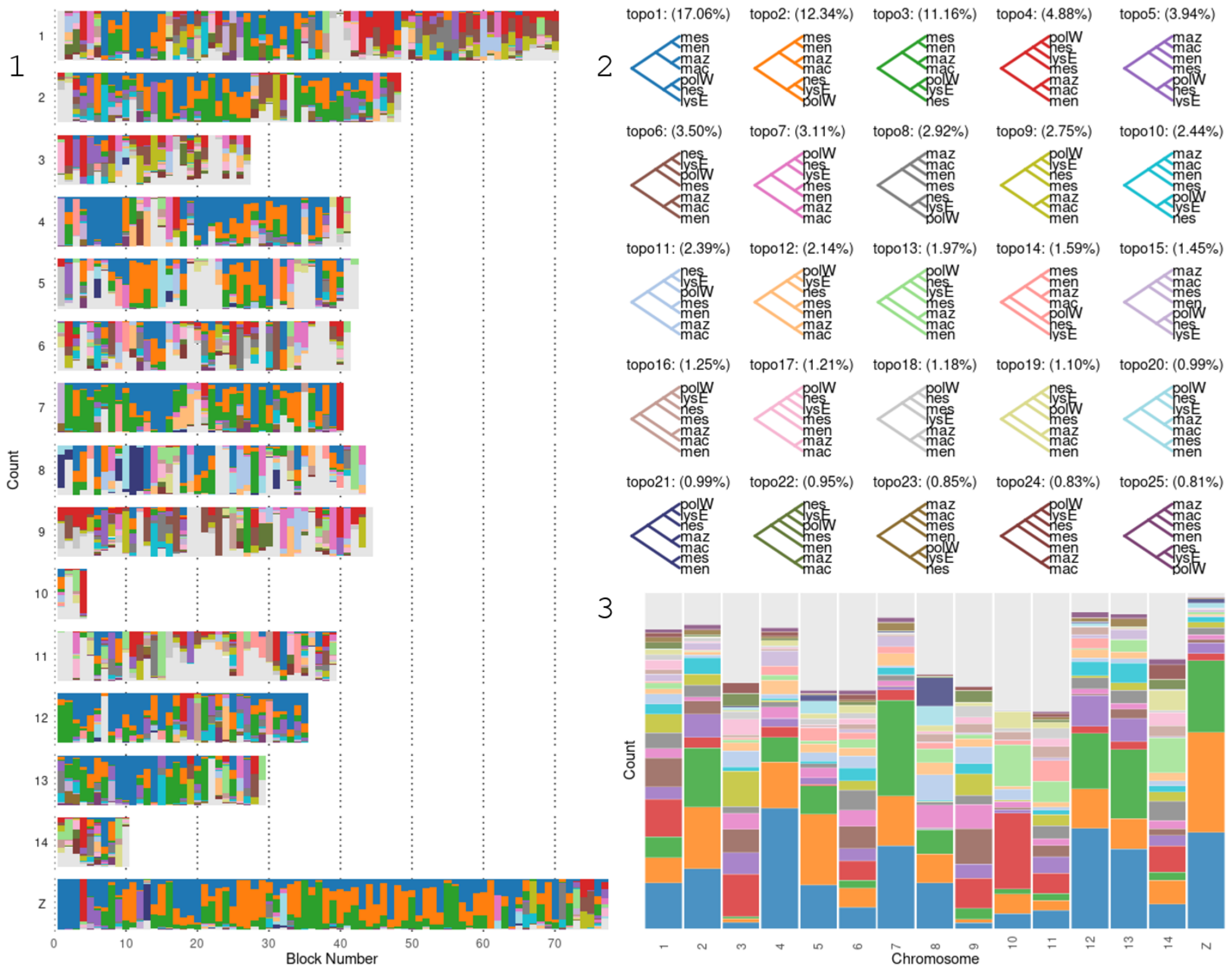

B. Mechanitis – all species; coloured by position of *Mec. messenoides*

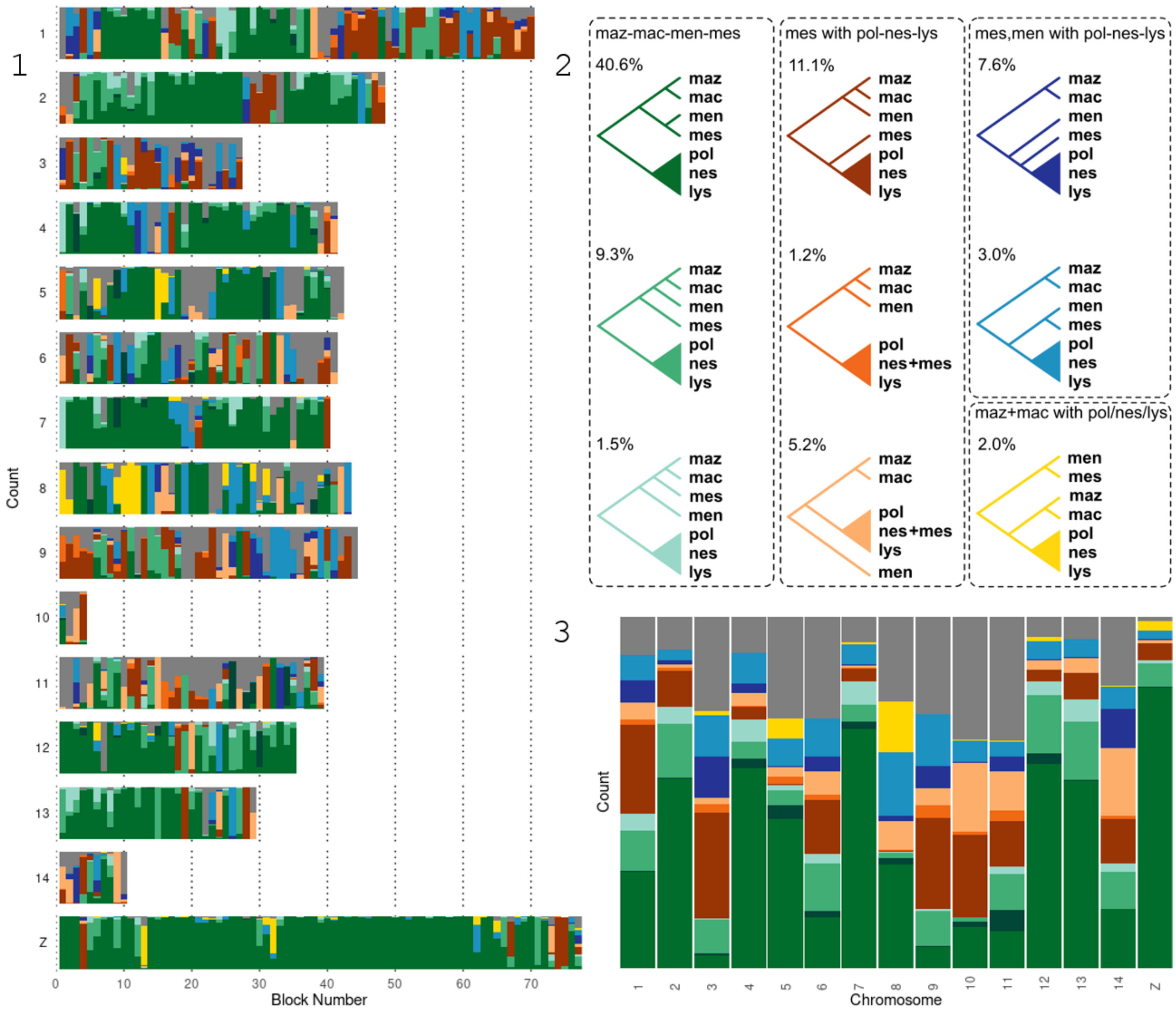

C. Mechanitis – all species; coloured by position of *Mec. nesaea*

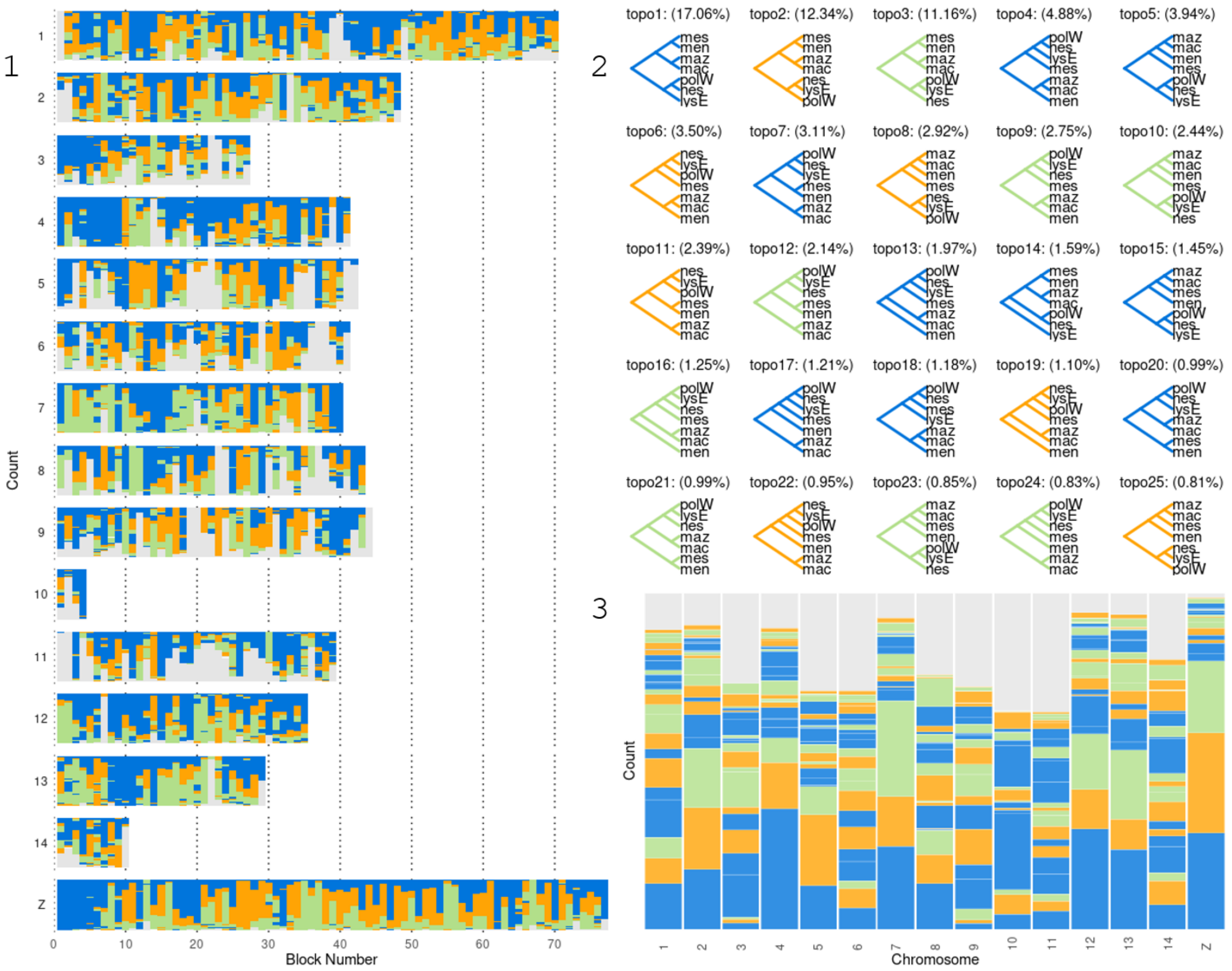

D. Mechanitis – Mec. polymnia, Mec. nesaea, Mec. lysimnia

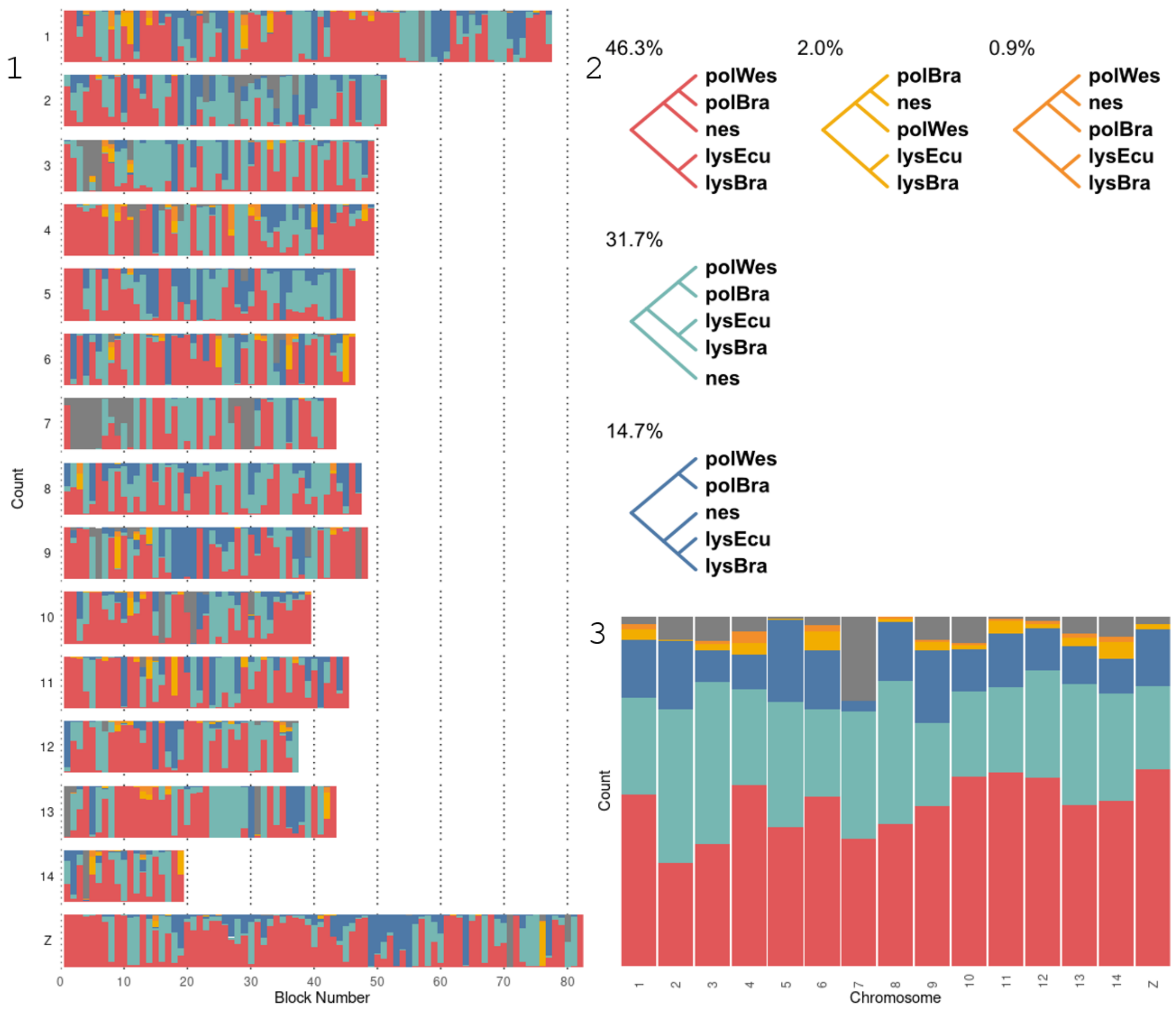

E. *Melinaea* – subset of species ; each topology coloured differently

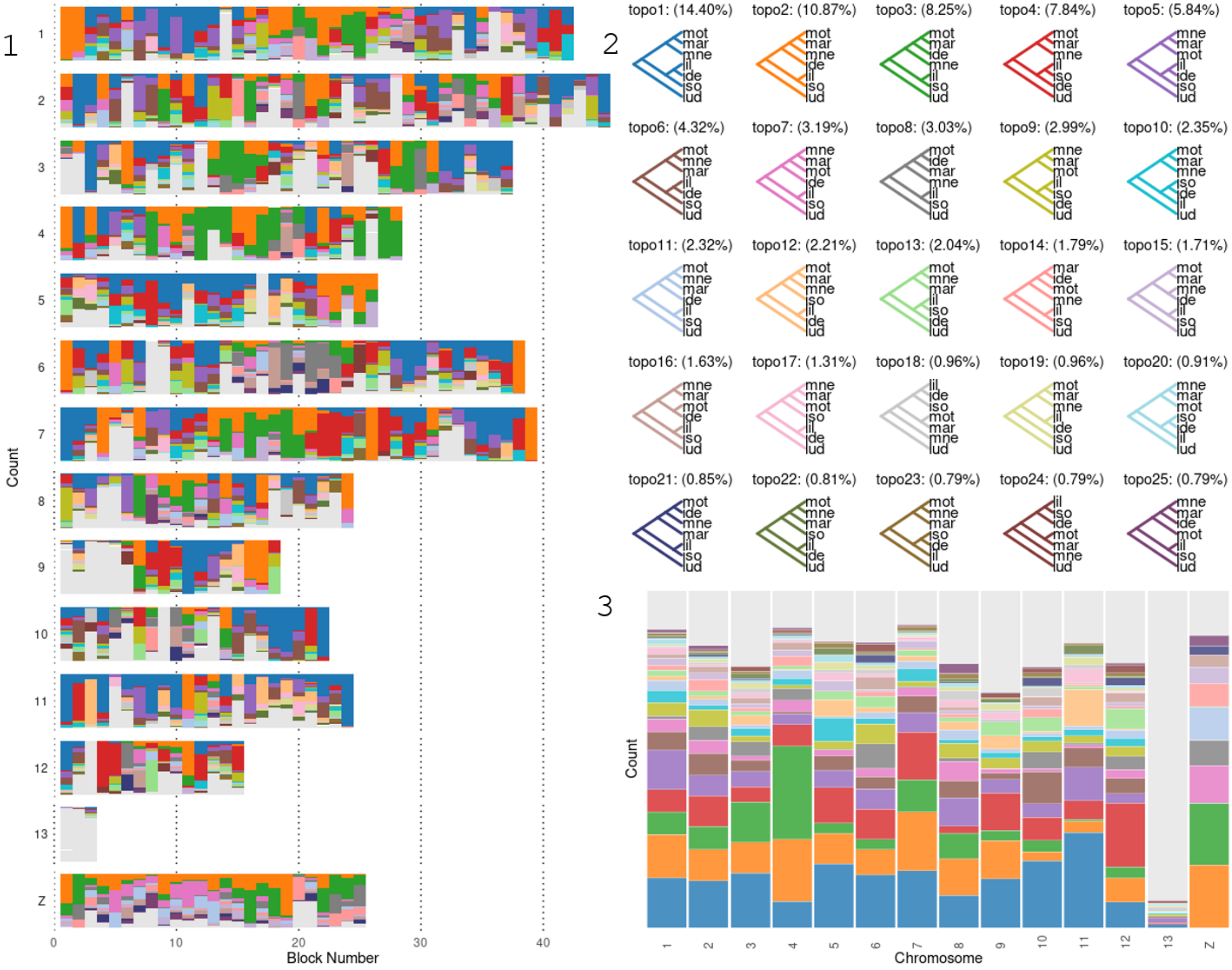

F. *Melinaea* – subset of species ; coloured by position of *Mel. idae*

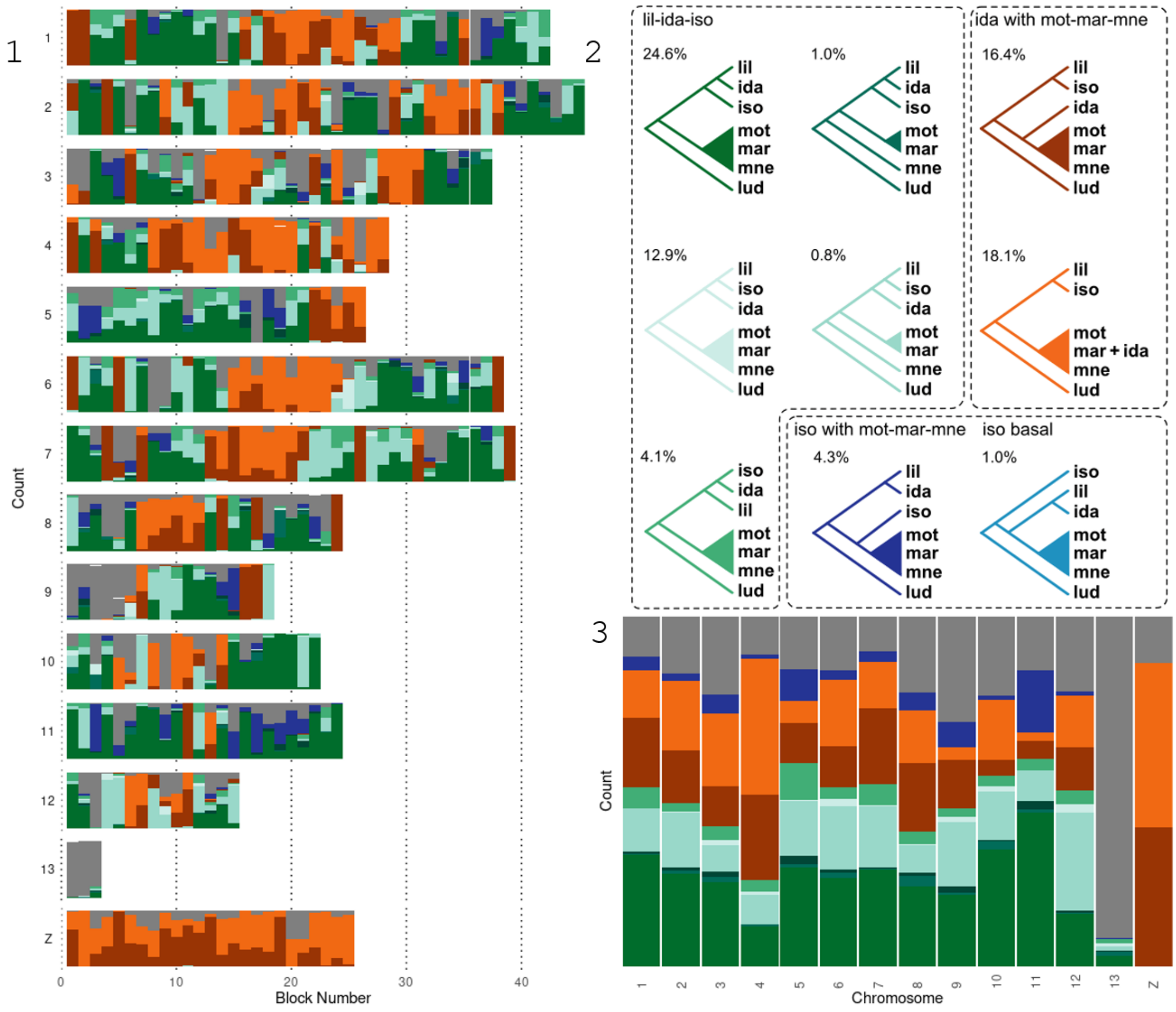

Fig. S10. BPP analysis for *Mechanitis* and *Melinaea*

BPP inferred tree topologies in windows across the genome. For each figure, part 1 shows one chromosome per row, with the windows across the chromosome coloured by the topologies for that window. Part 2 shows the various topologies, ordered by how often they occur or by an indicated grouping. The number above each topology indicates what percentage of the genome that topology occurs. Each column of part 3 represents a chromosome, coloured by how much each topology occurs in that chromosome. Grey colouration means that that window had a topology that occurred less than one of the top25 topologies. A, B and C show results for the analysis consisting of one individual of each Mechanitis *species, with A coloured differently for each topology, B coloured according to the position of* Mec. messenoides*, and C coloured for the position of* Mec. nesaea*. mes = Mec. messenoides; men = Mec. menapis; maz = Mec. mazaeus; mac = Mec. macrinus; polW = Mec. polymnia (west); nes = Mec. nesaea; lysE = Mec. lysimnia (Ecuador). D is the result of an analysis including* Mec. polymnia *from Brazil (polBra) and west of the Andes (polWes),* Mec. nesaea *(Brazil; nes) and* Mec. lysimnia *from Brazil (lysBra) and Ecuador (lysEcu). E and F are the results for a subset of the* Melinaea *species, with E coloured differently for each topology, and F coloured by the placement of* Mel. idae*. mot = Mel. mothone; mar = Mel. marsaeus; mne = Mel. mneme; lil = Mel. lilis; ida = Mel. idae; iso = Mel. isocomma; lud = Mel. ludovica.*

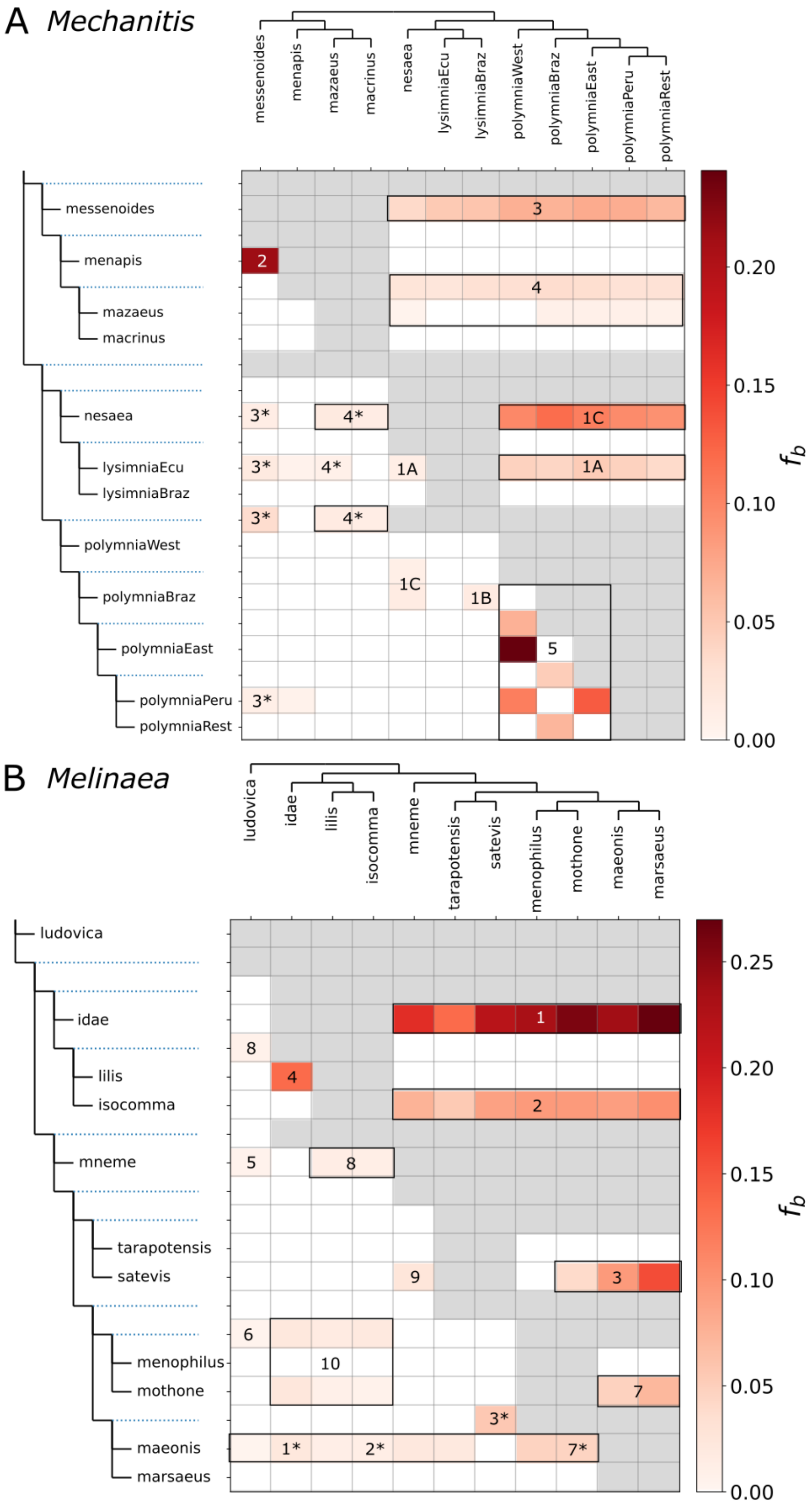

Fig. S11. Fbranch DSuite results

Fbranch results for Mechanitis *(A) and* Melinaea *(B). Stronger red colour indicates more excess allele sharing between the species shown on top and the species shown on the left as compared to the sister clade of the latter according to the phylogeny on the left. Note, if the direction of gene flow is from the species on top to the species on the left, close relatives of the species on top are also expected to show excess allele sharing. The numbers indicate which excess allele sharing pattern was used to draw the hybridisation arrows in Figure 2.*

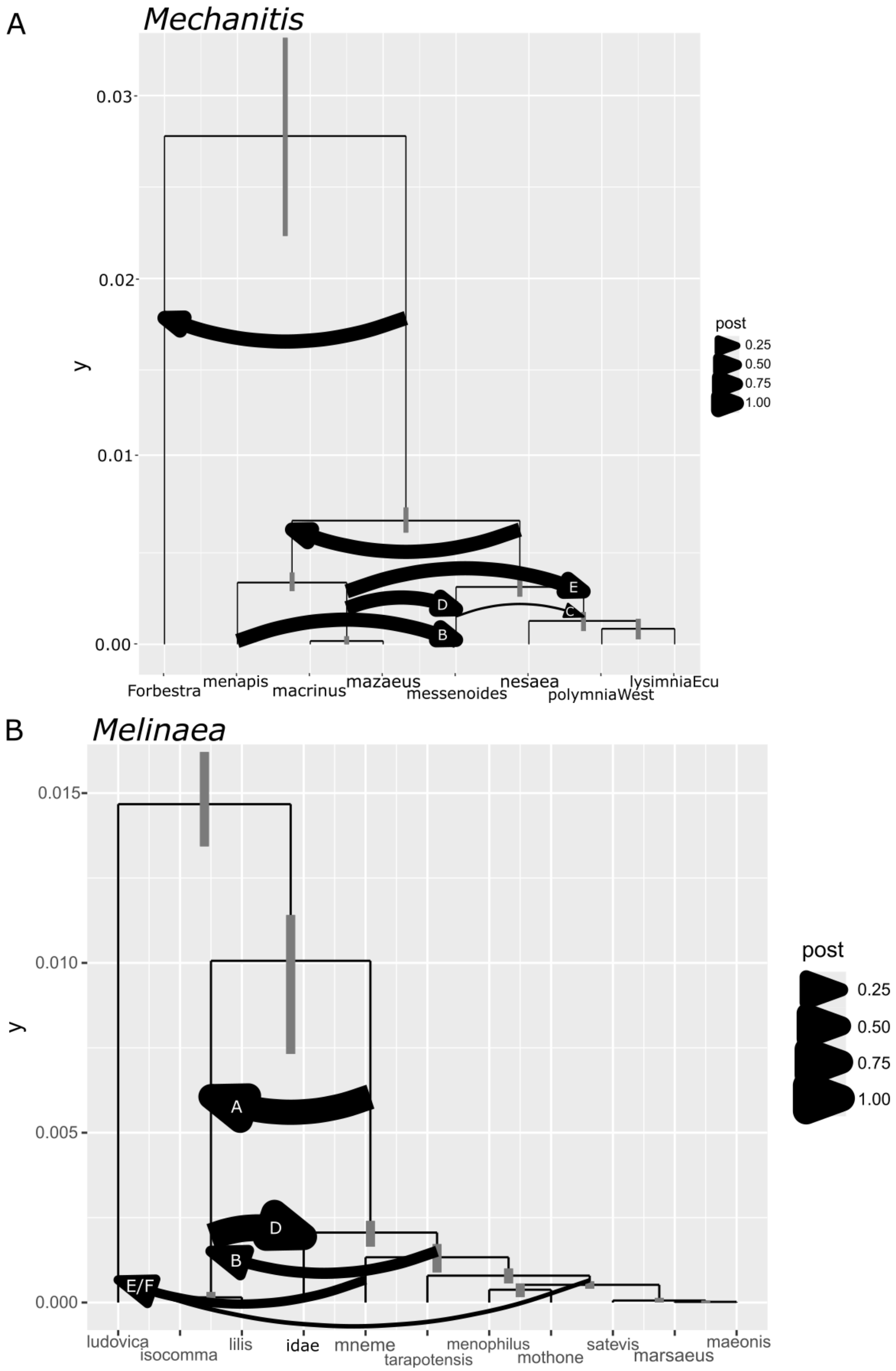

Fig. S12. AIM (BEAST2) admixture trees

A tree with a set of admixture arrows that might explain the phylogenetic history of Mechanitis *(A) and* Melinaea *(B), as produced by AIM. The letters in the arrows are used in the text to refer to different admixture events and they correspond to arrows in Figure 2.*

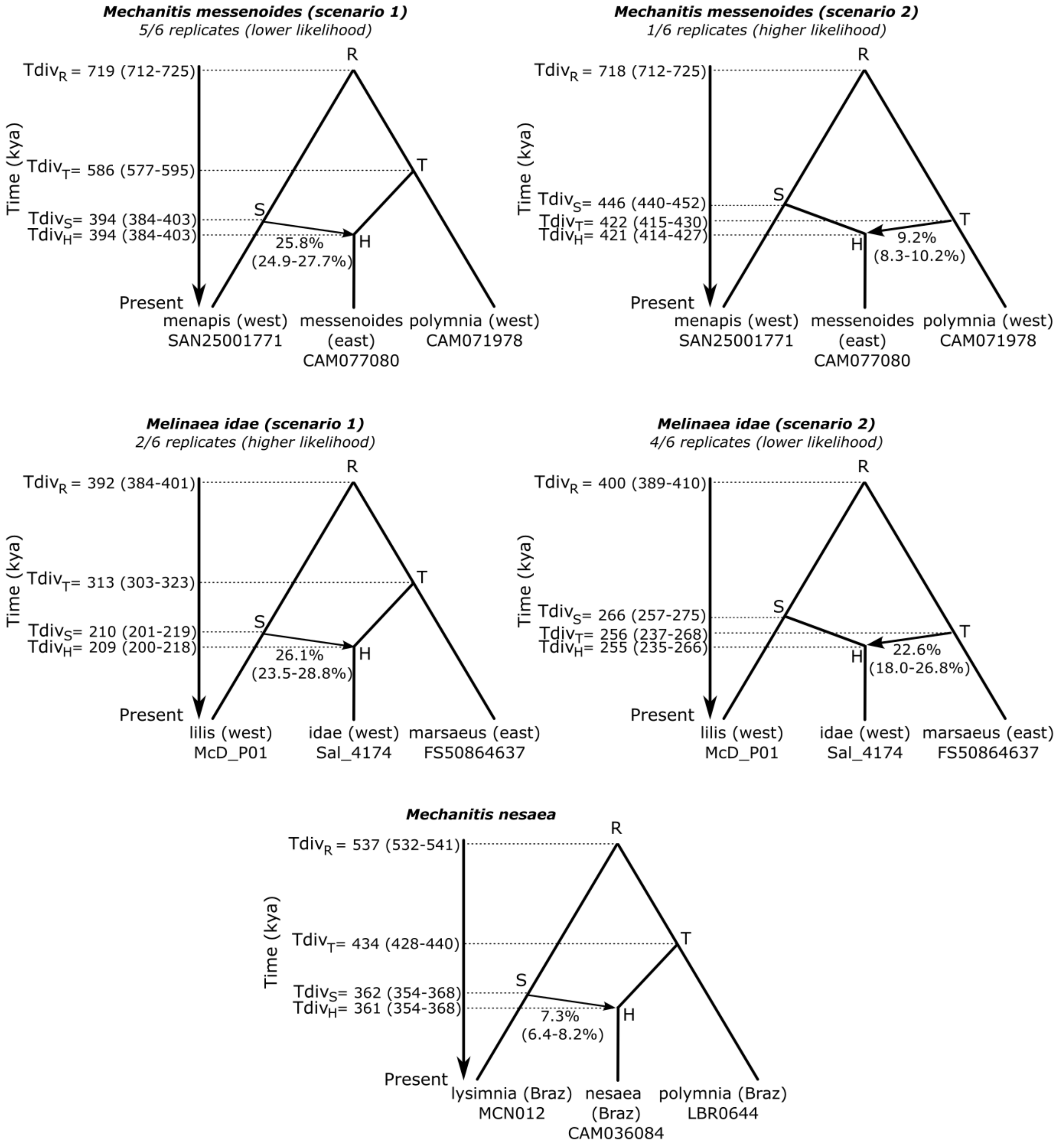

Fig. S13. MSCi model results

The figure shows the multi-species coalescent model with introgression (MSCi) for each introgressed species of interest, with the obtained divergence times and the corrresponding confidence intervals. We assumed 4 generations per year, and a mutation rate of 2.9x10^9. For Mechanitis messenoides and Melinaea idae, different replicates converged on a different output, so we have provided both scenarios. The branching order is not pre-determined in the model; T can be younger or older than S.

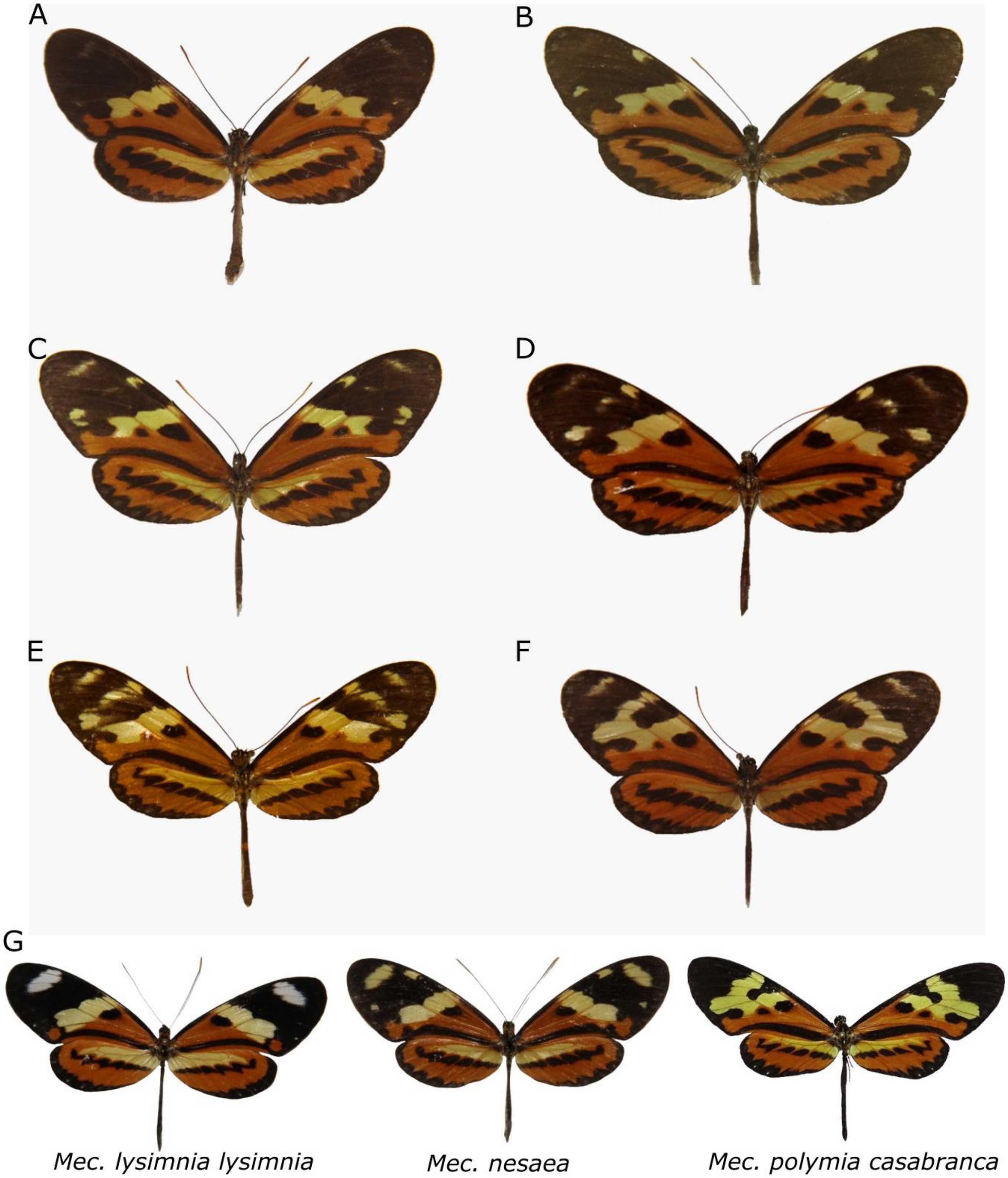

Fig. S14. Hybrids between *Mec. polymnia* and *Mec. lysimnia* look like *Mec. nesaea*

Hybrids between Mec. l. lysimnia and Mec. p. casabranca found in Brazil, some (e.g. C) look like the putative hybrid species Mec. nesaea. Photos coming from: A) Brazil, São Paulo, São Vicente, Morro do Japui, 25.v.1991 - ZUEC-AVLF. B) Brazil, Minas Gerais, Itueta, viii.1970 – ZUEC. C) Brazil, Minas Gerais, Itueta, v.1970 – ZUEC. D) Brazil, Espírito Santo, Baixo Guandú, vi.1970 – ZUEC. E) Brazil, Espírito Santo, Baixo Guandú, v.1970 – ZUEC. F) Brazil, Espírito Santo, Baixo Guandú, v.1970 – ZUEC. ZUEC = Zoological collection, Museu de Diversidade Biológica da Universidade Estadual de Campinas, Campinas, São Paulo, Brazil. ZUEC-AVLF = André V.L. Freitas Collection, Universidade Estadual de Campinas, Campinas, São Paulo, Brazil. G) Photos of Mec. lysimnia lysimnia, Mec. nesaea, and Mec. polymnia casabranca.

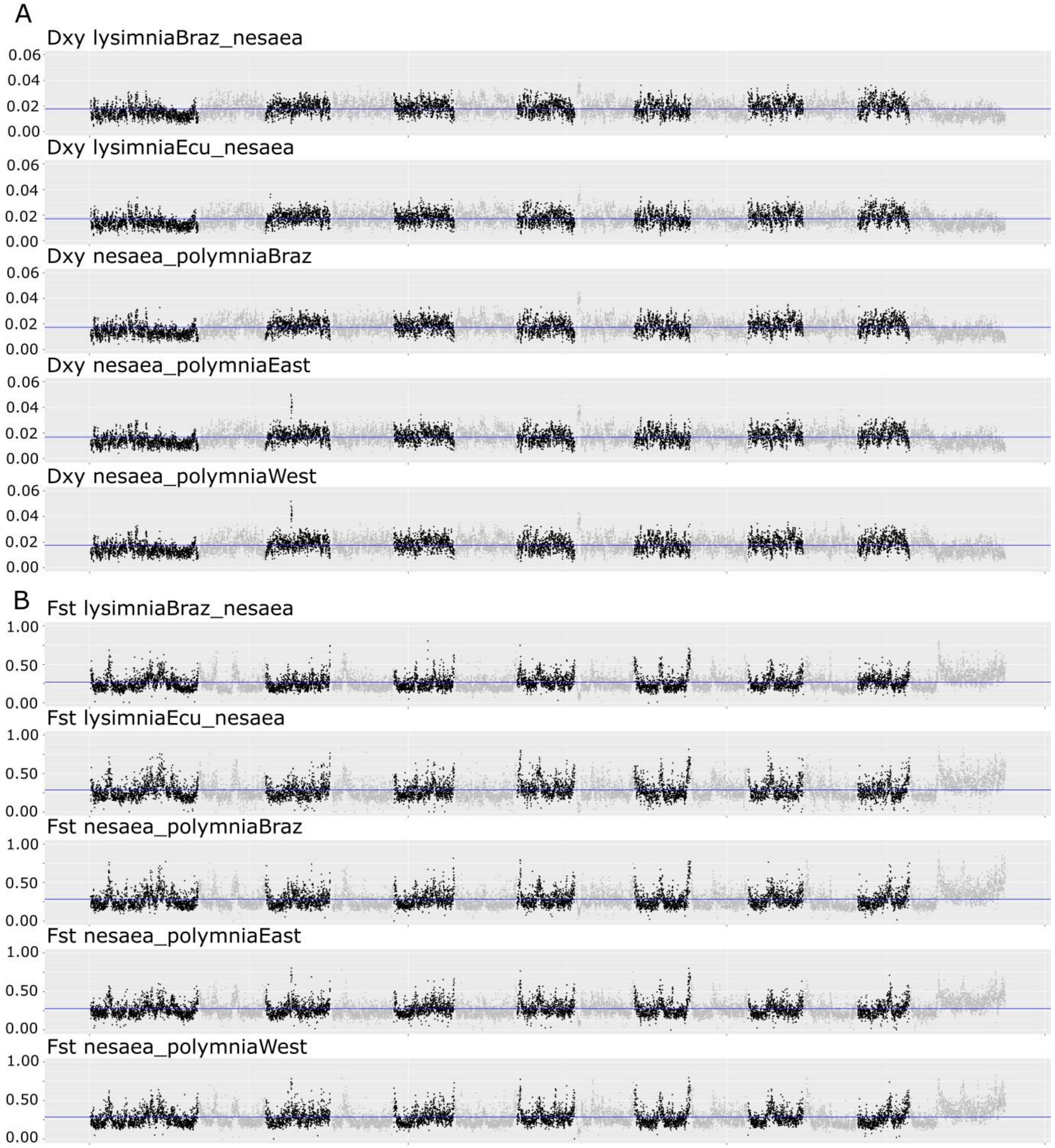

Fig. S15. Divergence and diversity in *Mec. polymnia, Mec. lysimnia* and *Mec. nesaea*

A) Dxy absolute divergence analysis. The title of each row indicates between which two populations the Dxy is calculated. High peaks indicate more divergence. B) Fst relative differentiation analysis. The title of each row indicates between which two populations the Fst was calculated. High peaks indicate more differentiation. Black versus grey dots indicate a new chromosome. Window-size was 20kb.

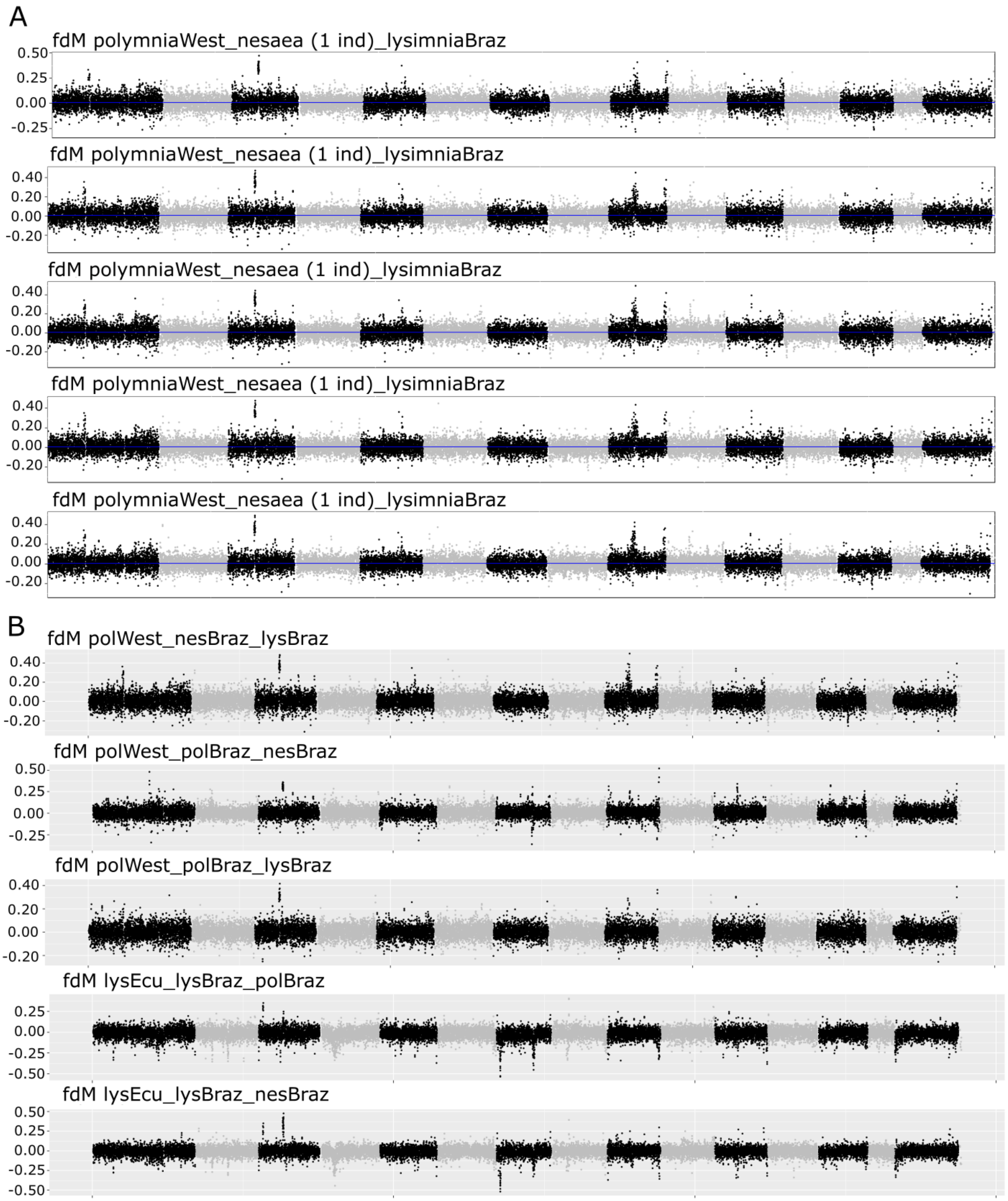

Fig. S16. Introgression analysis in Mec. polymnia, Mec. lysimnia and Mec. nesaea

***A**) fdM introgression analysis with P1 = Mec. polymnia (from West of the Andes), P2 = Mec. nesaea (1 individual from Brazil per row) and P3 = Mec. lysimnia (Brazil). P4 is the outgroup Forbestra. The same analysis was done for five different individuals of Mec. nesaea, to examine how consistent the results are between individuals. High fdM indicates high introgression between P2 and P3 (Mec. nesaea and Mec. lysimnia). **B**) Various fdM introgression analyses to compare gene flow between different populations. The order of the names in the titel of each row gives P1, P2 and P3. Forbestra is the outgroup (P4) in each analyses. One can see that certain peaks of introgression are shared between populations. Black versus grey dots indicate a new chromosome.*

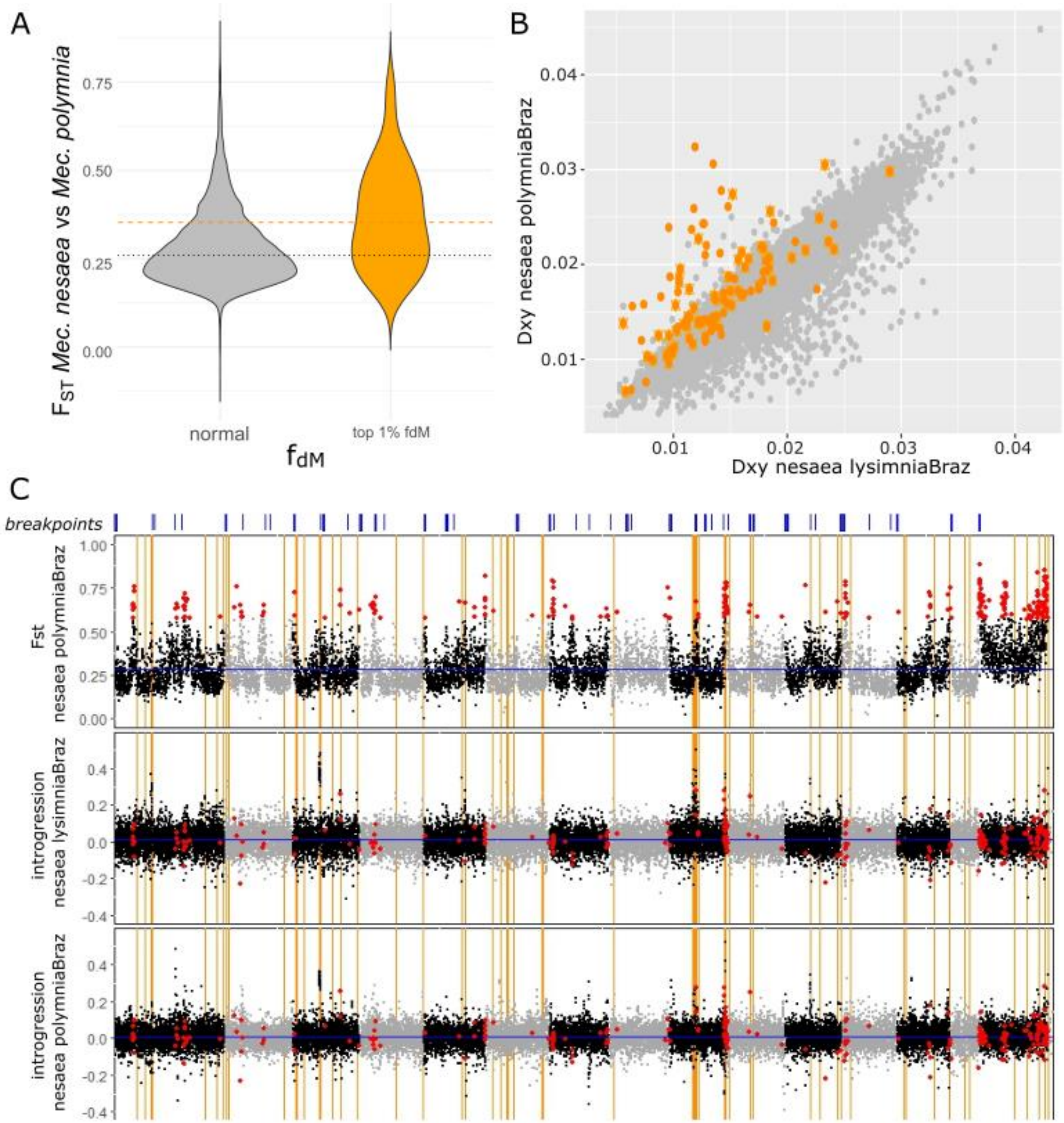

Fig. S17. ***Mec. nesaea*** **differentiation and introgression**

Mec. nesaea *shows increased differentiation from its sister species* Mec. polymnia *in regions with strong signatures of introgression with* Mec. lysimnia*. A) F_{ST} between* Mec. polymnia *and* Mec. nesaea *computed in 20kb windows is higher in top 1% f_{dM} windows (orange) than in the rest of the genome (grey, Kruskal-Wallis test: 39.395, df = 1, p-value < 3.5e-10). The dotted black horizontal line indicates the median value of Fst, and the dashed orange horizontal line indicates the median of the top 1% f_{dM} windows. B) D_{XY} values computed between* Mec. nesaea *and both close relatives with the top 1% fdM windows highlighted in orange. The orange stars are windows that also have low fdM values for* Mec. nesaea *and* Mec.

lysimnia. *C) A scan across the genome (20kb windows) showing* F_{ST} *(*Mec. nesaea *vs* Mec. polymnia *of Brazil) and* f_{dM} *(P1=polymniaWest, P2=nesaea, P3=lysimniaBrazil, P4=Forbestra) and (P1=polymniaWest, P2=polymniaBrazil, P3=nesaea, P4=Forbestra). Blue lines at the top indicate potential chromosomal breakpoints (breakpoints from* Mec. polymnia *with other four reference species, as we don't have a reference genome for* Mec. nesaea*). Orange vertical lines indicate regions with strong signatures of introgression (top 0.5%). The red dots are the top 2% Fst windows.*

Mec. macrinus

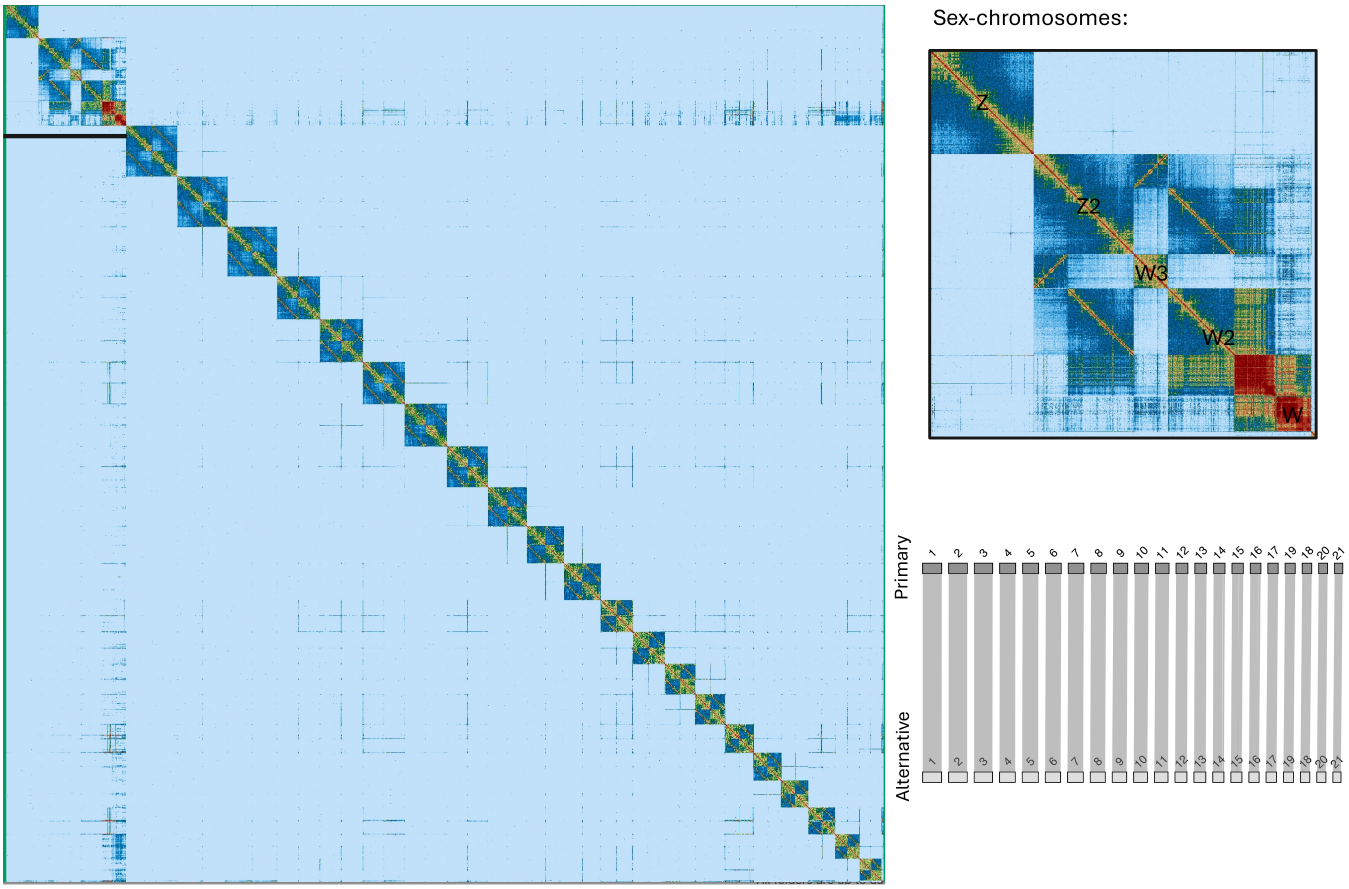

Mec. mazaeus

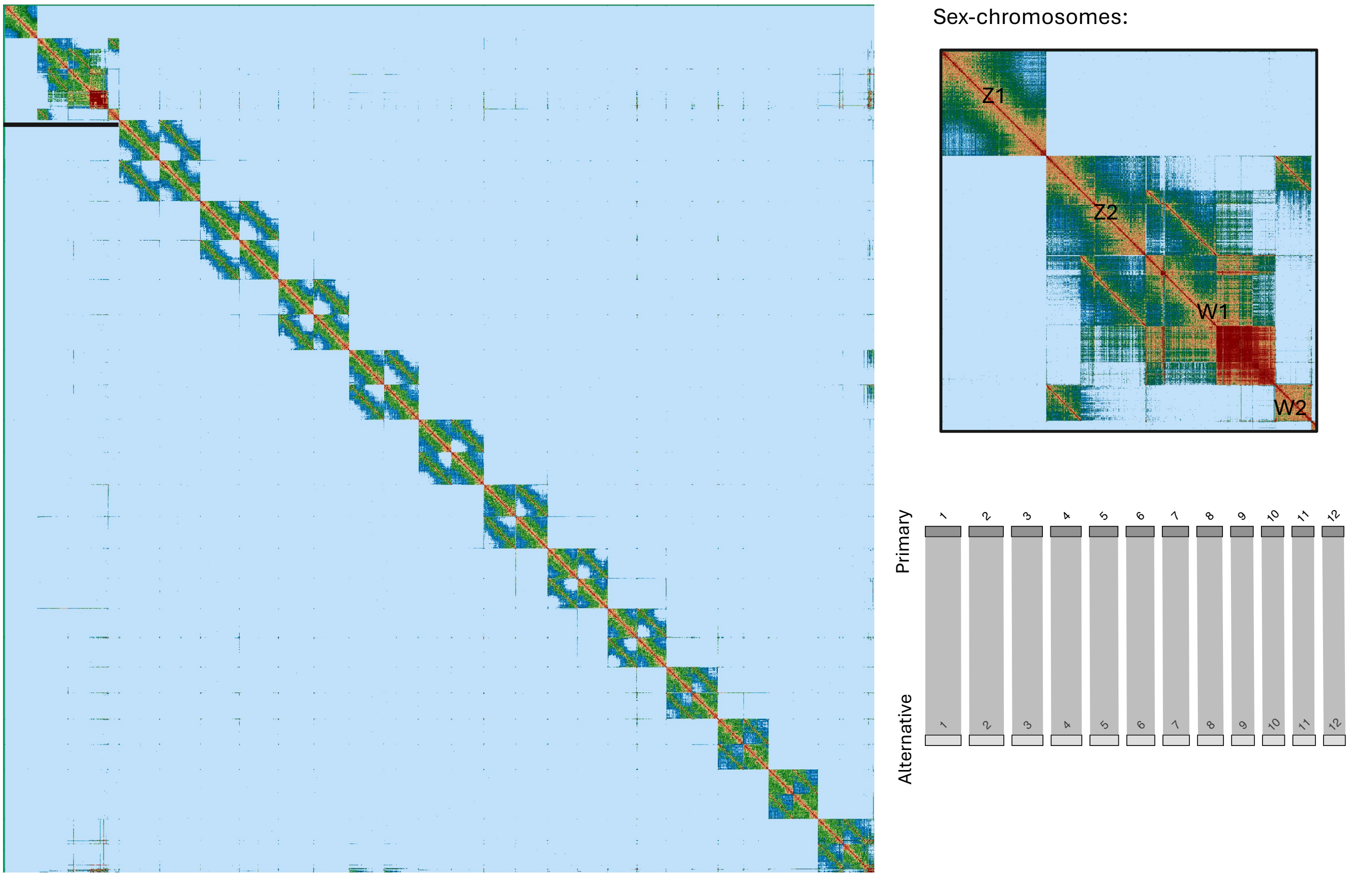

Mec. menapis

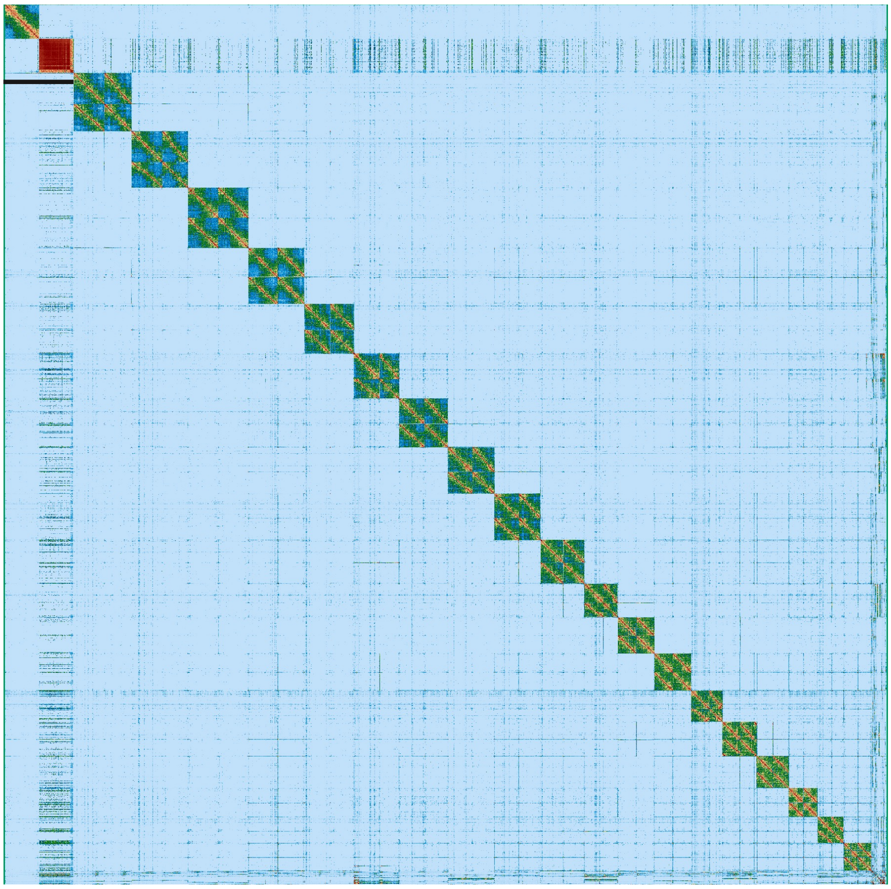

Sex-chromosomes:

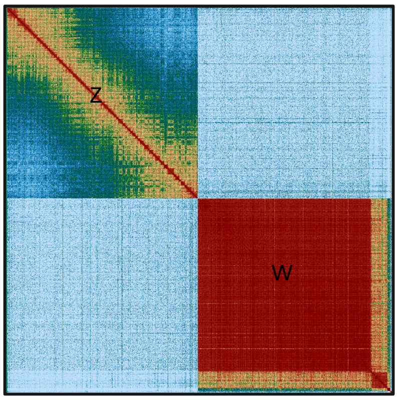

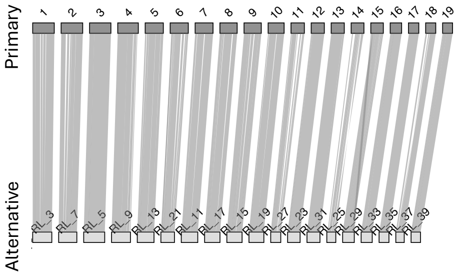

Mec. messenoides

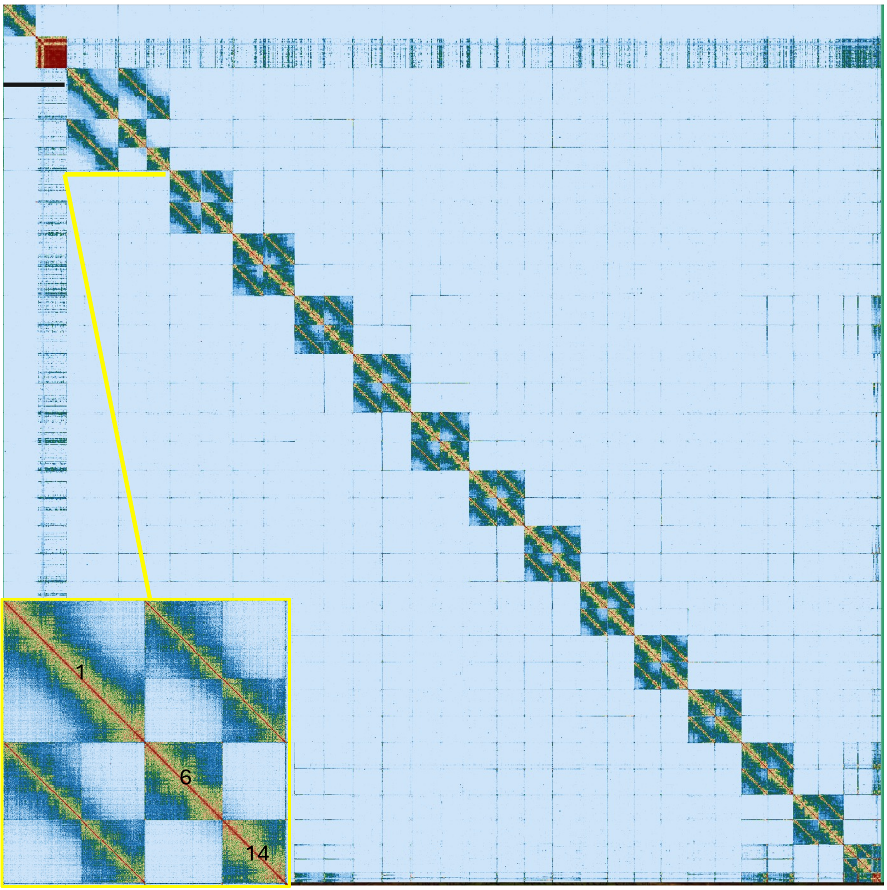

Sex-chromosomes:

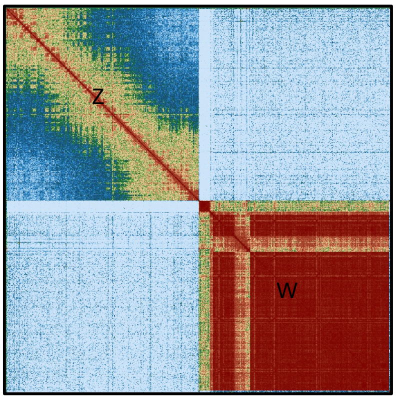

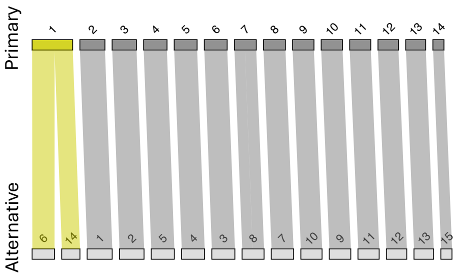

Mec. polymnia

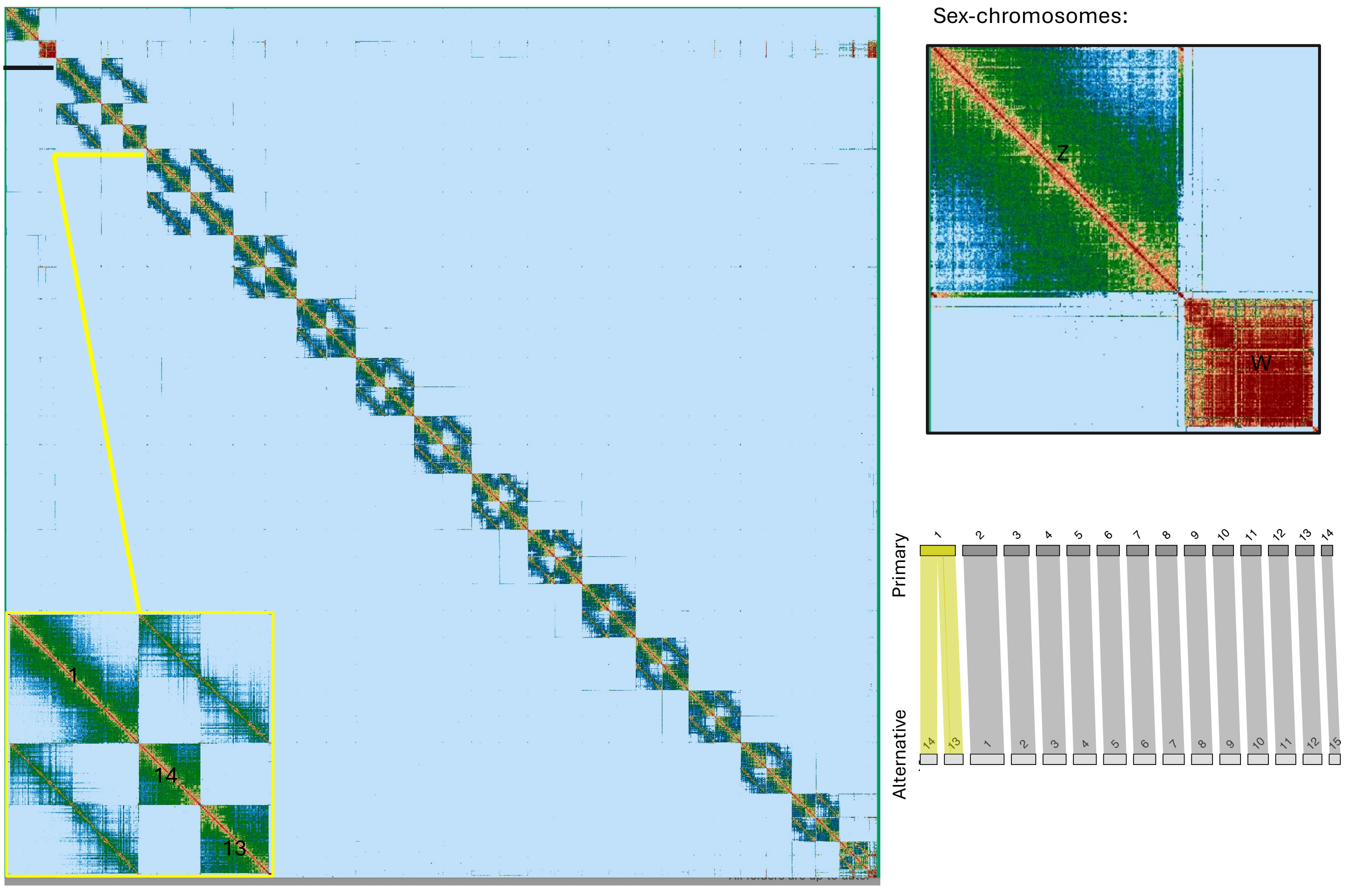

Fig. S18A*. *Haplotype mapping. *HiC contact maps of reads mapped simultaneously to the two haplotype phased assemblies in each* Mechanitis *genome, with sex-chromosomes (black line) and heterozygous rearrangements (yellow line) highlighted. Insets: Examples of autosomal heterozygous rearrangements. Right panel: Zoom in of sex-chromosomes. Bottom panel: Whole genome alignment between the two phased assemblies, excluding hemizygous chromosomes.*

Mel. ludovica

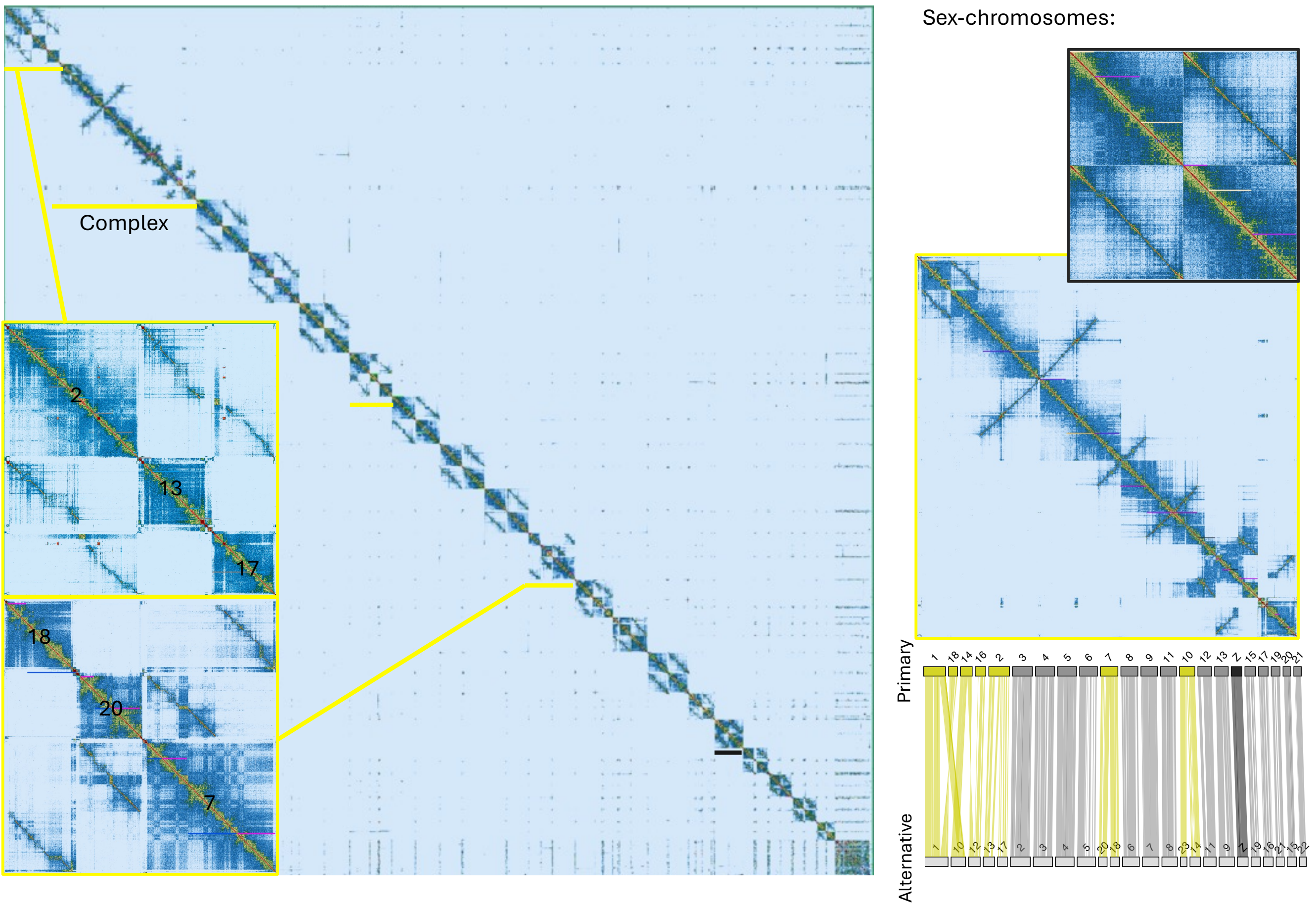

Mel. isocomma

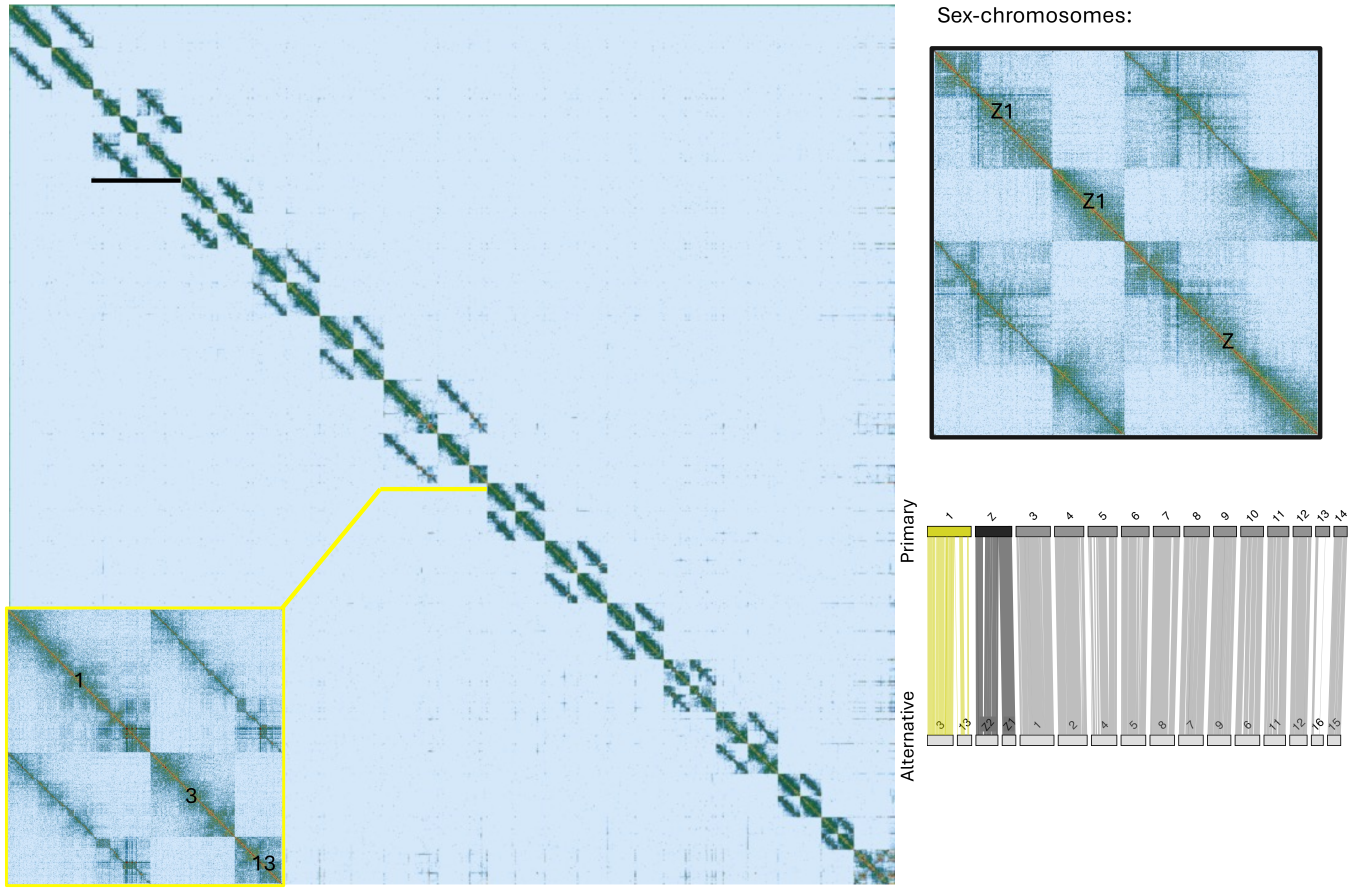

Mel. mothone

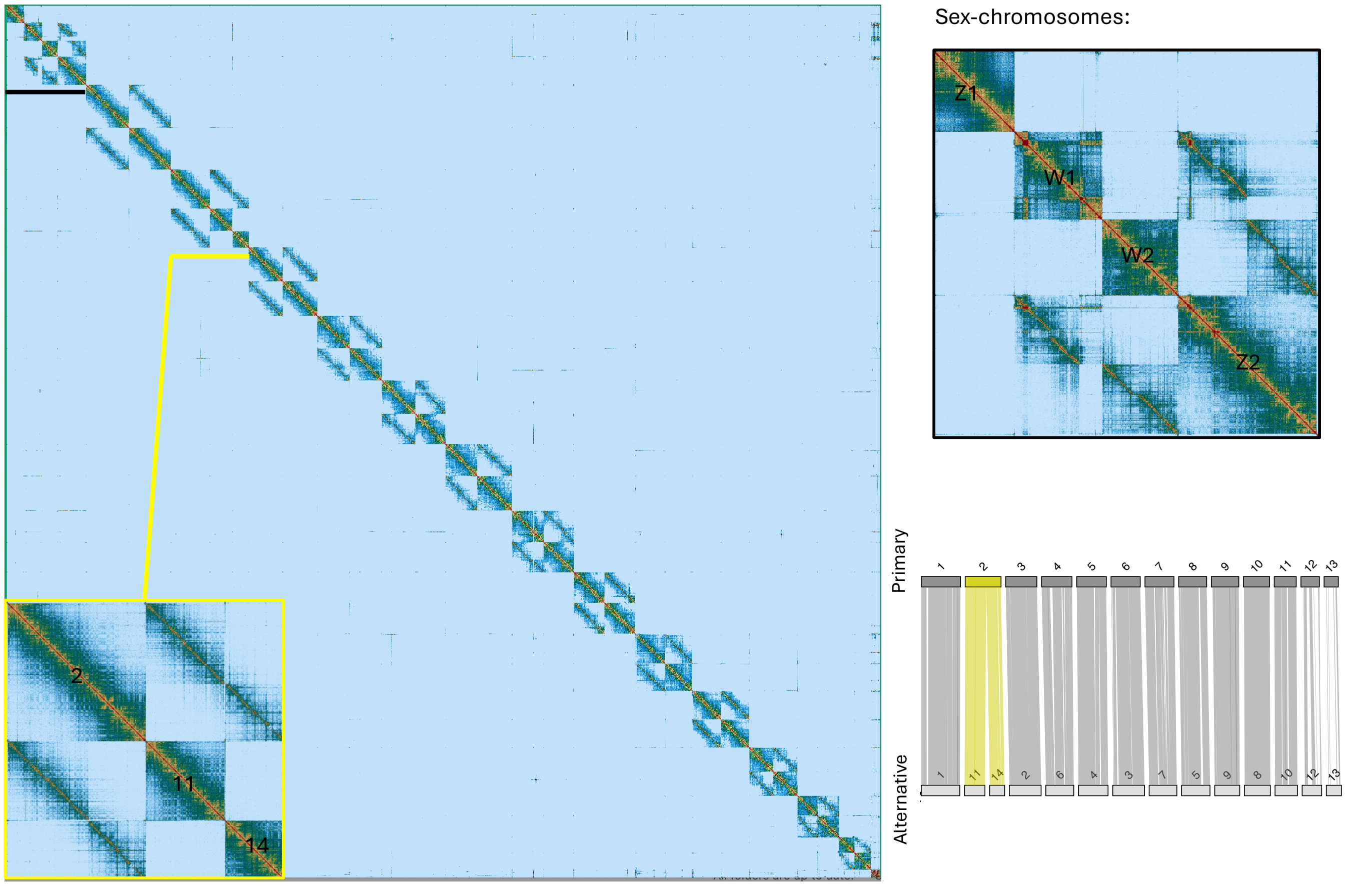

Mel. menophilus

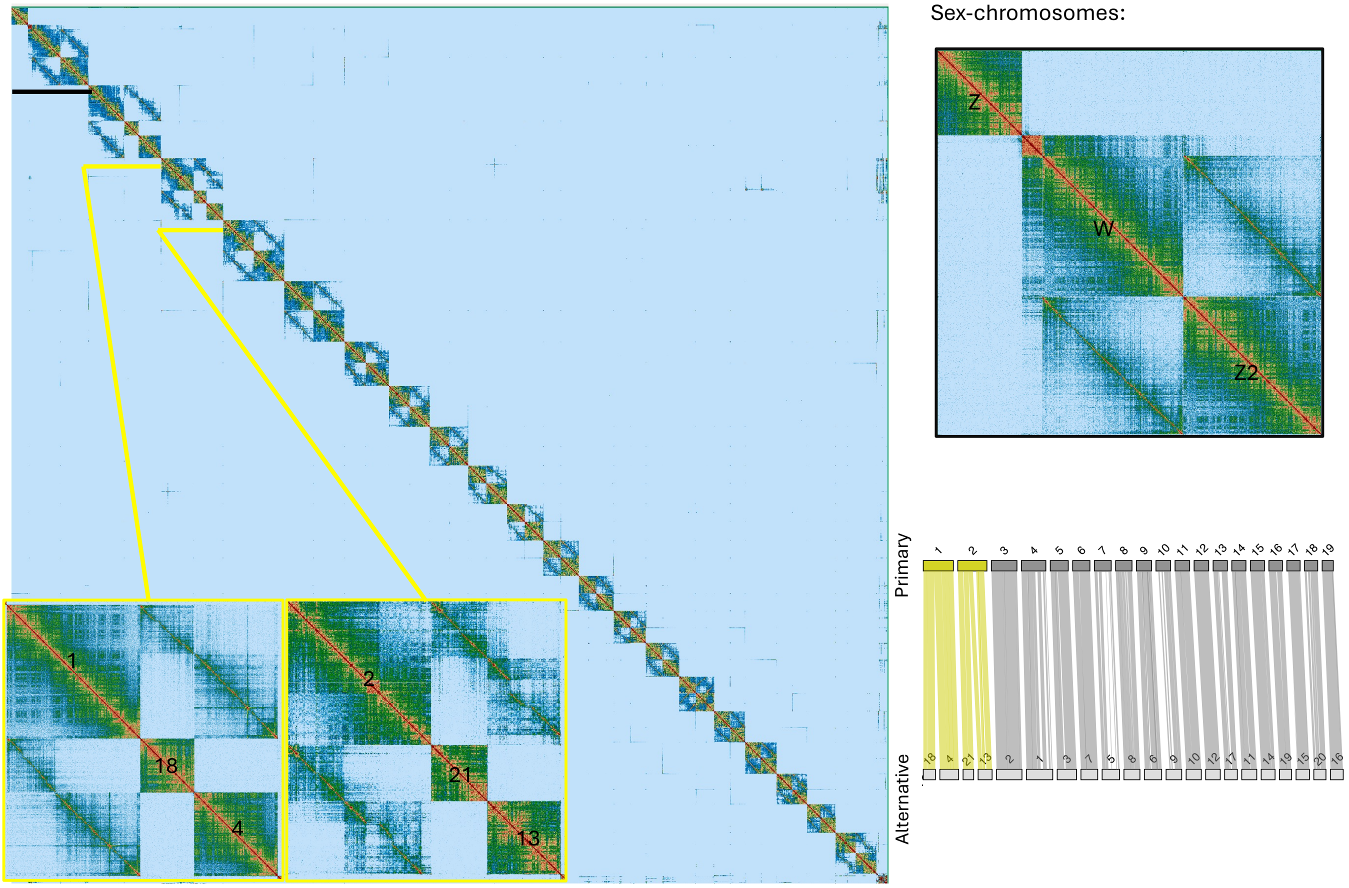

Mel. marsaeus

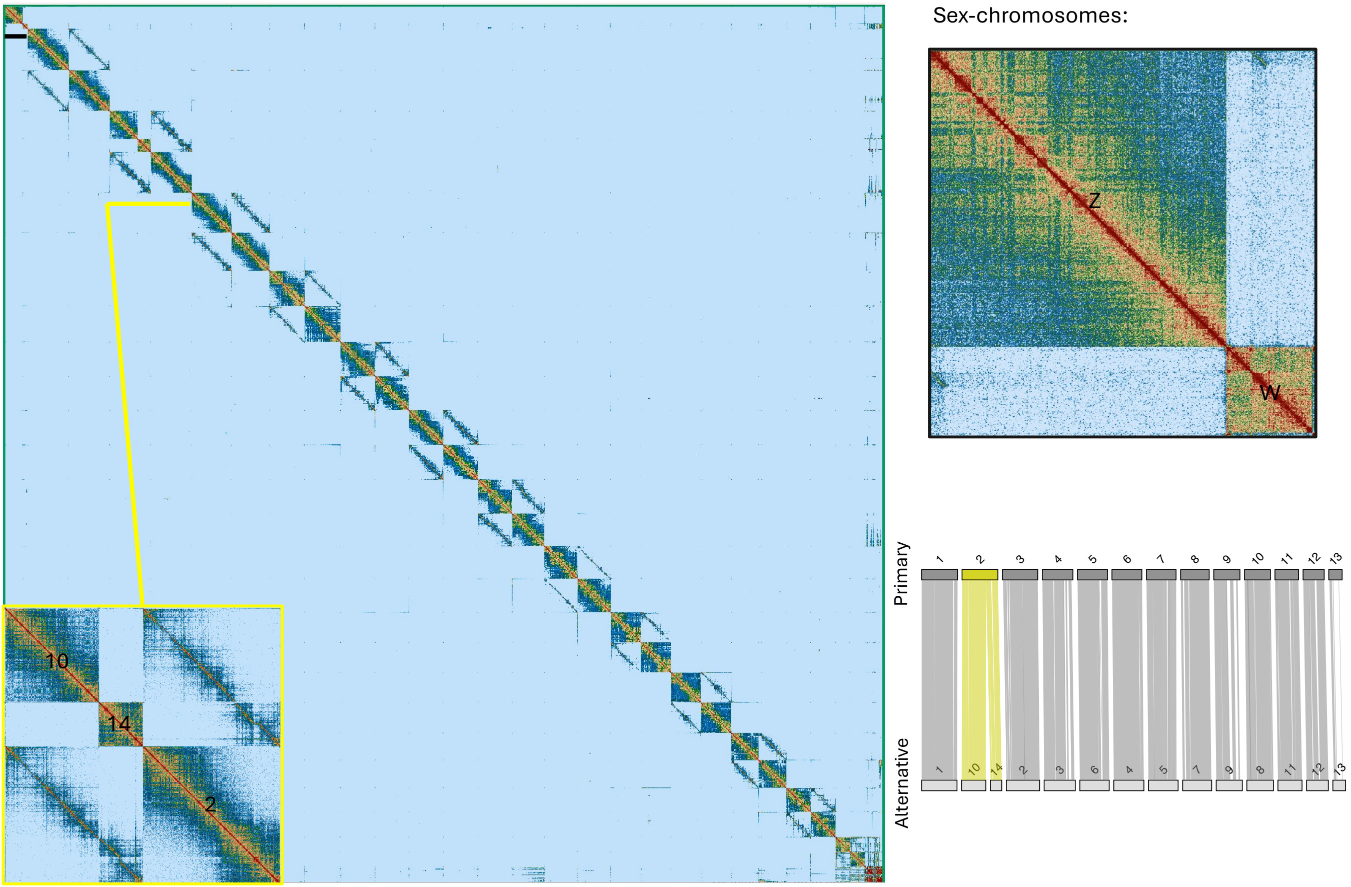

***Fig. S18B**. **Haplotype mapping.** HiC contact maps of reads mapped simultaneously to the two haplotype phased assemblies in each* Melinaea *genome, with sex-chromosomes (black line) and heterozygous rearrangements (yellow line) highlighted. Insets: Examples of autosomal heterozygous rearrangements. Right panel: Zoom in of sex-chromosomes. Bottom panel: Whole genome alignment between the two phased assemblies, excluding hemizygous chromosomes.*

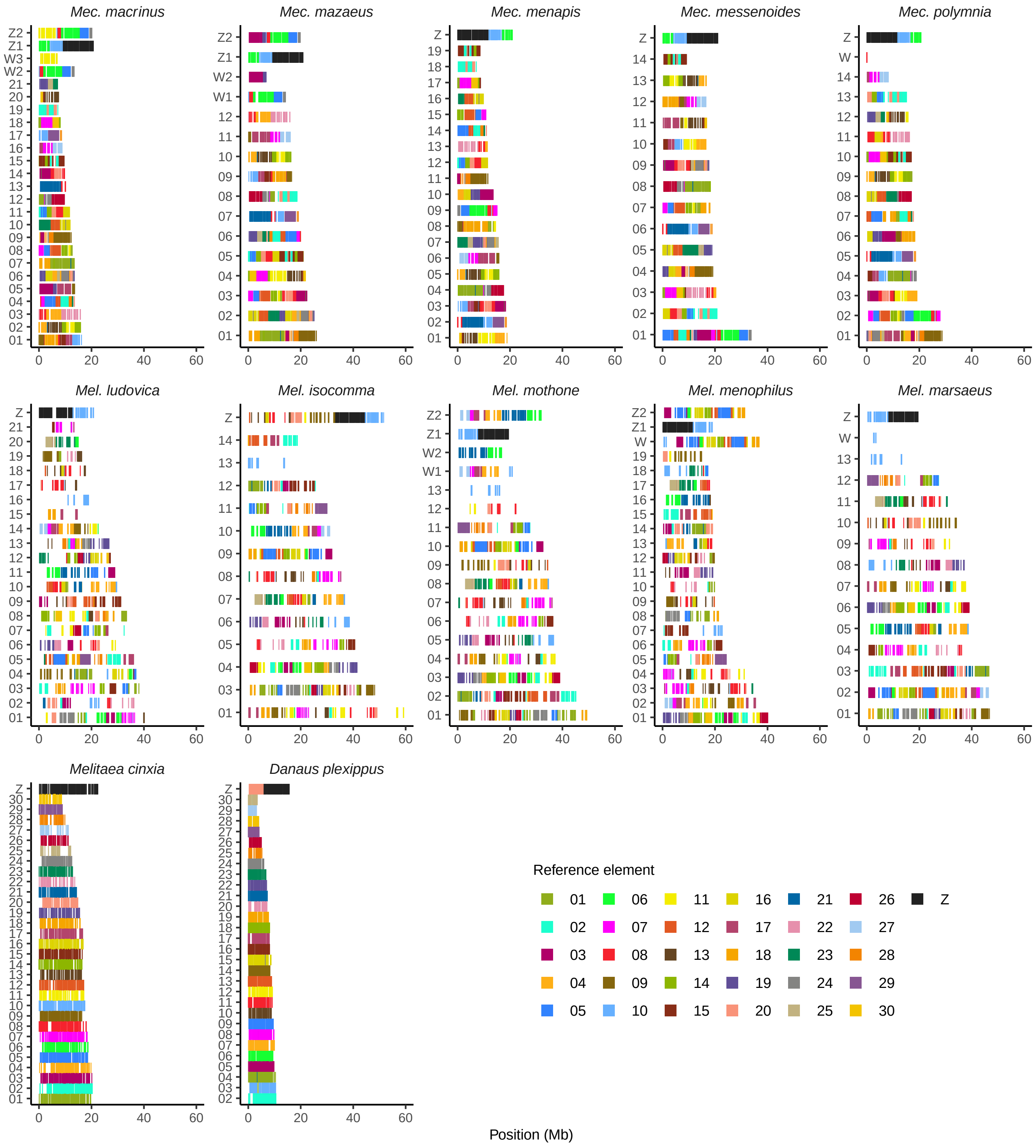

Fig. S19. Chromosome BUSCO painting

Complete and duplicated BUSCO-genes in Melinaea *and* Mechanitis *genomes coloured coloured by chromosome position in* Melitaea cinxia*. Outgroups* M. cinxia *and* Danaus plexippus *included for comparison. The chromosome number is on the y-axis and the start position of the BUSCO genes in megabases on the x-axis (Position(Mb)).*

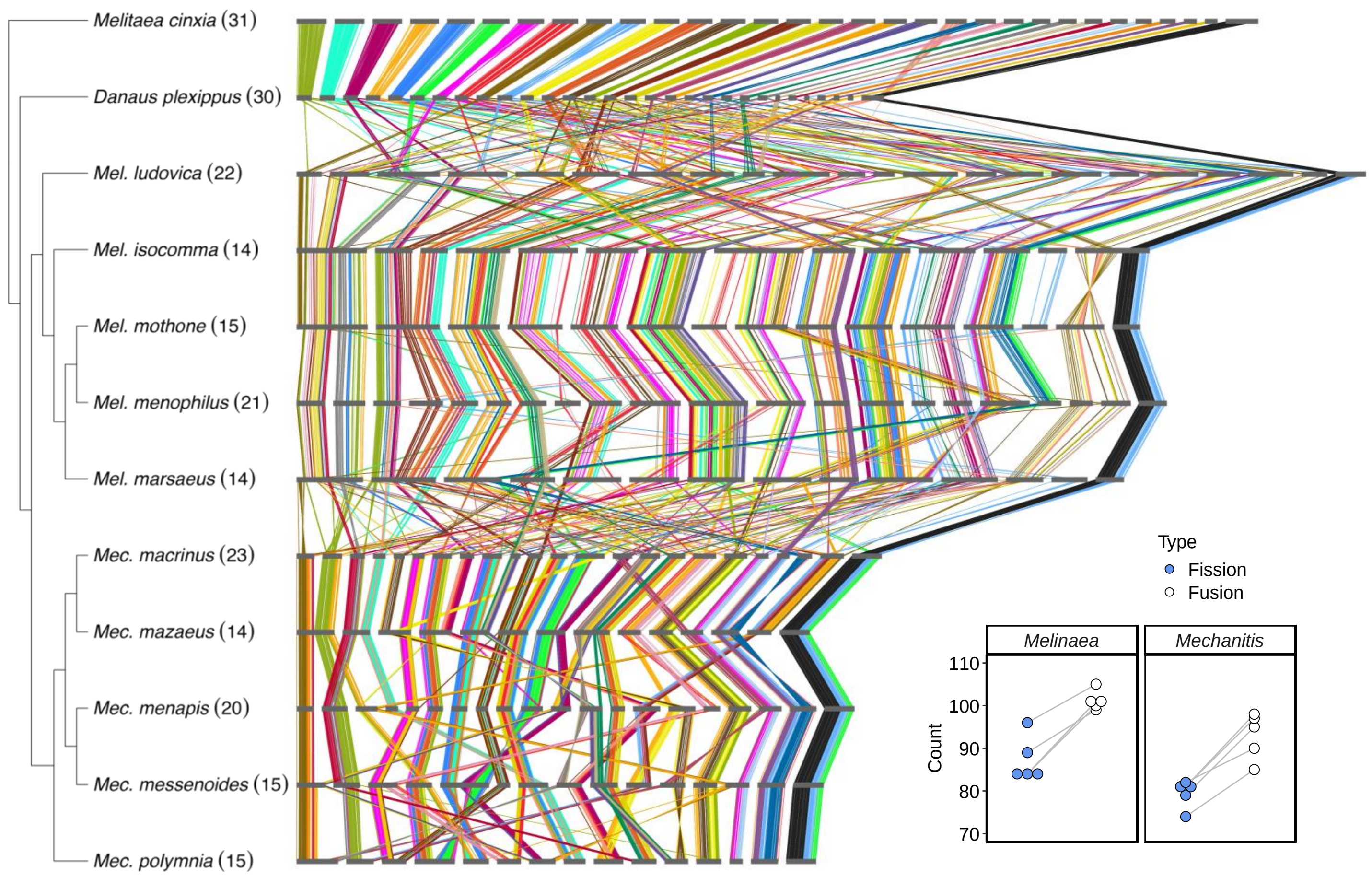

Fig. S20. Synteny between *Melinaea* and *Mechanitis*

Synteny between Melinaea *(yellow) and* Mechanitis *(blue) genomes based on BUSCO gene positions using* Melitaea cinxia *and* Danaus plexippus *as outgroups (grey). Horizontal bars represent individual chromosomes and vertical links represent BUSCO genes coloured by their ancestral linkage group assignment performed with the reference species* Melitaea cinxia*. The cladogram is based on Fig. 2 and shows haploid chromosome numbers in parentheses. Inset: Count of minimum number of chromosomal fission (blue) and fusion (white) events in* Melinaea *and* Mechanitis *relative the reference species* Melitaea cinxia*.*

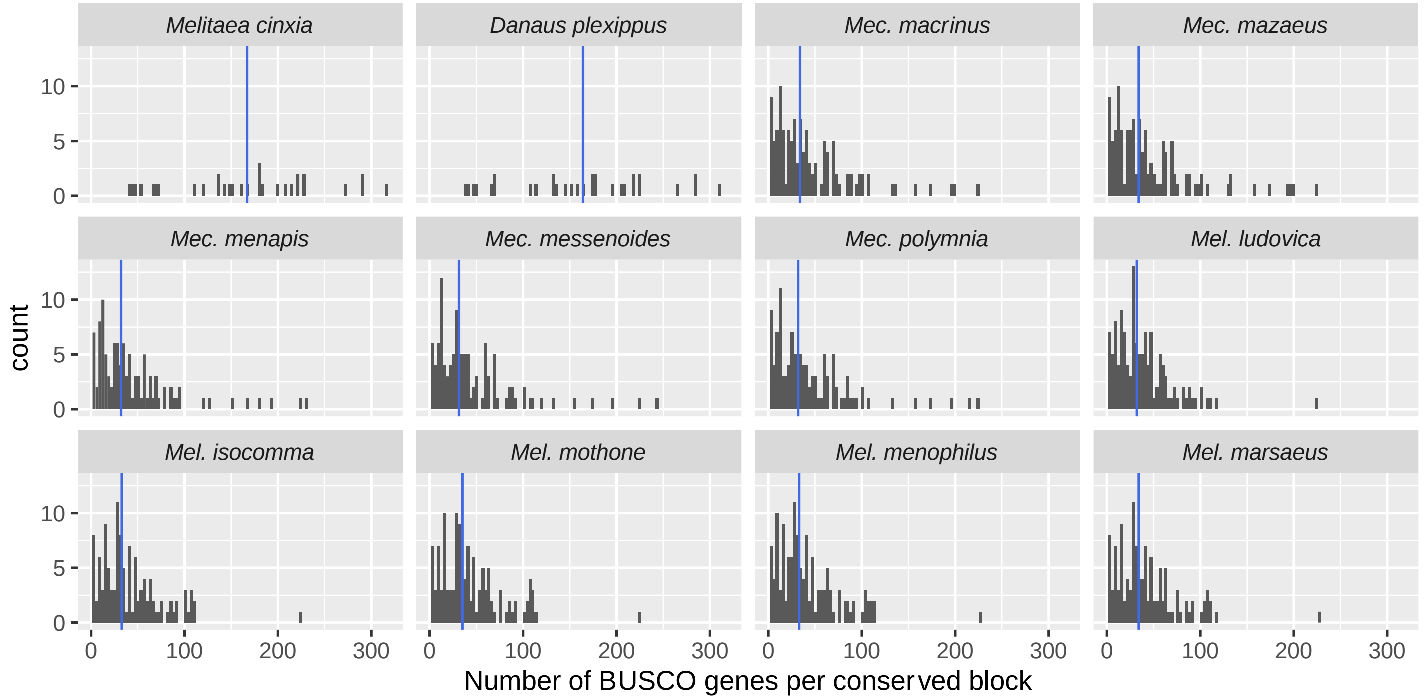

Fig. S21. Conserved syntenic blocks

Distribution of length of conserved syntenic blocks in number of BUSCO genes per block compared to the ancestral butterfly karyotype represented by *Melitaea cinxia*. Median number of genes per block (blue vertical line).

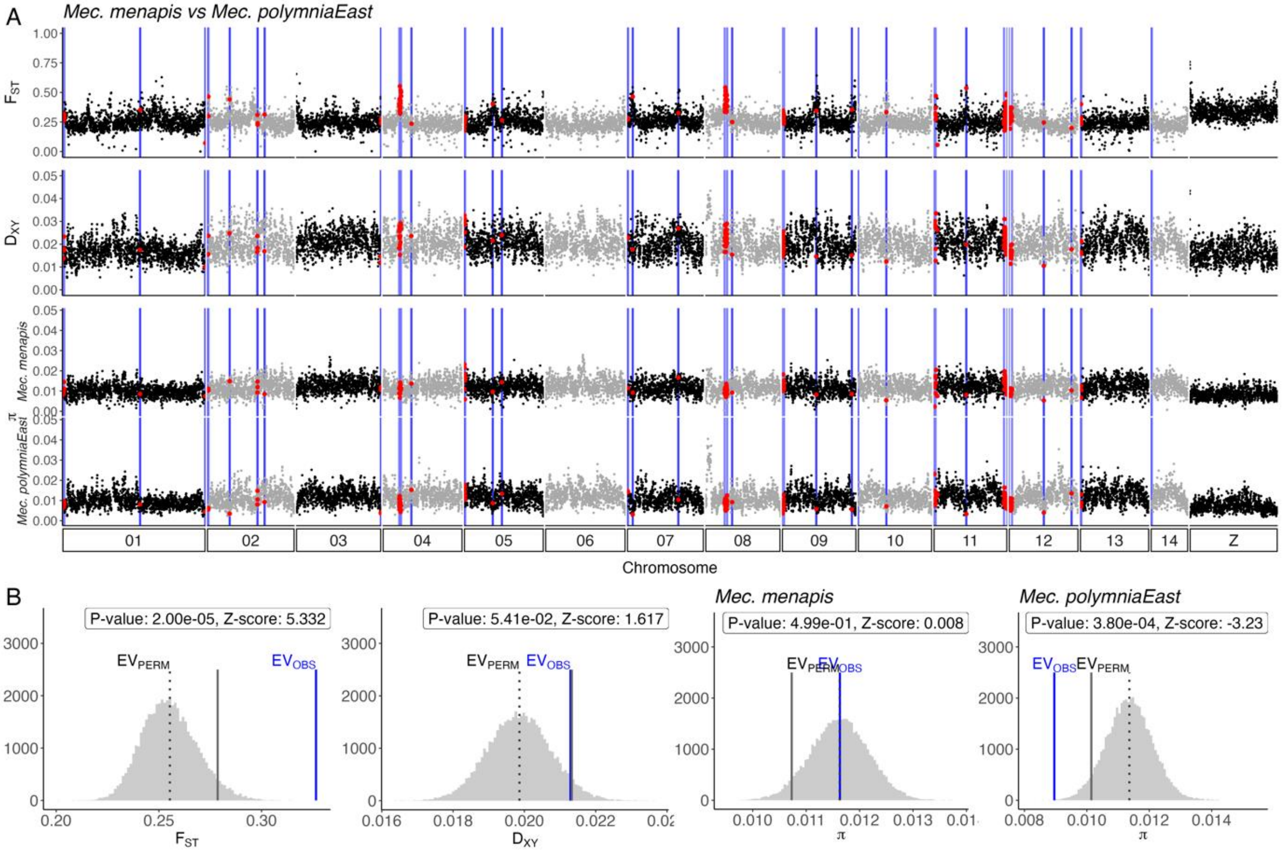
A
Mec. menapis vs Mec. polymniaEast
FST
DXY
π
Mec. menapis
Mec. polymniaEast
01 02 03 04 05 06 07 08 09 10 11 12 13 14 Z
Chromosome
B
P-value: 2.00e-05, Z-score: 5.332
EVPERM
EVOBS
FST
P-value: 5.41e-02, Z-score: 1.617
DXY
Mec. menapis
P-value: 4.99e-01, Z-score: 0.008
Mec. polymniaEast
P-value: 3.80e-04, Z-score: -3.23

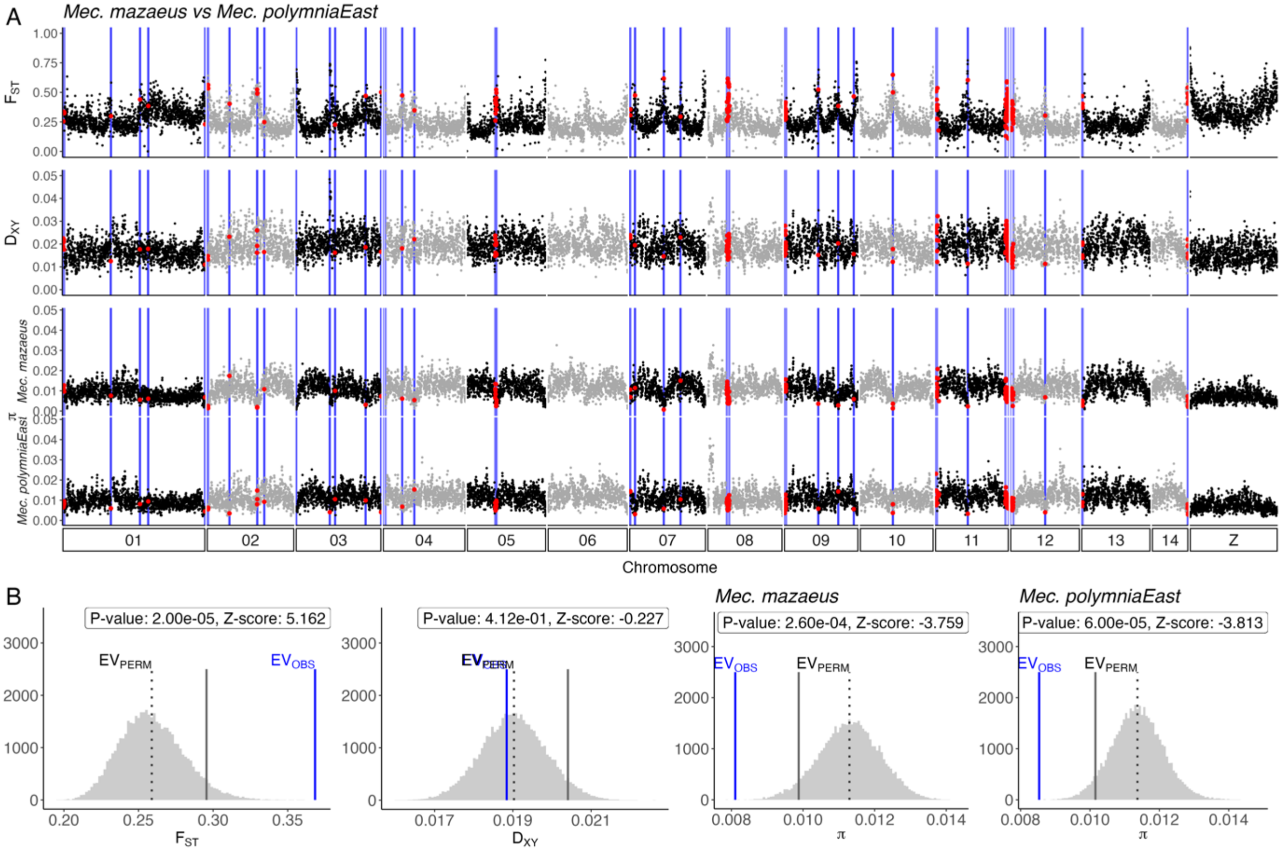
A
Mec. mazaeus vs Mec. polymniaEast
FST
DXY
π
Mec. mazaeus
Mec. polymniaEast
01 02 03 04 05 06 07 08 09 10 11 12 13 14 Z
Chromosome
B
P-value: 2.00e-05, Z-score: 5.162
EVPERM
EVOBS
FST
P-value: 4.12e-01, Z-score: -0.227
DXY
Mec. mazaeus
P-value: 2.60e-04, Z-score: -3.759
Mec. polymniaEast
P-value: 6.00e-05, Z-score: -3.813

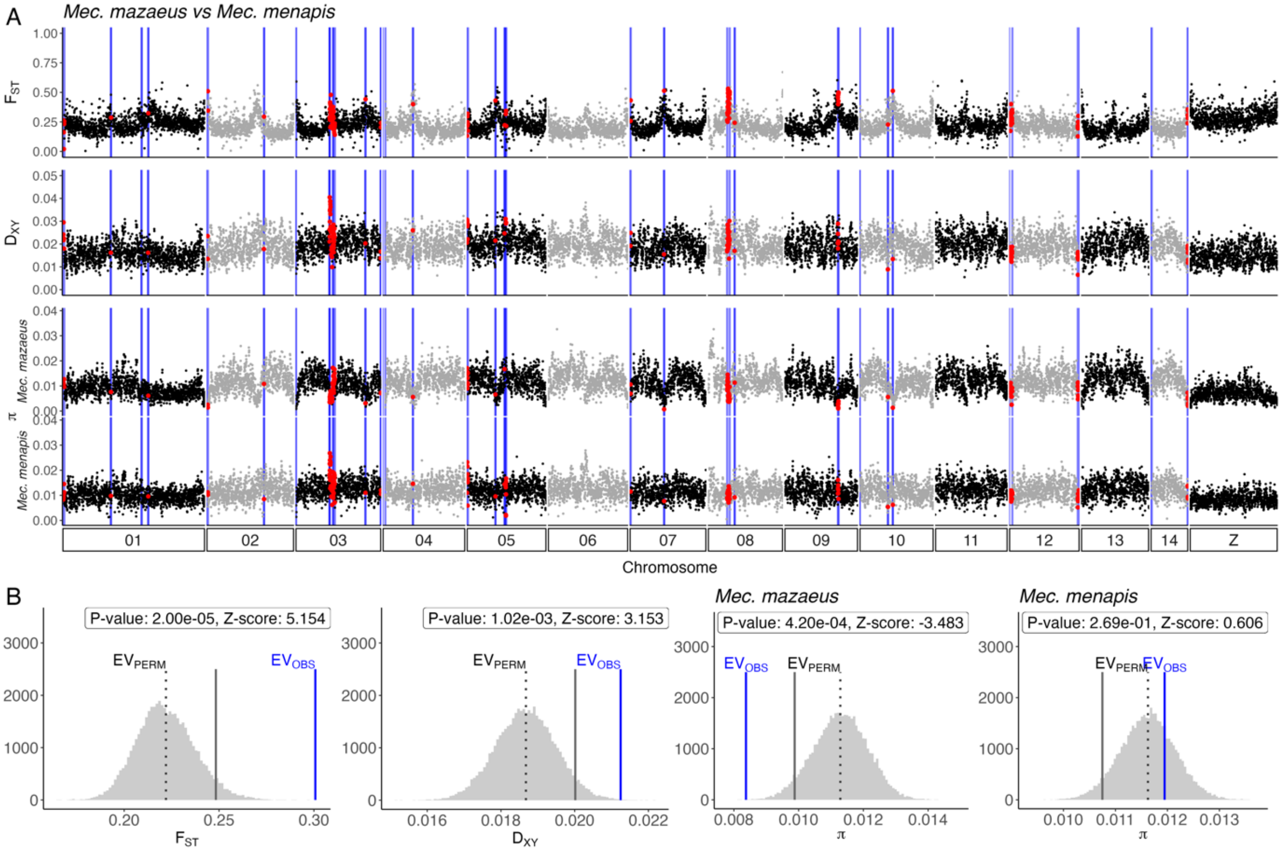
A
Mec. mazaeus vs Mec. menapis
FST
DXY
π
Mec. mazaeus
Mec. menapis
01 02 03 04 05 06 07 08 09 10 11 12 13 14 Z
Chromosome
B
Mec. mazaeus
Mec. menapis
P-value: 2.00e-05, Z-score: 5.154
P-value: 1.02e-03, Z-score: 3.153
P-value: 4.20e-04, Z-score: -3.483
P-value: 2.69e-01, Z-score: 0.606
EVPERM
EVOBS
FST
DXY
π

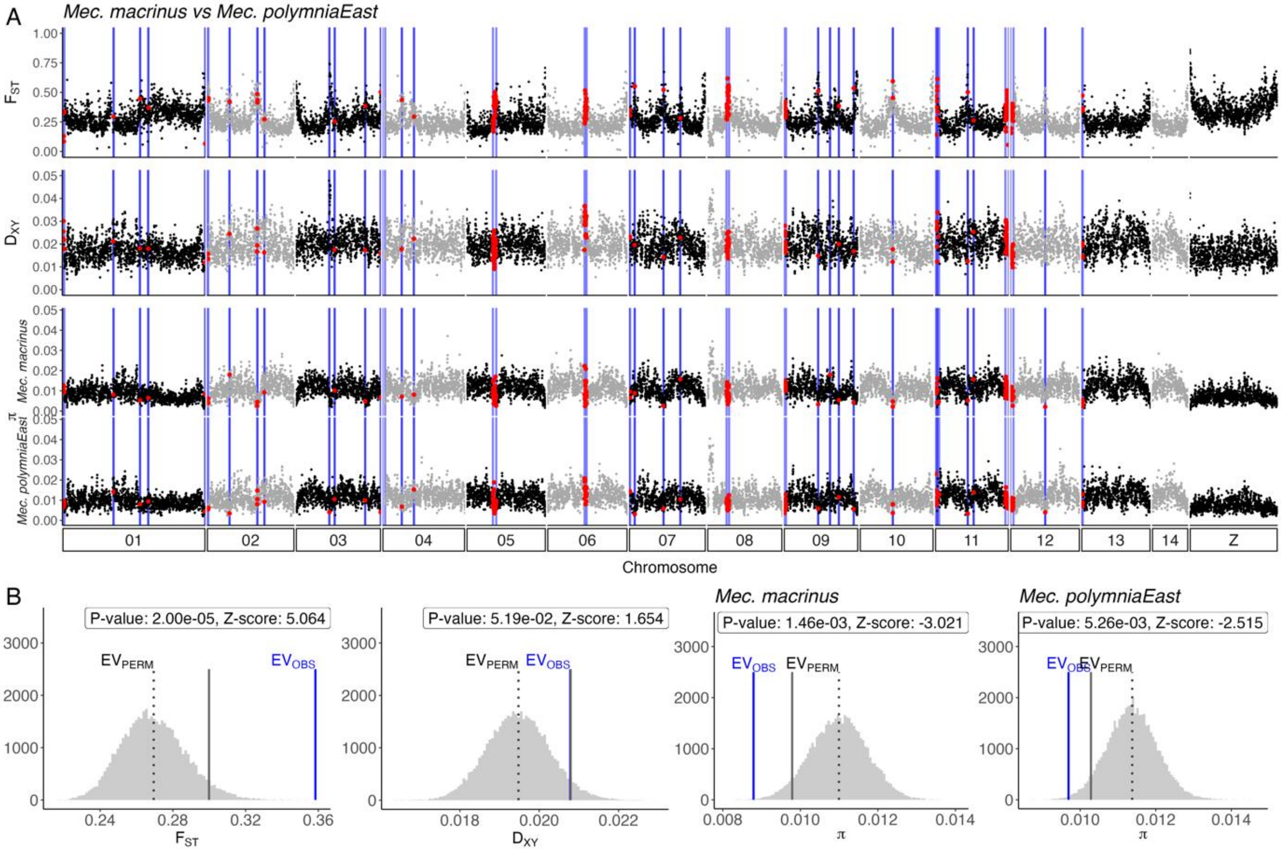
A
Mec. macrinus vs Mec. polymniaEast
FST
DXY
π
Mec. macrinus
Mec. polymniaEast
01 02 03 04 05 06 07 08 09 10 11 12 13 14 Z
Chromosome
B
Mec. macrinus
Mec. polymniaEast
P-value: 2.00e-05, Z-score: 5.064
P-value: 5.19e-02, Z-score: 1.654
P-value: 1.46e-03, Z-score: -3.021
P-value: 5.26e-03, Z-score: -2.515
EVPERM
EVOBS
FST
DXY
π

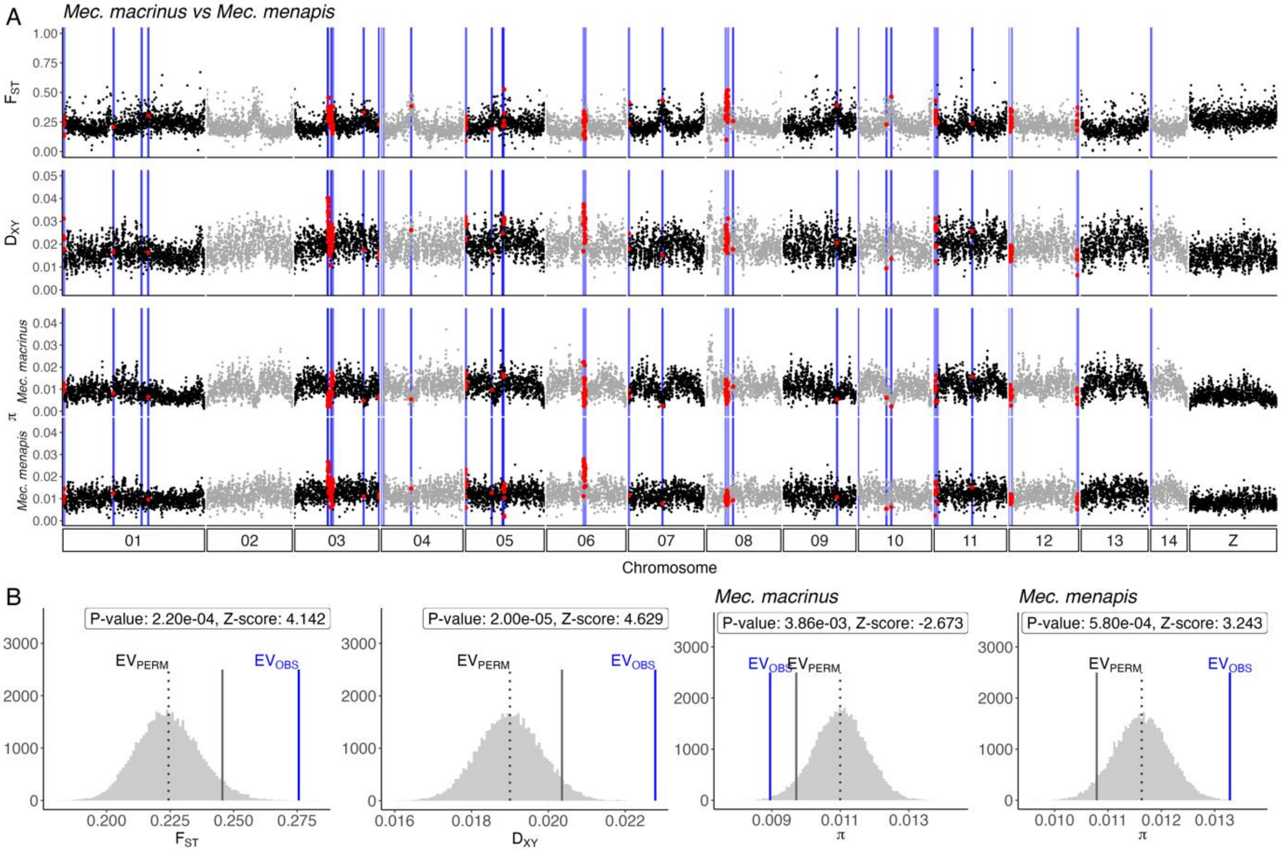
A
Mec. macrinus vs Mec. menapis
F_ST
D_XY
π
Mec. macrinus
Mec. menapis
01 02 03 04 05 06 07 08 09 10 11 12 13 14 Z
Chromosome
B
P-value: 2.20e-04, Z-score: 4.142
P-value: 2.00e-05, Z-score: 4.629
Mec. macrinus
P-value: 3.86e-03, Z-score: -2.673
Mec. menapis
P-value: 5.80e-04, Z-score: 3.243
EV_PERM
EV_OBS
F_ST
D_XY
π

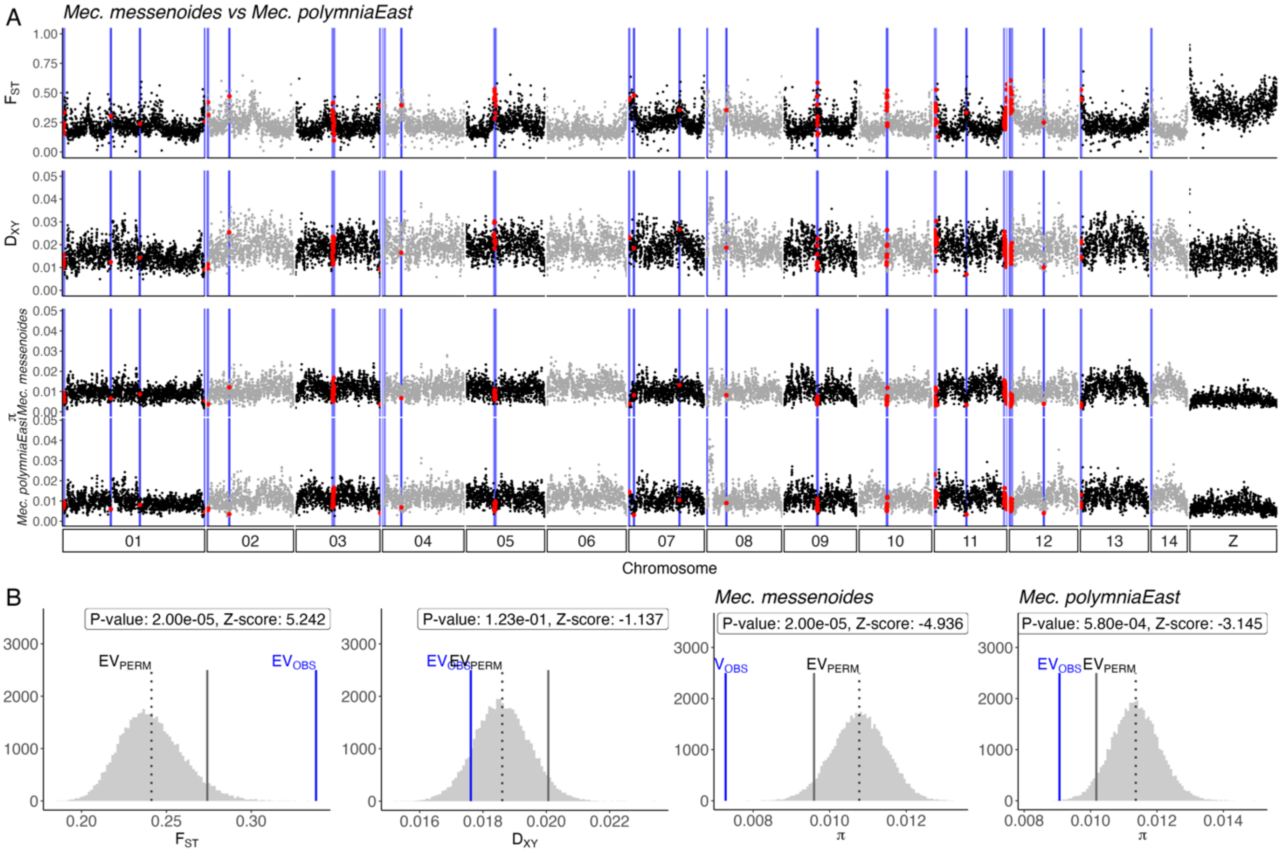
A
Mec. messenoides vs Mec. polymniaEast
F_ST
D_XY
π
Mec. messenoides
Mec. polymniaEast
01 02 03 04 05 06 07 08 09 10 11 12 13 14 Z
Chromosome
B
P-value: 2.00e-05, Z-score: 5.242
P-value: 1.23e-01, Z-score: -1.137
Mec. messenoides
P-value: 2.00e-05, Z-score: -4.936
Mec. polymniaEast
P-value: 5.80e-04, Z-score: -3.145
EV_PERM
EV_OBS
F_ST
D_XY
π

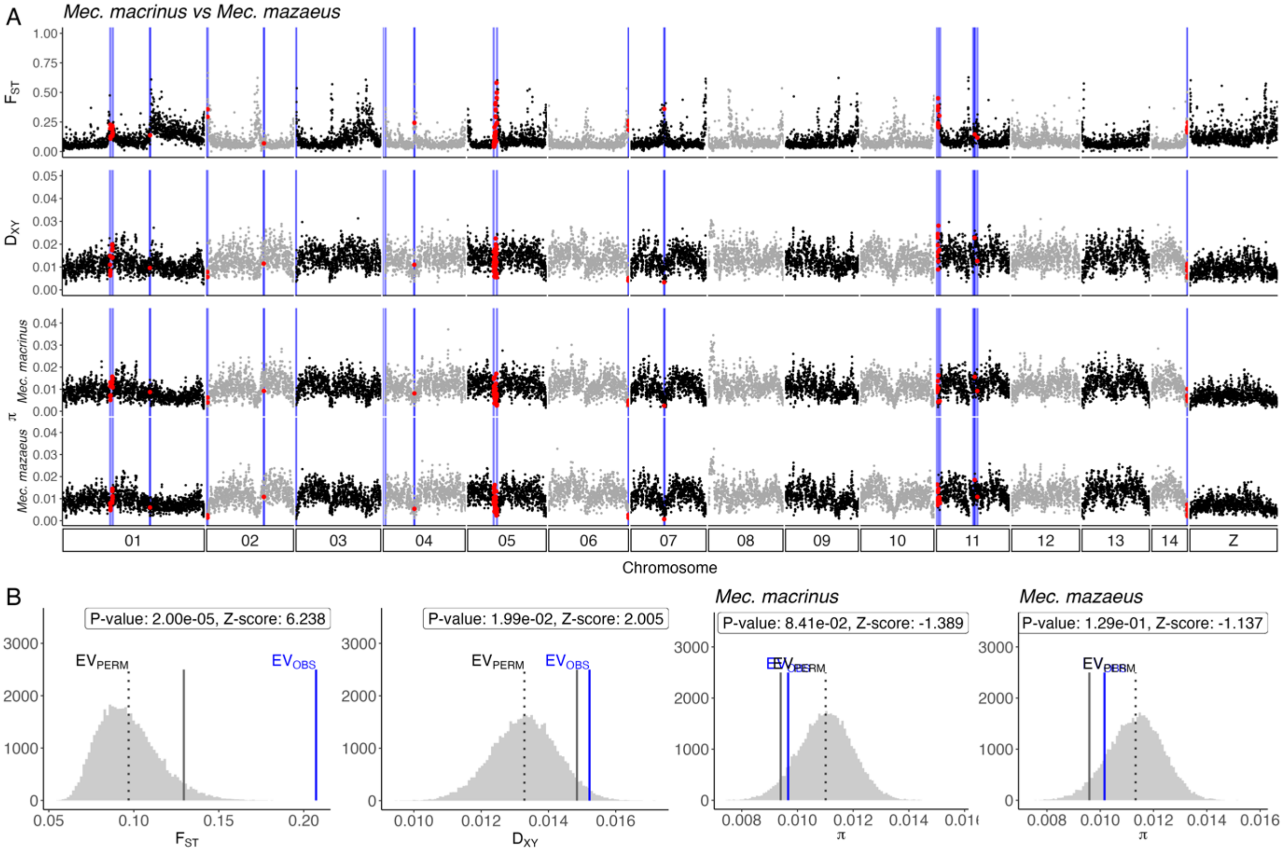
A
Mec. macrinus vs Mec. mazaeus
FST
DXY
π
Mec. macrinus
Mec. mazaeus
01
02
03
04
05
06
07
08
09
10
11
12
13
14
Z
Chromosome
B
P-value: 2.00e-05, Z-score: 6.238
EVPERM
EVOBS
FST
P-value: 1.99e-02, Z-score: 2.005
DXY
Mec. macrinus
P-value: 8.41e-02, Z-score: -1.389
Mec. mazaeus
P-value: 1.29e-01, Z-score: -1.137

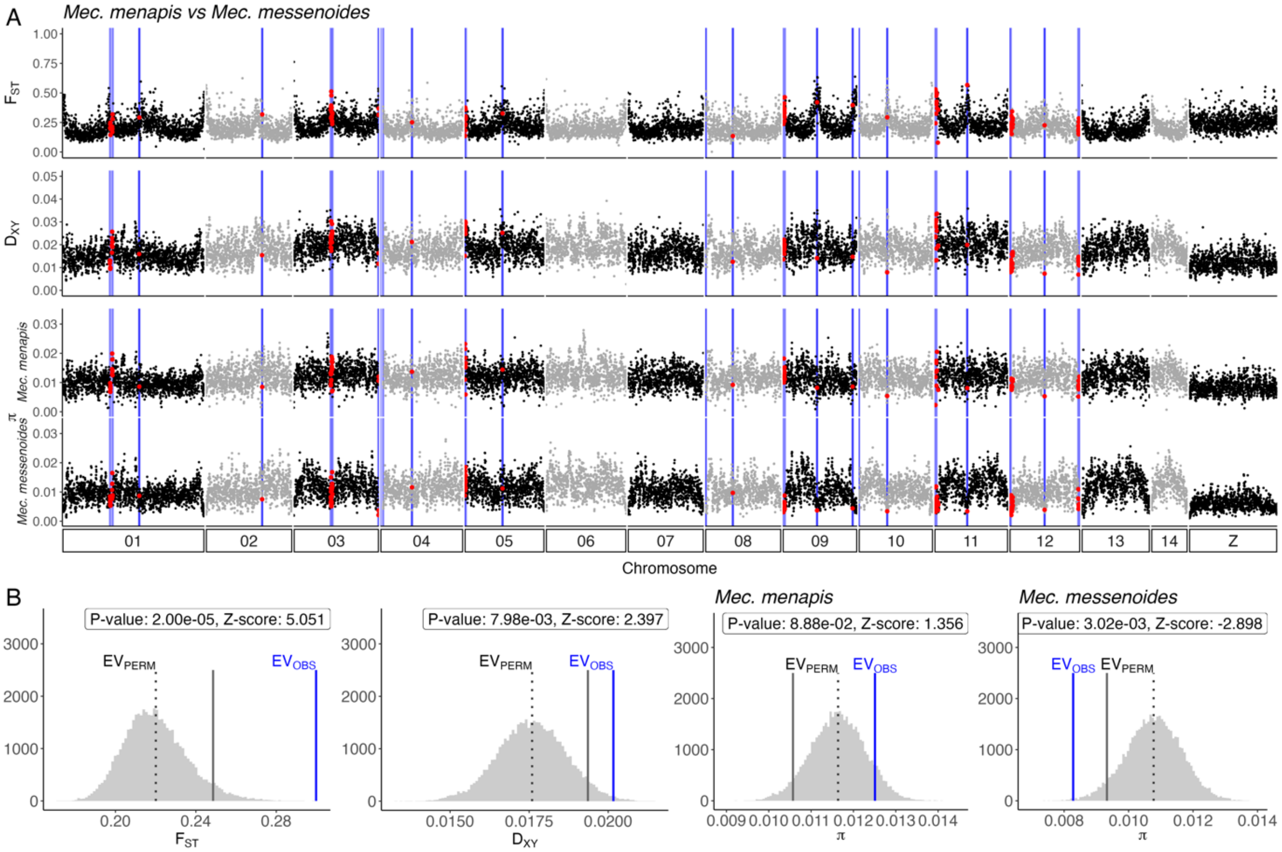
A
Mec. menapis vs Mec. messenoides
FST
DXY
π
Mec. menapis
Mec. messenoides
01
02
03
04
05
06
07
08
09
10
11
12
13
14
Z
Chromosome
B
P-value: 2.00e-05, Z-score: 5.051
EVPERM
EVOBS
FST
P-value: 7.98e-03, Z-score: 2.397
DXY
Mec. menapis
P-value: 8.88e-02, Z-score: 1.356
Mec. messenoides
P-value: 3.02e-03, Z-score: -2.898

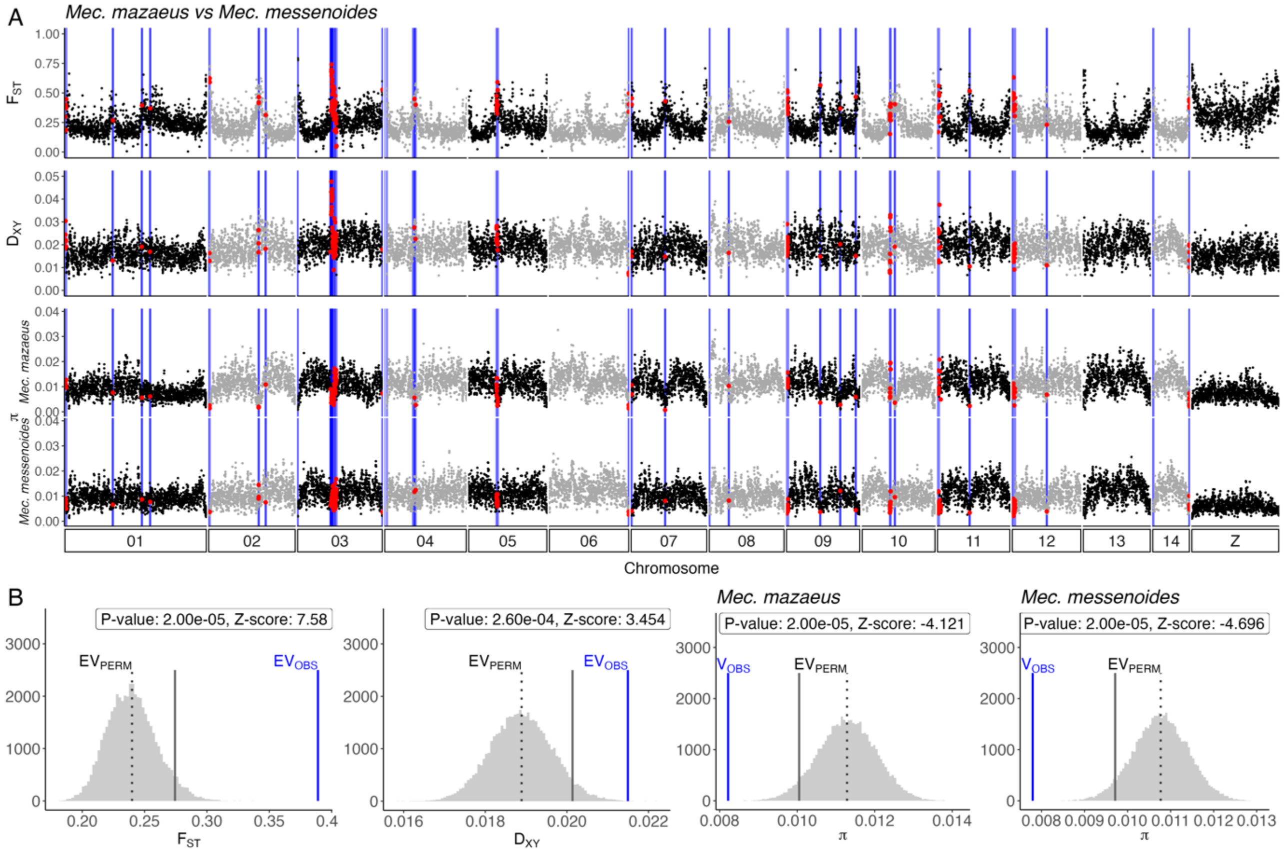
A
Mec. mazaeus vs Mec. messenoides
F_ST
D_XY
π
Mec. mazaeus
Mec. messenoides
01 02 03 04 05 06 07 08 09 10 11 12 13 14 Z
Chromosome
B
P-value: 2.00e-05, Z-score: 7.58
EV_PERM
EV_OBS
F_ST
P-value: 2.60e-04, Z-score: 3.454
EV_PERM
EV_OBS
D_XY
Mec. mazaeus
P-value: 2.00e-05, Z-score: -4.121
V_OBS
EV_PERM
π
Mec. messenoides
P-value: 2.00e-05, Z-score: -4.696
V_OBS
EV_PERM
π

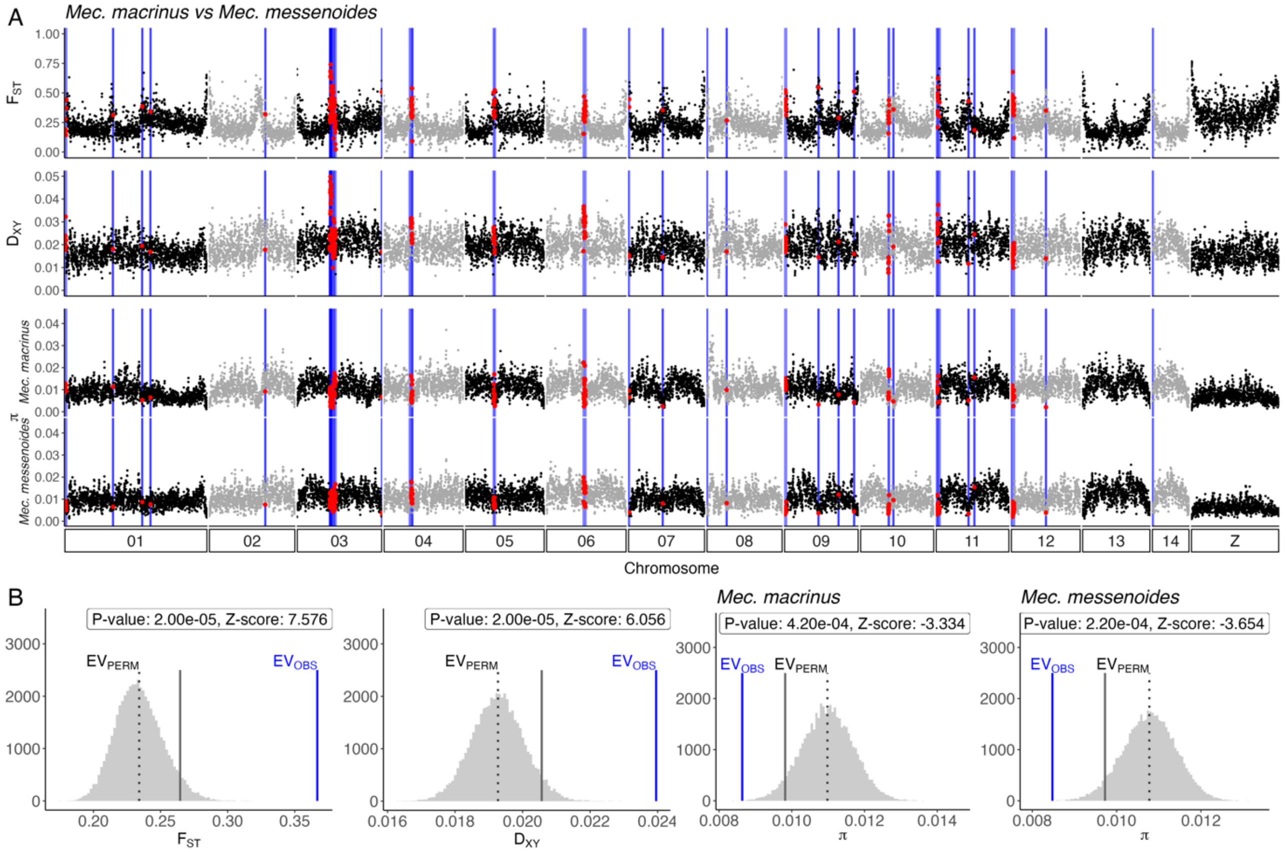
A
Mec. macrinus vs Mec. messenoides
F_ST
D_XY
π
Mec. macrinus
Mec. messenoides
01 02 03 04 05 06 07 08 09 10 11 12 13 14 Z
Chromosome
B
P-value: 2.00e-05, Z-score: 7.576
EV_PERM
EV_OBS
F_ST
P-value: 2.00e-05, Z-score: 6.056
EV_PERM
EV_OBS
D_XY
Mec. macrinus
P-value: 4.20e-04, Z-score: -3.334
EV_OBS
EV_PERM
π
Mec. messenoides
P-value: 2.20e-04, Z-score: -3.654
EV_OBS
EV_PERM
π

Fig. S22. Genome scans with breakpoints for *Mechanitis*

For each species-pair comparison: ***A****) Population genomics statistics per 20kb window across the genome for* F_{ST}, D_{XY} *for each species comparison and* π *for each species within* Mechanitis*. Grey and black dots are coloured by chromosome and red dots represent windows located in breakpoints.* ***B****) Randomisation test for each statistic across the genome with P-value at 5% significance level (lesser than or greater than randomised sample, grey solid line represents the threshold for the primary alternative hypothesis). Z-score in label. Mean observed population statistic within breakpoint regions (blue,* EV_{OBS}*) and permutation mean (dotted grey line,* EV_{PERM}*) for* F_{ST}, D_{XY} *and* π*.*

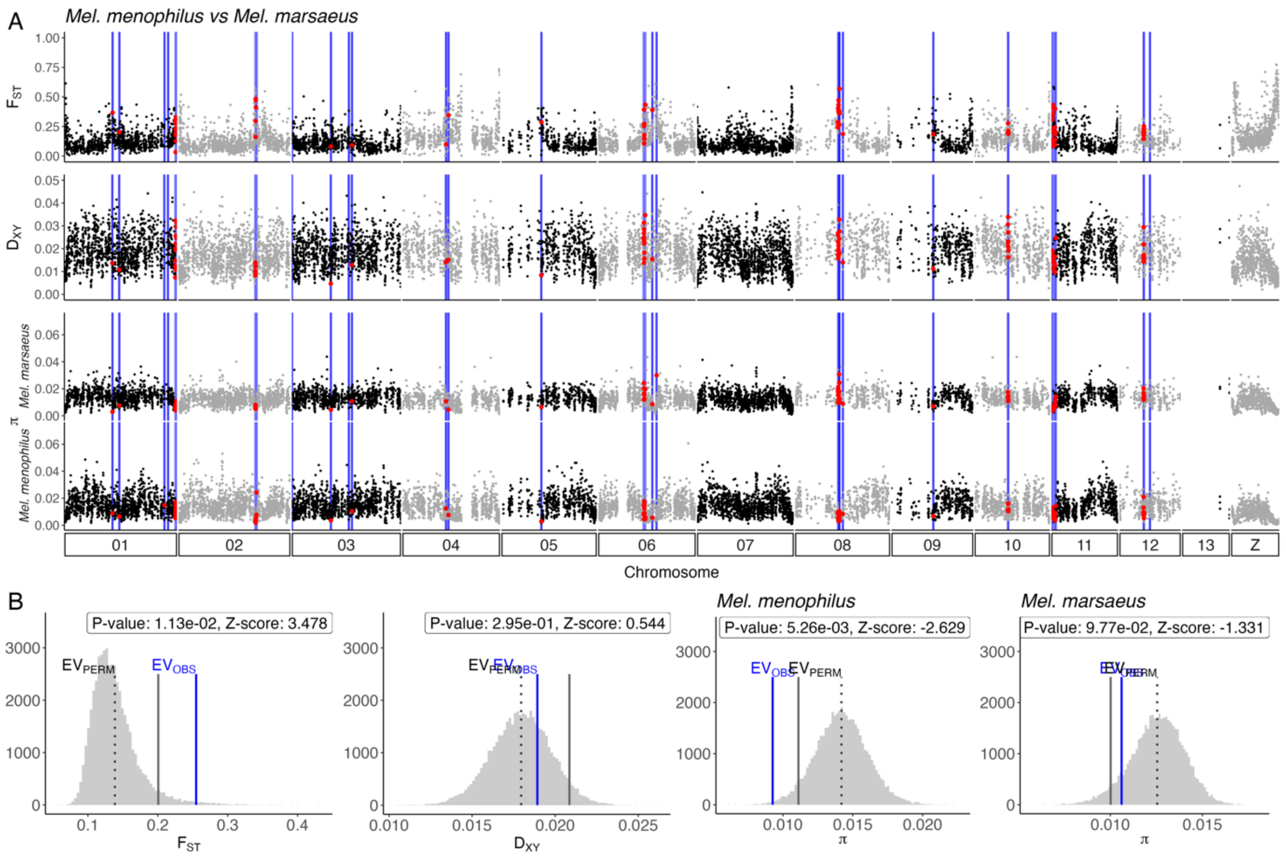
A
Mel. menophilus vs Mel. marsaeus
F_ST
D_XY
π
Mel. marsaeus
Mel. menophilus
01 02 03 04 05 06 07 08 09 10 11 12 13 Z
Chromosome
B
P-value: 1.13e-02, Z-score: 3.478
P-value: 2.95e-01, Z-score: 0.544
Mel. menophilus
P-value: 5.26e-03, Z-score: -2.629
Mel. marsaeus
P-value: 9.77e-02, Z-score: -1.331
EV_PERM
EV_OBS
F_ST
D_XY
π

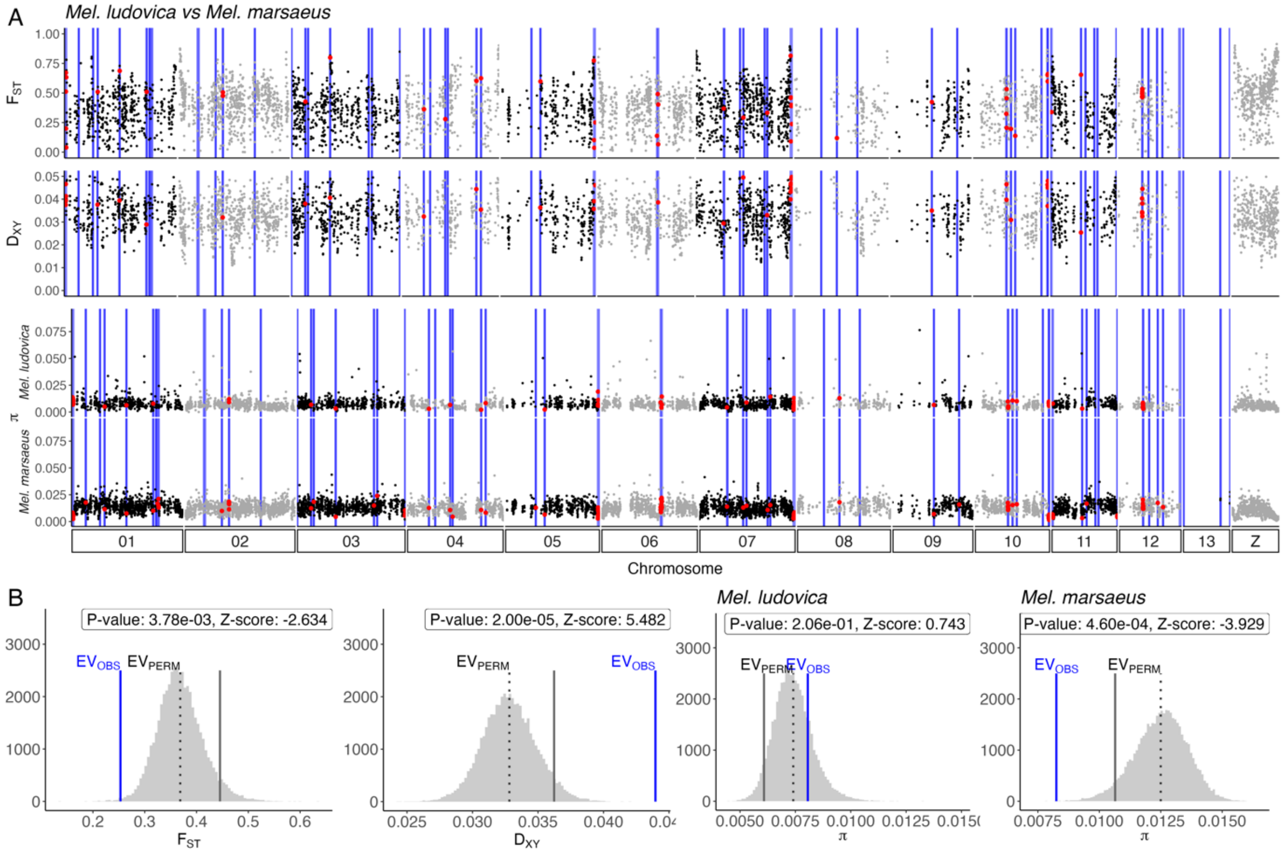
A
Mel. ludovica vs Mel. marsaeus
F_ST
D_XY
π
Mel. ludovica
Mel. marsaeus
01 02 03 04 05 06 07 08 09 10 11 12 13 Z
Chromosome
B
P-value: 3.78e-03, Z-score: -2.634
P-value: 2.00e-05, Z-score: 5.482
Mel. ludovica
P-value: 2.06e-01, Z-score: 0.743
Mel. marsaeus
P-value: 4.60e-04, Z-score: -3.929
EV_OBS
EV_PERM
F_ST
D_XY
π

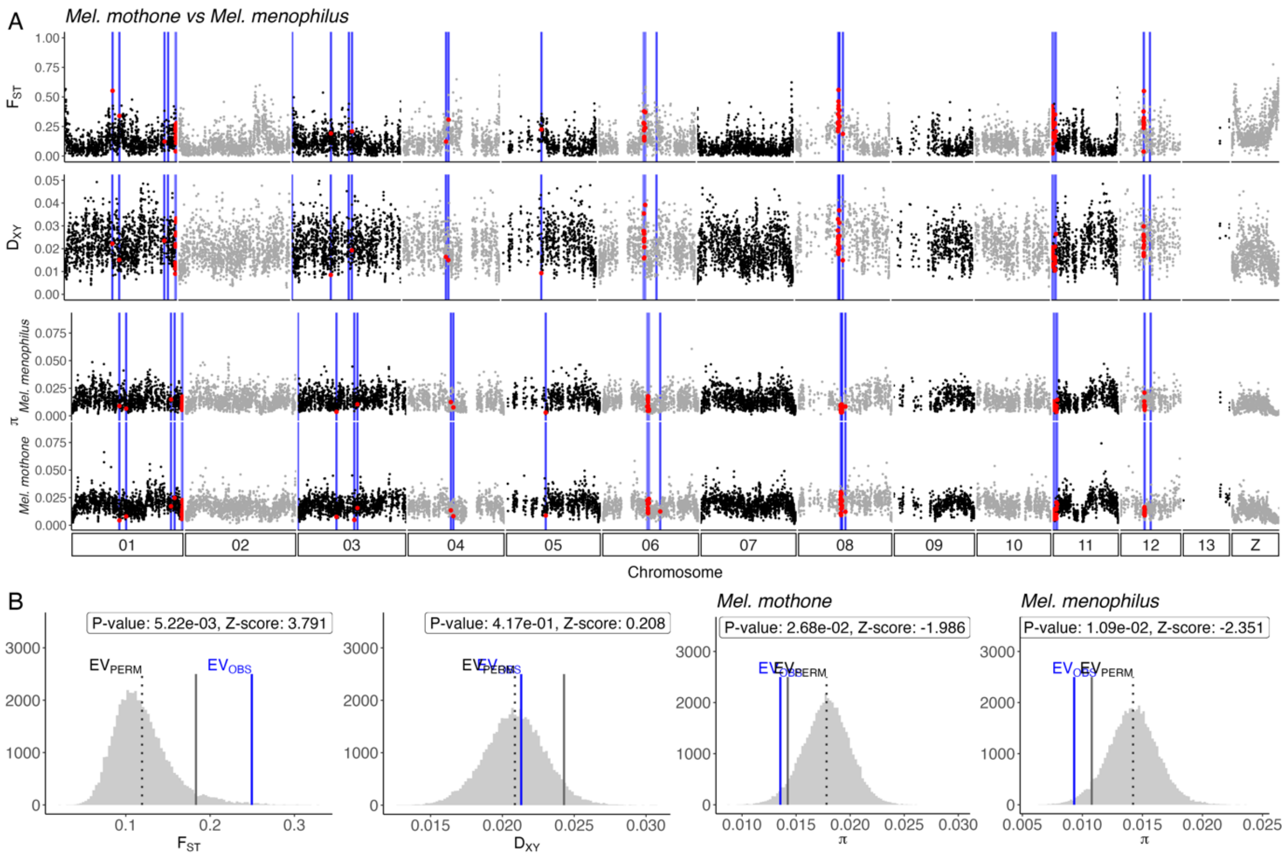
A
Mel. mothone vs Mel. menophilus
F_ST
D_XY
π
Mel. menophilus
Mel. mothone
01
02
03
04
05
06
07
08
09
10
11
12
13
Z
Chromosome
B
P-value: 5.22e-03, Z-score: 3.791
EV_PERM
EV_OBS
F_ST
P-value: 4.17e-01, Z-score: 0.208
D_XY
Mel. mothone
P-value: 2.68e-02, Z-score: -1.986
Mel. menophilus
P-value: 1.09e-02, Z-score: -2.351
π

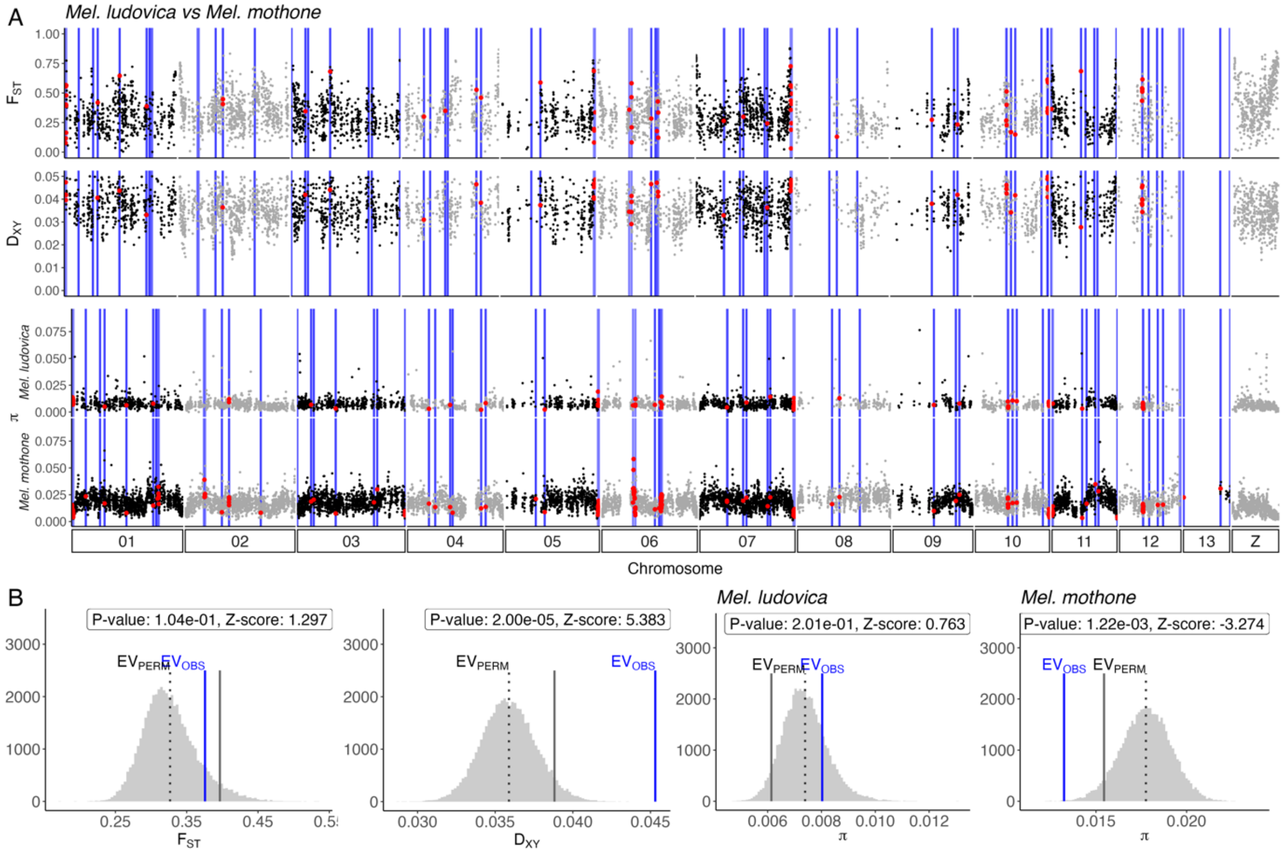
A
Mel. ludovica vs Mel. mothone
F_ST
D_XY
π
Mel. ludovica
Mel. mothone
01
02
03
04
05
06
07
08
09
10
11
12
13
Z
Chromosome
B
P-value: 1.04e-01, Z-score: 1.297
EV_PERM
EV_OBS
F_ST
P-value: 2.00e-05, Z-score: 5.383
D_XY
Mel. ludovica
P-value: 2.01e-01, Z-score: 0.763
Mel. mothone
P-value: 1.22e-03, Z-score: -3.274
π

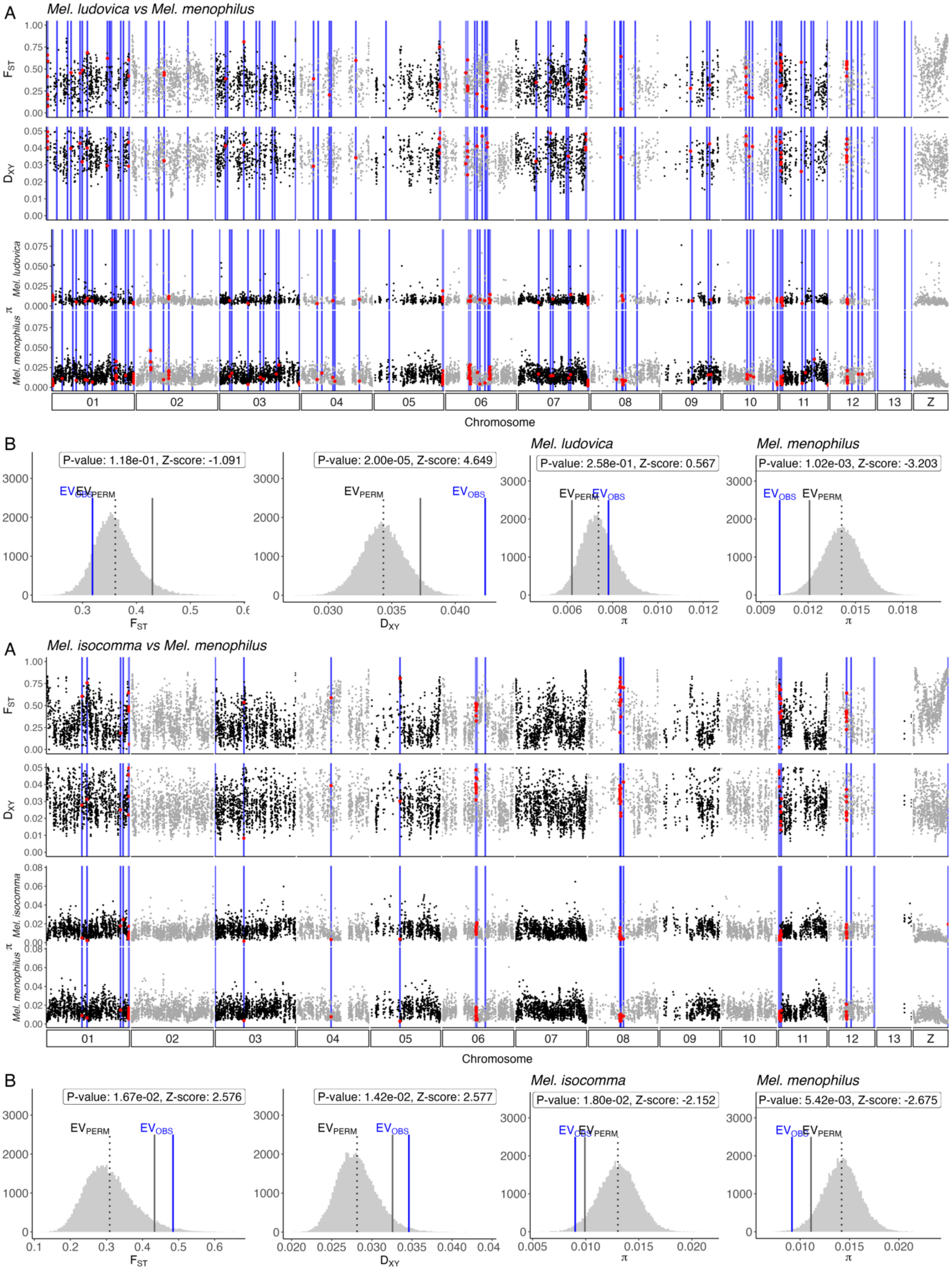
A
Mel. ludovica vs Mel. menophilus
FST
DXY
Mel. ludovica
π
Mel. menophilus
01
02
03
04
05
06
07
08
09
10
11
12
13
Z
Chromosome
B
P-value: 1.18e-01, Z-score: -1.091
P-value: 2.00e-05, Z-score: 4.649
Mel. ludovica
P-value: 2.58e-01, Z-score: 0.567
Mel. menophilus
P-value: 1.02e-03, Z-score: -3.203
EVOBS
EVPERM
A
Mel. isocomma vs Mel. menophilus
Mel. isocomma
B
P-value: 1.67e-02, Z-score: 2.576
P-value: 1.42e-02, Z-score: 2.577
P-value: 1.80e-02, Z-score: -2.152
P-value: 5.42e-03, Z-score: -2.675

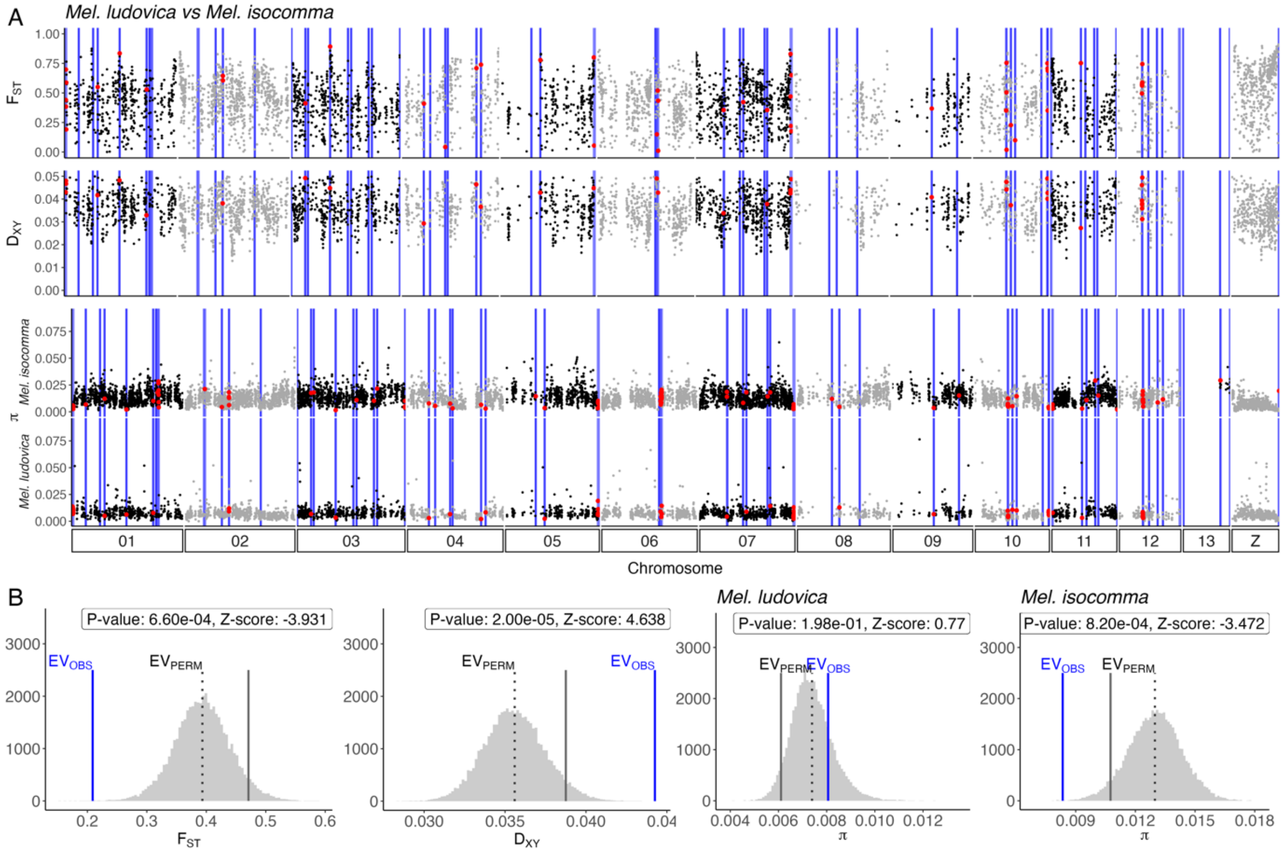
A
Mel. ludovica vs Mel. isocomma
FST
DXY
π
Mel. isocomma
Mel. ludovica
01 02 03 04 05 06 07 08 09 10 11 12 13 Z
Chromosome
B
P-value: 6.60e-04, Z-score: -3.931
EVOBS
EVPERM
FST
P-value: 2.00e-05, Z-score: 4.638
EVPERM
EVOBS
DXY
Mel. ludovica
P-value: 1.98e-01, Z-score: 0.77
EVPERM
EVOBS
π
Mel. isocomma
P-value: 8.20e-04, Z-score: -3.472
EVOBS
EVPERM
π

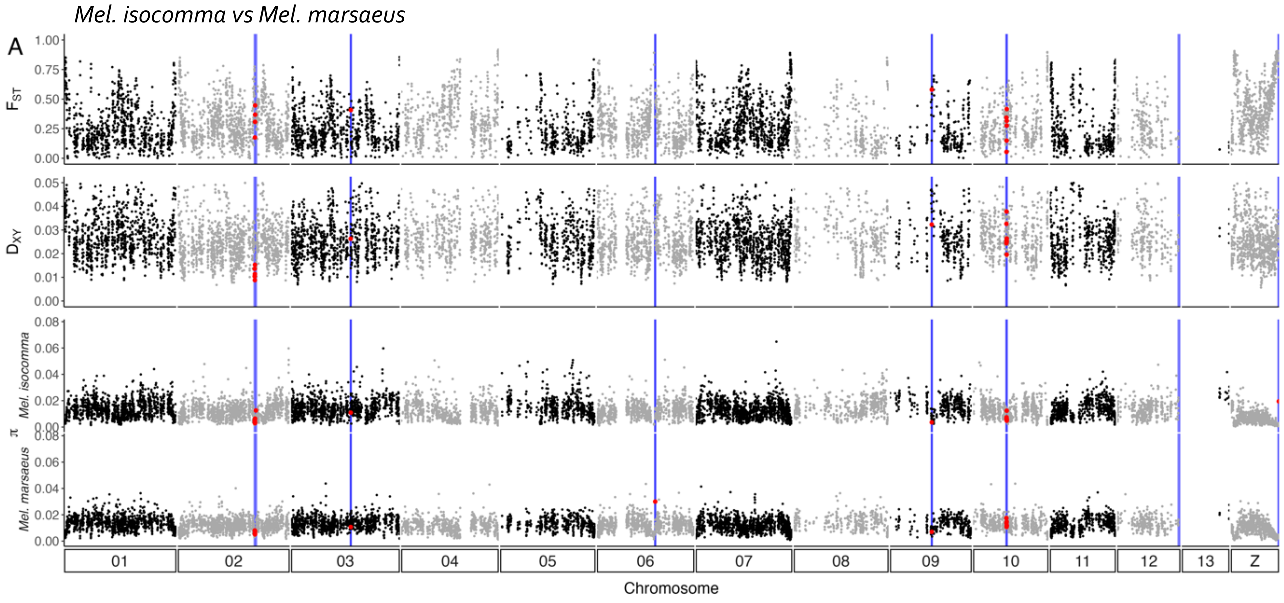
Mel. isocomma vs Mel. marsaeus
A
FST
DXY
π
Mel. isocomma
Mel. marsaeus
01 02 03 04 05 06 07 08 09 10 11 12 13 Z
Chromosome

Mel. isocomma vs Mel. mothone

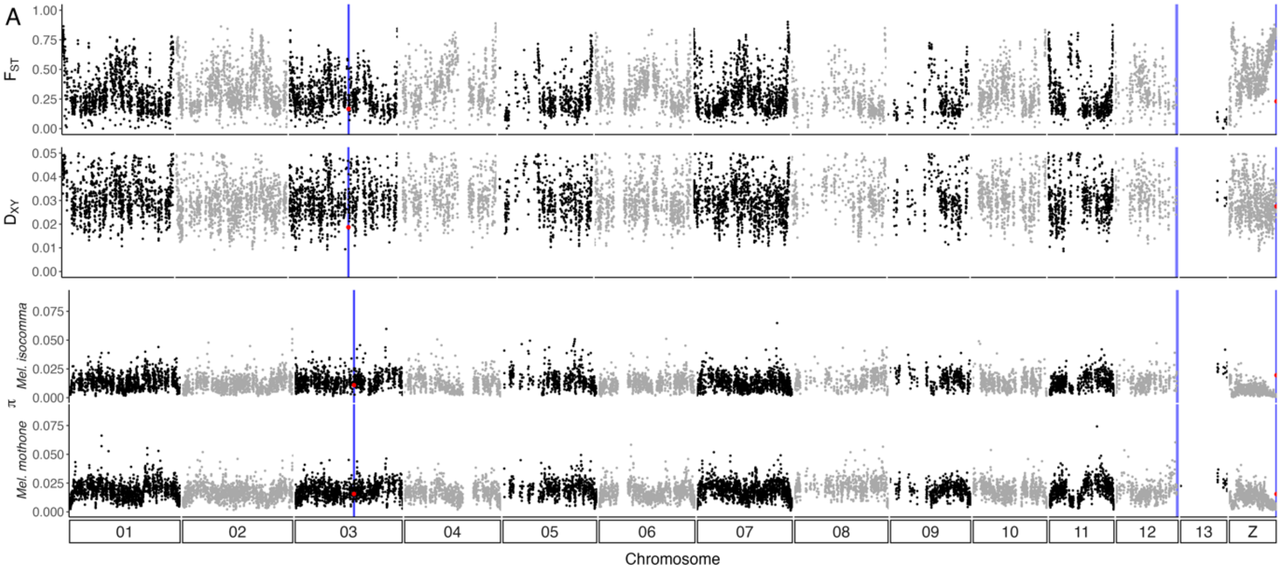

Mel. mothone vs Mel. marsaeus

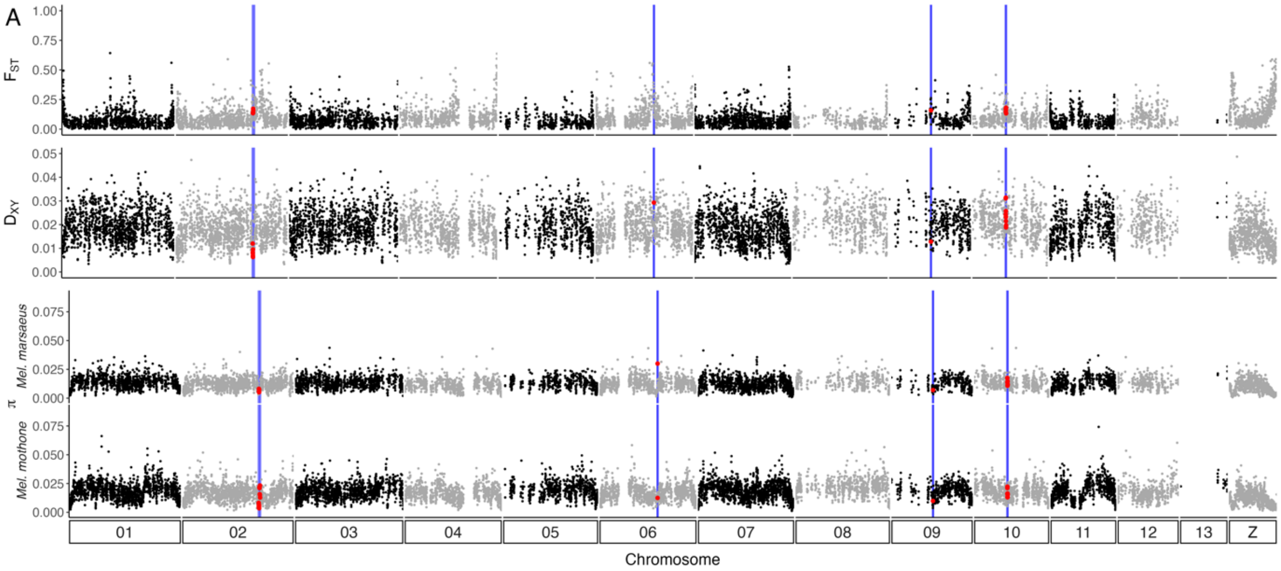

Fig. S23. Genome scans with breakpoints for *Melinaea*

*For each species-pair comparison: **A**) Population genomics statistics per 20kb window across the genome for F_{ST}, D_{XY} for each species comparison and π for each species within* Melinaea*. Grey and black dots are coloured by chromosome and red dots represent windows located in breakpoints. **B**) Randomisation test for each statistic across the genome with P-value at 5% significance level (lesser than or greater than randomised sample, grey solid line represents the threshold for the primary alternative hypothesis). Z-score in label. Mean observed population statistic within breakpoint regions (blue, EV_{OBS}) and permutation mean (dotted grey line, EV_{PERM}) for F_{ST}, D_{XY} and π.*

Table S1: Sequenced individuals. Information on all individuals resequenced with Illumina.

sex without asterisk: based on photos of hindwings (with bristles = male; without = female)

sex with asterisk: based on W and Z chromosome depth compared to autosome depth (for Mechanitis: W close to 0 and Z equal to autosome = male; W > 0 (usually half of depth Z) and Z half of autosome = female; for Melinaea: W < autosome and Z > autosome = male; W > autosome and Z < autosome = female. NOTE: take with some caution, there is a lot of variety)

ID (sequencing)	Original ID	full name (before reclassification)	full name (reclassification)	Dsuite	Phylogeny	Country	Year	latitude	longitude	Sex	Location	Elevation	Depth	study	Accession
CAM028090	CAM028090	*Forbestra equicola equicoloides*	*For. equicola equiloides*	Outgroup (Mec.)	include	Ecuador	2019	-1.1155667	-77.77833	F*	Apuya km2.7, Napo	551	8.39578	PRJEB87706	ERS23920414
CAM028271	CAM028271	*Forbestra equicola equicoloides*	*For. equicola equiloides*	Outgroup (Mec.)	include	Ecuador	2019	-0.9484307	-77.59437	M*	Rio Pusuno, Napo	835.795	8.54284	PRJEB87706	ERS23920415
CAM028327	CAM028327	*Forbestra equicola equicoloides*	*For. equicola equiloides*	Outgroup (Mec.)	include	Ecuador	2019	NA	NA	M*	Yutzupino 6.3 km, Napo	NA	8.28167	PRJEB87706	ERS23920416
CAM028193	CAM028193	*Forbestra olivencia juntana*	*For. olivencia juntana*	Outgroup (Mec.)	include	Ecuador	2019	-0.9484307	-77.59437	M*	Rio Pusuno, Napo	835.795	8.40125	PRJEB87706	ERS23920417
CAM028312	CAM028312	*Forbestra olivencia juntana*	*For. olivencia juntana*	Outgroup (Mec.)	include	Ecuador	2019	-0.9484307	-77.59437	F*	Rio Pusuno, Napo	835.795	8.32118	PRJEB87706	ERS23920418
CAM070582	CAM070582	*Forbestra olivencia juntana*	*For. olivencia juntana*	Outgroup (Mec.)	include	Ecuador	2022	-1.1155667	-77.77833	M	Apuya km2.7, Napo	551	24.5779	PRJEB63068	SAMEA114393970
CAM070479	CAM070479	*Forbestra proceris*	*For. proceris*	Outgroup (Mec.)	include	Ecuador	2022	-1.09759	-77.58389	F	San Pedro de Arajuno	374	21.0066	PRJEB63068	SAMEA114394218
CAM070480	CAM070480	*Forbestra proceris*	*For. proceris*	Outgroup (Mec.)	include	Ecuador	2022	-1.09759	-77.58389	F	San Pedro de Arajuno	374	18.6076	PRJEB63068	SAMEA114394219
CAM070483	CAM070483	*Forbestra proceris*	*For. proceris*	Outgroup (Mec.)	include	Ecuador	2022	-1.09759	-77.58389	M	San Pedro de Arajuno	374	18.8403	PRJEB63068	SAMEA114394220
CAM036283	CAM036283	*Mechanitis lysimnia lysimnia*	*Mec. lysimnia lysimnia*	lysimniaBraz	exclude	Brazil	2020	-15.406724	-39.54846	F	Serra Bonita	287.456	9.41379	PRJEB87706	ERS23920419
CAM036340	CAM036340	*Mechanitis lysimnia lysimnia*	*Mec. lysimnia lysimnia*	lysimniaBraz	exclude	Brazil	2020	-15.406724	-39.54846	M	Serra Bonita	287.456	11.7506	PRJEB87706	ERS23920420
CAM036460	CAM036460	*Mechanitis lysimnia lysimnia*	*Mec. lysimnia lysimnia*	lysimniaBraz	exclude	Brazil	2020	-22.437925	-44.61271	F	Itatiaia	1003.061	10.7128	PRJEB87706	ERS23920421
CAM036461	CAM036461	*Mechanitis lysimnia lysimnia*	*Mec. lysimnia lysimnia*	lysimniaBraz	exclude	Brazil	2020	-22.437925	-44.61271	F	Itatiaia	1003.061	12.1488	PRJEB87706	ERS23920422
CAM036544	CAM036544	*Mechanitis lysimnia lysimnia*	*Mec. lysimnia lysimnia*	lysimniaBraz	exclude	Brazil	2020	-23.230809	-46.92723	M	Japi	924.675	9.71835	PRJEB87706	ERS23920423
CAM036548	CAM036548	*Mechanitis lysimnia lysimnia*	*Mec. lysimnia lysimnia*	lysimniaBraz	exclude	Brazil	2020	-23.230809	-46.92723	M	Japi	924.675	8.97937	PRJEB87706	ERS23920424
LBR0211	LBR0211	*Mechanitis lysimnia lysimnia*	*Mec. lysimnia lysimnia*	lysimniaBraz	exclude	Brazil	2011	-22.656793	-52.85771	M*	Parana, Diamante do Norte	340.515	5.83743	PRJEB87706	ERS23920425
MCN001	MCN001	*Mechanitis lysimnia lysimnia*	*Mec. lysimnia lysimnia*	lysimniaBraz	exclude	Brazil	2023	NA	NA	F*	Bacchus, RJ	NA	13.8941	PRJEB87706	ERS23920426
MCN002	MCN002	*Mechanitis lysimnia lysimnia*	*Mec. lysimnia lysimnia*	lysimniaBraz	exclude	Brazil	2023	NA	NA	F*	Bacchus, RJ	NA	14.5659	PRJEB87706	ERS23920427
MCN003	MCN003	*Mechanitis lysimnia lysimnia*	*Mec. lysimnia lysimnia*	lysimniaBraz	include	Brazil	2023	NA	NA	F*	Bacchus, RJ	NA	15.7072	PRJEB87706	ERS23920428
MCN004	MCN004	*Mechanitis lysimnia lysimnia*	*Mec. lysimnia lysimnia*	lysimniaBraz	exclude	Brazil	2023	NA	NA	F*	Bacchus, RJ	NA	14.1557	PRJEB87706	ERS23920429
MCN005	MCN005	*Mechanitis lysimnia lysimnia*	*Mec. lysimnia lysimnia*	lysimniaBraz	exclude	Brazil	2023	NA	NA	M*	Bacchus, RJ	NA	13.1734	PRJEB87706	ERS23920430
MCN006	MCN006	*Mechanitis lysimnia lysimnia*	*Mec. lysimnia lysimnia*	lysimniaBraz	exclude	Brazil	2023	NA	NA	F*	Bacchus, RJ	NA	14.9662	PRJEB87706	ERS23920431
MCN007	MCN007	*Mechanitis lysimnia lysimnia*	*Mec. lysimnia lysimnia*	lysimniaBraz	exclude	Brazil	NA	NA	NA	F*	Camcan, BA	NA	13.7085	PRJEB87706	ERS23920432
MCN008	MCN008	*Mechanitis lysimnia lysimnia*	*Mec. lysimnia lysimnia*	lysimniaBraz	include	Brazil	2015	NA	NA	F*	Camcan, BA	NA	15.3574	PRJEB87706	ERS23920433
MCN009	MCN009	*Mechanitis lysimnia lysimnia*	*Mec. lysimnia lysimnia*	lysimniaBraz	include	Brazil	2021	-15.361908	-39.57668	F*	Serra Bonita	551.573	17.1437	PRJEB87706	ERS23920434
MCN010	MCN010	*Mechanitis lysimnia lysimnia*	*Mec. lysimnia lysimnia*	lysimniaBraz	include	Brazil	2021	-15.361908	-39.57668	M*	Serra Bonita	551.573	17.4601	PRJEB87706	ERS23920435
MCN012	MCN012	*Mechanitis lysimnia lysimnia*	*Mec. lysimnia lysimnia*	lysimniaBraz	include	Brazil	2019	-24.319406	-46.99659	M*	Peruibe, SP	8.861	18.361	PRJEB87706	ERS23920436
MCN014	MCN014	*Mechanitis lysimnia lysimnia*	*Mec. lysimnia lysimnia*	lysimniaBraz	include	Brazil	2019	-24.319406	-46.99659	F*	Peruibe, SP	8.861	15.6518	PRJEB87706	ERS23920437
CAM028192	CAM028192	*Mechanitis lysimnia roqueensis*	*Mec. lysimnia roqueensis*	lysimniaEcu	include	Ecuador	2019	-0.9484307	-77.59437	M*	Rio Pusuno, Napo	835.795	9.14504	PRJEB87706	ERS23920438
CAM028197	CAM028197	*Mechanitis lysimnia roqueensis*	*Mec. lysimnia roqueensis*	lysimniaEcu	include	Ecuador	2019	-0.9484307	-77.59437	M*	Rio Pusuno, Napo	835.795	8.15159	PRJEB87706	ERS23920439
CAM028198	CAM028198	*Mechanitis lysimnia roqueensis*	*Mec. lysimnia roqueensis*	lysimniaEcu	include	Ecuador	2019	-0.9484307	-77.59437	M*	Rio Pusuno, Napo	835.795	6.75433	PRJEB87706	ERS23920440
Eli_20338	20338	*Mechanitis lysimnia roqueensis*	*Mec. lysimnia roqueensis*	lysimniaEcu	include	Ecuador	2005	-0.524863	-76.38418	M	Anangu, Orellana	227.67	9.38569	PRJEB87706	ERS23920441
Eli_20367	20367	*Mechanitis lysimnia roqueensis*	*Mec. lysimnia roqueensis*	lysimniaEcu	include	Ecuador	2005	-0.524863	-76.38418	F	Anangu, Orellana	227.67	14.4172	PRJEB87706	ERS23920442
Eli_20547	20547	*Mechanitis lysimnia roqueensis*	*Mec. lysimnia roqueensis*	lysimniaEcu	include	Ecuador	2005	-0.524863	-76.38418	F	Anangu, Orellana	227.67	14.4104	PRJEB87706	ERS23920443
CAM008476	CAM008476	*Mechanitis lysimnia macrinus*	*Mec. macrinus macrinus*	macrinus	include	Panama	2001	8.713114	-79.90659	F	Cerro Campana	281.546	8.1879	PRJEB87706	ERS23920444
CAM008822	CAM008822	*Mechanitis lysimnia macrinus*	*Mec. macrinus macrinus*	macrinus	include	Panama	2002	7.8000592	-77.46355	F	Cana, around runway area, Darién	191.876	8.63882	PRJEB87706	ERS23920445
CAM008975	CAM008975	*Mechanitis lysimnia macrinus*	*Mec. macrinus macrinus*	macrinus	include	Panama	2003	7.6429589	-78.19232	F	Rio Piñas campsite, Darién	87.173	9.18807	PRJEB87706	ERS23920446
Sal_1986	M1986	*Mechanitis lysimnia macrinus*	*Mec. macrinus macrinus*	macrinus	include	Colombia	2006	4.9645722	-74.43481	F	Sasaima, Cundinamarca	1180.54	10.5562	PRJEB87706	ERS23920447
Sal_4176	M4176	*Mechanitis lysimnia macrinus*	*Mec. macrinus macrinus*	macrinus	include	Colombia	2016	5.6585829	-74.18148	M	El Carmen1-Otanche, Boyaca	1030.671	12.7209	PRJEB87706	ERS23920448
SAN25001776	LEP-08641	*Mechanitis lysimnia macrinus*	*Mec. macrinus macrinus*	macrinus	include	Ecuador	NA	1.13	-78.658	M*	Esmeraldas: Ridge N La Ceiba	254.2	30.1365	PRJEB63068	SAMEA114529896
SAN25001777	LEP-08646	*Mechanitis lysimnia macrinus*	*Mec. macrinus macrinus*	macrinus	include	Ecuador	NA	1.13	-78.658	M*	Esmeraldas: Ridge N La Ceiba	254.2	35.3045	PRJEB63068	SAMEA114529897
CAM008057	CAM008057	*Mechanitis lysimnia utemaia*	*Mec. macrinus utemaia*	macrinus	include	Panama	2000	8.9569032	-82.14786	F	Chiriqui Grande - Almirante 9.5km, Bocas del Toro	16.469	11.0399	PRJEB87706	ERS23920449
CAM008092	CAM008092	*Mechanitis lysimnia utemaia*	*Mec. macrinus utemaia*	macrinus	include	Panama	2001	8.9569738	-82.14413	F	Bocas del Toro, Chiriquí	5.541	11.2882	PRJEB87706	ERS23920450
CAM008432	CAM008432	*Mechanitis lysimnia utemaia*	*Mec. macrinus utemaia*	macrinus	include	Panama	2001	8.7443078	-82.24991	M	Quebrada Hornito Trail Fortuna, Chiriquí	1009.938	9.23806	PRJEB87706	ERS23920451
CAM042050	CAM042050	*Mechanitis mazaeus fallax*	*Mec. mazaeus fallax*	mazaeus	include	Ecuador	2019	-0.993443	-77.81284	M	Napo	506.416	9.29747	PRJEB87706	ERS23920452
CAM042128	CAM042128	*Mechanitis mazaeus fallax*	*Mec. mazaeus fallax*	mazaeus	include	Ecuador	2019	-0.993443	-77.81284	F	Napo	506.416	8.63007	PRJEB87706	ERS23920453
Eli_mm14_0014	mm14-14	*Mechanitis mazaeus fallax*	*Mec. mazaeus fallax*	mazaeus	include	Peru	2014	-6.4805835	-76.34799	M	PB km 7.5	411.595	10.9364	PRJEB87706	ERS23920454
Eli_mm15_0887	mm15-887	*Mechanitis mazaeus fallax*	*Mec. mazaeus fallax*	mazaeus	include	Peru	2015	-6.4805835	-76.34799	F	PB km 7.5	411.595	12.0112	PRJEB87706	ERS23920455
Eli_mm15_0888	mm15-888	*Mechanitis mazaeus fallax*	*Mec. mazaeus fallax*	mazaeus	include	Peru	2015	-6.4805835	-76.34799	F	PB km 7.5	411.595	12.2197	PRJEB87706	ERS23920456
Mon_2012_172	Yas_2012_StMo_172	*Mechanitis mazaeus fallax*	*Mec. mazaeus fallax*	mazaeus	include	Ecuador	2012	-0.5680873	-75.7582	M	Yasuni, Napo	215.942	16.0838	PRJEB87706	ERS23920457
Mon_2012_173	Yas_2012_StMo_173	*Mechanitis mazaeus fallax*	*Mec. mazaeus fallax*	mazaeus	include	Ecuador	2012	-0.5680873	-75.7582	F	Yasuni, Napo	215.942	11.7799	PRJEB87706	ERS23920458
Mon_2012_197	Yas_2012_StMo_197	*Mechanitis mazaeus fallax*	*Mec. mazaeus fallax*	mazaeus	include	Ecuador	2012	-0.5680873	-75.7582	M	Yasuni, Napo	215.942	13.8087	PRJEB87706	ERS23920459
Sal_1056	M1056	*Mechanitis mazaeus fallax*	*Mec. mazaeus fallax*	mazaeus	include	Colombia	2004	4.1659898	-73.68238	F	Buenavista, Meta	1183.243	12.1138	PRJEB87706	ERS23920460
Sal_1298	M1298	*Mechanitis mazaeus fallax*	*Mec. mazaeus fallax*	mazaeus	include	Colombia	2005	1.9097464	-75.15582	F	Puerto Rico, Caquetá	253	13.2132	PRJEB87706	ERS23920461
Sal_5319	M5319	*Mechanitis mazaeus fallax*	*Mec. mazaeus fallax*	mazaeus	include	Colombia	2018	-1.3220609	-69.57811	F	Vereda Angostura - La Pedrera, Amazonas	70.693	11.0242	PRJEB87706	ERS23920462
Sal_5427	M5427	*Mechanitis mazaeus fallax*	*Mec. mazaeus fallax*	mazaeus	include	Colombia	2018	-1.3220609	-69.57811	F	Sendero Bacurí - La Pedrera, Amazonas	70.693	12.2512	PRJEB87706	ERS23920463
Sal_2064	M2064	*Mechanitis menapis caribensis*	*Mec. menapis caribensis*	menapis	include	Colombia	2006	6.097464	-73.32032	F	Tolota, Santander	1711.3	11.0798	PRJEB87706	ERS23920464
Sal_2066	M2066	*Mechanitis menapis caribensis*	*Mec. menapis caribensis*	menapis	include	Colombia	2006	6.097464	-73.32032	F	Tolota, Santander	1711.3	12.0029	PRJEB87706	ERS23920465
Sal_5127	M5127	*Mechanitis menapis caribensis*	*Mec. menapis caribensis*	menapis	exclude	Colombia	NA	4.481846	-75.76605	M	La paila, Cucuta	1198.659	10.263	PRJEB87706	ERS23920466
Sal_5640	M5640	*Mechanitis menapis caribensis*	*Mec. menapis caribensis*	menapis	include	Colombia	2020	5.6803162	-73.52136	F	La Rosita -casa familia Valero, Boyaca	2233.268	12.8262	PRJEB87706	ERS23920467
Sal_5641	M5641	*Mechanitis menapis caribensis*	*Mec. menapis caribensis*	menapis	include	Colombia	2020	5.6803162	-73.52136	F	La Rosita -casa familia Valero, Boyaca	2233.268	12.2877	PRJEB87706	ERS23920468
Sal_1938	M1938	*Mechanitis menapis dariensis*	*Mec. menapis dariensis*	menapis	include	Colombia	2006	3.6105042	-76.59797	F	Bitaco, Valle del Cauca	1490.788	10.3853	PRJEB87706	ERS23920469
Sal_3342	M3342	*Mechanitis menapis dariensis*	*Mec. menapis dariensis*	menapis	include	Colombia	2011	6.5593804	-75.8256	F	Santafé de Antioquia, Antioquia	569.408	15.2219	PRJEB87706	ERS23920470
CAM070759	CAM070759	*Mechanitis menapis mantineus*	*Mec. menapis mantineus*	menapis	include	Ecuador	2022	0.1659866	-78.87828	M	Mashpi Lodge, Azuay	918.494	27.525	PRJEB63068	SAMEA112696604
SAN25001770	LEP-55153	*Mechanitis menapis mantineus*	*Mec. menapis mantineus*	menapis	include	Ecuador	NA	0.739	-78.242	F*	Imbabura: Bosque de Paz	967	29.9997	PRJEB63068	SAMEA114529898
SAN25001771	LEP-55155	*Mechanitis menapis mantineus*	*Mec. menapis mantineus*	menapis	include	Ecuador	NA	0.888	-78.438	F*	Carchi: Lita, Ridge east of Rio Baboso	895.64	31.3096	PRJEB63068	SAMEA114529899
SAN25001772	LEP-58365	*Mechanitis menapis mantineus*	*Mec. menapis mantineus*	menapis	include	Ecuador	NA	1.13	-78.658	F*	Esmeraldas: Ridge N La Ceiba	254.2	24.2407	PRJEB63068	SAMEA114529892
SAN25001773	LEP-58366	*Mechanitis menapis mantineus*	*Mec. menapis mantineus*	menapis	include	Ecuador	NA	1.13	-78.658	F*	Esmeraldas: Ridge N La Ceiba	254.2	26.5063	PRJEB63068	SAMEA114529893
Sal_5638	M5638	*Mechanitis menapis menapis*	*Mec. menapis menapis*	menapis	include	Colombia	2020	5.6803162	-73.52136	F	La Rosita -casa familia Valero, Boyaca	2233.268	11.6516	PRJEB87706	ERS23920471
Sal_4723	M4723	*Mechanitis menapis occasiva*	*Mec. menapis occasiva*	menapis	include	Colombia	2017	3.3230839	-76.62916	F	Pance - Topacio, Valle del Cauca	1582.093	15.8544	PRJEB87706	ERS23920472
Sal_5681	M5681	*Mechanitis menapis occasiva*	*Mec. menapis occasiva*	menapis	include	Colombia	2020	3.6855664	-76.52802	F	Montañitas, Valle del Cauca	1580.377	9.7703	PRJEB87706	ERS23920473
Sal_5683	M5683	*Mechanitis menapis occasiva*	*Mec. menapis occasiva*	menapis	include	Colombia	2020	3.6855664	-76.52802	M	Montañitas, Valle del Cauca	1580.377	12.5592	PRJEB87706	ERS23920474
CAM008026	CAM008026	*Mechanitis menapis saturata*	*Mec. menapis saturata*	menapis	include	Panama	2000	8.8340568	-82.71632	F	Cafe Duran Finca near Santa Clara, Chiriquí	1237.469	10.1695	PRJEB87706	ERS23920475
CAM008324	CAM008324	*Mechanitis menapis saturata*	*Mec. menapis saturata*	menapis	include	Panama	2001	8.7443078	-82.24991	M	Quebrada Hornito Trail Fortuna, Chiriquí	1009.938	12.5072	PRJEB87706	ERS23920476
CAM008325	CAM008325	*Mechanitis menapis saturata*	*Mec. menapis saturata*	menapis	include	Panama	2001	8.7443078	-82.24991	M	Quebrada Hornito Trail Fortuna, Chiriquí	1009.938	13.4405	PRJEB87706	ERS23920477
CAM008423	CAM008423	*Mechanitis menapis saturata*	*Mec. menapis saturata*	menapis	include	Panama	2001	8.7829144	-82.63632	F	Cafe Vista Hermosa Fortuna, Chiriquí	1421.614	11.8244	PRJEB87706	ERS23920478
CAM077079	M6152	*Mechanitis messenoides deceptus*	*Mec. mes. deceptus*	messenoides	include	Colombia	2022	1.147492	-76.64801	F*	Mocoa, Putumayo	604.321	32.2813	PRJEB63068	SAMEA112329128
CAM077080	M6153	*Mechanitis messenoides deceptus*	*Mec. mes. deceptus*	messenoides	include	Colombia	2022	1.147492	-76.64801	M*	Mocoa, Putumayo	604.321	43.3148	PRJEB63068	SAMEA112329131
Mon_2011_124	Yas_2011_StMo_124	*Mechanitis messenoides deceptus*	*Mec. mes. deceptus*	messenoides	include	Ecuador	2011	-0.5680873	-75.7582	F	Yasuni, Napo	215.942	15.133	PRJEB87706	ERS23920500
Mon_2011_195	Yas_2011_StMo_195	*Mechanitis messenoides deceptus*	*Mec. mes. deceptus*	messenoides	include	Ecuador	2011	-0.5680873	-75.7582	M	Yasuni, Napo	215.942	14.7891	PRJEB87706	ERS23920502
Mon_2012_146	Yas_2012_StMo_146	*Mechanitis messenoides deceptus*	*Mec. mes. deceptus*	messenoides	include	Ecuador	2012	-0.5680873	-75.7582	M	Yasuni, Napo	215.942	19.084	PRJEB87706	ERS23920506
Mon_2012_198	Yas_2012_StMo_198	*Mechanitis messenoides deceptus*	*Mec. mes. deceptus*	messenoides	include	Ecuador	2012	-0.5680873	-75.7582	F	Yasuni, Napo	215.942	17.194	PRJEB87706	ERS23920508
Mon_2012_243	Yas_2012_StMo_243	*Mechanitis messenoides deceptus*	*Mec. mes. deceptus*	messenoides	include	Ecuador	2012	-0.5680873	-75.7582	M	Yasuni, Napo	215.942	16.2453	PRJEB87706	ERS23920509
Mon_2012_88	Yas_2012_StMo_88	*Mechanitis messenoides deceptus*	*Mec. mes. deceptus*	messenoides	include	Ecuador	2012	-0.5680873	-75.7582	F*	Yasuni, Napo	215.942	15.2826	PRJEB87706	ERS23920513
Sal_1360	M1360	*Mechanitis messenoides deceptus*	*Mec. mes. deceptus*	messenoides	include	Colombia	2005	4.1659898	-73.68238	F	Buenavista, Meta	1183.243	12.7963	PRJEB87706	ERS23920514
Sal_1532	M1532	*Mechanitis messenoides deceptus*	*Mec. mes. deceptus*	messenoides	include	Colombia	2005	4.1659898	-73.68238	F	Buenavista, Meta	1183.243	12.0195	PRJEB87706	ERS23920515
Mon_2012_219	Yas_2012_StMo_219	*Mechanitis messenoides mes/dec*	*Mec. mes. mes/dec*	messenoides	include	Ecuador	2012	-0.5680873	-75.7582	M	Yasuni, Napo	215.942	14.8432	PRJEB87706	ERS23920520
CAM077045	M6102	*Mechanitis messenoides messenoides*	*Mec. mes. messenoides*	messenoides	include	Colombia	2022	1.147492	-76.64801	F*	Mocoa, Putumayo	604.321	33.8994	PRJEB63068	SAMEA112329095
CAM077081	M6154	*Mechanitis messenoides messenoides*	*Mec. mes. messenoides*	messenoides	include	Colombia	2022	1.147492	-76.64801	M*	Mocoa, Putumayo	604.321	35.5861	PRJEB63068	SAMEA112329134
CAM077083	M6156	*Mechanitis messenoides messenoides*	*Mec. mes. messenoides*	messenoides	include	Colombia	2022	1.147492	-76.64801	M*	Mocoa, Putumayo	604.321	34.4739	PRJEB63068	SAMEA112329140
CAM077084	M6157	*Mechanitis messenoides messenoides*	*Mec. mes. messenoides*	messenoides	include	Colombia	2022	1.147492	-76.64801	M*	Mocoa, Putumayo	604.321	36.8726	PRJEB63068	SAMEA112329143
CAM077091	M6158	*Mechanitis messenoides messenoides*	*Mec. mes. messenoides*	messenoides	include	Colombia	2022	1.147492	-76.64801	M*	Mocoa, Putumayo	604.321	42.212	PRJEB63068	SAMEA112329152
CAM077092	M6165	*Mechanitis messenoides messenoides*	*Mec. mes. messenoides*	messenoides	include	Colombia	2022	1.147492	-76.64801	M*	Mocoa, Putumayo	604.321	37.6697	PRJEB63068	SAMEA112329155
Mon_2012_220	Yas_2012_StMo_220	*Mechanitis messenoides messenoides*	*Mec. mes. messenoides*	messenoides	include	Ecuador	2012	-0.5680873	-75.7582	M	Yasuni, Napo	215.942	15.2761	PRJEB87706	ERS23920525
Mon_2012_242	Yas_2012_StMo_242	*Mechanitis messenoides messenoides*	*Mec. mes. messenoides*	messenoides	include	Ecuador	2012	-0.5680873	-75.7582	M	Yasuni, Napo	215.942	14.9384	PRJEB87706	ERS23920527
Mon_2012_244	Yas_2012_StMo_244	*Mechanitis messenoides messenoides*	*Mec. mes. messenoides*	messenoides	include	Ecuador	2012	-0.5680873	-75.7582	M	Yasuni, Napo	215.942	14.7535	PRJEB87706	ERS23920528
Mon_2012_90	Yas_2012_StMo_90	*Mechanitis messenoides messenoides*	*Mec. mes. messenoides*	messenoides	include	Ecuador	2012	-0.5680873	-75.7582	M	Yasuni, Napo	215.942	14.2619	PRJEB87706	ERS23920531
Mon_2012_99	Yas_2012_StMo_99	*Mechanitis messenoides messenoides*	*Mec. mes. messenoides*	messenoides	include	Ecuador	2012	-0.5680873	-75.7582	F*	Yasuni, Napo	215.942	15.419	PRJEB87706	ERS23920532
50-02-348	50-02-348	*Mechanitis messenoides phasianita*	*Mec. mes. phasianita*	messenoides	include	Peru	2002	-6.3353	-76.2542	F	Pongo de Caynarachi	252.739	30.6697	PRJEB87706	ERS23920572
55-02-1284	55-02-1284	*Mechanitis messenoides phasianita*	*Mec. mes. phasianita*	messenoides	include	Peru	2002	-6.2868	-76.2317	F	Km-7.2, Pongo-Barranquita	190.83	28.5735	PRJEB87706	ERS23920573
60-02-1290	60-02-1290	*Mechanitis messenoides phasianita*	*Mec. mes. phasianita*	messenoides	include	Peru	2002	-6.2868	-76.2317	F	Km-7.2, Pongo-Barranquita	190.83	34.3459	PRJEB87706	ERS23920574
61-02-1896	61-02-1896	*Mechanitis messenoides phasianita*	*Mec. mes. phasianita*	messenoides	include	Peru	2002	-6.6247	-76.4564	F	Km-30, Tarapoto-Yurimaguas	826.117	27.4076	PRJEB87706	ERS23920575
65-02-477	65-02-477	*Mechanitis messenoides phasianita*	*Mec. mes. phasianita*	messenoides	include	Peru	2002	-6.4083	-76.325	F	Km-28, Tarapoto-Yurimaguas	806.12	30.6185	PRJEB87706	ERS23920576
68-02-677	68-02-677	*Mechanitis messenoides phasianita*	*Mec. mes. phasianita*	messenoides	include	Peru	2002	-6.4618	-76.2919	M	Biodiversidad USM, Km-19, Tarapoto-Yurimaguas	921.67	31.2007	PRJEB87706	ERS23920577
CAM036038	CAM036038	*Mechanitis lysimnia nesaea*	*Mec. nesaea nesaea*	nesaea	exclude	Brazil	2020	-8.725661	-35.17961	F	Saltinho	57.311	9.94508	PRJEB87706	ERS23920580

CAM036042	CAM036042	*Mechanitis lysimnia nesaea*	*Mec. nesaea nesaea*	nesaea	exclude	Brazil	2020	-8.725661	-35.17961	F	Saltinho	57.311	9.85616	PRJEB87706	ERS23920581
CAM036060	CAM036060	*Mechanitis lysimnia nesaea*	*Mec. nesaea nesaea*	nesaea	exclude	Brazil	2020	-8.725661	-35.17961	F	Saltinho	57.311	10.6923	PRJEB87706	ERS23920582
CAM036081	CAM036081	*Mechanitis lysimnia nesaea*	*Mec. nesaea nesaea*	nesaea	exclude	Brazil	2020	-7.925017	-34.93332	M	Caetes	93.024	10.131	PRJEB87706	ERS23920583
CAM036084	CAM036084	*Mechanitis lysimnia nesaea*	*Mec. nesaea nesaea*	nesaea	include	Brazil	2020	-7.925017	-34.93332	M	Caetes	93.024	12.0614	PRJEB87706	ERS23920584
CAM036146	CAM036146	*Mechanitis lysimnia nesaea*	*Mec. nesaea nesaea*	nesaea	exclude	Brazil	2020	-6.730095	-35.1427	M	Guaribas	112.818	10.224	PRJEB87706	ERS23920585
CAM036153	CAM036153	*Mechanitis lysimnia nesaea*	*Mec. nesaea nesaea*	nesaea	exclude	Brazil	2020	-6.724533	-35.14482	F	Guaribas	94.231	10.5213	PRJEB87706	ERS23920586
CAM036207	CAM036207	*Mechanitis lysimnia nesaea*	*Mec. nesaea nesaea*	nesaea	include	Brazil	2020	-6.724533	-35.14482	M	Guaribas	94.231	12.0259	PRJEB87706	ERS23920587
CAM036278	CAM036278	*Mechanitis lysimnia nesaea*	*Mec. nesaea nesaea*	nesaea	exclude	Brazil	2020	-15.405748	-39.54844	M	Serra Bonita	304.581	8.94742	PRJEB87706	ERS23920588
CAM036280	CAM036280	*Mechanitis lysimnia nesaea*	*Mec. nesaea nesaea*	nesaea	include	Brazil	2020	-15.405748	-39.54844	M	Serra Bonita	304.581	12.021	PRJEB87706	ERS23920589
CAM036281	CAM036281	*Mechanitis lysimnia nesaea*	*Mec. nesaea nesaea*	nesaea	exclude	Brazil	2020	-15.403946	-39.54858	F	Serra Bonita	322.801	9.48588	PRJEB87706	ERS23920590
CAM036337	CAM036337	*Mechanitis lysimnia nesaea*	*Mec. nesaea nesaea*	nesaea	exclude	Brazil	2020	-15.406724	-39.54846	M	Serra Bonita	287.456	11.0083	PRJEB87706	ERS23920591
MCN011	MCN011	*Mechanitis lysimnia nesaea*	*Mec. nesaea nesaea*	nesaea	include	Brazil	2021	-15.361908	-39.57668	M*	Serra Bonita	551.573	17.6524	PRJEB87706	ERS23920592
MCN015	MCN015	*Mechanitis lysimnia nesaea*	*Mec. nesaea nesaea*	nesaea	include	Brazil	2022	-10.685365	-37.43606	F*	PNSerra Itabaiana, SE	206.258	13.1977	PRJEB87706	ERS23920593
MCN016	MCN016	*Mechanitis lysimnia nesaea*	*Mec. nesaea nesaea*	nesaea	include	Brazil	NA	NA	NA	F*	Areia Branca, SE	NA	16.6144	PRJEB87706	ERS23920594
CAM036418	CAM036418	*Mechanitis polymnia casabranca*	*Mec. pol. casabranca*	polymniaBraz	exclude	Brazil	2020	-22.428577	-44.61974	M	Itatiaia	1106.66	11.9244	PRJEB87706	ERS23920595
CAM036419	CAM036419	*Mechanitis polymnia casabranca*	*Mec. pol. casabranca*	polymniaBraz	exclude	Brazil	2020	-22.437925	-44.61271	M	Itatiaia	1003.061	10.2508	PRJEB87706	ERS23920596
LBR0644	LBR0644	*Mechanitis polymnia casabranca*	*Mec. pol. casabranca*	polymniaBraz	include	Brazil	2018	-18.355049	-39.84209	M*	ES, Conceição da Barra, Flona do Rio Preto, Trilha da Lagoa Seca	33.734	16.6629	PRJEB87706	ERS23920597
LBR0693	LBR0693	*Mechanitis polymnia casabranca*	*Mec. pol. casabranca*	polymniaBraz	exclude	Brazil	2018	-18.355049	-39.84209	M*	ES, Conceição da Barra, Flona do Rio Preto, Trilha da Lagoa Seca	33.734	14.5857	PRJEB87706	ERS23920598
LBR0703	LBR0703	*Mechanitis polymnia casabranca*	*Mec. pol. casabranca*	polymniaBraz	include	Brazil	2018	-18.582374	-39.72888	M*	Conceicao da Barra, ES	0	15.0083	PRJEB87706	ERS23920599
LBR0716	LBR0716	*Mechanitis polymnia casabranca*	*Mec. pol. casabranca*	polymniaBraz	include	Brazil	2018	-22.317409	-46.32517	M*	Incofidentes, MG	861.216	16.0719	PRJEB87706	ERS23920600
LBR2110	LBR2110	*Mechanitis polymnia casabranca*	*Mec. pol. casabranca*	polymniaBraz	include	Brazil	2019	-9.2423133	-36.41815	F*	Alagoas, Ponto 3, REBIO de Pedra Talhada	824.032	17.5549	PRJEB87706	ERS23920601
MCN013	MCN013	*Mechanitis polymnia casabranca*	*Mec. pol. casabranca*	polymniaBraz	include	Brazil	2018	-24.319406	-46.99659	F*	Peruibe, SP	8.861	17.4261	PRJEB87706	ERS23920602
MCN017	MCN017	*Mechanitis polymnia casabranca*	*Mec. pol. casabranca*	polymniaBraz	include	Brazil	2017	-20.284035	-50.24386	F*	Fernandopolis, SP	525.182	16.8255	PRJEB87706	ERS23920603
Sal_2038	M2038	*Mechanitis polymnia caucaensis*	*Mec. pol. caucaensis*	polymniaWest	include	Colombia	2008	3.6604101	-76.92442	M	Ladrilleros, Valle del Cauca	335.542	11.4886	PRJEB87706	ERS23920604
Sal_2113	M2113	*Mechanitis polymnia caucaensis*	*Mec. pol. caucaensis*	polymniaWest	include	Colombia	2008	3.6604101	-76.92442	M	Ladrilleros, Valle del Cauca	335.542	11.0647	PRJEB87706	ERS23920605
Sal_2115	M2115	*Mechanitis polymnia caucaensis*	*Mec. pol. caucaensis*	polymniaWest	include	Colombia	2008	3.6604101	-76.92442	F	Ladrilleros, Valle del Cauca	335.542	12.3321	PRJEB87706	ERS23920606
Sal_2193	M2193	*Mechanitis polymnia caucaensis*	*Mec. pol. caucaensis*	polymniaWest	include	Colombia	2008	3.6604101	-76.92442	M	Ladrilleros, Valle del Cauca	335.542	14.9999	PRJEB87706	ERS23920607
Sal_2195	M2195	*Mechanitis polymnia caucaensis*	*Mec. pol. caucaensis*	polymniaWest	include	Colombia	2008	3.6604101	-76.92442	F	Ladrilleros, Valle del Cauca	335.542	14.4424	PRJEB87706	ERS23920608
Sal_4604	M4604	*Mechanitis polymnia caucaensis*	*Mec. pol. caucaensis*	polymniaWest	include	Colombia	2017	3.6604101	-76.92442	F	Ladrilleros- Buenaventura, Valle del cauca	335.542	4.41947	PRJEB87706	ERS23920609
CAM071976	CAM071976	*Mechanitis polymnia chimborazona*	*Mec. pol. chimborazona*	polymniaWest	include	Ecuador	2023	1.182986	-78.75295	M	Tundaloma Lodge, Esmeraldas	33.465	30.7778	PRJEB63068	SAMEA114395576
CAM071977	CAM071977	*Mechanitis polymnia chimborazona*	*Mec. pol. chimborazona*	polymniaWest	include	Ecuador	2023	1.182986	-78.75295	M	Tundaloma Lodge, Esmeraldas	33.465	31.4595	PRJEB63068	SAMEA114395577
CAM071978	CAM071978	*Mechanitis polymnia chimborazona*	*Mec. pol. chimborazona*	polymniaWest	include	Ecuador	2023	1.182986	-78.75295	M	Tundaloma Lodge, Esmeraldas	33.465	32.8102	PRJEB63068	SAMEA114395578
CAM071979	CAM071979	*Mechanitis polymnia chimborazona*	*Mec. pol. chimborazona*	polymniaWest	include	Ecuador	2023	1.182986	-78.75295	M	Tundaloma Lodge, Esmeraldas	33.465	32.495	PRJEB63068	SAMEA114395579
CAM028186	CAM028186	*Mechanitis polymnia dorissides*	*Mec. pol. dorissides*	polymniaEast	exclude	Ecuador	2019	-0.9484307	-77.59437	M*	Rio Pusuno, Napo	835.795	11.271	PRJEB87706	ERS23920610
CAM028191	CAM028191	*Mechanitis polymnia dorissides*	*Mec. pol. dorissides*	polymniaEast	exclude	Ecuador	2019	-0.9484307	-77.59437	M*	Rio Pusuno, Napo	835.795	10.8787	PRJEB87706	ERS23920611
CAM028219	CAM028219	*Mechanitis polymnia dorissides*	*Mec. pol. dorissides*	polymniaEast	exclude	Ecuador	2019	-0.9484307	-77.59437	M*	Rio Pusuno, Napo	835.795	5.06811	PRJEB87706	ERS23920612
CAM028274	CAM028274	*Mechanitis polymnia dorissides*	*Mec. pol. dorissides*	polymniaEast	exclude	Ecuador	2019	-0.9484307	-77.59437	M*	Rio Pusuno, Napo	835.795	11.2457	PRJEB87706	ERS23920613
CAM028276	CAM028276	*Mechanitis polymnia dorissides*	*Mec. pol. dorissides*	polymniaEast	include	Ecuador	2019	-0.9484307	-77.59437	M*	Rio Pusuno, Napo	835.795	11.5295	PRJEB87706	ERS23920614
CAM028331	CAM028331	*Mechanitis polymnia dorissides*	*Mec. pol. dorissides*	polymniaEast	include	Ecuador	2019	-0.9501798	-77.86562	F*	Via Tena-Nuevo Angosta Km2.7, Napo	626.435	13.4645	PRJEB87706	ERS23920615
Eli_20339	20339	*Mechanitis polymnia dorissides*	*Mec. pol. dorissides*	polymniaEast	include	Ecuador	2005	-0.524863	-76.38418	M	Anangu, Orellana	227.67	13.0535	PRJEB87706	ERS23920616
Eli_20340	20340	*Mechanitis polymnia dorissides*	*Mec. pol. dorissides*	polymniaEast	include	Ecuador	2005	-0.524863	-76.38418	M	Anangu, Orellana	227.67	15.4496	PRJEB87706	ERS23920617
Eli_20728	20728	*Mechanitis polymnia dorissides*	*Mec. pol. dorissides*	polymniaEast	include	Ecuador	2005	-0.524863	-76.38418	M	Anangu, Orellana	227.67	11.4839	PRJEB87706	ERS23920618
Eli_21673	21673	*Mechanitis polymnia dorissides*	*Mec. pol. dorissides*	polymniaEast	include	Ecuador	2007	-0.524863	-76.38418	M	Anangu, Orellana	227.67	15.3154	PRJEB87706	ERS23920619
Eli_21677	21677	*Mechanitis polymnia dorissides*	*Mec. pol. dorissides*	polymniaEast	exclude	Ecuador	2007	-0.4617211	-76.9936	F	Coca, Orellana	250.982	9.50495	PRJEB87706	ERS23920620
Sal_1313	M1313	*Mechanitis polymnia dorissides*	*Mec. pol. dorissides*	polymniaEast	include	Colombia	2005	1.9097464	-75.15582	F	Puerto Rico, Caquetá	253	9.40955	PRJEB87706	ERS23920621
Sal_1374	M1374	*Mechanitis polymnia dorissides*	*Mec. pol. dorissides*	polymniaEast	include	Colombia	2005	1.7473478	-75.68861	F	Paraiso, Caquetá	1343.533	8.32645	PRJEB87706	ERS23920622
Sal_1413	M1413	*Mechanitis polymnia dorissides*	*Mec. pol. dorissides*	polymniaEast	include	Colombia	2005	1.5256897	-75.47937	M	Itarca, Caquetá	233.025	7.30326	PRJEB87706	ERS23920623
Sal_1416	M1416	*Mechanitis polymnia dorissides*	*Mec. pol. dorissides*	polymniaEast	include	Colombia	2005	1.5256897	-75.47937	F	Itarca, Caquetá	233.025	7.03627	PRJEB87706	ERS23920624
Sal_4944	M4944	*Mechanitis polymnia dorissides*	*Mec. pol. dorissides*	exclude	include	Colombia	2017	2.5824902	-72.68893	M	Playa Güio, Guaviare	189	15.7139	PRJEB87706	ERS23920625
CAM002881	CAM002881	*Mechanitis polymnia isthmia*	*Mec. pol. isthmia*	polymniaWest	include	Panama	2000	8.7663202	-78.17282	F	El Tirao, Darién	50.019	7.87924	PRJEB87706	ERS23920626
CAM002894	CAM002894	*Mechanitis polymnia isthmia*	*Mec. pol. isthmia*	polymniaWest	include	Panama	2000	8.7663202	-78.17282	F	El Tirao, Darién	50.019	9.20144	PRJEB87706	ERS23920627
CAM008058	CAM008058	*Mechanitis polymnia isthmia*	*Mec. pol. isthmia*	polymniaWest	include	Panama	2000	8.9569032	-82.14786	M	Chiriqui Grande - Almirante 9.5km, Bocas del Toro	16.469	12.2865	PRJEB87706	ERS23920628
CAM008287	CAM008287	*Mechanitis polymnia isthmia*	*Mec. pol. isthmia*	polymniaWest	include	Panama	2001	8.713114	-79.90659	M	Cerro Campana	281.546	13.5779	PRJEB87706	ERS23920629
CAM008288	CAM008288	*Mechanitis polymnia isthmia*	*Mec. pol. isthmia*	polymniaWest	include	Panama	2001	8.713114	-79.90659	M	Cerro Campana	281.546	9.89353	PRJEB87706	ERS23920630
Sal_5107	M5107	*Mechanitis polymnia isthmia*	*Mec. pol. isthmia*	polymniaWest	include	Colombia	2018	5.7121727	-75.31006	F	Municipio de Sonson, Experiencia viva Hotel campestre, Antioquia	2498.822	13.8266	PRJEB87706	ERS23920631
CAM009318	CAM009318	*Mechanitis polymnia polymnia*	*Mec. pol. polymnia*	polymniaGui	include	Fr Guiana	NA	4.5493027	-52.49203	M	Cacao - Cayenne, 9.9km, Cayenne	9.014	9.222	PRJEB87706	ERS23920632
CAM009319	CAM009319	*Mechanitis polymnia polymnia*	*Mec. pol. polymnia*	polymniaGui	include	Fr Guiana	NA	4.9399653	-52.33921	M	Devez Road, Cayenne	0	3.99727	PRJEB87706	ERS23920633
CAM021051	CAM021051	*Mechanitis polymnia polymnia*	*Mec. pol. polymnia*	polymniaGui	include	Suriname	2014	5.0255233	-54.99384	F	Brokopondo Road East, Brokopondo	17.324	10.4614	PRJEB87706	ERS23920634
Eli_mm15_0350	mm15-350	*Mechanitis polymnia proceriformis*	*Mec. pol. proceriformis*	polymniaPeru	include	Peru	2015	-6.025899	-75.87202	F	shucushyacu	141.873	12.5199	PRJEB87706	ERS23920635
Eli_mm15_0528	mm15-528	*Mechanitis polymnia proceriformis*	*Mec. pol. proceriformis*	polymniaPeru	include	Peru	2015	-6.0421934	-76.96754	M	Moyo, Rumipata	881.104	12.7524	PRJEB87706	ERS23920636
Eli_mm15_0530	mm15-530	*Mechanitis polymnia proceriformis*	*Mec. pol. proceriformis*	polymniaPeru	include	Peru	2015	-6.0421934	-76.96754	M	Moyo, Rumipata	881.104	13.2699	PRJEB87706	ERS23920637
CAM072004	CAM072004	*Eutresis hypereia*	*Eut. hypereia*	Outgroup (Mel.)	include	Ecuador	NA	-0.730204	-77.773665	M	Narupa Birding	1228.522	4.5897	PRJEB63068	SAMEA114298532
FS50864856	CAM071929	*Olyras insignis*	*Oly. insignis*	Outgroup (Mel.)	include	Ecuador	2023	0.885174	-78.436529	F	Lita Ridge	895.64	2.64989	PRJEB87706	ERS23920741
CAM008101	CAM008101	*Melinaea idae idae*	*Mel. idae idae*	idea	include	Panama	2001	9.2261912	-80.01518731	M*	Achiote Road, Colón	5.577	3.82078	PRJEB87706	ERS23920638
Sal_4172	M4172	*Melinaea idae idae*	*Mel. idae idae*	idea	include	Colombia	2016	5.6585829	-74.18148119	M	El Carmen1-Otanche, Boyaca	1030.671	4.86217	PRJEB87706	ERS23920639
Sal_4174	M4174	*Melinaea idae idae*	*Mel. idae idae*	idea	include	Colombia	2016	5.6585829	-74.18148119	M	El Carmen1-Otanche, Boyaca	1030.671	6.19511	PRJEB87706	ERS23920640
Sal_5620	M5620	*Melinaea idae idae*	*Mel. idae idae*	idea	include	Colombia	2020	5.6585668	-74.18118948	M	Potrero doña Blanca-Otanche, Boyaca	1030.364	5.45716	PRJEB87706	ERS23920641
Sal_4286	M4286	*Melinaea isocomma isocomma*	*Mel. isocomma isocomma*	isocomma	include	Colombia	2017	1.2040722	-76.70956725	M	Campucana, Putumayo	1531.454	5.28586	PRJEB87706	ERS23920642
SAN25001766	NHMUK015104092	*Melinaea isocomma isocomma*	*Mel. isocomma isocomma*	isocomma	include	Colombia	NA	NA	NA	M	NA	NA	3.80833	PRJEB63068	SAMEA114222960
SAN25001769	NHMUK015200312	*Melinaea isocomma isocomma*	*Mel. isocomma isocomma*	isocomma	include	Colombia	NA	NA	NA	M	NA	NA	2.42611	PRJEB63068	SAMEA114222958
CAM070923	CAM070923	*Melinaea isocomma simulator*	*Mel. isocomma simulator*	isocomma	include	Ecuador	2023	-0.725164	-77.767354	M	Finca Narupa, Bridge, Napo	1111	13.4451	PRJEB63068	SAMEA112696743
CAM070930	CAM070930	*Melinaea isocomma simulator*	*Mel. isocomma simulator*	isocomma	include	Ecuador	2022	-0.725164	-77.767354	M	Finca Narupa, Bridge, Napo	1111	12.0203	PRJEB63068	SAMEA114211500
CAM070960	CAM070960	*Melinaea isocomma simulator*	*Mel. isocomma simulator*	isocomma	include	Ecuador	2022	-0.725164	-77.767354	M	Finca Narupa, Bridge, Napo	1111	13.6209	PRJEB63068	SAMEA114211491
CAM071351	CAM071351	*Melinaea isocomma simulator*	*Mel. isocomma simulator*	isocomma	include	Ecuador	2022	-0.6746389	-77.60886111	F	Wildsumaco	1327.06	12.7849	PRJEB63068	SAMEA114394229
CAM071523	CAM071523	*Melinaea isocomma simulator*	*Mel. isocomma simulator*	isocomma	include	Ecuador	2023	-0.730204	-77.773665	F	Narupa Birding	1228.522	12.399	PRJEB63068	SAMEA114211495
CAM072002	CAM072002	*Melinaea isocomma simulator*	*Mel. isocomma simulator*	isocomma	include	Ecuador	2023	-0.730204	-77.773665	F	Narupa Birding	1228.522	12.1011	PRJEB63068	SAMEA114394231
FS50864630	2002-0688	*Melinaea isocomma simulator*	*Mel. isocomma simulator*	isocomma	include	Peru	2002	-5.6754	-77.6746	F*	Puente Serranoyacu, San Martin	1195.554	6.48787	PRJEB87706	ERS23920643
FS50864631	2002-0689	*Melinaea isocomma simulator*	*Mel. isocomma simulator*	isocomma	include	Peru	2002	-5.6754	-77.6746	F	Puente Serranoyacu, San Martin	1195.554	8.66875	PRJEB87706	ERS23920644
FS50864632	2002-0690	*Melinaea isocomma simulator*	*Mel. isocomma simulator*	isocomma	include	Peru	2002	-5.6754	-77.6746	F	Puente Serranoyacu, San Martin	1195.554	5.43407	PRJEB87706	ERS23920645
McD_I01	McD_I01	*Melinaea lilis imitata*	*Mel. lilis imitata*	lilis	include	Panama	2020	9.1604389	-82.51808014	NA	Palo Seco Forest Reserve, Bocas del Toro	660.751	3.10555	PRJEB87706	ERS23920646
McD_I02	McD_I02	*Melinaea lilis imitata*	*Mel. lilis imitata*	lilis	include	Panama	2020	9.1604389	-82.51808014	NA	Palo Seco Forest Reserve, Bocas del Toro	660.751	3.22922	PRJEB87706	ERS23920647
McD_I03	McD_I03	*Melinaea lilis imitata*	*Mel. lilis imitata*	lilis	include	Panama	2020	9.1604389	-82.51808014	NA	Palo Seco Forest Reserve, Bocas del Toro	660.751	3.17569	PRJEB87706	ERS23920648
McD_I04	McD_I04	*Melinaea lilis imitata*	*Mel. lilis imitata*	lilis	include	Panama	2020	9.1604389	-82.51808014	NA	Palo Seco Forest Reserve, Bocas del Toro	660.751	3.58474	PRJEB87706	ERS23920649
CAM027255	CAM027255	*Melinaea lilis parallelis*	*Mel. lilis parallelis*	lilis	include	Panama	2018	8.6254434	-80.0512129	F	Laguna San Carlo	771.373	2.64285	PRJEB87706	ERS23920650
McD_P01	McD_P01	*Melinaea lilis parallelis*	*Mel. lilis parallelis*	lilis	include	Panama	2020	9.1604389	-82.51808014	NA	Palo Seco Forest Reserve, Bocas del Toro	660.751	3.80605	PRJEB87706	ERS23920651
McD_P02	McD_P02	*Melinaea lilis parallelis*	*Mel. lilis parallelis*	lilis	include	Panama	2020	9.1604389	-82.51808014	NA	Palo Seco Forest Reserve, Bocas del Toro	660.751	3.58577	PRJEB87706	ERS23920652
McD_P03	McD_P03	*Melinaea lilis parallelis*	*Mel. lilis parallelis*	lilis	include	Panama	2020	9.1604389	-82.51808014	NA	Palo Seco Forest Reserve, Bocas del Toro	660.751	3.53204	PRJEB87706	ERS23920653
McD_P04	McD_P04	*Melinaea lilis parallelis*	*Mel. lilis parallelis*	lilis	include	Panama	2020	9.1604389	-82.51808014	NA	Palo Seco Forest Reserve, Bocas del Toro	660.751	3.5841	PRJEB87706	ERS23920654
CAM009348	CAM009348	*Melinaea ludovica*	*Mel. ludovica*	ludovica	include	Fr Guiana	NA	3.6437425	-53.99537461	M	N. Wakapou, Maripausala, Saint-Laurent-du-Maroni	112.622	3.78025	PRJEB87706	ERS23920655
Eli_mm16_007	mm17-007	*Melinaea ludovica ludovica*	*Mel. ludovica ludovica*	ludovica	include	Peru	2017	-6.025899	-75.87202157	M	Shucushyacu	141.873	2.45813	PRJEB87706	ERS23920656
Sal_5553	M5553	*Melinaea ludovica ludovica*	*Mel. ludovica ludovica*	ludovica	include	Colombia	NA	-3.79	-70.36	M	Puerto Nariño- Quebrada Aguas Rojas, Amazonas	91.915	4.04485	PRJEB87706	ERS23920657
CAM036282	CAM036282	*Melinaea ludovica parayia*	*Mel. ludovica parayia*	ludovica	include	Brazil	2020	-15.403946	-39.54857899	M	Serra Bonita	322.801	7.5135	PRJEB87706	ERS23920658
CAM036354	CAM036354	*Melinaea ludovica parayia*	*Mel. ludovica parayia*	ludovica	include	Brazil	2020	-21.684803	-48.09023939	M	Santa Lucia	701.711	4.07876	PRJEB87706	ERS23920659
CAM036594	CAM036594	*Melinaea ludovica parayia*	*Mel. ludovica parayia*	ludovica	include	Brazil	2020	NA	NA	M	Jureia	NA	5.13687	PRJEB87706	ERS23920660
CAM028332	CAM028332	*Melinaea satevis maeonis*	*Mel. maeonis*	maeonis	include	Ecuador	2019	-0.9501798	-77.8656204	F*	Via Tena-Nuevo Angosta Km2.7, Napo	626.435	5.0276	PRJEB87706	ERS23920661
CAM071086	CAM071086	*Melinaea satevis maeonis*	*Mel. maeonis*	maeonis	include	Ecuador	2022	0.1995667	-77.471	M	Puerto Libre Linea 2	710.48	8.67416	PRJEB63068	SAMEA114211497
Eli_20301	20301	*Melinaea satevis maeonis*	*Mel. maeonis*	maeonis	include	Ecuador	2005	-0.524863	-76.384183	M	Anangu, Napo	226.958	4.12592	PRJEB87706	ERS23920662
Eli_20549	20549	*Melinaea satevis maeonis*	*Mel. maeonis*	maeonis	include	Ecuador	2005	-0.524863	-76.384183	F	Anangu, Napo	226.958	3.90169	PRJEB87706	ERS23920663
Eli_20594	20594	*Melinaea satevis maeonis*	*Mel. maeonis*	maeonis	include	Ecuador	2005	-0.524863	-76.384183	F*	Anangu, Napo	226.958	4.45302	PRJEB87706	ERS23920664
FS50864626	E450	*Melinaea satevis maeonis*	*Mel. maeonis*	maeonis	include	Ecuador	2002	NA	NA	F	Jatun Sacha, Napo	NA	9.60045	PRJEB87706	ERS23920665

FS50864637	2002-3562	*Melinaea marsaeus clara*	*Mel. marsaeus clara*	marsaeus	include	Peru	2002	-13.2333	-70.75	M	Quincemil, Cuzco	629.76	7.60517	PRJEB87706	ERS23920666
Eli_21708	21708	*Melinaea marsaeus macaria*	*Mel. marsaeus macaria*	marsaeus	include	Ecuador	2007	NA	NA	M	Luis Garcia, Panayaku	NA	3.85919	PRJEB87706	ERS23920667
Eli_21726	21726	*Melinaea marsaeus macaria*	*Mel. marsaeus macaria*	marsaeus	include	Ecuador	2007	-0.4235457	-76.10119703	F	Ramiro Viteri, Panacocha	221.472	3.98785	PRJEB87706	ERS23920668
Eli_mm16_0292	mm16-292	*Melinaea marsaeus phasiana*	*Mel. marsaeus phasiana*	marsaeus	include	Peru	2016	-6.573466	-76.1375285	M	Chazuta	184.58	4.87241	PRJEB87706	ERS23920669
Eli_mm16_0327	mm16-327	*Melinaea marsaeus phasiana*	*Mel. marsaeus phasiana*	marsaeus	include	Peru	2016	-6.573466	-76.1375285	F	Chazuta	184.58	4.0949	PRJEB87706	ERS23920670
Eli_20811	20811	*Melinaea marsaeus rileyi*	*Mel. marsaeus rileyi*	marsaeus	include	Ecuador	2005	-0.524863	-76.384183	M	Anangu, Napo	226.958	3.4349	PRJEB87706	ERS23920671
Eli_mm16_0227	mm16-227	*Melinaea marsaeus rileyi*	*Mel. marsaeus rileyi*	marsaeus	include	Peru	2016	-6.025899	-75.87202157	F	Shucushyacu	141.873	3.92723	PRJEB87706	ERS23920672
Eli_mm16_0326	mm16-326	*Melinaea marsaeus rileyi*	*Mel. marsaeus rileyi*	marsaeus	include	Peru	2016	-6.025899	-75.87202157	M	Shucushyacu	141.873	3.6942	PRJEB87706	ERS23920673
CAM077050	M6120	*Melinaea menophilus ernestoi*	*Mel. men. ernestoi*	menophilus	include	Colombia	2022	1.147492	-76.64800808	M	Mocoa, Putumayo	604.321	14.628	PRJEB63068	SAMEA112663948
CAM077076	M6149	*Melinaea menophilus ernestoi*	*Mel. men. ernestoi*	menophilus	include	Colombia	2022	1.147492	-76.64800808	M	Mocoa, Putumayo	604.321	15.3842	PRJEB63068	SAMEA112664072
Sal_1135	M1135	*Melinaea menophilus ernestoi*	*Mel. men. ernestoi*	menophilus	include	Colombia	2005	1.786823	-75.65298657	F	Sucre, Caquetá	991.427	3.93483	PRJEB87706	ERS23920674
Sal_2983	M2983	*Melinaea menophilus ernestoi*	*Mel. men. ernestoi*	menophilus	include	Colombia	2010	0.8794321	-73.86996313	F	Q. Doraditas 1, Caquetá	239.956	4.41367	PRJEB87706	ERS23920675
Sal_408	M408	*Melinaea menophilus ernestoi*	*Mel. men. ernestoi*	menophilus	include	Colombia	2003	1.786823	-75.65298657	M	Sucre, Caquetá	991.427	4.97289	PRJEB87706	ERS23920676
Sal_4284	M4284	*Melinaea menophilus ernestoi*	*Mel. men. ernestoi*	menophilus	include	Colombia	2017	1.2040722	-76.70956725	F	Campucana, Putumayo	1531.454	4.74927	PRJEB87706	ERS23920677
Eli_mm16_0141	mm16-141	*Melinaea menophilus hicetas*	*Mel. men. hicetas*	menophilus	include	Peru	2016	-6.025899	-75.87202157	M	Shucushyacu	141.873	4.00594	PRJEB87706	ERS23920678
MJ09_4179	MJ09_4179	*Melinaea menophilus mediatrix*	*Mel. men. mediatrix*	menophilus	include	Fr Guiana	2019	4.01106	-52.64197	NA	Les Nouragues-Saut Pararé	119.755	13.8982	PRJEB87706	ERS23920679
MJ09_4208	MJ09_4208	*Melinaea menophilus mediatrix*	*Mel. men. mediatrix*	menophilus	include	Fr Guiana	2019	4.01106	-52.64197	NA	Les Nouragues-Inselberg	119.755	10.1738	PRJEB87706	ERS23920680
CAM070948	CAM070948	*Melinaea menophilus menophilus*	*Mel. men. menophilus*	menophilus	include	Ecuador	2022	-0.49673	-76.839035	F	Pozo Anaconda	255.514	12.1792	PRJEB63068	SAMEA114211490
CAM071272	CAM071272	*Melinaea menophilus menophilus*	*Mel. men. menophilus*	menophilus	include	Ecuador	2022	0.0101333	-76.259317	M	Pozo Cuyabeno	276.026	13.0653	PRJEB63068	SAMEA114211498
Eli_20234	20234	*Melinaea menophilus menophilus*	*Mel. men. menophilus*	menophilus	include	Ecuador	2005	-0.524863	-76.384183	M	Anangu, Napo	226.958	3.60695	PRJEB87706	ERS23920681
Eli_20252	20252	*Melinaea menophilus menophilus*	*Mel. men. menophilus*	menophilus	include	Ecuador	2005	-0.524863	-76.384183	M	Anangu, Napo	226.958	3.55453	PRJEB87706	ERS23920682
Eli_20302	20302	*Melinaea menophilus menophilus*	*Mel. men. menophilus*	menophilus	include	Ecuador	2005	-0.524863	-76.384183	M	Anangu, Napo	226.958	3.61136	PRJEB87706	ERS23920683
Eli_20336	20336	*Melinaea menophilus menophilus*	*Mel. men. menophilus*	menophilus	include	Ecuador	2005	-0.524863	-76.384183	F	Anangu, Napo	226.958	4.03646	PRJEB87706	ERS23920684
Eli_20730	20730	*Melinaea menophilus menophilus*	*Mel. men. menophilus*	menophilus	include	Ecuador	2005	-0.524863	-76.384183	F	Anangu, Napo	226.958	3.9815	PRJEB87706	ERS23920685
Sal_447	M447	*Melinaea menophilus menophilus*	*Mel. men. menophilus*	menophilus	include	Colombia	2003	1.786823	-75.65298657	M	Sucre, Caquetá	991.427	6.1394	PRJEB87706	ERS23920686
Eli_mm16_0313	mm16-313	*Melinaea menophilus ssp1*	*Mel. men. ssp1*	menophilus	include	Peru	2016	-6.4969024	-76.35857168	M	rio shilcayo	290.272	4.2959	PRJEB87706	ERS23920687
Eli_mm16_0314	mm16-314	*Melinaea menophilus ssp1*	*Mel. men. ssp2*	menophilus	include	Peru	2016	-6.4969024	-76.35857168	M	rio shilcayo	290.272	4.39352	PRJEB87706	ERS23920688
Eli_mm16_0317	mm16-317	*Melinaea menophilus ssp1*	*Mel. men. ssp3*	menophilus	include	Peru	2016	-6.4969024	-76.35857168	F	rio shilcayo	290.272	4.62917	PRJEB87706	ERS23920689
Eli_mm16_0324	mm16-324	*Melinaea menophilus ssp1*	*Mel. men. ssp4*	menophilus	include	Peru	2016	-6.0421934	-76.96753632	F	Moyo, Rumipata	881.104	4.00648	PRJEB87706	ERS23920690
CAM008526	CAM008526	*Melinaea menophilus zaneka*	*Mel. men. zaneka*	menophilus	include	Ecuador	2002	-1.4074569	-78.19110354	F*	El Topo, Baños - Puyo road, Tungurahua	1235.224	2.96625	PRJEB87706	ERS23920691
CAM008527	CAM008527	*Melinaea menophilus zaneka*	*Mel. men. zaneka*	menophilus	include	Ecuador	2002	-1.4074569	-78.19110354	F*	El Topo, Baños - Puyo road, Tungurahua	1235.224	4.54474	PRJEB87706	ERS23920692
CAM009136	CAM009136	*Melinaea menophilus zaneka*	*Mel. men. zaneka*	menophilus	include	Ecuador	2006	-2.2627388	-78.20150396	NA	Rio Abanico, Macas, Morona-Santiago	1713.55	4.23755	PRJEB87706	ERS23920693
CAM028162	CAM028162	*Melinaea menophilus zaneka*	*Mel. men. zaneka*	menophilus	include	Ecuador	2019	-0.725164	-77.767354	F*	Finca Narupa, Bridge, Napo	1111	4.8315	PRJEB87706	ERS23920694
CAM028163	CAM028163	*Melinaea menophilus zaneka*	*Mel. men. zaneka*	menophilus	include	Ecuador	2019	-0.725164	-77.767354	F*	Finca Narupa, Bridge, Napo	1111	3.41743	PRJEB87706	ERS23920695
CAM028398	CAM028398	*Melinaea menophilus zaneka*	*Mel. men. zaneka*	menophilus	include	Ecuador	2019	-0.7200278	-77.74708333	F*	Mina Negra, Napo	1239.95	3.83182	PRJEB87706	ERS23920696
CAM028402	CAM028402	*Melinaea menophilus zaneka*	*Mel. men. zaneka*	menophilus	include	Ecuador	2019	-0.725164	-77.767354	NA	Finca Narupa, Bridge, Napo	1111	4.10872	PRJEB87706	ERS23920697
CAM028403	CAM028403	*Melinaea menophilus zaneka*	*Mel. men. zaneka*	menophilus	include	Ecuador	2019	-0.725164	-77.767354	F*	Finca Narupa, Bridge, Napo	1111	3.91533	PRJEB87706	ERS23920698
CAM009349	CAM009349	*Melinaea mneme*	*Mel. mneme*	mneme	include	Fr Guiana	NA	3.6437425	-53.99537461	M	N. Wakapou, Maripausala, Saint-Laurent-du-Maroni	112.622	4.53344	PRJEB87706	ERS23920699
MJ09_4176	MJ09_4176	*Melinaea mneme*	*Mel. mneme*	mneme	include	Fr Guiana	2019	4.01106	-52.64197	NA	Les Nouragues-Saut Pararé	119.755	9.8136	PRJEB87706	ERS23920700
MJ09_4199	MJ09_4199	*Melinaea mneme*	*Mel. mneme*	mneme	include	Fr Guiana	2019	4.01106	-52.64197	NA	Les Nouragues-Inselberg	119.755	11.5887	PRJEB87706	ERS23920701
Sal_5371	M5371	*Melinaea mneme*	*Mel. mneme*	mneme	include	Colombia	2018	-1.3220609	-69.57810912	M	Vereda Madroño - La Pedrera, Amazonas	70.693	5.40237	PRJEB87706	ERS23920702
Sal_5393	M5393	*Melinaea mneme*	*Mel. mneme*	mneme	include	Colombia	2018	-1.3220609	-69.57810912	F	Sendero Bacurí - La Pedrera, Amazonas	70.693	5.79792	PRJEB87706	ERS23920703
CAM077033	M6090	*Melinaea mothone messenina*	*Mel. mothone messenina*	mothone	include	Colombia	2023	1.147492	-76.64800808	F	Mocoa, Putumayo	604.321	12.6359	PRJEB63068	SAMEA112329085
CAM077035	M6092	*Melinaea mothone messenina*	*Mel. mothone messenina*	mothone	include	Colombia	2022	1.147492	-76.64800808	F	Mocoa, Putumayo	604.321	16.3272	PRJEB63068	SAMEA112329086
Sal_1069	M1069	*Melinaea mothone messenina*	*Mel. mothone messenina*	mothone	include	Colombia	2005	1.786823	-75.65298657	M	Sucre, Caquetá	991.427	5.68367	PRJEB87706	ERS23920704
Sal_1136	M1136	*Melinaea mothone messenina*	*Mel. mothone messenina*	mothone	include	Colombia	2005	1.786823	-75.65298657	F	Sucre, Caquetá	991.427	5.18706	PRJEB87706	ERS23920706
CAM077000	M6039	*Melinaea mothone mot/mess*	*Mel. mothone mot/mess*	mothone	include	Colombia	2022	1.147492	-76.64800808	F	Mocoa, Putumayo	604.321	15.2665	PRJEB63068	SAMEA112329041
CAM070362	CAM070362	*Melinaea mothone mothone*	*Mel. mothone mothone*	mothone	include	Ecuador	2023	-0.725164	-77.767354	M*	Finca Narupa, Bridge, Napo	1111	15.8951	PRJEB63068	SAMEA114394288
CAM070921	CAM070921	*Melinaea mothone mothone*	*Mel. mothone mothone*	mothone	include	Ecuador	2023	-0.725164	-77.767354	M*	Finca Narupa, Bridge, Napo	1111	14.5282	PRJEB63068	SAMEA112696739
CAM070922	CAM070922	*Melinaea mothone mothone*	*Mel. mothone mothone*	mothone	include	Ecuador	2023	-0.725164	-77.767354	M*	Finca Narupa, Bridge, Napo	1111	14.6653	PRJEB63068	SAMEA112696741
CAM070924	CAM070924	*Melinaea mothone mothone*	*Mel. mothone mothone*	mothone	include	Ecuador	2023	-0.725164	-77.767354	M*	Finca Narupa, Bridge, Napo	1111	16.0467	PRJEB63068	SAMEA112696745
CAM070925	CAM070925	*Melinaea mothone mothone*	*Mel. mothone mothone*	mothone	include	Ecuador	2023	-0.725164	-77.767354	M*	Finca Narupa, Bridge, Napo	1111	15.0785	PRJEB63068	SAMEA112696747
CAM070926	CAM070926	*Melinaea mothone mothone*	*Mel. mothone mothone*	mothone	include	Ecuador	2023	-0.725164	-77.767354	M*	Finca Narupa, Bridge, Napo	1111	16.5245	PRJEB63068	SAMEA112696749
CAM077032	M6089	*Melinaea mothone mothone*	*Mel. mothone mothone*	mothone	include	Colombia	2023	1.147492	-76.64800808	F	Mocoa, Putumayo	604.321	13.6359	PRJEB63068	SAMEA112329082
FS50864628	2002-0378	*Melinaea mothone mothone*	*Mel. mothone mothone*	mothone	include	Peru	2023	-6.455	-76.2983	F	La Antena, Km-16, Tarapoto-Yurimaguas; San Martin	1167.12	8.77395	PRJEB87706	ERS23920719
FS50864629	2002-0675	*Melinaea mothone mothone*	*Mel. mothone mothone*	mothone	include	Peru	2023	-6.46183	-76.2919	M	Biodiversidad USM, Km-19, Tarapoto-Yurimaguas; San Martin	921.67	8.20904	PRJEB87706	ERS23920720
FS50864633	2002-0982	*Melinaea mothone mothone*	*Mel. mothone mothone*	mothone	include	Peru	2023	-6.4619	-76.3231	M	Km-10, Tarapoto-Yurimaguas, San Martin	729.032	8.06032	PRJEB87706	ERS23920721
FS50864635	2002-3558	*Melinaea mothone mothone*	*Mel. mothone mothone*	mothone	include	Peru	2023	-13.2167	-70.75	M	Quincemil, Cuzco	788.56	9.31932	PRJEB87706	ERS23920722
FS50864636	2002-3560	*Melinaea mothone mothone*	*Mel. mothone mothone*	mothone	include	Peru	2023	-13.2167	-70.75	M	Quincemil, Cuzco	788.56	7.51254	PRJEB87706	ERS23920723
FS50864642	2005-1342	*Melinaea mothone mothone*	*Mel. mothone mothone*	mothone	include	Peru	2023	-6.4519	-76.3461	M*	Río Shilcayo, Tarapoto, San Martin	476.659	9.24518	PRJEB87706	ERS23920725
Eli_mm16_0205	mm16-205	*Melinaea satevis cydon*	*Mel. satevis cydon*	satevis	include	Peru	2016	-6.4805835	-76.34799034	M	PB Km 7.5	411.595	3.53011	PRJEB87706	ERS23920731
Eli_mm16_0206	mm16-206	*Melinaea satevis cydon*	*Mel. satevis cydon*	satevis	include	Peru	2016	-6.4805835	-76.34799034	F	PB Km 7.5	411.595	4.11701	PRJEB87706	ERS23920732
Sal_5517	M5517	*Melinaea satevis cydon*	*Mel. satevis cydon*	satevis	include	Colombia	NA	-3.7886904	-70.35546689	M	Puerto Nariño- Quebrada Aguas Rojas, Amazonas	91.915	5.11485	PRJEB87706	ERS23920733
Sal_5570	M5570	*Melinaea satevis cydon*	*Mel. satevis cydon*	satevis	include	Colombia	NA	-3.79	-70.36	M	Puerto Nariño- Quebrada Aguas Rojas, Amazonas	91.915	4.7983	PRJEB87706	ERS23920734
SAN25001774	LEP-56663	*Melinaea satevis cydon*	*Mel. satevis cydon*	satevis	include	Ecuador	NA	-2.902	-77.742	F*	2.5 km N Puerto Morona	213	14.1981	PRJEB63068	SAMEA114529894
SAN25001775	LEP-57574	*Melinaea satevis cydon*	*Mel. satevis cydon*	satevis	include	Ecuador	NA	-2.912	-77.707	F*	km 3 Puerto Morona-San José de Morona	213	15.5379	PRJEB63068	SAMEA114529895
FS50864634	2002-3262	*Melinaea satevis lamasi*	*Mel. satevis lamasi*	satevis	include	Peru	2002	-12.9	-71.4	F	Pilcopata, Cuzco	512	8.72197	PRJEB87706	ERS23920735
Sal_5372	M5372	*Melinaea satevis maelus*	*Mel. satevis maelus*	satevis	include	Colombia	2018	-1.3220609	-69.57810912	F	Vereda Madroño - La Pedrera, Amazonas	70.693	4.21181	PRJEB87706	ERS23920736
Eli_mm16_0161	mm16-161	*Melinaea tarapotensis*	*Mel. tarapotensis*	tarapotensis	include	Peru	2016	-6.4837118	-76.3706028	M	Tarapoto	320.982	4.12229	PRJEB87706	ERS23920737
Eli_mm16_0173	mm16-173	*Melinaea tarapotensis*	*Mel. tarapotensis*	tarapotensis	include	Peru	2016	-6.4837118	-76.3706028	F	Tarapoto	320.982	3.81347	PRJEB87706	ERS23920738
Eli_mm16_0197	mm16-197	*Melinaea tarapotensis*	*Mel. tarapotensis*	tarapotensis	include	Peru	2016	-6.4837118	-76.3706028	M	Tarapoto	320.982	4.38264	PRJEB87706	ERS23920739
Eli_mm16_0296	mm16-296	*Melinaea tarapotensis*	*Mel. tarapotensis*	tarapotensis	include	Peru	2016	-6.4969024	-76.35857168	F	rio shilcayo	290.272	4.34819	PRJEB87706	ERS23920740

Table S2: MSCi results.

Raw (A) and scaled (B) results of the MSCi runs for each set of species. Scaling factors are shown at the top. For each replicate and parameter a median and confidence interval are shown.

**u**	2.90E-09 mutation rate subs/site/generation	**Ne**	Effective population size (x10^6)
**g**	4 generations per year	**Tdiv**	Time in years (x10^6)

A

Raw

Set	initial phi	Run		theta_1A	theta_2B	theta_3C	theta_4R	theta_5S	theta_6T	theta_7H	theta_8H	tau_4R	tau_5S	tau_6T	tau_7H	phi_H<-T	lnL
lil,ida,mar			median	0.005709	0.023926	0.006146	0.007284	0.00491	0.002574	0.014967	0.0048085	0.004639	0.003083	0.002973	0.002958	0.226106	-4104416
	0.9	rep1	2.5%HPD	0.00538	0.020673	0.00565	0.006949	0.004449	0.002282	0.003142	0.001193	0.004512	0.00298	0.002752	0.002729	0.180464	-4104588
			97.5%HPD	0.006042	0.027756	0.006813	0.007588	0.005364	0.002898	0.056334	0.03309	0.004755	0.003191	0.003104	0.00309	0.267851	-4104243
			median	0.007895	0.01357	0.006961	0.007334	0.002109	0.004215	0.001902	0.015137	0.004556	0.00244	0.003629	0.002429	0.738719	-4104328
	0.5	rep2	2.5%HPD	0.007261	0.01251	0.006462	0.007024	0.001913	0.003598	0.001543	0.003384	0.004449	0.002331	0.003512	0.00232	0.712043	-4104498
			97.5%HPD	0.008573	0.014657	0.00746	0.00765	0.002302	0.004853	0.002266	0.054689	0.004657	0.002541	0.003743	0.002532	0.764557	-4104155
			median	0.007894	0.013566	0.006964	0.007334	0.002113	0.004216	0.001902	0.0147135	0.004556	0.002439	0.00363	0.002428	0.738495	-4104336
	0.1	rep3	2.5%HPD	0.007248	0.012524	0.006479	0.007023	0.001915	0.003569	0.001546	0.003658	0.004449	0.002334	0.003508	0.002318	0.711632	-4104507
			97.5%HPD	0.008558	0.014676	0.007477	0.007645	0.002306	0.004846	0.002266	0.047249	0.004657	0.002547	0.003743	0.002533	0.76465	-4104165
			median	0.005794	0.023113	0.005998	0.007191	0.005005	0.002651	0.03758	0.035415	0.004717	0.003025	0.003016	0.003004	0.247922	-4104416
	0.9	rep4	2.5%HPD	0.005459	0.02046	0.005671	0.006881	0.004582	0.002399	0.008969	0.008048	0.004622	0.002952	0.002938	0.002929	0.218803	-4104588
			97.5%HPD	0.006127	0.02597	0.006343	0.007493	0.00546	0.002907	0.121584	0.118853	0.004813	0.003097	0.003088	0.003077	0.276993	-4104246
			median	0.0058	0.023127	0.005994	0.007193	0.005017	0.002655	0.0373215	0.035993	0.004717	0.003023	0.003014	0.003002	0.247677	-4104423
	0.5	rep5	2.5%HPD	0.005464	0.020469	0.005648	0.006891	0.004588	0.002405	0.008984	0.007798	0.004619	0.002947	0.002939	0.002925	0.219389	-4104593
			97.5%HPD	0.00613	0.025987	0.00633	0.007495	0.00546	0.002913	0.118262	0.126347	0.004808	0.003094	0.00309	0.003074	0.27737	-4104250
			median	0.005793	0.023151	0.005998	0.007194	0.00502	0.00265	0.037641	0.0362615	0.004716	0.003023	0.003014	0.003002	0.247292	-4104430
	0.1	rep6	2.5%HPD	0.005459	0.02043	0.005658	0.006895	0.004571	0.002393	0.00856	0.00829	0.004616	0.002947	0.002934	0.002924	0.218636	-4104600
			97.5%HPD	0.006131	0.025998	0.006331	0.007499	0.005473	0.002911	0.128795	0.125983	0.004808	0.003097	0.00309	0.003077	0.276438	-4104255

Set	initial phi	Run		theta_1A	theta_2B	theta_3C	theta_4R	theta_5S	theta_6T	theta_7H	theta_8H	tau_4R	tau_5S	tau_6T	tau_7H	phi_H<-T	lnL
men,mes,pol			median	0.002439	0.020932	0.022299	0.008954	0.018812	0.007279	0.0142365	0.001115	0.008334	0.005175	0.0049	0.004878	0.092437	-19212611
	0.9	rep1	2.5%HPD	0.00238	0.020105	0.02128	0.008736	0.017825	0.006922	0.003166	0.000892	0.008262	0.005101	0.004815	0.004797	0.083397	-19212942
			97.5%HPD	0.002498	0.021781	0.023362	0.009164	0.019851	0.00764	0.044824	0.001359	0.008405	0.005245	0.004983	0.004957	0.10177	-19212288
			median	0.002426	0.01634	0.022952	0.009017	0.007364	0.011995	0.002296	0.013993	0.008346	0.004629	0.006775	0.004624	0.7325595	-19212861
	0.5	rep2	2.5%HPD	0.002364	0.015831	0.021902	0.008784	0.006695	0.011003	0.001965	0.003448	0.008265	0.004516	0.006666	0.004514	0.712119	-19213192
			97.5%HPD	0.002485	0.016849	0.024044	0.009231	0.008036	0.013005	0.002633	0.041549	0.008421	0.004729	0.006879	0.004727	0.752037	-19212533
			median	0.002426	0.016341	0.022938	0.00902	0.007367	0.011996	0.002285	0.014879	0.008345	0.004634	0.006773	0.004629	0.732153	-19212854
	0.1	rep3	2.5%HPD	0.002363	0.015824	0.02186	0.008793	0.006714	0.010977	0.001937	0.00325	0.008266	0.004512	0.006661	0.004504	0.71147	-19213180
			97.5%HPD	0.002483	0.016838	0.02402	0.009236	0.008045	0.012987	0.002632	0.048943	0.008421	0.004742	0.006875	0.004735	0.752086	-19212524
			median	0.00243	0.016313	0.023064	0.009038	0.007238	0.012048	0.002547	0.0356325	0.008335	0.004572	0.006801	0.004566	0.7427165	-19212828
	0.9	rep4	2.5%HPD	0.002367	0.015817	0.021985	0.008812	0.006589	0.011024	0.002231	0.009034	0.008256	0.004467	0.006694	0.004463	0.724138	-19213156
			97.5%HPD	0.002486	0.016831	0.024161	0.009258	0.007911	0.013052	0.002866	0.106088	0.008412	0.004677	0.006901	0.004674	0.761274	-19212502
			median	0.00243	0.016312	0.02307	0.009036	0.00724	0.012046	0.002556	0.0407865	0.008335	0.004568	0.006802	0.004562	0.742936	-19212823
	0.5	rep5	2.5%HPD	0.002369	0.015803	0.022021	0.008809	0.006589	0.011013	0.002219	0.009558	0.008257	0.004462	0.006695	0.004457	0.723981	-19213156
			97.5%HPD	0.002489	0.01681	0.024178	0.00926	0.007906	0.013063	0.002881	0.147218	0.008414	0.004676	0.006902	0.004671	0.761847	-19212497
			median	0.00243	0.016315	0.023064	0.009035	0.007249	0.012054	0.002546	0.039615	0.008336	0.004571	0.006799	0.004565	0.742421	-19212816
	0.1	rep6	2.5%HPD	0.002373	0.015814	0.021982	0.008807	0.00661	0.011063	0.002229	0.00894	0.008255	0.004458	0.006694	0.00446	0.723496	-19213153
			97.5%HPD	0.002492	0.016828	0.024154	0.009255	0.007928	0.013083	0.002878	0.114832	0.008412	0.004673	0.0069	0.004676	0.761143	-19212494

Set	initial phi	Run		theta_1A	theta_2B	theta_3C	theta_4R	theta_5S	theta_6T	theta_7H	theta_8H	tau_4R	tau_5S	tau_6T	tau_7H	phi_H<-T	lnL
pol,nes,lys			median	0.040143	0.017117	0.007383	0.006955	0.003729	0.00978	0.001525	0.014472	0.006226	0.004193	0.005037	0.004185	0.92675	-19413289
	0.9	rep1	2.5%HPD	0.037547	0.016539	0.007168	0.0068	0.003518	0.009032	0.001319	0.003476	0.006178	0.004113	0.004962	0.004107	0.917633	-19413635
			97.5%HPD	0.042721	0.017712	0.007598	0.007107	0.003931	0.010544	0.001727	0.046269	0.006277	0.004267	0.005108	0.004262	0.935551	-19412936
			median	0.040165	0.017115	0.007383	0.006955	0.003731	0.009783	0.001528	0.014533	0.006226	0.004191	0.005038	0.004183	0.926784	-19413297
	0.5	rep2	2.5%HPD	0.037595	0.016546	0.007167	0.006798	0.003514	0.009022	0.001337	0.003526	0.006178	0.004115	0.004963	0.004107	0.91768	-19413648
			97.5%HPD	0.042764	0.017718	0.007597	0.007108	0.003934	0.010528	0.001731	0.041393	0.006277	0.004271	0.005108	0.004265	0.935586	-19412948
			median	0.040185	0.017115	0.007382	0.006953	0.003735	0.009774	0.001531	0.014828	0.006227	0.004191	0.005038	0.004183	0.926875	-19413294
	0.1	rep3	2.5%HPD	0.037622	0.016518	0.007171	0.006799	0.00353	0.009046	0.001329	0.003759	0.006178	0.004109	0.004966	0.004098	0.917832	-19413642
			97.5%HPD	0.042817	0.01769	0.007598	0.007111	0.003944	0.010525	0.001741	0.045361	0.006277	0.004268	0.00511	0.004259	0.935698	-19412939
			median	0.040121	0.017115	0.007381	0.006961	0.00372	0.009788	0.001521	0.014974	0.006224	0.004196	0.005036	0.004188	0.9267475	-19413277
	0.9	rep4	2.5%HPD	0.037567	0.016547	0.00717	0.006803	0.003516	0.009048	0.001305	0.003658	0.006174	0.00411	0.004962	0.004102	0.91767	-19413630
			97.5%HPD	0.042766	0.017712	0.007593	0.00711	0.003938	0.010554	0.001728	0.045614	0.006273	0.004271	0.005108	0.004265	0.935572	-19412928
			median	0.040155	0.01712	0.007382	0.006953	0.003729	0.009786	0.001527	0.0140535	0.006226	0.004193	0.005037	0.004185	0.926724	-19413282
	0.5	rep5	2.5%HPD	0.037645	0.016532	0.007175	0.006801	0.00352	0.009043	0.001312	0.003844	0.006177	0.004113	0.004963	0.004106	0.917785	-19413627
			97.5%HPD	0.04281	0.017702	0.007599	0.00711	0.003938	0.010541	0.001733	0.039683	0.006276	0.004271	0.005108	0.004265	0.935799	-19412921
			median	0.040155	0.017117	0.007384	0.006953	0.00373	0.009781	0.001526	0.01534	0.006227	0.004193	0.005037	0.004185	0.926719	-19413289
	0.1	rep6	2.5%HPD	0.037642	0.016537	0.007169	0.006797	0.003515	0.009007	0.001317	0.003356	0.006175	0.004107	0.004963	0.004098	0.917582	-19413643
			97.5%HPD	0.042858	0.017709	0.007597	0.007117	0.003952	0.010523	0.001741	0.050939	0.006277	0.004272	0.00511	0.004265	0.935588	-19412939

B

Scaled

Set	initial phi	Run	Stat	Ne_1A	Ne_2B	Ne_3C	Ne_4R	Ne_5S	Ne_6T	Ne_7H	Ne_8H	Tdiv_4R	Tdiv_5S	Tdiv_6T	Tdiv_7H	phi_H<-T	lnL
lil,ida,mar			**median**	**0.49**	**2.06**	**0.53**	**0.63**	**0.42**	**0.22**	**1.29**	**0.41**	**0.400**	**0.266**	**0.256**	**0.255**	**22.6%**	
	0.9	rep1	**2.5%HPD**	**0.46**	**1.78**	**0.49**	**0.60**	**0.38**	**0.20**	**0.27**	**0.10**	**0.389**	**0.257**	**0.237**	**0.235**	**18.0%**	-4,104,243
			**97.5%HPD**	**0.52**	**2.39**	**0.59**	**0.65**	**0.46**	**0.25**	**4.86**	**2.85**	**0.410**	**0.275**	**0.268**	**0.266**	**26.8%**	
			**median**	**0.68**	**1.17**	**0.60**	**0.63**	**0.18**	**0.36**	**0.16**	**1.30**	**0.393**	**0.210**	**0.313**	**0.209**	**73.9%**	
	0.5	rep2	**2.5%HPD**	**0.63**	**1.08**	**0.56**	**0.61**	**0.16**	**0.31**	**0.13**	**0.29**	**0.384**	**0.201**	**0.303**	**0.200**	**71.2%**	-4,104,155
			**97.5%HPD**	**0.74**	**1.26**	**0.64**	**0.66**	**0.20**	**0.42**	**0.20**	**4.71**	**0.401**	**0.219**	**0.323**	**0.218**	**76.5%**	
			median	0.68	1.17	0.60	0.63	0.18	0.36	0.16	1.27	0.39	0.21	0.31	0.21	73.8%	
	0.1	rep3	2.5%HPD	0.62	1.08	0.56	0.61	0.17	0.31	0.13	0.32	0.38	0.20	0.30	0.20	71.2%	-4,104,165
			97.5%HPD	0.74	1.27	0.64	0.66	0.20	0.42	0.20	4.07	0.40	0.22	0.32	0.22	76.5%	
			median	0.50	1.99	0.52	0.62	0.43	0.23	3.24	3.05	0.41	0.26	0.26	0.26	24.8%	
	0.9	rep4	2.5%HPD	0.47	1.76	0.49	0.59	0.40	0.21	0.77	0.69	0.40	0.25	0.25	0.25	21.9%	-4,104,246
			97.5%HPD	0.53	2.24	0.55	0.65	0.47	0.25	10.48	10.25	0.41	0.27	0.27	0.27	27.7%	
			median	0.50	1.99	0.52	0.62	0.43	0.23	3.22	3.10	0.41	0.26	0.26	0.26	24.8%	
	0.5	rep5	2.5%HPD	0.47	1.76	0.49	0.59	0.40	0.21	0.77	0.67	0.40	0.25	0.25	0.25	21.9%	-4,104,250
			97.5%HPD	0.53	2.24	0.55	0.65	0.47	0.25	10.20	10.89	0.41	0.27	0.27	0.27	27.7%	
			median	0.50	2.00	0.52	0.62	0.43	0.23	3.24	3.13	0.41	0.26	0.26	0.26	24.7%	
	0.1	rep6	2.5%HPD	0.47	1.76	0.49	0.59	0.39	0.21	0.74	0.71	0.40	0.25	0.25	0.25	21.9%	-4,104,255
			97.5%HPD	0.53	2.24	0.55	0.65	0.47	0.25	11.10	10.86	0.41	0.27	0.27	0.27	27.6%	

Set	initial phi	Run	Stat	Ne_1A	Ne_2B	Ne_3C	Ne_4R	Ne_5S	Ne_6T	Ne_7H	Ne_8H	Tdiv_4R	Tdiv_5S	Tdiv_6T	Tdiv_7H	phi_H<-T	lnL
men,mes,pol			**median**	**0.21**	**1.80**	**1.92**	**0.77**	**1.62**	**0.63**	**1.23**	**0.10**	**0.718**	**0.446**	**0.422**	**0.421**	**9.2%**	
	0.9	rep1	**2.5%HPD**	**0.21**	**1.73**	**1.83**	**0.75**	**1.54**	**0.60**	**0.27**	**0.08**	**0.712**	**0.440**	**0.415**	**0.414**	**8.3%**	-19,212,611
			**97.5%HPD**	**0.22**	**1.88**	**2.01**	**0.79**	**1.71**	**0.66**	**3.86**	**0.12**	**0.725**	**0.452**	**0.430**	**0.427**	**10.2%**	
			median	0.21	1.41	1.98	0.78	0.63	1.03	0.20	1.21	0.719	0.399	0.584	0.399	73.3%	
	0.5	rep2	2.5%HPD	0.20	1.36	1.89	0.76	0.58	0.95	0.17	0.30	0.713	0.389	0.575	0.389	71.2%	-19,212,861
			97.5%HPD	0.21	1.45	2.07	0.80	0.69	1.12	0.23	3.58	0.726	0.408	0.593	0.408	75.2%	
			median	0.21	1.41	1.98	0.78	0.64	1.03	0.20	1.28	0.719	0.399	0.584	0.399	73.2%	
	0.1	rep3	2.5%HPD	0.20	1.36	1.88	0.76	0.58	0.95	0.17	0.28	0.713	0.389	0.574	0.388	71.1%	-19,212,854
			97.5%HPD	0.21	1.45	2.07	0.80	0.69	1.12	0.23	4.22	0.726	0.409	0.593	0.408	75.2%	
			median	0.21	1.41	1.99	0.78	0.62	1.04	0.22	3.07	0.719	0.394	0.586	0.394	74.3%	
	0.9	rep4	2.5%HPD	0.20	1.36	1.90	0.76	0.57	0.95	0.19	0.78	0.712	0.385	0.577	0.385	72.4%	-19,212,828
			97.5%HPD	0.21	1.45	2.08	0.80	0.68	1.13	0.25	9.15	0.725	0.403	0.595	0.403	76.1%	
			median	0.21	1.41	1.99	0.78	0.62	1.04	0.22	3.52	0.719	0.394	0.586	0.393	74.3%	
	0.5	rep5	2.5%HPD	0.20	1.36	1.90	0.76	0.57	0.95	0.19	0.82	0.712	0.385	0.577	0.384	72.4%	-19,212,823
			97.5%HPD	0.21	1.45	2.08	0.80	0.68	1.13	0.25	12.69	0.725	0.403	0.595	0.403	76.2%	
			**median**	**0.21**	**1.41**	**1.99**	**0.78**	**0.62**	**1.04**	**0.22**	**3.42**	**0.719**	**0.394**	**0.586**	**0.394**	**74.2%**	
	0.1	rep6	**2.5%HPD**	**0.20**	**1.36**	**1.90**	**0.76**	**0.57**	**0.95**	**0.19**	**0.77**	**0.712**	**0.384**	**0.577**	**0.384**	**72.3%**	**-19,212,816**
			**97.5%HPD**	**0.21**	**1.45**	**2.08**	**0.80**	**0.68**	**1.13**	**0.25**	**9.90**	**0.725**	**0.403**	**0.595**	**0.403**	**76.1%**	

Set	initial phi	Run	Stat	Ne_1A	Ne_2B	Ne_3C	Ne_4R	Ne_5S	Ne_6T	Ne_7H	Ne_8H	Tdiv_4R	Tdiv_5S	Tdiv_6T	Tdiv_7H	phi_H<-T	lnL
pol,nes,lys			median	3.46	1.48	0.64	0.60	0.32	0.84	0.13	1.25	0.537	0.361	0.434	0.361	92.68%	
	0.9	rep1	2.5%HPD	3.24	1.43	0.62	0.59	0.30	0.78	0.11	0.30	0.533	0.355	0.428	0.354	91.76%	-19,413,289
			97.5%HPD	3.68	1.53	0.66	0.61	0.34	0.91	0.15	3.99	0.54	0.37	0.44	0.37	93.56%	
			median	3.46	1.48	0.64	0.60	0.32	0.84	0.13	1.25	0.537	0.361	0.434	0.361	92.68%	
	0.5	rep2	2.5%HPD	3.24	1.43	0.62	0.59	0.30	0.78	0.12	0.30	0.533	0.355	0.428	0.354	91.77%	-19,413,297
			97.5%HPD	3.69	1.53	0.65	0.61	0.34	0.91	0.15	3.57	0.54	0.37	0.44	0.37	93.56%	
			median	3.46	1.48	0.64	0.60	0.32	0.84	0.13	1.28	0.537	0.361	0.434	0.361	92.69%	
	0.1	rep3	2.5%HPD	3.24	1.42	0.62	0.59	0.30	0.78	0.11	0.32	0.533	0.354	0.428	0.353	91.78%	-19,413,294
			97.5%HPD	3.69	1.53	0.66	0.61	0.34	0.91	0.15	3.91	0.54	0.37	0.44	0.37	93.57%	
			**median**	**3.46**	**1.48**	**0.64**	**0.60**	**0.32**	**0.84**	**0.13**	**1.29**	**0.537**	**0.362**	**0.434**	**0.361**	**92.67%**	
	0.9	rep4	**2.5%HPD**	**3.24**	**1.43**	**0.62**	**0.59**	**0.30**	**0.78**	**0.11**	**0.32**	**0.532**	**0.354**	**0.428**	**0.354**	**91.77%**	-19,413,277
			**97.5%HPD**	**3.69**	**1.53**	**0.65**	**0.61**	**0.34**	**0.91**	**0.15**	**3.93**	**0.54**	**0.37**	**0.44**	**0.37**	**93.56%**	
			median	3.46	1.48	0.64	0.60	0.32	0.84	0.13	1.21	0.537	0.361	0.434	0.361	92.67%	
	0.5	rep5	2.5%HPD	3.25	1.43	0.62	0.59	0.30	0.78	0.11	0.33	0.533	0.355	0.428	0.354	91.78%	-19,413,282
			97.5%HPD	3.69	1.53	0.66	0.61	0.34	0.91	0.15	3.42	0.54	0.37	0.44	0.37	93.58%	
			median	3.46	1.48	0.64	0.60	0.32	0.84	0.13	1.32	0.537	0.361	0.434	0.361	92.67%	
	0.1	rep6	2.5%HPD	3.25	1.43	0.62	0.59	0.30	0.78	0.11	0.29	0.532	0.354	0.428	0.353	91.76%	-19,413,289
			97.5%HPD	3.69	1.53	0.65	0.61	0.34	0.91	0.15	4.39	0.54	0.37	0.44	0.37	93.56%	

Table S3: Details of the androconial chemicals

ID	Endemism Centre	Municipality / State	Sampling Area	Sampling altitude (m)	1150	1252	1264	1318	1365	1481	1543	1551	1589	1599	1619	1673	1749	1792	1804	1843	1852	1950	2145	2162	2172	2220	2572	2675	2832	2873
M.polymnia662	Serra do Mar	Tremembé / SP	Urban Park	500-550	0	0	0	1.81439521	0	83.6593395	0	0	10.4149599	0	0	0.17222797	0	0	0	0	0	0.79566418	0	0	0	1.15038913	0	0	1.99302409	0
M.polymnia663	Serra do Mar	Tremembé / SP	Urban Park	500-550	0	0	0	1.94328697	0	63.7618607	0	0	13.3624124	0	0	0	0	0	0	0	0	1.24065814	0	0	8.8986205	5.53252409	0	0	5.26063714	0
M.polymnia664	Serra do Mar	Tremembé / SP	Urban Park	500-550	0	0	0	2.9964548	0	77.2110151	0	0	13.1212435	0	0	0.35608499	0	0	0	0	0	1.846734	0	0	0	2.3477962	0	0	2.12067133	0
M.polymnia665	Serra do Mar	Tremembé / SP	Urban Park	500-550	0	0	0	5.67371665	0	73.217209	0	0	11.8430092	0	0	0	0	0	0	0	0	1.80468429	0	0	3.2520484	2.55198207	0	0	1.65735041	0
M.polymnia667	Serra do Mar	Tremembé / SP	Urban Park	500-550	0	0	0	2.19323793	0	76.7622863	0	0	9.31193319	0	0	0	0	0	0	0	0	1.06136489	0	0	0	2.2846597	0	0	8.38651798	0
M.polymnia833	Bahia	Camacã / BA	Serra Bonita Private Reserve	900-1200	0	0	0	9.36687735	0	64.0583884	0	0	18.3922813	0	0	0.73056347	0	0	0	0	0	0	0	0	0	5.06884983	0	0	2.38303962	0
M.polymnia854	Bahia	Camacã / BA	Serra Bonita Private Reserve	900-1200	0	0	0	0	0	72.4732005	0	0	14.7582432	0	0	0.4780874	0	0	0	0	0	0	0	0	3.83463556	3.28049463	0	0	5.17533874	0
M.polymnia900	Bahia	Camacã / BA	Serra Bonita Private Reserve	900-1200	0	0	0	1.41084366	0	86.0571083	0	0	9.54388731	0	0	0.47157343	0	0	0	0	0	0.90067489	0	0	0	1.03601031	0	0	0.57990212	0
M.nesaea020	Pernambuco	Recife / PE	Dois Irmãos State Park	0-90	1.52572254	0	0	5.24432672	0	44.5626369	0	0	10.1267351	5.58784774	1.59271975	0	31.3600112	0	0	0	0	0	0	0	0	0	0	0	0	0
M.nesaea035	Pernambuco	Recife / PE	Dois Irmãos State Park	0-90	0.33821466	0.13306722	0.08442876	1.54591636	0	48.668333	0	0	26.1962521	0.97397827	0	0	10.8023542	5.58187185	0	0	0	0	3.26903446	2.40654909	0	0	0	0	0	0
M.nesaea036	Pernambuco	Recife / PE	Dois Irmãos State Park	0-90	0.31349946	0.14552659	0.11681349	1.4956552	0	72.1367891	0	0	12.6474082	0.71529957	0	0	4.43678864	4.06444378	0	0	0	0	1.97862165	1.94915426	0	0	0	0	0	0
M.nesaea038	Pernambuco	Recife / PE	Dois Irmãos State Park	0-90	0.15338311	0.18760495	0.13020199	2.59710595	0	15.5581534	0	0	45.0832855	1.90221249	0	0	10.6471401	15.6697098	0	0	0	0	4.68990615	3.3812965	0	0	0	0	0	0
M.nesaea039	Pernambuco	Recife / PE	Dois Irmãos State Park	0-90	0.10870219	0.03930754	0.0291563	0.7355774	0	79.9825674	0	0	10.8642061	0.79703566	0.16757587	0	2.47709684	1.26979241	0	0	0	0	1.75651129	1.77247102	0	0	0	0	0	0
M.nesaea040	Pernambuco	Recife / PE	Dois Irmãos State Park	0-90	0.20016814	0.14663471	0.06540147	2.03564319	0	50.3294291	0	0	18.2613387	0.88415731	0.5889274	0	9.28768588	6.70928124	0	0	0	0	7.78531065	3.70602225	0	0	0	0	0	0
M.nesaea043	Pernambuco	Recife / PE	Dois Irmãos State Park	0-90	0.5902113	0.18628157	0.05506498	2.41954446	0	31.3911179	0	0	32.0312501	1.0186932	0.80223424	0	7.74394019	10.2370252	0	0	0	0	8.65055345	4.87408334	0	0	0	0	0	0
M.nesaea044	Pernambuco	Recife / PE	Dois Irmãos State Park	0-90	0.55445468	0.1612009	0.47202586	1.75752794	0	25.0544892	0	0	39.7925166	0.96280315	0.62234884	0	9.15891223	7.75568651	0	0	0	0	10.2535338	3.45450034	0	0	0	0	0	0
M.nesaea045	Pernambuco	Recife / PE	Dois Irmãos State Park	0-90	0.23157719	0.10939794	0.07978331	1.24165094	0	74.4950508	0	0	14.5900225	0.6038832	0.33231768	0	3.88687802	2.38655513	0	0	0	0	1.28170406	0.76117922	0	0	0	0	0	0
M.nesaea046	Pernambuco	Recife / PE	Dois Irmãos State Park	0-90	0.18724146	0.08390034	0	1.75335352	0	42.5794399	0	0	30.8738894	1.06610547	0.39054728	0	5.63316346	5.15455461	0	0	0	0	8.34567795	3.93212669	0	0	0	0	0	0
M.nesaea087	Pernambuco	Jaqueira / PE	Frei Caneca Private Reserve	65-750	0	0	0	0	0	46.5063357	0	0	25.429765	2.59915127	0	0	4.97726985	9.41545418	0	0	0	0	3.99685721	7.07516681	0	0	0	0	0	0
M.nesaea102	Pernambuco	Jaqueira / PE	Frei Caneca Private Reserve	65-750	0	0.14464449	0	0.91308019	0	50.6636059	0	0	26.7843712	0	0	0	10.9851887	7.9535418	1.62188134	0.93368629	0	0	0	0	0	0	0	0	0	0
M.nesaea118	Pernambuco	Jaqueira / PE	Frei Caneca Private Reserve	65-750	0	0	0	0	0	81.1633652	0	0	17.1518647	0	0	0	0	0.41583589	0	1.26893418	0	0	0	0	0	0	0	0	0	0
M.nesaea202	Pernambuco	Jaqueira / PE	Frei Caneca Private Reserve	65-750	0	0	0	0	0	92.2113636	0	0	0	0	5.8348718	0	0	0	0	0	0	0	0	1.95376459	0	0	0	0	0	0
M.nesaea203	Pernambuco	Jaqueira / PE	Frei Caneca Private Reserve	65-750	0	0	0	0	0	53.8744099	0	0	33.3416348	0	0	0	8.18048358	3.53078494	0	1.07268675	0	0	0	0	0	0	0	0	0	0
M.nesaea221	Pernambuco	Jaqueira / PE	Frei Caneca Private Reserve	65-750	0	0	0	0.27993289	0	93.6045275	0	0	0	0	0	0	1.67288721	0	0	0	3.94576115	0	0	0.49689124	0	0	0	0	0	0
M.nesaea222	Pernambuco	Jaqueira / PE	Frei Caneca Private Reserve	65-750	0	0	0	0.18136872	0	70.4832876	0	0	19.2036469	0	0	0	3.86227704	4.50174246	0	0.97324084	0	0	0.34168013	0.45275629	0	0	0	0	0	0
M.nesaea234	Pernambuco	Jaqueira / PE	Frei Caneca Private Reserve	65-750	0	0	0	4.9714E-08	0	59.6803655	0	0	26.6567765	0	0	0	7.51637575	4.13183701	0	0.18914715	0	0	1.42623378	0.39926428	0	0	0	0	0	0
M.nesaea235	Pernambuco	Jaqueira / PE	Frei Caneca Private Reserve	65-750	0	0	0	0.95068788	0	60.6470534	0	0	11.367888	0	0	0	11.3809504	12.1622495	2.10827498	1.38289585	0	0	0	0	0	0	0	0	0	0
M.nesaea236	Pernambuco	Jaqueira / PE	Frei Caneca Private Reserve	65-750	0	0.36932291	0	2.53410742	0	68.9102389	0	0	7.8613312	0	0	0	8.37015876	10.9336561	0	1.02118472	0	0	0	0	0	0	0	0	0	0
M.nesaea533	Pernambuco	São Lourenço da Ma	Tapacurá Ecological Station	90-200	0.17159864	0	0	0.53690802	0	54.9852364	0	0	19.8689227	0	0	0	3.15558407	6.57707692	1.87827533	0.69560409	0	0	10.0700481	2.06074575	0	0	0	0	0	0
M.nesaea536	Pernambuco	São Lourenço da Ma	Tapacurá Ecological Station	90-200	0	0	0	0.15454465	0	80.3805376	0	0.4203331	10.3003392	0	0.25796396	0	2.04237404	1.39763109	0.24334424	0.26952226	0	0	3.61897201	0.9144378	0	0	0	0	0	0
M.nesaea537	Pernambuco	São Lourenço da Ma	Tapacurá Ecological Station	90-200	0.17758948	0	0	0.51496119	0	82.2010896	0	0.29852666	7.95694948	0	0.14295581	0	2.54203843	1.43715413	0	0	0	0	3.55634453	1.17239069	0	0	0	0	0	0
M.nesaea538	Pernambuco	São Lourenço da Ma	Tapacurá Ecological Station	90-200	0	0	0	0.24257932	0	72.3208027	0	0.54985388	11.3750309	0	0.23403757	0	4.05225666	3.26565574	0	0.46672734	0	0	5.79222339	1.70083249	0	0	0	0	0	0
M.nesaea539	Pernambuco	São Lourenço da Ma	Tapacurá Ecological Station	90-200	0	0	0	0.54563051	0	66.5919142	0	1.26983502	17.448529	0	0	0	4.31080425	5.47475637	1.40535676	0	0	0	1.89082447	1.06234948	0	0	0	0	0	0
M.nesaea540	Pernambuco	São Lourenço da Ma	Tapacurá Ecological Station	90-200	0	0	0	0.27128333	0	76.9589273	0	0	10.1985806	0	0.38672858	0	1.88102054	3.5339065	0.2546119	0.62638047	0	0	5.72648277	0.16207804	0	0	0	0	0	0
M.nesaea541	Pernambuco	São Lourenço da Ma	Tapacurá Ecological Station	90-200	0	0	0	0.18157334	0	57.7408749	0	0	20.5707512	0	0.47767806	0	2.53068235	4.17170471	2.5712897	0	0	0	9.88407036	1.87137534	0	0	0	0	0	0
M.nesaea542	Pernambuco	São Lourenço da Ma	Tapacurá Ecological Station	90-200	0	0	0	1.11709317	0.32362685	68.9265684	0	0	11.9678201	0	0.4064465	0	4.66185672	6.95795162	0.71054373	0.57704831	0	0	2.77277846	1.57826609	0	0	0	0	0	0
M.nesaea543	Pernambuco	São Lourenço da Ma	Tapacurá Ecological Station	90-200	0.12423537	0	0	0.21131605	0.09539051	61.5862993	0	0.38092025	12.9809013	0	0.36693071	0	2.28850126	1.85429498	1.00629475	0.28209576	0	0	15.9485028	2.87431691	0	0	0	0	0	0
M.nesaea544	Pernambuco	São Lourenço da Ma	Tapacurá Ecological Station	90-200	0	0	0	0.26835783	0.16284207	76.3000568	0	0.29209251	7.83703832	0	0.2030904	0	2.46034377	4.65597284	0.3618209	0.36424184	0	0	5.01040837	2.08373435	0	0	0	0	0	0
M.nesaea545	Pernambuco	São Lourenço da Ma	Tapacurá Ecological Station	90-200	0	0	0	0.75262587	0.17674278	74.01797	0	0.49784931	11.8039213	0	0.3675869	0	2.81624867	7.47172875	0.18717271	0.67701446	0	0	0.85010156	0.38103776	0	0	0	0	0	0
M.nesaea277	Pernambuco	Igarassu / PE	Usina São José	45-150	0	0	0	0.05634414	0	77.7230607	0	1.07195327	13.9293281	0	0	0	2.78586211	3.85382121	0	0.57963049	0	0	0	0	0	0	0	0	0	0
M.nesaea320	Pernambuco	Igarassu / PE	Usina São José	45-150	0	0	0	0.28043298	0	55.9353938	0	0.35165242	27.7710039	0	0	0	5.35201552	6.56824979	2.24475303	1.49649861	0	0	0	0	0	0	0	0	0	0
M.nesaea496	Pernambuco	Igarassu / PE	Usina São José	45-150	0	0	0	0.70203145	0	60.4550456	0	1.46176474	23.2237326	0	0	0	5.95989483	3.57466127	0	2.20209271	0	0	1.1694511	1.25132571	0	0	0	0	0	0
M.nesaea500	Pernambuco	Igarassu / PE	Usina São José	45-150	0	0	0	0.89808957	0	66.9952625	0	0.52823674	15.4414509	0	0	0	6.83414799	4.84191571	0	2.57491844	0	0	1.30796172	0.57801641	0	0	0	0	0	0
M.nesaea505	Pernambuco	Igarassu / PE	Usina São José	45-150	0	0	0	0.45685696	0	70.701603	0	0.65172337	15.7417586	0	0	0	4.19645369	3.44652309	0.47547359	1.70939902	0	0	1.56804338	1.05216535	0	0	0	0	0	0
M.nesaea507	Pernambuco	Igarassu / PE	Usina São José	45-150	0	0	0	0.90966346	0	63.8239869	0	0.40268144	11.8497254	0	0	0	4.33811877	5.72234753	0.45652846	1.170253	0	0	6.74923171	4.5774633	0	0	0	0	0	0
M.nesaea508	Pernambuco	Igarassu / PE	Usina São José	45-150	0	0	0	1.32613139	0	59.1077337	0	0.54179944	27.9673951	0	0	0	3.27765349	1.58565487	0	3.17303925	0	0	1.99220816	1.02838466	0	0	0	0	0	0
M.nesaea509	Pernambuco	Igarassu / PE	Usina São José	45-150	0	0	0	1.09334491	0	62.594211	0	0.54933791	21.0720504	0	0	0	2.8466889	2.24611469	0	2.49399752	0	0	4.44371797	2.66053665	0	0	0	0	0	0
M.nesaea510	Pernambuco	Igarassu / PE	Usina São José	45-150	0	0	0	0.94581047	0	63.452466	0	0.28478783	14.0836797	0	0	0	5.2454887	4.99973604	0.8713368	2.69204388	0	0	4.13124903	3.29340156	0	0	0	0	0	0
M.nesaea552	Pernambuco	Igarassu / PE	Usina São José	45-150	0	0	0	2.00585217	8.65E-07	37.4260056	0	8.65E-07	23.9752062	0.84601945	0	0	4.42277181	22.0483151	0	0	0	0	3.96708827	5.30873977	0	0	0	0	0	0
M.nesaea551	Pernambuco	Igarassu / PE	Usina São José	45-150	0	0	0	0.20365179	0.69331635	44.463291	0	3.6827E-07	24.0967616	0.69772237	0	0	7.78218469	7.44895376	0	0	0	0	7.86883865	6.74527935	0	0	0	0	0	0
M.nesaea388	Brejos Nordestinos	Caruara / PE	Serro dos Cavalos Municipal Park	780-950	0	0	0	0	0	16.1119837	0	3.72837702	22.1410509	0	0	0	12.1463365	4.67309381	0	3.54555642	0	0	18.1891243	19.4644773	0	0	0	0	0	0
M.nesaea390	Brejos Nordestinos	Caruara / PE	Serro dos Cavalos Municipal Park	780-950	0	0	0	0	0	96.8852822	0	1.81226163	0	0	0	0	0	0	0	0	0	0	0	1.30245618	0	0	0	0	0	0
M.nesaea394	Brejos Nordestinos	Caruara / PE	Serro dos Cavalos Municipal Park	780-950	0	0	0	0.18225358	0	62.8233766	0	1.37105538	27.628429	0	0	0	2.48969723	3.71747642	0.16286055	1.62485123	0	0	0	0	0	0	0	0	0	0
M.nesaea395	Brejos Nordestinos	Caruara / PE	Serro dos Cavalos Municipal Park	780-950	0	0	0	0.49473308	0	64.9160115	0	1.92919768	23.4516103	0	0	0	2.9861818	4.39505189	0	1.82721384	0	0	0	0	0	0	0	0	0	0
M.nesaea396	Brejos Nordestinos	Caruara / PE	Serro dos Cavalos Municipal Park	780-950	0	0	0	0.25437375	0	98.9859345	0	0.7596918	0	0	0	0	0	0	0	0	0	0	0	0	0	0	0	0	0	0
M.nesaea397	Brejos Nordestinos	Caruara / PE	Serro dos Cavalos Municipal Park	780-950	0	0	0	0.65521053	0	82.3308724	0	0.63471038	8.64324002	0	0	0	2.99146168	3.75125446	0	0.99325054	0	0	0	0	0	0	0	0	0	0
M.nesaea398	Brejos Nordestinos	Caruara / PE	Serro dos Cavalos Municipal Park	780-950	0	0	0	0.21029195	0	77.6793295	0	1.64913193	13.7271934	0	0	0	2.61140116	3.62859151	2.0052E-07	0.49406037	0	0	0	0	0	0	0	0	0	0
M.nesaea399	Brejos Nordestinos	Caruara / PE	Serro dos Cavalos Municipal Park	780-950	0	0	0	0	0	82.2137399	0	1.25944171	10.4731659	0	0	0	3.00554347	2.5148674	0	0.53324166	0	0	0	0	0	0	0	0	0	0
M.nesaea405	Brejos Nordestinos	Caruara / PE	Serro dos Cavalos Municipal Park	780-950	0	0	0	0.67301442	0	67.3947729	0	1.58340446	18.8843408	0	0	0	7.05015909	3.37927139	0	1.03503695	0	0	0	0	0	0	0	0	0	0
M.nesaea406	Brejos Nordestinos	Caruara / PE	Serro dos Cavalos Municipal Park	780-950	0	0	0	0	0	72.8401964	0	0.78541377	20.4889964	0	0	0	2.49715257	1.56941933	0	1.17777363	0	0	0.42963048	0.21141749	0	0	0	0	0	0
M.nesaea_BA839	Bahia	Camacã / BA	Serra Bonita Private Reserve	900-1200	8.0992228	0	0	5.30904006	0	63.7847457	0	0	11.9499554	0	0	0	0	0	0	0	0	0	0	10.857036	0	0	0	0	0	0
M.nesaea_BA859	Bahia	Camacã / BA	Serra Bonita Private Reserve	900-1200	0	0	0	0.81582799	1.62021591	45.2250879	0	3.39706368	24.5555012	0	0	0	7.6459486	6.30211393	0	2.10563671	0	0	0	7.92558821	0	0	0	0.4070159	0	0
M.nesaea_BA860	Bahia	Camacã / BA	Serra Bonita Private Reserve	900-1200	0	0	0	0.25579374	0	45.5849012	0	1.61585511	23.1059642	0	0	0	3.5116344	5.18359937	0	0.89076369	0	0	10.7065303	8.75543942	0	0	0	0.38951844	0	0
M.nesaea_BA867	Bahia	Camacã / BA	Serra Bonita Private Reserve	900-1200	1.30278721	0	0	0.76998378	0	63.8785459	0	0.7692554	10.3407428	0	0	0	5.7143533	4.93774402	0	1.29358238	0	0	5.9372679	5.05573724	0	0	0	0	0	0
M.nesaea_BA870	Bahia	Camacã / BA	Serra Bonita Private Reserve	900-1200	0	0	0	0	0	53.9770754	2.3678E-07	3.98959814	25.5440064	0	0	0	4.14023545	4.06639219	0	0	0	0	2.89081409	5.39187807	0	0	0	0	0	0
M.nesaea_BA896	Bahia	Camacã / BA	Serra Bonita Private Reserve	900-1200	0	0.28507915	0	1.59769373	0	54.9955068	1.0769E-07	7.55753194	13.8430969	0	0	0	4.17280083	1.8375579	0	1.23024519	0	0	6.41772642	8.0627611	0	0	0	0	0	0
M.nesaea_BA901	Bahia	Camacã / BA	Serra Bonita Private Reserve	900-1200	0	0	0	0.62798312	0	67.9284588	1.9509E-07	1.9509E-07	20.6426119	0	0	0	5.32497448	3.36597346	0	0.79823777	0	0	1.9509E-07	1.3117599	0	0	0	0	0	0
M.nesaea_BA902	Bahia	Camacã / BA	Serra Bonita Private Reserve	900-1200	0	0	0	0.32148326	0	57.5366063	7.8267E-08	3.53543235	25.8334977	0	0	0	3.91160995	2.06339339	0	1.18335986	0	0	2.89581243	2.71880463	0	0	0	0	0	0
M.nesaea_BA903	Bahia	Camacã / BA	Serra Bonita Private Reserve	900-1200	0	0	0	0.62511315	7.8446E-08	54.5306714	7.8446E-08	1.72716156	16.5301704	0	0	0	4.28834424	2.37328454	0	1.08924402	0	0	9.14067746	9.69533304	0	0	0	0	0	0
M.nesaea_BA904	Bahia	Camacã / BA	Serra Bonita Private Reserve	900-1200	0	0	0	0.47785162	0	47.7019442	3.529E-07	4.22305855	20.7101864	0	0	0	14.9425637	8.36740363	0	0	0	0	0	3.57699156	0	0	0	0	0	0
M.nesaea_BA905	Bahia	Camacã / BA	Serra Bonita Private Reserve	900-1200	0	0	0	1.58043559	0	64.7703745	4.805E-07	4.99377903	9.88951589	0	0	0	9.77176831	5.31952502	0	0	0	0	0	3.67460121	0	0	0	0	0	0
M.nesaea_BA907	Bahia	Camacã / BA	Serra Bonita Private Reserve	900-1200	0	0	0	0.08818658	0	61.3384521	2.3176E-07	7.59782526	13.5358067	0	0	0	7.10769804	3.64528046	0	0	0	0	2.65229621	4.03445442	0	0	0	0	0	0
M.nesaea_BA908	Bahia	Camacã / BA	Serra Bonita Private Reserve	900-1200	0	0	0	0.19041305	0	83.2554207	1.0259E-07	1.66668529	11.1654831	0	0	0	1.85920208	1.86279558	0	0	0	0	0	0	0	0	0	0	0	0
M.lysimnia589	Serra do Mar	Jundiaí / SP	Serra do Japi Biological Reserve	900-1200	0	0	0	0.14052936	0	5.31147495	0.43210003	0.35093199	0	0	0	0	0.27990906	0	0	0	0.79653893	0	0	0	0	0	0	88.4268811	0	4.26163461
M.lysimnia591	Serra do Mar	Jundiaí / SP	Serra do Japi Biological Reserve	900-1200	0	0	0	0.23464036	0	0	0.81789675	1.03102831	0	0	0	0	0	0	0	0	6.81580343	0	0	0	0	0	0	88.9122651	0	2.18836602
M.lysimnia596	Serra do Mar	Jundiaí / SP	Serra do Japi Biological Reserve	900-1200	0	0	0	0.03915117	0	6.72422316	0.07998742	0.06936629	0	0	0	0	0.09175904	0	0	0	0.85733598	0	0	0	0	0	2.28933707	86.9884168	0	2.86042307
M.lysimnia597	Serra do Mar	Jundiaí / SP	Serra do Japi Biological Reserve	900-1200	0	0	0	0.00645283	0	2.07448828	0.34946033	0.16651877	0	0	0	0	0.25697011	0	0	0	2.29751036	0	0	0	0	0	1.71138566	90.5609475	0	2.57626616
M.lysimnia636	Serra do Mar	Jundiaí / SP	Serra do Japi Biological Reserve	900-1200	0	0	0	0.10986487	0	6.59742104	0.04274752	0.07053895	0	0	0	0	0.1117124	0	0	0	0.77332049	0	0	0	0	0	0	89.9841975	0	2.31019722
M.lysimnia650	Serra do Mar	Jundiaí / SP	Serra do Japi Biological Reserve	900-1200	0	0	0	0	0	1.3992E-06	1.12946198	1.3992E-06	0	0	0	0	0.41978898	0	0	0	2.14239862	0	0	0	0	0	0	93.3740169	0	2.93433072
M.lysimnia656	Serra do Mar	Jundiaí / SP	Serra do Japi Biological Reserve	900-1200	0	0	0	0.16094917	0	14.045796	0.2052649	0.24658731	0	0	0	0	0.24358273	0	0	0	2.3324385	0	0	0	0	0	2.42759821	78.3918119	0	1.94597129
M.lysimnia_BA827	Bahia	Camacã / BA	Serra Bonita Private Reserve	900-1200	0	0	0	0	0	1.58067177	0.47791712	0.27413119	0	0	0	0	0	0	0	0	0.71741309	0	0	0.07924479	0	0	0.98682932	92.9024271	0	2.98136562
M.lysimnia_BA845	Bahia	Camacã / BA	Serra Bonita Private Reserve	900-1200	0	0	0	0	0	17.1109466	0	0.15548734	0.27432519	0	0	0	0.30716948	0	0	0	0.35723725	0	0	0.05328654	0	0	1.18812441	77.7457301	0	2.80769311
M.lysimnia_BA855	Bahia	Camacã / BA	Serra Bonita Private Reserve	900-1200	0	0	0	0	0	0.21924994	0	0.01371651	0	0	0	0	0	0	0	0	0.19125711	0	0	0.60763643	0	0	0.36583366	97.8774511	0	0.72485522
M.lysimnia_BA858	Bahia	Camacã / BA	Serra Bonita Private Reserve	900-1200	0	0	0	0	0	0.12550766	0	0	0	0	0	0	0	0	0	0	0.03738595	0	0	0	0	0	0	99.703839	0	0.13326741
M.lysimnia_BA877	Bahia	Camacã / BA	Serra Bonita Private Reserve	900-1200	0	0	0	0	0	0.34411777	1.19130692	0	0	0	0	0	0	0	0	0	1.26151335	0	0	0	0	0	0	96.5346245	0	0.66843741
M.lysimnia_BA878	Bahia	Camacã / BA	Serra Bonita Private Reserve	900-1200	0	0	0	0	0	1.95105027	0	0	0	0	0	0	0	0	0	0	0.13159952	0	0	0	0	0	0	97.6140244	0	0.30332581
M.lysimnia_BA879	Bahia	Camacã / BA	Serra Bonita Private Reserve	900-1200	0	0	0	0	0	2.17434067	0	0	0	0	0	0	0	0	0	0	0	0	0	0	0	0	0	97.402953	0	0.42270629
M.lysimnia_BA880	Bahia	Camacã / BA	Serra Bonita Private Reserve	900-1200	0	0	0	0	0	1.2737009	0.02613134	0	0	0	0	0	0	0	0	0	0.1816224	0	0	0	0	0	0	97.9720837	0	0.54646164
M.lysimnia_BA892	Bahia	Camacã / BA	Serra Bonita Private Reserve	900-1200	0	0	0	0	0	0.98577966	0	0	0	0	0	0	0	0	0	0	0	0	0	0	0	0	0	98.7128486	0	0.30137174
M.lysimnia_BA898	Bahia	Camacã / BA	Serra Bonita Private Reserve	900-1200	0	0	0	0	0	1.14553891	0.07527714	0.03599215	0	0	0	0	0.04596619	0	0	0	0	0	0	0	0	0	0	98.3244313	0	0.37279429
M.lysimnia_BA906	Bahia	Camacã / BA	Serra Bonita Private Reserve	900-1200	0	0	0	0	0	0.22879488	0	0	0	0	0	0	0.01341094	0	0	0	0.03162097	0	0	0	0	0	0	99.0613974	0	0.66477584
M.lysimnia_BA909	Bahia	Camacã / BA	Serra Bonita Private Reserve	900-1200	0	0	0	0	0	2.43979649	0	0	0	0	0	0	0	0	0	0	0	0	0	0	0	0	0	96.4758354	0	1.08436806

Table S4. Pheromone results summary

Median relative concentration values of chemical compounds identified in androconial extracts of *Mechanitis* individuals from the Brazilian Atlantic forest. Values between brackets indicate the number of analysed samples in which the compound was present, per investigated species. *RI*: Retention Index.

RI	**Compound identity or mass spectra**	***M. nesaea* N = 65**	***M. l. lysimnia* N = 19**	***M. polymnia casabranca* N = 8**
**1150**	2,6,6-Trimethyl-2-cyclohexene-1,4-dione	0.18 [15]	-	-
**1252**	*m/z:* 98, 41, 43, 69, 39	0.12 [12]	-	-
**1264**	*m/z:* 43, 41, 57, 55, 39	0.06 [8]	-	-
**1318**	4-Hydroxy-3,5,5-trimethylcyclohex-2-enone	0.45 [56]	0.12 [6]	2.19 [7]
**1365**	Eugenol	0.11 [8]	-	-
**1486**	Hydroxydanaidal	44.68 [65]	1.76 [18]	74.99 [8]
**1543**	*m/z:* 98, 43, 71, 41, 57	-	0.35 [11]	-
**1551**	Dihydroactinidiolide	0.67 [40]	0.15 [11]	-
**1588**	Methyl hydroxydanaidoate	11.81 [61]	0.27 [1]	12.48 [8]
**1599**	Hexadecane	0.77 [13]	-	-
**1619**	*m/z:* 86, 43, 71, 41, 84	0.27 [16]	-	-
**1673**	*m/z* 120, 43, 65, 44, 55	-	-	0.47 [5]
**1749**	Methyl farnesoate isomer	3.09 [61]	0.23 [9]	-
**1792**	Methyl (*E,E*)-farnesoate	2.97 [60]	-	-
**1804**	4-Hydroxy-3,5,5-trimethyl-4-[3-oxo-1-butenyl]-2-cyclohexen-1-one	0.41 [18]	-	-
**1843**	Methyl oxodanaidoate	0.77 [39]	-	-
**1852**	*m/z:* 57, 45, 55, 43, 71	2.95 [1]	0.76 [15]	-
**1950**	*m/z* 57, 43, 55, 56, 85	-	-	1.15 [6]
**2145**	*m/z* 79, 67, 55, 41, 95	2.69 [41]	-	-
**2160**	Octadecanoic acid	1.66 [48]	0.08 [3]	-
**2172**	Ethyl linolenate	-	-	3.83 [3]
**2220**	Phytol acetate <(*E*)->	-	-	2.45 [8]
**2572**	Hexacosene	4.26 [1]	1.44 [6]	-
**2675**	Heptacosene	0.27 [2]	96.27 [19]	-
**2832**	Squalene	-	-	2.25 [8]
**2873**	Nonacosene	-	1.08 [19]	-

Table S5. Reference genome statistics

Assembled chromosomes and completeness of the genomes generated in this study, with primary and alternative haplotype for each individual. Individuals with a different alternative autosome number are heterozygous for chromosomal rearrangements. All sex-chromosomes are included in the primary assembly. Estimated k-mer coverage included for PacBio, HiC-data and, where applicable, 10X-data.

Species	NCBI accession	Autos.	Sex-chr	Sex	Genome size (Mb)	In chr (Mb)	%	Scaffold N50 (Mb)	Contigs N50 (Mb)	Gaps	Scaffolds	PacBio	HiC	10X
Mec. polymnia	GCA_965197365	14	W and Z	F	295.9	293.1	99	18.4	1.4	0.03%	122	29.03	136.9	
	GCA_965197355	15			264.4	261.7	99	17.5	1.2	0.03%	104			
Mec. menapis	GCA_965234935	19	W and Z	F	305.3	301.8	99	15.7	0.5	0.08%	213	13.61	54.46	
	GCA_965234955	19			237.4	231.5	98	13.4	0.5	0.07%	140			
Mec. messenoides	GCA_959347415	14	W and Z	F	311.1	307.5	99	19.1	8.6	0.01%	102	52.83	164.6	
	GCA_959347375	15			269.0	266.2	99	18.7	5.9	0.01%	53			
Mec. macrinus	GCA_965612265	21	W1, W2, W3, Z1 and Z2	F	320.1	319.4	100	13.6	6.6	0.01%	53	12.2	73.91	
	GCA_965612315	21			241.6	241.0	100	12.5	6.6	0.00%	45			
Mec. mazaeus	GCA_959347395	12	W1, W2, Z1, Z2	F	318.5	315.7	99	20.9	9.3	0.01%	88	45.32	196.9	
	GCA_959347365	12			244.5	242.9	99	20.6	15.9	0.00%	46			
Mel. ludovica	GCA_965234865	21	Z|Z	M	661.4	627.9	95	33.1	4.6	0.01%	176	57.19	60.47	
	GCA_965234875	23			647.8	625.1	97	30.3	3.8	0.01%	282			
Mel. isocomma	GCA_965282635	13	Z|Z1 and Z2	M	527.6	519.4	98	39.4	4.9	0.01%	78	21.4	33.3	
	GCA_965282685	14			504.8	498.2	99	34.9	4.1	0.01%	94			
Mel. mothone	GCA_965197345	13	W1, W2, Z1 and Z2	F	559.0	552.3	99	37.0	5.0	0.01%	185	20.74	87.54	
	GCA_965197325	14			461.6	457.7	99	35.6	4.4	0.01%	92			
Mel. marsaeus	GCA_918358865	13	W and Z	F	516.2	510.6	99	39.5	17.2	0.00%	143	25.7 (CLR)	19.45	15.11
	GCA_918361175	14			490.1	483.7	99	38.8	22.0	0.00%	141			
Mel. menophilus	GCA_918358695	19	W, Z1, Z2	F	541.2	536.4	99	22.9	14.5	0.00%	130	24.86 (CLR)	20.19	11.03
	GCA_918361105	21			453.2	449.8	99	21.2	10.7	0.00%	81			

Table S6. BUSCO results

Result from BUSCO analysis of the genomes included in the analyses. All results are in percent of the total number of genes (5286 genes) in the BUSCO *lepidoptera_odb10* database.

Species		Complete	Single	Duplicated	Fragmented	Missing	Internal stop codon (%)
Mec. polymnia	Hap1	99	98.1	0.9	0.5	0.5	2.7
	Hap2	91.8	91.3	0.5	0.5	7.7	2.9
Mec. menapis	Hap1	96.6	96.1	0.5	1.2	2.2	3.4
	Hap2	75.4	74.9	0.5	1.3	23.3	3.5
Mec. messenoides	Hap1	99.2	98.7	0.5	0.5	0.3	2.8
	Hap2	91.9	91.4	0.5	0.5	7.6	2.9
Mec. macrinus	Hap1	99.1	91.6	7.5	0.5	0.4	2.8
	Hap2	79.3	78.9	0.4	0.4	20.3	2.7
Mec. mazaeus	Hap1	99.2	89.3	9.9	0.5	0.3	2.6
	Hap2	82.5	82.2	0.3	0.4	17.1	2.7
Mel. ludovica	Hap1	98.8	97.2	1.6	0.7	0.5	2.6
	Hap2	98.4	97.4	1	0.8	0.8	2.6
Mel. isocomma	Hap1	98.1	96.3	1.8	0.6	1.3	2.7
	Hap2	97.9	96.6	1.3	0.7	1.4	2.7
Mel. mothone	Hap1	98.9	90.6	8.3	0.6	0.5	2.6
	Hap2	85.5	84.8	0.7	0.5	14	2.5
Mel. marsaeus	Hap1	99	98.1	0.9	0.6	0.4	2.8
	Hap2	93.1	92.4	0.7	0.5	6.4	2.6
Mel. menophilus	Hap1	99	87.3	11.7	0.6	0.4	2.7
	Hap2	82.6	81.8	0.8	0.5	16.9	2.7

Table S7. Gene count per conserved syntenic block

Total number of BUSCO-genes included per taxa, mean, standard deviation, median and maximum number of genes per ancestral syntenic block per taxa.

Species	Total nr genes	Mean	SD	Median	Max
Melitaea cinxia	5076.00	163.74	77.02	167	315
Danaus plexippus	4963.00	160.10	75.98	164	311
Mec. macrinus	5390.00	45.29	42.93	34	226
Mec. mazaeus	5506.00	46.27	45.08	34	226
Mec. menapis	4754.00	45.28	45.29	32	230
Mec. messenoides	5014.00	45.58	44.78	31.5	244
Mec. polymnia	4998.00	44.23	42.97	32	226
Mel. ludovica	4956.00	39.02	31.10	32	225
Mel. isocomma	4899.00	42.60	33.68	33	226
Mel. mothone	5383.00	44.12	34.14	35	226
Mel. menophilus	5532.00	42.23	33.42	33	227
Mel. marsaeus	4998.00	42.72	33.88	34	227

Table S8. Sex-chromosomes and autosomal polymorphisms.

Constituent parts of the sex-chromosomes (Chr) in the ten *Mechanitis* and *Melinaea* genome assemblies based on BUSCO-genes and whole genome alignment (W). Fusions of autosomes or autosomal parts (Ancestral element) with the canonical Z-chromosome (Z*) are indicated with "+" signs and the number of the ancestral autosome. The "w*" in the W-ancestral column is a common W-element in all *Mechanitis* assemblies with high sequence homology but no BUSCO-genes. Autosomal polymorphic fusion-fissions with homologous chromosomes separated with "=" and the split chromosomes in one haplotype are separated by "|". Ancestral elements in parenthesis ( ) are only found in the split chromosomes, elements in square brackets [ ] are only found in the fused chromosome and "|" represent the breakpoint between the split chromosomes.

**Species**	**Z**	**Ancestral elements**	**W**	**Ancestral elements**	**Autosomal polymorphism**	**Ancestral elements**
Melinaea Ludovica (male)	Z	Z*+10	NA	NA	2 = 13 | 17	21+20+2+3+10 | 8+22
					7 = 20 | 18	11+2+26+5 | 14+17
					10 = 23 | 14	12+8+16+1+21 | 21+4+10
					1,18,14,16 = 1,10,12	Complex rearrangement
Melinaea isocomma (male)	Z = Z2 | Z1	12+13+16+[24]+ 8+20+11+9 | Z*+10	NA	NA	1 = 3 |13	17+18+9+11+7+27+13+2 | 8+4+20 +(11)
Melinaea mothone	Z1 | Z2	Z*+10 | 27+7+17+4+21+6	W1 | W2	27+7+17+4+10 | 21+6	2 = 11 | 14	1+21+20+2+3+15 | 18+12+17+(2)
Melinaea menophilus	Z1 | Z2	Z*+10 | 3+5+6+16+14+12+18	W	10+3+5+6+16+14+12+18	1 = 18 | 4	19+24+4+14 |14+30+6+3+11+2+26
					2 = 21 | 13	27+7+17+[18]+4+(3) | 11+9+[Z]+(18+17)
Melinaea marsaeus	Z	Z*+10	W	10	2 = 10 | 14	3+5+6+16+14+12+18 | 4+17+7+27
Mechanitis macrinus	Z1 | Z2	Z*+10+6 | 11+10+8+6+5+24	W1 | W2 | W3	w* | 10+8+6+5+24 | 11	Not found	
Mechanitis mazaeus	Z1 | Z2	Z*+10+6 | 3+19+10+8+6+5+24	W1 | W2	w*+10+8+6+5+24 | 3+19	Not found	
Mechanitis menapis	Z	Z*+10+6	W	w*	Not found	
Mechanitis messenoides	Z	Z*+10+6	W	w*	1 = 6 | 14	5+12+2+3+28+23+20+19 | 7+8+10+6+(5)+24
Mechanitis polymnia	Z	Z*+10+6	W	w*+8	1 = 14 | 13	19+24+20+4+25+17 | (4)+9+3+28

Table S9. Result table for randomization test

Mean values across *Mechanitis* and *Melinaea* genomes and within breakpoint regions (break) for a) F_{ST}, D_{XY} and b) π. Result of a randomisation test of observed population statistic within breakpoint regions and permutated regions across the genome. Z-score and P-value at 5% significance level (lesser than or greater than randomised sample). Bold represent significant results after Bonferroni correction. The randomisation test was not performed in three of the comparisons due to limited number of informative sites.

A)

Comparison Mec	F_{ST}	SD	F_{ST} break	SD	P-value	Z-score	D_{XY}	SD	D_{XY} break	SD	P-value	Z.score
Mec. macrinus vs Mec. mazaeus	9.84E-02	7.32E-02	2.06E-01	1.18E-01	**2.00E-05**	**6.238**	1.32E-02	4.46E-03	1.26E-02	5.60E-03	1.99E-02	2.01E+00
Mec. macrinus vs Mec. menapis	2.25E-01	9.61E-02	2.80E-01	1.18E-01	**2.20E-04**	**4.142**	1.89E-02	5.06E-03	2.32E-02	6.93E-03	**2.00E-05**	**4.63E+00**
Mec. macrinus vs Mec. messenoides	2.35E-01	1.01E-01	3.63E-01	1.37E-01	**2.00E-05**	**7.576**	1.92E-02	5.13E-03	2.39E-02	8.02E-03	**2.00E-05**	**6.06E+00**
Mec. macrinus vs Mec. polymna	2.71E-01	9.51E-02	3.56E-01	1.30E-01	**2.00E-05**	**5.064**	1.94E-02	5.33E-03	2.05E-02	5.55E-03	5.19E-02	1.65E+00
Mec. mazaeus vs Mec. menapis	2.22E-01	9.27E-02	3.09E-01	1.21E-01	**2.00E-05**	**5.154**	1.86E-02	4.88E-03	2.14E-02	6.39E-03	**1.02E-03**	**3.15E+00**
Mec. mazaeus vs Mec. messenoides	2.40E-01	1.07E-01	3.94E-01	1.32E-01	**2.00E-05**	**7.58**	1.88E-02	4.95E-03	2.13E-02	8.01E-03	**2.60E-04**	**3.45E+00**
Mec. mazaeus vs Mec. polymnia	2.60E-01	1.04E-01	3.74E-01	1.32E-01	**2.00E-05**	**5.162**	1.90E-02	5.06E-03	1.90E-02	4.39E-03	4.12E-01	-2.27E-01
Mec. menapis vs Mec. messenoides	2.21E-01	7.63E-02	2.92E-01	1.06E-01	**2.00E-05**	**5.051**	1.75E-02	5.01E-03	1.85E-02	6.69E-03	7.98E-03	2.40E+00
Mec. menapis vs Mec. polymnia	2.55E-01	8.33E-02	3.25E-01	1.23E-01	**2.00E-05**	**5.332**	1.98E-02	5.29E-03	2.15E-02	4.95E-03	5.41E-02	1.62E+00
Mec. messenoides vs Mec. polymnia	2.42E-01	9.23E-02	3.34E-01	1.16E-01	**2.00E-05**	**5.242**	1.86E-02	5.21E-03	1.79E-02	4.82E-03	1.23E-01	-1.14E+00
**Comparison Mel**	**F_{ST}**	**SD**	**F_{ST} break**	**SD**	**P-value**	**Z-score**	**D_{XY}**	**SD**	**D_{XY} break**	**SD**	**P-value**	**Z.score**
Mel. isocomma vs Mel. marsaeus	2.73E-01	2.13E-01	2.69E-01	1.86E-01	NA	NA	2.57E-02	8.48E-03	2.16E-02	9.00E-03	NA	NA
Mel. isocomma vs Mel. menophilus	3.11E-01	2.02E-01	4.94E-01	1.92E-01	1.67E-02	2.576	2.79E-02	9.37E-03	3.42E-02	1.11E-02	1.42E-02	2.58E+00
Mel. isocomma vs Mel. mothone	3.13E-01	1.82E-01	1.98E-01	4.55E-02	NA	NA	3.07E-02	8.52E-03	2.31E-02	6.22E-03	NA	NA
Mel. ludovica vs Mel. isocomma	4.00E-01	2.57E-01	2.53E-01	4.95E-01	**6.60E-04**	**-3.931**	3.54E-02	7.64E-03	4.36E-02	7.23E-03	**2.00E-05**	**4.64E+00**
Mel. ludovica vs Mel. marsaeus	3.75E-01	2.06E-01	2.98E-01	3.39E-01	**3.78E-03**	**-2.634**	3.26E-02	8.03E-03	4.27E-02	8.53E-03	**2.00E-05**	**5.48E+00**
Mel. ludovica vs Mel. menophilus	3.65E-01	1.95E-01	3.41E-01	2.61E-01	1.18E-01	-1.091	3.42E-02	8.26E-03	4.13E-02	8.25E-03	**2.00E-05**	**4.65E+00**
Mel. ludovica vs Mel. mothone	3.30E-01	1.61E-01	3.79E-01	1.82E-01	1.04E-01	1.297	3.57E-02	7.66E-03	4.46E-02	7.74E-03	**2.00E-05**	**5.38E+00**
Mel. menophilus vs Mel. marsaeus	1.39E-01	1.00E-01	2.52E-01	1.30E-01	1.13E-02	3.478	1.77E-02	7.02E-03	1.85E-02	6.61E-03	2.90E-01	5.44E-01
Mel. mothone vs Mel. marsaeus	8.79E-02	8.07E-02	1.19E-01	1.31E-01	NA	NA	1.85E-02	6.61E-03	1.74E-02	9.14E-03	NA	NA
Mel. mothone vs Mel. menophilus	1.20E-01	1.03E-01	2.52E-01	1.15E-01	5.22E-03	3.791	2.06E-02	7.57E-03	2.09E-02	6.58E-03	4.17E-01	2.08E-01

B)

Comparison Mec	Species	π	SD	π break	SD	P-value	Z-score
Mec. macrinus vs Mec. mazaeus	*Mec. macrinus*	1.09E-02	4.28E-03	8.61E-03	3.71E-03	8.41E-02	-1.389
Mec. macrinus vs Mec. mazaeus	*Mec. mazaeus*	1.12E-02	4.35E-03	8.13E-03	4.13E-03	1.29E-01	-1.137
Mec. macrinus vs Mec. menapis	*Mec. macrinus*	1.09E-02	4.27E-03	9.02E-03	4.15E-03	**3.86E-03**	**-2.673**
Mec. macrinus vs Mec. menapis	*Mec. menapis*	1.16E-02	3.44E-03	1.34E-02	5.41E-03	**5.80E-04**	**3.243**
Mec. macrinus vs Mec. messenoides	*Mec. macrinus*	1.09E-02	4.27E-03	8.94E-03	4.20E-03	**4.20E-04**	**-3.334**
Mec. macrinus vs Mec. messenoides	*Mec. messenoides*	1.07E-02	3.98E-03	8.58E-03	3.74E-03	**2.20E-04**	**-3.654**
Mec. macrinus vs Mec. polymna	*Mec. macrinus*	1.09E-02	4.28E-03	9.01E-03	3.86E-03	**1.46E-03**	**-3.021**
Mec. macrinus vs Mec. polymna	*Mec. polymniaEast*	1.13E-02	4.10E-03	9.37E-03	3.60E-03	5.26E-03	-2.515
Mec. mazaeus vs Mec. menapis	*Mec. mazaeus*	1.13E-02	4.34E-03	7.91E-03	4.00E-03	**4.20E-04**	**-3.483**
Mec. mazaeus vs Mec. menapis	*Mec. menapis*	1.16E-02	3.47E-03	1.19E-02	4.18E-03	2.69E-01	0.606
Mec. mazaeus vs Mec. messenoides	*Mec. mazaeus*	1.13E-02	4.34E-03	8.38E-03	4.33E-03	**2.00E-05**	**-4.121**
Mec. mazaeus vs Mec. messenoides	*Mec. messenoides*	1.08E-02	3.98E-03	7.33E-03	2.86E-03	**2.00E-05**	**-4.696**
Mec. mazaeus vs Mec. polymnia	*Mec. mazaeus*	1.13E-02	4.35E-03	8.15E-03	3.81E-03	**2.60E-04**	**-3.759**
Mec. mazaeus vs Mec. polymnia	*Mec. polymniaEast*	1.13E-02	4.10E-03	8.55E-03	2.97E-03	**6.00E-05**	**-3.813**
Mec. menapis vs Mec. messenoides	*Mec. menapis*	1.16E-02	3.48E-03	1.19E-02	4.00E-03	8.88E-02	1.356
Mec. menapis vs Mec. messenoides	*Mec. messenoides*	1.07E-02	3.98E-03	7.51E-03	3.63E-03	**3.02E-03**	**-2.898**
Mec. menapis vs Mec. polymnia	*Mec. menapis*	1.16E-02	3.49E-03	1.17E-02	3.26E-03	4.99E-01	8.00E-03
Mec. menapis vs Mec. polymnia	*Mec. polymniaEast*	1.13E-02	4.10E-03	8.97E-03	3.17E-03	**3.80E-04**	**-3.23**
Mec. messenoides vs Mec. polymnia	*Mec. messenoides*	1.07E-02	3.98E-03	7.63E-03	3.12E-03	**2.00E-05**	**-4.936**
Mec. messenoides vs Mec. polymnia	*Mec. polymniaEast*	1.13E-02	4.10E-03	9.04E-03	3.14E-03	**5.80E-04**	**-3.145**

Comparison Mel	Species	π	SD	π break	SD	P-value	Z-score
Mel. isocomma vs Mel. marsaeus	*Mel. isocomma*	1.29E-02	6.67E-03	6.81E-03	4.11E-03	NA	NA
Mel. isocomma vs Mel. marsaeus	*Mel. marsaeus*	1.24E-02	5.19E-03	1.10E-02	6.47E-03	NA	NA
Mel. isocomma vs Mel. menophilus	*Mel. isocomma*	1.29E-02	6.67E-03	8.92E-03	5.43E-03	1.80E-02	-2.152
Mel. isocomma vs Mel. menophilus	*Mel. menophilus*	1.40E-02	7.14E-03	9.12E-03	3.86E-03	5.42E-03	-2.675
Mel. isocomma vs Mel. mothone	*Mel. isocomma*	1.29E-02	6.67E-03	1.52E-02	6.08E-03	NA	NA
Mel. isocomma vs Mel. mothone	*Mel. mothone*	1.76E-02	7.05E-03	1.56E-02	7.07E-05	NA	NA
Mel. ludovica vs Mel. isocomma	*Mel. isocomma*	1.29E-02	6.66E-03	8.95E-03	6.19E-03	1.98E-01	0.77
Mel. ludovica vs Mel. isocomma	*Mel. ludovica*	7.32E-03	4.99E-03	7.54E-03	3.45E-03	**8.20E-04**	**-3.472**
Mel. ludovica vs Mel. marsaeus	*Mel. ludovica*	7.32E-03	4.99E-03	7.54E-03	3.45E-03	2.06E-01	0.743
Mel. ludovica vs Mel. marsaeus	*Mel. marsaeus*	1.24E-02	5.17E-03	8.86E-03	5.12E-03	**4.60E-04**	**-3.929**
Mel. ludovica vs Mel. menophilus	*Mel. ludovica*	7.32E-03	5.00E-03	7.43E-03	3.19E-03	2.58E-01	0.567
Mel. ludovica vs Mel. menophilus	*Mel. menophilus*	1.41E-02	7.12E-03	1.06E-02	6.67E-03	**1.02E-03**	**-3.203**
Mel. ludovica vs Mel. mothone	*Mel. ludovica*	7.32E-03	4.99E-03	7.52E-03	3.34E-03	2.01E-01	0.763
Mel. ludovica vs Mel. mothone	*Mel. mothone*	1.77E-02	7.01E-03	1.38E-02	7.90E-03	**1.22E-03**	**-3.274**
Mel. menophilus vs Mel. marsaeus	*Mel. marsaeus*	1.24E-02	5.18E-03	1.06E-02	5.68E-03	5.26E-03	-2.63E+00
Mel. menophilus vs Mel. marsaeus	*Mel. menophilus*	1.40E-02	7.14E-03	9.13E-03	4.30E-03	9.77E-02	-1.331
Mel. mothone vs Mel. marsaeus	*Mel. marsaeus*	1.24E-02	5.19E-03	1.11E-02	6.71E-03	NA	NA
Mel. mothone vs Mel. marsaeus	*Mel. mothone*	1.76E-02	7.05E-03	1.26E-02	6.31E-03	NA	NA
Mel. mothone vs Mel. menophilus	*Mel. menophilus*	1.40E-02	7.14E-03	9.17E-03	3.83E-03	2.68E-02	-1.986
Mel. mothone vs Mel. menophilus	*Mel. mothone*	1.76E-02	7.05E-03	1.33E-02	5.99E-03	1.09E-02	-2.351

Supplementary Information References

1. R. M. Fox, A monograph of the Ithomiidae (Lepidoptera). Part II. The Tribe Melinaeini Clark. *Transactions of the American Entomological Society* **86**, 109–171 (1960).

2. R. M. Fox, A monograph of the Ithomiidae (Lepidoptera). Part III. The Tribe Mechanitini. *Memoirs of the American Entomological Society* **22** (1967).

3. K. S. Brown, Geographical patterns of evolution in Neotropical Lepidoptera: differentiation of the species of Melinaea and Mechanitis (Nymphalidae, Ithomiinae). *Systematic Entomology* **2**, 161–197 (1977).

4. J. F. Le Crom, W. Winhard, *Mariposas de Colombia Tomo 3: Nymphalidae (partim): Libytheinae y Danainae* (CARLEC, 2024).

5. N. Chazot, *et al.*, Renewed diversification following Miocene landscape turnover in a Neotropical butterfly radiation. *Global Ecology and Biogeography* **28**, 1118–1132 (2019).

6. M. McClure, B. Dutrillaux, A.-M. Dutrillaux, V. Lukhtanov, M. Elias, Heterozygosity and Chain Multivalents during Meiosis Illustrate Ongoing Evolution as a Result of Multiple Holokinetic Chromosome Fusions in the Genus *Melinaea* (Lepidoptera, Nymphalidae). *Cytogenet Genome Res* **153**, 213–222 (2017).

7. S. Scudder, Historical sketch of the generic names proposed for butterflies. *Proceedings of the American Academy of Arts and Sciences* **10**, 91–293 (1875).

8. A. Hemming, in *The Generic Names of the Holarctic Butterflies*, (Richard Clay and Sons, 1934), pp. 1758–1863.

9. A. Hemming, On the types of certain butterfly genera. *Entomologist* **66**, 196–200 (1933).

10. R. Vane-Wright, Oxford University Museum of Natural History, *Iconotypes. A compendium of butterflies & moths or: Jones's Icones complete. An enhanced facsimile.* (Thames & Hudson; University of California Press, 2021).

11. G. Lamas, Los Danainae e Ithomiinae descritos por R. Haensch (Lepidoptera: Nymphalidae). *Shilap* **22**, 271–297 (1994).

12. G. Lamas, Checklist: Part 4A. Hesperioidea - Papilionoidea. *Atlas of Neotropical Lepidoptera* **5** (2004).

13. G. Lamas, Los Ithomiinae descritos por J. Röber (Lepidoptera: Nymphalidae). *Shilap* **20**, 175–178 (1992).

14. A. D. Warren, *et al.*, Butterflies of America. *www.butterfliesofamerica.com* (2024). [Accessed 4 October 2024].

15. G. Lamas, Las mariposas sudamericanas descritas por Ambrosio Fernández (Lepidoptera: Papilionoidea). *Shilap* **27**, 131–137 (1999).

16. G. Lamas, Brother Apolinar Maria (1867-1949) and his contributions to Colombian lepidopterology. *Journal of the Lepidopterists' Society* **33**, 65–67 (1979).

17. R. I. Hill, *et al.*, Ecologically relevant cryptic species in the highly polymorphic Amazonian butterfly Mechanitis mazaeus s.l. (Lepidoptera: Nymphalidae; Ithomiini). *Biological Journal of the Linnean Society* **106**, 540–560 (2012).

18. A. Neild, *The Butterflies of Venezuela. Part 2: Nymphalidae II (Acraeinae, Libytheinae, Nymphalinae, Ithomiinae, Morphinae). A comprehensive guide to the identification of adult Nymphalidae, Papilionidae, and Pieridae.* (Meridian Publications, 2008).

19. G. Lamas, The identity of Mechanitis doryssus Bates and M. saturata Godman (Lepidoptera: Nymphalidae, Ithomiinae). *Revista peruana de Entomología* **30**, 48–50 (1988).

20. R. D’Almeida, Notas sinonímicas sôbre Ithomiidae (Lepidoptera, Rhopalocera). *Boletim do Museu Nacional (Zoologia)* **143**, 1–18 (1956).

21. M. R. Honey, M. J. Scoble, Linnaeus’s butterflies (Lepidoptera: Papilionoidea and Hesperioidea). *Zoological Journal of the Linnean Society* **132**, 277–399 (2001).

22. A. Hemming, in *Annotationes Lepidopterologicae*, (Hepburn & Sons, 1964), pp. 113–151.

23. R. M. Fox, Additional notes on Melinaea Hübner (Lepidoptera: Ithomiidae). *Proceedings of the Royal Entomological Society of London (B)* **34**, 77–82 (1965).

24. H. Jordan, List of the butterflies figured on Plate IV. *Novitates zoologicae* **30**, 270 (1923).

25. W. Winhard, Neue Tagfalterunterarten aus Kolumbien, Ecuador und Bolivien (Lepidoptera: Nymphalidae). *Atalanta* **52**, 478–482 (2021).

26. H. de Lesse, Formules Chromosomiques de Quelques Lépidoptères Rhopalocères de Guyane. *Annales de la Société entomologique de France (N.S.)* **6**, 849–855 (1970).

27. W. Kaye, The types of the ithomiine butterflies Melinaea mneme Linn., and M. crameri Godm. *Proceedings of the entomological Society of London* **1/2**, xxii–xxiii (1925).

28. J. Mallet, N. Besansky, M. W. Hahn, How reticulated are species? *BioEssays* **38**, 140–149 (2015).

29. J. M. Walker, *et al.*, Common misconceptions of speciation. *Evolutionary Journal of the Linnean Society* kzae029 (2024). https://doi.org/10.1093/evolinnean/kzae029.

30. L. L. Mota, A. H. B. Rosa, L. R. Vasconcellos, K. R. Willmott, A. V. L. Freitas, A new subspecies of Mechanitis lysimnia from southern Amazonia (Nymphalidae: Danainae: Ithomiini). (2022). https://doi.org/10.5281/zenodo.6604683.

31. K. S. Brown, B. V. Schoultz, E. Suomalainen, Chromosome evolution in Neotropical Danainae and Ithomiinae (Lepidoptera). *Hereditas* **141**, 216–236 (2004).

32. M. Carvalho, A. Victor, L. Freitas, E. Barbosa, Immature stages of Mechanitis polymnia casabranca (Nymphalidae, Danainae). (2019). https://doi.org/10.5281/zenodo.2650300.

33. P. Stamm, F. Mann, M. McClure, M. Elias, S. Schulz, Chemistry of the Androconial Secretion of the Ithomiine Butterfly Oleria onega. *J Chem Ecol* **45**, 768–778 (2019).

34. F. Mann, *et al.*, 3-Acetoxy-fatty acid isoprenyl esters from androconia of the ithomiine butterfly *Ithomia salapia*. *Beilstein J. Org. Chem.* **16**, 2776–2787 (2020).

35. M. McClure, *et al.*, Does divergent selection predict the evolution of mate preference and reproductive isolation in the tropical butterfly genus *Melinaea* (Nymphalidae: Ithomiini)? *Journal of Animal Ecology* **88**, 940–952 (2019).

36. S. Schulz, *et al.*, Semiochemicals derived from pyrrolizidine alkaloids in male ithomiine butterflies (Lepidoptera: Nymphalidae: Ithomiinae). *Biochemical Systematics and Ecology* **32**, 699–713 (2004).

37. J. Vasconcellos-Neto, K. S. Brown, Interspecific Hybridization in Mechanitis Butterflies (Ithomiinae): a Novel Pathway for the Breakdown of Isolating Mechanisms. *Biotropica* **14**, 288 (1982).

38. H. van D. Dool, P. D. Kratz, A generalization of the retention index system including linear temperature programmed gas-liquid partition chromatography. *Journal of Chromatography A* **11**, 463–471 (1963).

39. S. Schulz, A. Möllerke, MACE – An Open Access Data Repository of Mass Spectra for Chemical Ecology. *J Chem Ecol* **48**, 589–597 (2022).

40. R Core Team, A language and environment for statistical computing. *R foundation for statistical computing* (2024). https://doi.org/10.1080/01621459.1972.10481279.

41. A. Denton, H. Yatsenko, J. Jay, C. Howard, Sanger Tree of Life Wet Laboratory Protocol Collection v1. [Preprint] (2023). Available at: https://protocols.cloud/view/sanger-tree-of-life-wet-laboratory-protocol-collec-cy5rxy56 [Accessed 23 June 2024].

42. H. Cheng, G. T. Concepcion, X. Feng, H. Zhang, H. Li, Haplotype-resolved de novo assembly using phased assembly graphs with hifiasm. *Nat Methods* **18**, 170–175 (2021).

43. D. Guan, *et al.*, Identifying and removing haplotypic duplication in primary genome assemblies. *Bioinformatics* **36**, 2896–2898 (2020).

44. C. Zhou, S. A. McCarthy, R. Durbin, YaHS: yet another Hi-C scaffolding tool. *Bioinformatics* **39**, btac808 (2023).

45. D.-L. Pointon, W. Eagles, Y. Sims, M. Muffato, P. Surana, sanger-tol/treeval: v1.0.0 – Ancient Atlantis. (2023).

46. P. Kerpedjiev, *et al.*, HiGlass: web-based visual exploration and analysis of genome interaction maps. *Genome Biol* **19**, 125 (2018).

47. P. Surana, M. Muffato, G. Qi, sanger-tol/readmapping: v1.1.0 – Hebridean Black. (2023).

48. M. Muffato, C. Sadasivan Baby, P. Surana, sanger-tol/genomenote v1.0.dev. (2023).

49. M. Muffato, *et al.*, sanger-tol/blobtoolkit: v0.3.0 – Poliwag. (2024).

50. M. Doré, *et al.*, Anthropogenic pressures coincide with Neotropical biodiversity hotspots in a flagship butterfly group. *Diversity and Distributions* **28**, 2912–2930 (2022).
